# A Comprehensive Analysis of *In Vitro* and *In Vivo* Genetic Fitness of *Pseudomonas aeruginosa* Using High-Throughput Sequencing of Transposon Libraries

**DOI:** 10.1371/journal.ppat.1003582

**Published:** 2013-09-05

**Authors:** David Skurnik, Damien Roux, Hugues Aschard, Vincent Cattoir, Deborah Yoder-Himes, Stephen Lory, Gerald B. Pier

**Affiliations:** 1 Division of Infectious Diseases, Department of Medicine, Brigham and Women's Hospital, Harvard Medical School, Boston, Massachusetts, United States of America; 2 Department of Epidemiology, Harvard School of Public Health, Boston, Massachusetts, United States of America; 3 Department of Microbiology and Immunobiology, Harvard Medical School, Boston, Massachusetts, United States of America; Yale University, United States of America

## Abstract

High-throughput sequencing of transposon (Tn) libraries created within entire genomes identifies and quantifies the contribution of individual genes and operons to the fitness of organisms in different environments. We used insertion-sequencing (INSeq) to analyze the contribution to fitness of all non-essential genes in the chromosome of *Pseudomonas aeruginosa* strain PA14 based on a library of ∼300,000 individual Tn insertions. *In vitro* growth in LB provided a baseline for comparison with the survival of the Tn insertion strains following 6 days of colonization of the murine gastrointestinal tract as well as a comparison with Tn-inserts subsequently able to systemically disseminate to the spleen following induction of neutropenia. Sequencing was performed following DNA extraction from the recovered bacteria, digestion with the MmeI restriction enzyme that hydrolyzes DNA 16 bp away from the end of the Tn insert, and fractionation into oligonucleotides of 1,200–1,500 bp that were prepared for high-throughput sequencing. Changes in frequency of Tn inserts into the *P. aeruginosa* genome were used to quantify *in vivo* fitness resulting from loss of a gene. 636 genes had <10 sequencing reads in LB, thus defined as unable to grow in this medium. During *in vivo* infection there were major losses of strains with Tn inserts in almost all known virulence factors, as well as respiration, energy utilization, ion pumps, nutritional genes and prophages. Many new candidates for virulence factors were also identified. There were consistent changes in the recovery of Tn inserts in genes within most operons and Tn insertions into some genes enhanced *in vivo* fitness. Strikingly, 90% of the non-essential genes were required for *in vivo* survival following systemic dissemination during neutropenia. These experiments resulted in the identification of the *P. aeruginosa* strain PA14 genes necessary for optimal survival in the mucosal and systemic environments of a mammalian host.

## Author Summary

To determine the contribution of all non-essential genes of *Pseudomonas aeruginosa* to overall fitness in laboratory and infectious settings, we used a high throughput sequencing method that quantitatively identifies the bacterial genes interrupted by random insertion of transposons (Tn) into the genome of strain PA14. This Tn-library was used to colonize the gastrointestinal tract of mice for 6 days after which systemic spread was induced by making mice neutropenic. DNA from the bacteria recovered from the cecum of colonized mice and spleens of systemically infected mice underwent high-throughput sequencing leading to identification of all non-essential Tn-interrupted genes whose levels were either increased or decreased in these selective situations compared to growth in a laboratory medium. A complete dataset was generated that yielded a comprehensive view of the contribution of all non-essential genes of *P.* aeruginosa to genetic fitness in *in vivo* settings.

## Introduction

The complex interaction of a pathogenic bacterium with a host leading to disease can be viewed as the coordinated and highly-regulated actions of a multitude of factors that allows the infecting organisms to successfully colonize tissue, occasionally disseminate and avoid the activities of host defense mechanisms. In each setting, individual or combinations of specific genetically-encoded factors contribute to the overall fitness of an organism and its ability to survive within a specific environment. Tools and strategies of bacterial genetics, particularly the ability to engineer isogenic mutants, have been extensively exploited for precise determinations of the requirements for particular gene products (including those providing various sensory and regulatory inputs) at diverse stages of the infectious process. More recently, DNA microarray based methods, such as signature-tagged mutagenesis [1], [2], [3] or transposon site hybridization [4], [5], have been utilized to determine the importance of individual genes in the infection process based on the negative selection of mutants. Such genome-wide approaches provide another level of depth for understanding the role of virulence factors in the host, since they analyze the growth phenotype of individual bacterial mutants in the context of the entire population, i.e., in cells surrounded by otherwise phenotypically wild type (WT) siblings.

The availability of high-throughput DNA sequencing technologies makes it feasible to obtain millions of DNA sequences from a single microbial sample. This tool has emerged as a major means to detect variations in genetic fitness of individual mutants in a population undergoing selection in infected hosts. By preparing highly saturated random transposon (Tn) insertion libraries using a specifically designed Tn followed by ascertaining the site of insertion via sequencing of the Tn junctions within the chromosomal DNA (variably referred to as Tn-seq or INSeq) [6], [7], [8], unique insights into the role of individual virulence factors and their regulators in the infectious process can be obtained. The basic principles and applications of the INSeq methodology have been published [6]. Fitness, as determined from INSeq results, can identify negatively selected phenotypes, presumably due to mutational inactivation of genes whose products are required for optimal growth and/or survival within the host. A large number of these phenotypes represent virulence factors or their regulators. Moreover, positively selected phenotypes whose loss enhances virulence in the host could indicate genes that repress the expression of virulence determinants or genes affecting production of conserved microbe-associated molecules such as flagellin that are recognized by the host defense mechanisms and increase resistance to infection. Additionally, the INSeq technology can identify genes involved in other novel and important aspects of microbial virulence, including those coordinately regulated both within and outside of defined operons. Furthermore, studies of fitness can lead to improved gene annotation. By comparing quantitative levels of mutated genes between input and output populations that contribute to specific phenotypes (inhibited or enhanced growth in a particular environment), logical follow-up studies can be carried out to determine the biological role played by the products of genes of unknown function. Finally, by recognition of co-selected mutations with comparable quantitative changes in the occurrence of Tn insertions in a population undergoing selection, relationships of shared or related biological function can be identified.

We applied the INSeq approach to analyze a comprehensive library of ∼300,000 mariner Tn insertions in the extensively characterized *Pseudomonas aeruginosa* strain PA14 [9] known for its high virulence in numerous models due to the production of the ExoU cytotoxin and carriage of two important pathogenicity islands, but also containing some endogenous mutations such as in the *ladS* regulatory gene whose dysfunction also increases virulence [10]. We utilized a well-established mouse infection model that strongly mimics the course of human infection in patients with cancer and bone-marrow transplantation [11], [12], [13] to determine factors needed for mucosal colonization and systemic dissemination following induction of neutropenia [14], [15]. When compared to the input mutant population, many of the Tn insertions underrepresented in the pool of colonizing organisms were in genes for well-established virulence factors, which not only validated the overall approach and its accuracy but also allowed a unique and comprehensive view of all the known virulence factors of *P. aeruginosa* (http://www.mgc.ac.cn/VFs/main.htm). The results presented here demonstrate the power of comprehensive, genome-wide approaches, such as INSeq in an established animal model of infection, for uncovering finer details of processes that bacteria deploy during establishment, progression and outcome of an infection. Importantly, this work and similar studies could facilitate assignments of new biological activities to proteins of unknown functions leading to a more complete understanding of the complex biological processes taking place during host-pathogen interactions.

## Results

### Generation of the *P. aeruginosa* PA14 Tn insertion bank and determination of fitness in mouse colonization and dissemination models

**Figure 1:**
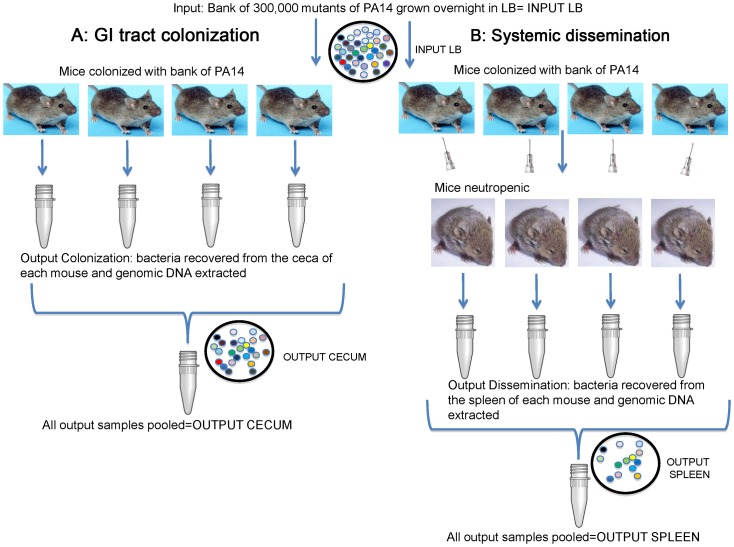
Animal models used. A: GI tract colonization. Mice (n = 4) received streptomycin and penicillin in sterile drinking water for 5 days to clear endogenous microbiota. Then the PA14 Tn insertion library grown overnight in LB containing gentamicin (input LB) was added to sterile water containing penicillin and gentamicin, renewed after 72 h, and administered to mice over the course of 6 days. The drinking water was changed for sterile water containing penicillin and gentamicin and administered for another 24 h. Ceca were then harvested for bacterial recovery. B: Systemic dissemination. Colonized mice (n = 4) were injected with 250 µg of a neutrophil-depleting monoclonal antibody, RB6, to induce a deep neutropenia the same day sterile water was given. Moribund mice were sacrificed and spleens were harvested.

We generated a bank of approximately 300,000 Tn insertions in *P. aeruginosa* strain PA14 which was used to determine fitness dynamics in an infection cycle starting with colonization of the ceca followed by dissemination into the spleens after induction of neutropenia, as outlined in Figure 1 and described previously in detail [15]. To identify the genes that influence colonization, the ceca of the mice were harvested 6 days after colonization commenced (Figure 1A), the surviving *P. aeruginosa* strains with the Tn insertions grown, DNA extracted and digested with the MmeI enzyme, DNA fragments in the range of 1,200–1,500 bp obtained by gel fractionation and the recovered oligonucleotides prepared for high-throughput sequencing [6]. To determine all the genes of *P. aeruginosa* necessary for systemic dissemination following neutrophil depletion, strains with Tn insertions that were able to disseminate were recovered from the spleens (Figure 1B). Since colonization is a prerequisite for dissemination, we would only be able to quantify changes in representation among Tn insertions able to colonize the ceca which, following dissemination, would show a reduced or enhanced representation in the Tn library recovered from the spleens.

### Properties of the *P. aeruginosa* PA14 Tn insertion bank grown in LB

**Figure 2:**
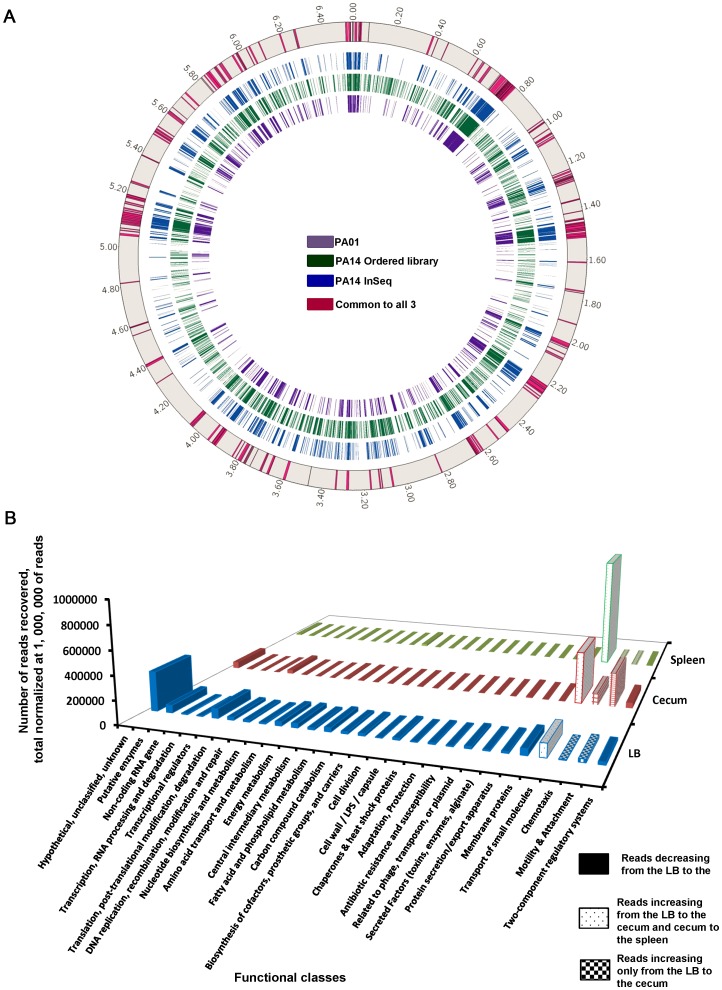
Essential *Pseudomonas aeruginosa* genes and functional classes. A: Comparative identification of essential genes in *P. aeruginosa*. In strain PAO1, 645 genes have been identified as essential (purple circle) whereas in the ordered transposon library of strain PA14, 1,454 genes were considered essential (green circle) [16]. Using a criterion off <10 sequencing reads from the InSeq analysis of strains growing in LB as indicative of an essential gene, 636 genes were classified as essential in strain PA14 (blue circle). Table S1A gives a complete listing of the essential genes from the three different analyses. B: Evolution of the number of reads in each functional class from the LB to the ceca and then to the spleens. The 5,977 genes of PA14 were classified into 27 functional classes [20]. Among a total number of sequencing reads normalized at 1,000,000, the prevalence of reads corresponding to each functional class is presented in the input LB, the output Cecum and the output Spleen.

Quantitative analysis of the overall frequency of Tn insertions into the chromosome was based on use of one million sequencing reads to normalize the data from different DNA preparations. In the LB-grown input bank there was a relatively homogeneous distribution of Tn insertions across the PA14 chromosome. There were 636 genes with <10 sequencing reads (Figure 2A and Table S1), thus defined as unable to grow. Of these 636 genes, 407 were previously identified as essential in strain PA14 [16] and 198 of these 407 are also deemed essential in strain PAO1 [17] (Figure 2A) while 210 essential genes in PA14 are absent from the PAO1 genome (Table S1). Forty-five (0.77%) genes had Tn insertions with more than 1,000 sequencing reads, and they had an average adenine-plus-thymine (A+T) composition of 40.2%, whereas the (A+T) content of the entire genome of PA14 is 33.7% (www.pseudomonas.com). This increase in the density of Tn insertions in A/T rich genes is likely due to the site preference of the mariner transposon, targeting any A/T dinucleotide [16], [18]. The gene with the largest number of Tn insertions was PA14_39470 (7,399 reads), encoding for a hypothetical protein that has an A+T content of 57% (Figure S1 and Table S2). Interestingly, strains with Tn insertions in some well-characterized global regulatory genes such as the *vfr*, *mvfR* and *lasR* that control many aspects of quorum sensing (QS) and virulence in *P. aeruginosa* [19] were also highly represented in the input library, with more than 1,000 reads recovered per gene in the LB-grown Tn insertion pool. The corresponding Tn insertions were absent from the strains recovered from the spleens. Thus, maintaining an intact QS capability is essential for *P. aeruginosa*'s ability to cause systemic infection but has little selective advantage for survival in planktonic cultures.

### Analysis by functional classes of *P. aeruginosa* and identification of positively-selected Tn insertion mutants

The sequencing results allowed us to comprehensively analyze at the full genomic scale the genetic basis for the relative contributions to overall fitness of *P. aeruginosa* under these *in vivo* conditions. By ranking the differential changes in the recovery of the Tn insertions from LB with the cecal and the splenic outputs, we determined the contribution to fitness in three specific areas (Figure S2): 27 functional genomic classes [20] (www.pseudomonas.com) (Figure 2B); individual operons; and finally, genes potentially encoding targets for immunotherapy. These were defined as those making a strong contribution to virulence due to a >10-fold decrease in sequencing reads when comparing the splenic and cecal outputs, but with reads not decreasing more than two times from the LB to the cecum and with at least 10 reads in the cecum (i.e., able to colonize this tissue), combined with an annotation indicative of a likely outer membrane location and thus surface exposure (Table S3).

The analyses of the 27 described functional genomic classes of *P. aeruginosa* revealed differential requirements for colonization and systemic spread. In all but three categories, a decrease in the number of reads from the input pool of LB grown Tn-insertion strains was obtained when compared to the sequencing reads in Tn insertions recovered from ceca (Figure 2B). Interestingly, we found 89 genes where Tn insertions resulted in positive selection during Gastrointestinal (GI) colonization, defined as a ≥2-fold increase in sequencing reads of the bacterial population in the cecal output compared to the LB input, with a minimum of 10 reads (Table S4). Insertions in genes belonging to three functional classes (“Transport of Small Molecules,” “Motility and Attachment” and “Chemotaxis) represented the majority of those undergoing positive selection during cecal colonization. Among these 89 positively selected Tn insertions only a subset were also recovered from the spleens, with most unable to systemically disseminate. Thus, strains with Tn insertions resulting in enhanced GI colonization mostly displayed reduced fitness for systemic dissemination during neutropenia.

**Figure 3:**
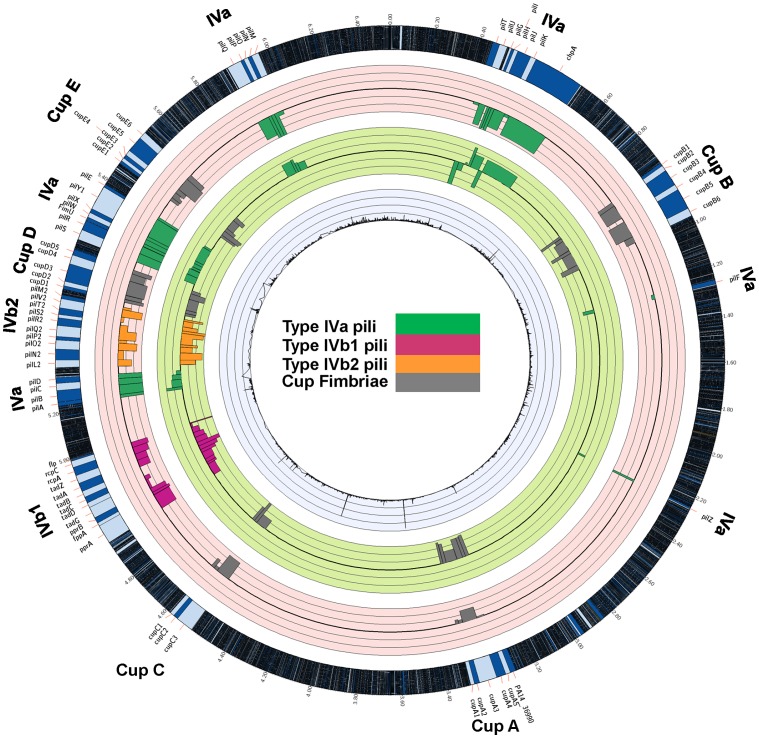
Representation of the changes in the occurrence of Tn-interrupted genes encoding for various classes of pili and Cup fimbriae. Large colored circles represent the PA14 genome for depiction of changes in sequencing reads in different selective conditions. The inner light blue circle represents reads of all of the Tn inserts in the bank grown in LB. The green circle represents the fold change of the reads of the Tn insertions from the LB to the ceca. The pink circle represents the changes from the ceca to the spleens. The outermost circle represents the full PA14 genome with a 30-times magnification of the regions of interest shown as light and dark blue portions of the genome. Black circular lines in the middle of the colored circles represent baseline and thin grey circular lines above and below the central black circular line represent 10-fold-changes (i.e., a log_10_ scale). The limit of the fold change representation is 1,000, except in the inner light blue circle where the changes depicted 0, 2,000, 4,000, 6,000 and 8,000 reads recovered from the LB input (linear scale). Bars represent changes in individual genes and are color coded (key in central part of circle) for genes involved in the indicated functions. Bars pointing toward the circle's center represent Tn interrupted genes resulting in negative fitness whereas bars pointed outward represent genes whose loss increases fitness. The results unveil a positive fitness for GI tract colonization, but not splenic dissemination, of the Type IVa pili Tn insertions but uniform attenuation of Tn mutants in genes encoding the Cup fimbriae and the Type IVb1 and IVb2 pili.

A closer examination of the specific genes with Tn insertions leading to enhanced cecal colonization revealed a consistent trend among those involved in the formation of type IVa pili [21] (Figures 3 and S3). The Tn insertions were scattered among several gene clusters around the *P. aeruginosa* chromosome encoding proteins associated with production of the pilus structural components, including regulation of expression and assembly. The shared phenotype for all of these Tn insertion strains is the lack of synthesis of the pilin subunit and/or the absence of pili on the bacterial surface. Strong positive selection was demonstrated for strains with Tn insertions in genes specifying structural components and biogenesis functions (*pilA, B, C, D, pilE, G, H, I, J, K, M, N, O, P, W, X, Y1, Z* and *fimU*) as well as the *pilS*/*pilR* encoding for the pilin gene specific regulatory two-component system. In contrast, genes that have been previously shown not to have an effect on piliation (*pilK*) and those that do not affect pilus assembly but whose loss appears to result in a hyper-piliated phenotype leading to defective twitching motility (*pilU* and *pilT*) [22], [23], [24] did not display an increased fitness for cecal colonization, although Tn-insertions into *pilH*, which had decreased *in vivo* fitness, had an overlapping phenotype with Tn-insertions into the *pilU* and *pilT* genes [25]. There was also a positive selection for cecal colonization for all of the Tn insertions in *chpA*, a pilin-linked chemosensory system (Figures 3 and S3). Insertions in *pilF*, encoding a lipoprotein needed for proper membrane localization and multimerization of the secretin, PilQ that are both essential for type IVa pilus synthesis displayed poor growth in LB with only 5.8 reads (i.e., essential) compared to an average of 479.8 reads for the Tn insertions in the other pilin genes in strains grown in LB. The basis for this is unclear as a Tn-insertion in *pilF* in *P. aeruginosa* strain PAO1 did not appear to affect growth [26].

The enhanced GI colonization by the strains defective in the production of Type IVa pili suggests that during this stage of infection the pilus structure is a potential target of early recognition by host defense mechanisms, promoting clearance of WT *P. aeruginosa* PA14. Notably, enhanced colonization did not lead to enhanced dissemination as every strain with a Tn insertion in a gene that affected the production or function of the Type IVa pili was recovered in reduced numbers in the spleens (Figures 3 and S4), indicating that these organelles play a critical role in systemic dissemination. However, as a majority of the population maintained production of these possible PAMPs, another explanation such as greater metabolic efficiency from loss of pilus production could account for the observed colonization advantage of pilus-defective mutants.

Several strains with Tn insertions in genes encoding three global regulators of multiple virulence factors, including alginate and pilus production (*rpoN*, *algZ*, *algR*, Table S4) were also over-represented in the ceca compared to LB. Since mutations in genes involved in the biosynthesis of the alginate exopolysaccharide showed reduced fitness for cecal colonization (see below), these findings indicate that the increased fitness of organisms lacking pili compensates for a potential decrease in the ability of alginate-defective bacteria to colonize.

A number of additional surface adhesive organelles, some of which assemble into pilus-like filaments, have been described in *P. aeruginosa* [27], [28], [29]. In contrast to the type IVa class, we observed uniform attenuation in GI colonization of Tn insertions in genes encoding the Cup fimbriae (*cupA, B, C, E*) and the Type IVb1 (*flp*) pilus (Figure 3). Interestingly, insertions into genes encoding for two fimbrial systems within in the PAPI-1 pathogenicity island, CupD and Type IVb2 pili, also resulted in poor cecal colonization and subsequently, poor systemic dissemination (Figure 3). Although common features of all these surface fimbrial structures is their role in attachment to abiotic surfaces or to each other, only the Type IVa pili appear to have an inhibitory role in establishing GI mucosal colonization.

**Figure 4:**
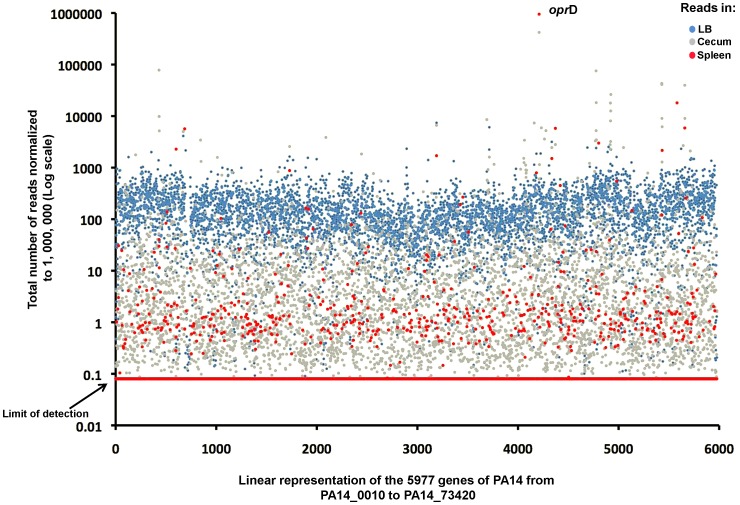
Comparison of sequence reads in each sample. Sequencing reads in each of the 5,977 genes of PA14 recovered from the LB (blue dots), cecum (grey dots) or spleen (red dots).

The strongest positively selected strains were those with Tn insertions into the *oprD* gene encompassing 42% of the Tn insertions recovered from the ceca. They were enriched 816-fold compared to LB (420,621 reads vs 515 reads; Figure 4) and also comprised a striking 94.7% of the strains recovered from the spleens (947,397 reads; Figure 4). OprD is an outer membrane porin previously identified as the main channel for the entry of carbapenem antibiotics into the *P. aeruginosa* periplasm and its loss confers resistance to this important class of antimicrobials [30]. Enhanced fitness of *oprD*-deficient strains both during cecal colonization and systemic dissemination indicates that the expression of this protein in WT *P. aeruginosa* may be detrimental for its survival in mice. OprD could be either involved in mediating the transport of toxic molecules found in mouse tissues or, like the Type IVa pili, this outer membrane protein could present a target for recognition and elimination by the innate host defenses. A detailed analysis of the molecular basis for this enhanced fitness and the role of carbapenem resistance in pathogenesis of *P. aeruginosa* is currently ongoing.

Strains with Tn insertions in 13 genes encoding components of the *P. aeruginosa* flagella system were also positively selected, consistent with the known ability of flagellin to activate host innate immunity via the Toll-like receptor (TLR) 5 and cytosolic innate immune responses that promote bacterial clearance [31], [32], [33] (Table S4).

To avoid any bias due to an overrepresentation in the drinking water of the Tn-insertions in either the *oprD* gene or the genes needed for type IVa pili production, we sequenced the bank of mutants present after 48 h in the drinking water. Tn-insertions in *oprD* represented less than 0.5% of the mutants in the water while the sum of the Tn-insertions in the genes encoding the type IVa pili that represented 34% of the Tn-insertions recovered from the ceca represented only 1.15% of the Tn-insertions in the water. Thus, selection in the drinking water did not account for the enhanced recovery of *oprD* and type IVa pilus Tn-inserts in the cecum.

### Negative selection of Tn insertion strains during cecal colonization

As expected, negative selection was the predominant feature of the changes in the relative proportions of the Tn insertions comparing the LB-grown input pool to the cecal output pool with Tn insertions in 1,333 genes (24.9%) able to grow in the input pool that were completely absent from the cecal output pool (Table S5). Among these 1,333 genes with Tn insertions absent in this pool the largest number, 583 (43.7%), are annotated as hypothetical, unclassified or unknown. These could represent an interesting group of genes for further research towards assigning their products biological functions based on the phenotype of reduced fitness during animal colonization.

### Analysis by operons provides a comprehensive view of *P. aeruginosa* pathogenesis

**Figure 5:**
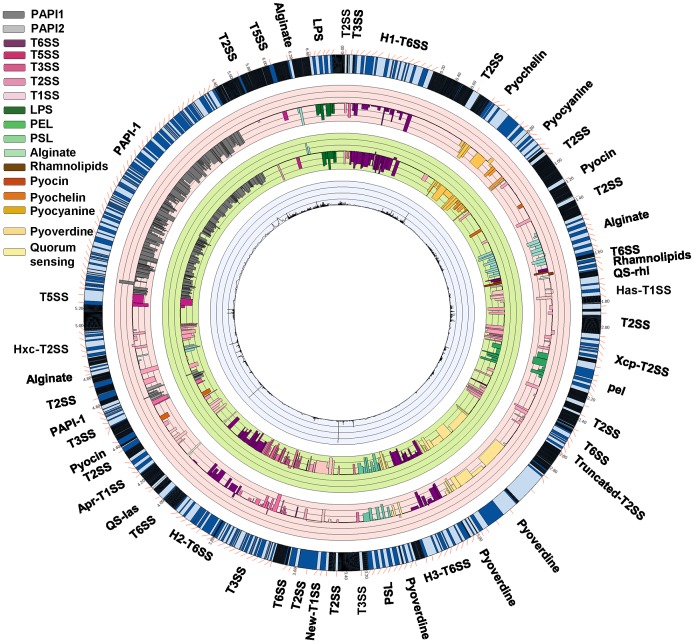
Representation of the changes in the occurrence of Tn-interrupted genes encoding for the identified virulence factor (VF) genes of *P. aeruginosa* PA14. Changes occurring in the ceca in comparison to LB (pink circle) or in the spleens compared to the ceca (pink circle). The VFs are ranked in 4 categories which are color-coded and depicted across a gradient of lighter to darker bars (upper left legend). PAPI-1 and -2 (grey bars); secretion systems (purple bars); exopolysaccharide/LPS (green bars), and others (brown bars) including pyoverdine, pyochelin, pyocyanine, pyocin, rhamnolipids, and quorum sensing (QS). The Tn insertions in the genes encoding for all the known major VF of *P. aeruginosa* are weak colonizers of the GI tract and unable to disseminate to the spleens. In PAPI-1, the genes PA14_59960 had no decrease in the number of reads between the LB and the ceca (408 versus 425, respectively) and PA14_58990 was associated with a positive phenotype for the dissemination as PA14_51580 in PAPI-2. These genes are encoding hypothetical proteins and thus a plausible explanation of these results could be that their products are negative regulators or repressors of functions associated with PAPI-1 and 2. Each circle is as defined in the legend to Figure 3.

**Figure 6:**
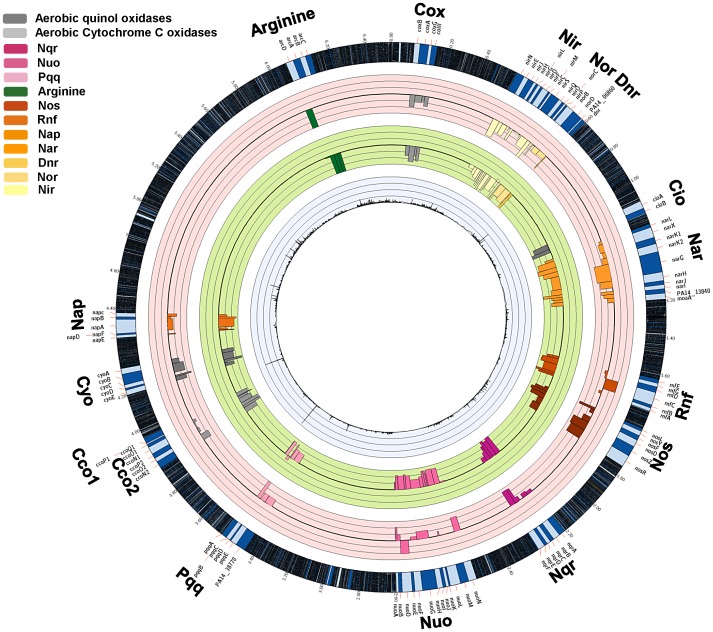
Representation of the changes in the occurrence of Tn interrupted genes encoding for proteins involved in energy production. Tn insertions in the genes involved in the oxygen-dependent respiratory pathways (grey bars), in the denitrification (gradient of yellow/brown bars), in the utilization of energy arginine via the ADI pathway (green bar) and in several other oxygen independent energy generating pathways (gradient of purple bars) showed a decrease *in vivo* fitness. Each circle is as defined in the legend to Figure 3.

The analysis of the distribution of Tn insertions combined with the direction and magnitude of the resultant fitness phenotype can be exploited for the identification of genes that are coordinately regulated and therefore could be related by function. Many of these are found in operons and we therefore analyzed the INSeq results utilizing the previously developed list of defined transcriptional units (TU) of *P. aeruginosa* PA14 [34]. We organized the TUs into three functional categories (virulence factors, aerobic/anaerobic respiration and utilization of nutrients) since this would be most informative about *P. aeruginosa* fitness for survival and proliferation in the mammalian host environment (Figures 5 and 6). For this analysis and the discussion of phenotypes, the effects of different insertions within an operon are considered comparable, regardless of the location of insertions, whether they arose by polar effects on the downstream gene or by direct inactivation of individual genes. This assumption is not unreasonable, considering most of the genes within operons affect related functions. Similarly, for insertions that are thought to be linked to the same pathway, a double mutation should not have an additive effect on fitness. From this analysis we were able to assign putative functions to groups of previously un-annotated genes due to the similarity of their sequences to other annotated genes as well as exhibiting quantitative changes in the output comparable to the annotated genes. The 113 operons of un-annotated genes found to be important for GI colonization were classified as operons specific to the PA14 genome (Table S6), operons generally found among sequenced *P. aeruginosa* strains (Table S7) or operons also present in other *Pseudomonas* species (Table S8).

### Virulence factors: substantiation of known factors and identification and annotation of new ones

Analysis of the negatively-selected genes and operons showed that all of the known *P. aeruginosa* virulence factors compiled in the Virulence Factors of Pathogenic Bacteria database (http://www.mgc.ac.cn/VFs/main.htm) contribute to the overall fitness of *P. aeruginosa* for both colonization and systemic spread (Figure 5A and Figure S5 for a detailed representation of the genes). For example, strains with Tn insertions in all of the known genes associated with extracellular secretion pathways of *P. aeruginosa* (Types 1–3, 5 and 6 secretion systems), including all their previously-characterized secreted effectors, were recovered from the ceca in reduced numbers relative to their representation in LB and were absent from the spleens (Figure 5A). Moreover, we identified a cluster of Tn insertions in previously un-annotated genes that were weak colonizers and unable to disseminate to the spleens (Figure S5). Careful analysis of sequence similarities to proteins in GenBank led to the conclusion that these represent coding sequences for a new Type 1 secretion system (T1SS) that appears to be expressed and functional, and a truncated T2SS similar to the HplR-X export machinery in strain PAO1 (Figure S6A) [35]. These two secretion systems appear to mediate the secretion of virulence factors that contribute to the fitness of *P. aeruginosa* in the host during GI colonization and systemic spread, although it was not determined precisely where and when in the infection cycle they are functional.

### Description of the genes in a new T1SS and truncated T2SS and the inferred functions of encoded proteins

The new T1SS was defined by insertions in the putative T1SS operon (PA14_40230-60) that has limited sequence similarity to the Apr-T1SS and Has-T1SS. The first three genes in this cluster, PA14_40230, PA14_40240, PA14_40250 encode products belonging to the HlyD family of membrane fusion proteins, an ABC transporter and a predicted outer membrane protein, respectively, an arrangement found in all T1SSs (Figure S6A). The factor secreted by this new T1SS is likely encoded by the last gene of the operon PA14_40260 that is predicted to encode a large protein (1,256 amino acids) with 17 repeats of the Big_3_4 sequence (“Bacterial Ig-like domain”, Pfam PF13754) and a single C-terminal domain called a SWM_repeat (“Putative flagellar system-associated repeat”, Pfam PF13753). It has similarity to the LapA protein that functions as an adhesin during colonization of plant seeds by *P. putida* [36] and to an ortholog in *P. fluorescens* that plays a role in irreversible attachment to abiotic surfaces during biofilm formation [37]. It also shares sequence similarity to several proteins of Gram-negative bacteria annotated as hemolysins/hemagglutinins.

A second set of clustered insertions were in an operon that likely represents a truncated T2SS similar to the *hpl*R-X genes described in strain PA01 [35] composed of a cluster of 10 genes (PA14_29480-570) (Figure S6A). They include proteins encoding an ABC transporter, proteins with 69, 49, 57, 45, 51, 65 and 43% sequence similarities to XcpRSTVW, HxcW and XcpX, respectively, an MFS transporter and a hypothetical protein (Figure S6). Missing from this operon are genes encoding the components needed for a complete T2SS including homologs of XcpP/HxcP and XcpQ/HxcQ. Products of two unlinked genes *xphA* and *xqhA* that were also reduced in frequency in the cecal output could conceivably carry out this function. Also missing from this cluster is the gene for the prepilin peptidase required for processing of the pseudopilin components of the T2SS (PA14_21950-PA14_29550), however, it is possible that this function is provided by the product of the highly-conserved *pilD* (*xcpA*) gene.

### Role of surface polysaccharides in *in vivo* fitness

In addition to observing the expected negative selection of Tn insertions into genes needed for LPS O-side chain production, whose loss greatly increases the serum sensitivity of *P. aeruginosa* [38], Tn insertions into genes involved in synthesis of the Pel and alginate polysaccharides also showed reduced fitness for colonization and dissemination (Figure S6B) [39], [40]. Pel (PA14_22480-22560) has been previously shown to be a component of the biofilm matrix promoting bacterial attachment to each other or to inert surfaces. While less firmly associated with biofilm formation by phenotypically non-mucoid (i.e. low alginate) strains like PA14, alginate is a strong candidate for a *bona fide* virulence factor for strain PA14 as Tn insertions into genes within the alginate biosynthetic or regulatory operons led to a colonization defect (Figure S6B). Exceptions were in certain genes involved in global regulation of alginate expression (*rpoN, algZ, algR*), where they also controlled the expression of Type IVa pili. Thus, their inactivation by Tn insertions likely provided a fitness benefit by eliminating the production of pili although at this point we cannot exclude more pleiotropic effects resulting from loss of these global regulatory genes. Nonetheless, these findings indicate that either significant amounts of alginate are induced early during animal colonization, or the low levels of alginate produced by non-mucoid strains provides the bacterial cells with a fitness benefit needed to establish mucosal infection.

An interesting finding was decreased colonization fitness in strains with Tn insertions in the PA14_35550-35690 genes, annotated as *pslE-pslO*. In strain PAO1 and 4 other sequenced *P. aeruginosa* strains, the orthologs of these genes are part of a larger operon containing 4 additional genes, *pslABCD*, needed for synthesis of the Psl polysaccharide. The lack of *pslABCD* in strain PA14 results in no synthesis of the Psl polysaccharide, yet the decreased colonization fitness of Tn insertions in the PA14_35550-35690 genes indicates they have a role in *P. aeruginosa* GI colonization unrelated to Psl polysaccharide synthesis.

### Prophages play a role in the GI tract colonization and systemic dissemination

Two clusters of genes, PA14_07990-08300 and PA14_48880-49010 correspond to integrated prophages, similar to phages 1 and 6 found as either intact or partial genomes in the chromosome of various *P. aeruginosa* strains [41]. The INSeq analysis for fitness in the ceca and spleens demonstrated that Tn insertions in the phage 1 and 6 genes in strain PA14 had reduced fitness (Figure S8B). Except for the Tn insertions in the *gpI* gene (PA14_08040), all the Tn insertions in all of the other genes for these two prophages were weak GI colonizers and none were able to disseminate to the spleens (zero reads recovered for all of them; Figure S8B). Interestingly, the transcription of genes from these two prophages in strain PAO1 grown under anaerobic conditions in the presence of nitrate and nitrite were highly up-regulated [42], consistent with a role for these prophages in *P. aeruginosa in vivo* fitness.

### Requirements for respiration and energy generation, ion pumps and redox reactions, nutrient utilization and potential targets for immunotherapy

The supporting information text (Text S1) contains detailed descriptions of the InSeq results for these aspects of *P. aeruginosa* pathogenesis and host responses.

### Overall changes from the ceca in the frequency of reads of Tn insertion strains recovered from the spleens

Strikingly, even in neutropenic mice, among the 4,522 strains carrying Tn insertions in genes that still allowed some level of colonization of the mouse ceca, only 493 (10.9%) of these Tn insertion strains were able to disseminate to the spleens (Table S9). This unexpected large number of *P. aeruginosa* genes essential for systemic dissemination during neutropenia highlights the complexity and cooperation among the various bacterial factors needed to overcome host barriers and disseminate into organs.

### Validation of INSeq for identifying genes associated with phenotypes important in *P. aeruginosa* virulence

**Figure 7:**
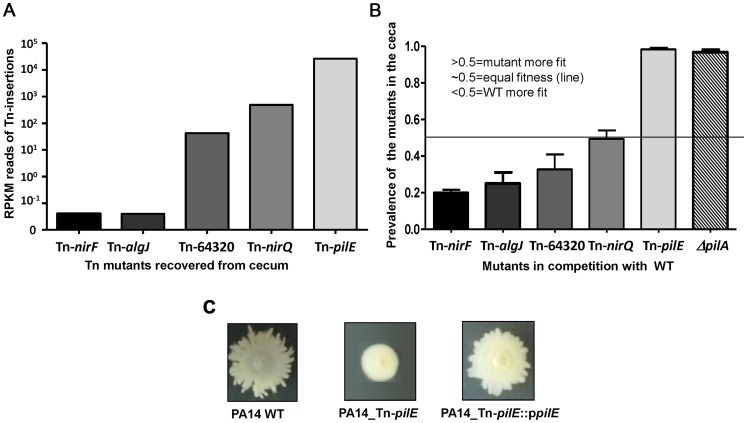
Comparative analysis of fitness for GI colonization of strains identified as having Tn insertions associated with negative fitness, no fitness cost, or positive fitness. A. Relative number of sequencing reads for the Tn-inserts in the indicated genes (name or number) recovered from the cecum following 6 days of colonization with the *P. aeruginosa* PA14 library. B. Proportional recovery of strains selected from the *P. aeruginosa* PA14 ordered Tn library with Tn insertions or deletions in the indicated gene compared to WT PA14 following six days of cecal colonization. C. Defective twitching motility was observed for the Tn-insertion into the *pilE* gene (PA14_Tn-pilE) that was restored when complemented with the *pilE* gene in trans (PA14_Tn-*pilE*::p*pilE*).

To validate the INSeq approach for identification of genes and gene products affecting *P. aeruginosa* fitness, we used competitive challenges by inoculating mice with a 1∶1 mixture of WT *P. aeruginosa* PA14 and different strains selected from the ordered Tn insertion library created in the same strain [16]. The Tn insertions were chosen based on their relative fitness for GI tract colonization as ascertained by the number of sequencing reads recovered (Figure 7A) and included the Tn-*algJ* and Tn-*nirF* strains with reduced fitness, the Tn-PA14-64320 and Tn-*nirQ* strains with slightly reduced and slightly increased fitness for colonization, respectively, and the Tn-*pilE* strain with enhanced fitness for colonization. We confirmed that the Tn insertion in the *pilE* gene resulted in the expected loss of twitching motility (Figure 7C). Overall, the competition experiments validated the INSeq results, with the more fit Tn-*pilE* strain representing more than 99% of the strains recovered from the ceca after inoculation with a 1∶1 mixture of WT strain PA14 (Figure 7B) and the less fit PA14 Tn-*algJ* and Tn-*nirF* insertions representing, on average, only 25% and 20% of the recovered isolates relative to WT, respectively, from the tested mice. For the two strains with intermediate Tn insertions, on average, 50% recovered from the ceca were the PA14 Tn-*nirQ* strain while the PA14 Tn-64320 insertion strain, which had lower reads than the Tn-*nirQ* insertion strains, survived on average, at a level of about 38% compared to WT PA14 (Figure 7B). Thus the ratio of strains with Tn insertions in these five genes to WT *P. aeruginosa* determined by INSeq were experimentally verified by direct *in vivo* competition experiments. The increased fitness of a mutant in the formation of type IVa pili was also confirmed using a strain with a clean deletion of the *pilA* gene (Figure 7B).

### Determination of potential bottleneck effect for splenic dissemination

**Figure 8:**
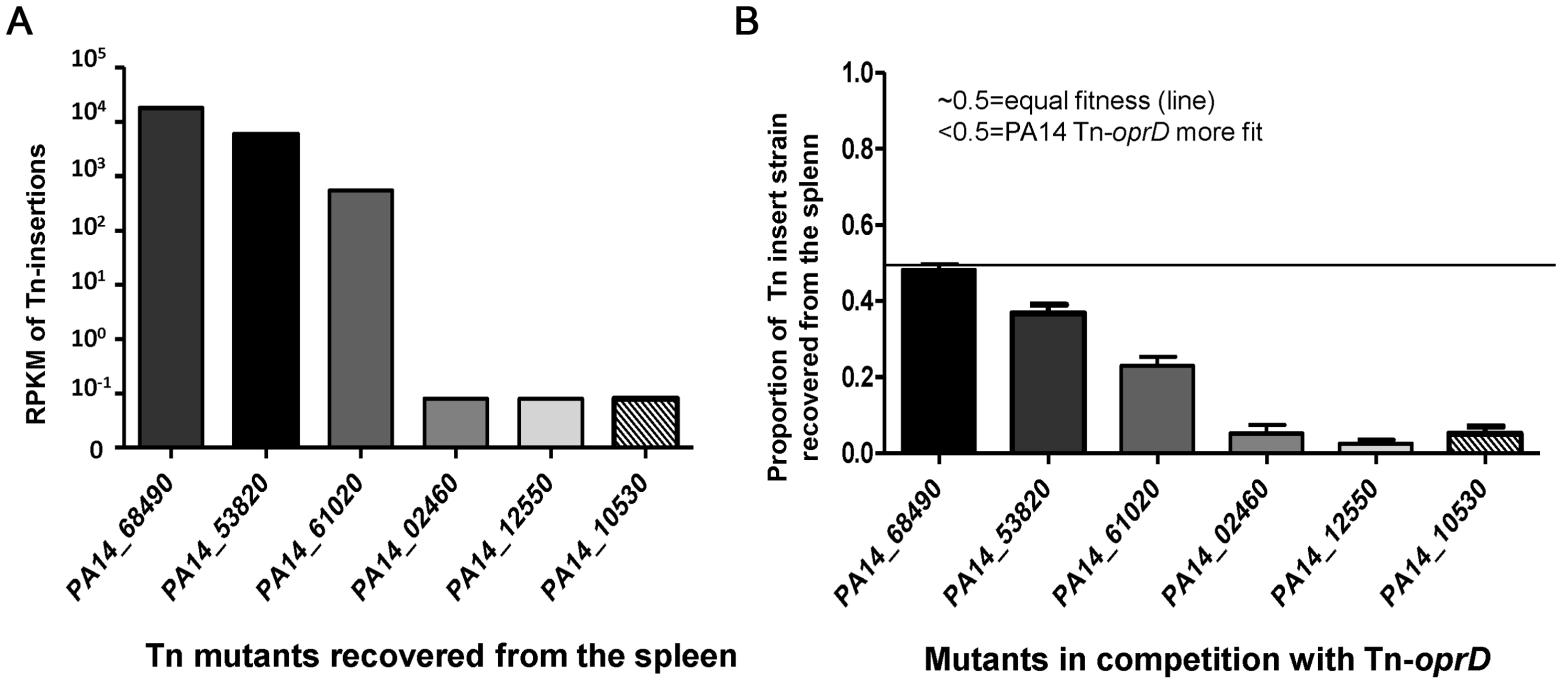
Confirmation of the lack of a bottleneck accounting for enhanced fitness of strains with *oprD* Tn-insertions in the spleen. A: RPKM reads in six strains with Tn insertions in unannotated genes recovered from the spleen in the original saturated Tn-insertion library reflecting three strains recovered from the spleens and three with <0.1 RPKM reads. B: Proportion of six *P. aeruginosa* PA14 Tn-insertion strains recovered from the spleen of GI colonized and neutropenic mice in comparison to Tn-insertion strain PA14 Tn-*oprD*. Bars represent the means, error bars the S.D.

It is important to rule out a potential bottleneck effect that could account for the high level of recovery of Tn_*oprD* strains from the spleen. We therefore hypothesized that strains with Tn-insertions capable of both colonizing the GI tract and disseminating to the spleen in the original saturated Tn library would be recovered from the spleens of neutropenic mice (following 6 days of GI colonization) when in direct competition with a Tn_*oprD* strain at relative levels comparable to those detected in the original bank. In contrast, Tn-insertions in genes that still allowed for measurable GI colonization but eliminated the ability to spread systemically would similarly be restricted from spreading when in competition with a Tn_*oprD* strain. In these experiments the Tn_*oprD* strain selected from the ordered Tn-insertion library in strain PA14 [16] was mixed at a 1∶1 ratio with one of six strains from this same library with Tn-insertions in un-annotated genes, representing strains with Tn insertions that were recovered from the GI tract with 30–2,800 RPKM reads, indicative of a range of abilities to colonize the cecum. Three of the Tn-insertion strains (PA14 Tn-68490, PA14 Tn-53820 and PA14 Tn-61020) were also able to disseminate to the spleen at three different levels (∼7×10^2^ RPKM, 7×10^3^ RPKM and 2×10^4^ RPKM, Figure 8A) when present in the original saturated Tn-insert library, whereas the additional three Tn-insertion strains, although able to colonize the GI tract, were unable to disseminate to the spleen (strains PA14 Tn-02460, PA14 Tn-12550, and PA14 Tn-10530) (Figure 8A). The three strains with Tn-insertions in genes that had been able to disseminate when part of the original Tn-insert library were recovered from the spleens when in competition with the Tn-*oprD* strain at levels comparable to their *in vivo* fitness in the entire saturated Tn-insertion library (Figure 8B). This result indicated that the mutants with the Tn insert in the *oprD* gene did not disseminate to the spleen due to an advantage reflecting a bottleneck effect. The three strains unable to disseminate to the spleen from the initial saturated Tn-insertion library were recovered at levels of <2% when in competition with the Tn_*oprD* strain. These outcomes confirmed that strains in the original Tn library with Tn inserts that essentially completely compromised their ability to disseminate systemically in the setting of neutropenia were similarly unable to do so when placed in competition with a Tn_*oprD* strain, indicative of a true selective advantage for the Tn_*oprD* strain.

## Discussion

We used the INSeq technique and high-throughput sequencing that determine the location and abundance of Tn insertions in a chromosome to assess the comparative fitness of strains with these insertions in *P. aeruginosa* PA14 grown in laboratory media, recovered from the ceca of colonized mice and then from the spleens of neutropenic mice. Fitness determinations were based on the normalized quantification of the changes in the relative amounts of the Tn-interrupted genes determined in each population. The findings produced a data set that appears to be both comprehensive and quantitative and in several cases yielded unique information about *P. aeruginosa* fitness in an *in vivo* setting that is likely closely related to human infection [11]. Notably, we identified and validated positively-selected phenotypes in an *in vivo* setting, identified and annotated some putative new virulence factors, determined that regulatory circuits that operate in a complex environment of an infected animal are differentially impacted by their contributions to fitness of the genes and gene-products they regulate, and have uncovered a potential means to identify new *P. aeruginosa* vaccines. Moreover, the relative fitness of different Tn insertion strains provided important information about host environments, ranging from the activities of the host defense mechanisms that recognized an infectious organism to respiration conditions to nutrient availability and how these conditions might impact the genes needed by the pathogen for survival. As with many such tools, the amount of data generated has resulted in identifying a broad array of factors to be studied in more detail, encompassing the entire range of *P. aeruginosa* biology, virulence, metabolism, and genetic regulation.

Previously INSeq was used by Goodman et al. [6] to identify genes in the GI symbiont *Bacteroides thetaiotaomicron* that are needed to establish colonization in both WT and immunodeficient gnotobiotic mice. Experimental variations included the presence or absence of different commensals from the human GI tract. They recorded significant changes in the population of *B. thetaiotaomicron* recovered from the GI tract, identifying genes needed for survival and nutrient acquisition when in competition with different organisms. Overall they found fewer (280) essential genes needed for GI colonization by *B. thetaiotamicron* compared with what we report here for *P. aeruginosa* (1,333), but this difference would be expected when comparing genes essential for colonization in germ-free versus antibiotic-treated conventional mice and between a normal GI commensal and an opportunist. Van Opijnen et al. [7] used the INSeq/Tn-Seq method to identify gene interactions during *in vitro* growth of *Streptococcus pneumoniae* and additionally utilized analysis of genes involved in nasal colonization and dissemination to the lungs along with analysis of sequencing results from 17 *in vitro* conditions to construct a detailed genetic interaction network for this organism. Other investigators used variations of the INSeq/Tn-Seq technology to identify genes whose products interfere with the clearance of *Hemophilus influenzae* from the lungs of infected mice [8], genes responsible for tobramycin resistance in *P. aeruginosa* [43] and genes needed for growth of *Salmonella enterica* serovar Typhimurium in three different *in vitro* conditions mimicking aspects of the GI tract [44]. Along with our results here, only the studies of Goodman et al. [6] and van Opijnen and Camilli [7] utilized the *Mme*1 restriction enzyme binding site to quantify levels of Tn-interrupted genes surviving in different conditions, a experimental approach that all of the studies found led to vigorous and quantitative insights into pathogenesis and fitness of microbes in the settings studied.

There were several important features of the approach that we judged to be critical for obtaining a robust data set identifying genetic requirements that optimized *P. aeruginosa* survival under different environments and were subsequently experimentally validated using *in vivo* competition experiments. A period of six days of GI colonization followed by dissemination during neutropenia [15] not only recapitulates many features of *P. aeruginosa* infection in hospitalized patients [12], [13] but also likely reduced or eliminated strains with Tn insertions that could survive a shorter selective period, thus providing a strong pressure for survival of truly fit Tn-insertion strains. Neutropenia is a well-known risk factor for mouse and human *P. aeruginosa* sepsis [45], [46] and the level of neutropenia we achieved minimizes host resistance to *P. aeruginosa* infection [15]. Nonetheless, we found that only 493 strains with Tn-interrupted genes out of 4,522 strains carrying Tn insertions that were able to colonize the GI tract were also able to disseminate to the spleen. The need for >90% of all of the genes in *P. aeruginosa* PA14 to be intact in order to have an organism maximally fit for systemic dissemination in an immunocompromised setting seems quite remarkable and might form a novel basis for reassessing what constitutes a microbial virulence factor.

Additionally, the results we obtained were consistent with many previous studies on *P. aeruginosa* virulence factors in that loss of the ability to produce these well-characterized factors, encompassing secretion systems, LPS, pyocyanin, quorum-sensing controlled entities and many others, was readily ascertainable as strains with Tn insertions in the genes needed for production of these factors were deficient in GI colonization and systemic spread. While classifying a gene or operon as encoding a virulence factor based on decreased recovery from an *in vivo* setting may be an overly simplistic application of this term, it was nonetheless a strong validation of the techniques and tools used in our study to find that essentially all of the genes encoding currently classified virulence factors were recovered at much lower rates from *in vivo* settings when compared to their occurrence after growth in LB. Validation of the accuracy of INSeq at identifying contributions to relative fitness was provided by confirming the correlation between the sequencing reads recovered from the ceca and the competitive fitness for colonization of the Tn insertion strains. Taken as a whole, the INSeq methodology used to assess the fitness of essentially all obtainable gene interruptions in *P. aeruginosa* PA14 yielded a plethora of data and possible opportunities for forming and testing hypotheses about microbial biology. Prioritization of the next set of experiments based on these data is challenging, but high levels of interest might be predicted for identifying potential targets for antimicrobial therapy and/or active or passive vaccination, further analysis of positively-selected phenotypes or perhaps analysis of the role of genes and associated gene products with a strong phenotype for negative selection but without an obvious functionality explaining the phenotype. Another finding undergoing further evaluation is related to the observation that many of the endogenous chromosomal antibiotic resistance genes were needed for full fitness for GI colonization and dissemination in the absence of the selective pressures from antibiotics. Finally, as our group has been interested in use of live attenuated vaccines for inducing immunity to *P. aeruginosa* [47], [48], [49] identification of factors like the Type IVa pili whose loss promotes mucosal colonization but also compromises the organism's ability to disseminate, even during neutropenia, suggests that Type IVa pilus-deficient strains might be part of an attenuated oral vaccine that maximizes GI colonization, and hence immunization, potential, while not being proficient at causing serious systemic disease.

## Materials and Methods

### Ethics statement

The Harvard Medical School animal management program is accredited by the Association for the Assessment and Accreditation of Laboratory Animal Care, International (AAALAC), and meets National Institutes of Health standards as set forth in the 8th edition of the Guide for the Care and Use of Laboratory Animals (National Research Council. “2 Animal Care and Use Program.” Guide for the Care and Use of Laboratory Animals: Eighth Edition. Washington, DC: The National Academies Press, 2011). The institution also accepts as mandatory the PHS Policy on Humane Care and Use of Laboratory Animals by Awardee Institutions and NIH Principles for the Utilization and Care of Vertebrate Animals Used in Testing, Research, and Training. There is on file with the Office of Laboratory Animal Welfare (OLAW) an approved Assurance of Compliance (A3431-01). All animal studies conducted in this research were approved by the Institutional Animal Care and Use Committee under protocol number 02791.

### Generation of the PA14 transposon mutant library

The transposase and its upstream regulatory region from pBTK30 [50] was PCR amplified and cloned as a *BamH*I restriction fragment into pSAM_Bt [6]. The erythromycin cassette was then removed from the resultant plasmid, pSAM_DYH, using *Mfe*I and *Xba*I restriction enzymes, and replaced using a PCR fragment containing the gentamicin resistance cassette from pUC19Gm. The resulting plasmid, pSAM_DGm was verified by PCR and sequencing and subsequently transformed into *E. coli* Sm10λpir [51] to generate a donor strain for conjugation (Figure S10).

The donor strain, *E. coli* Sm10λpir pSAM_DGm was grown overnight in LB with 10 µg/ml gentamicin, and the recipient *P. aeruginosa* PA14 [52] was grown in LB without antibiotics. The cells were centrifuged, washed in LB, centrifuged again and re-suspended to O.D._600 nm_ of 2.0. Equal volumes of donor and recipient were mixed and 100 µL of the suspension was pipetted onto pre-warmed LB agar plates. For the preparation of the library, 100 independent conjugation mixtures were used. The plates were left at room temperature for 10 min to allow the liquid to absorb into the agar and the mating continued for 3 hours at 37°C. Each conjugation reaction was scraped off, suspended in 10 ml LB and 150 µl aliquots were plated on 100 LB plates containing irgasan (25 µg/ml) and gentamicin (75 µg/mL). After approximately 12 hours at 37°C, the plates were flooded with LB and colonies scraped off using a cotton swab and pooled. The yield was approximately 300,000 individual colonies from 100 plates. The pooled library was further incubated for two additional hours in LB broth with gentamicin. Following centrifugation of the culture, the library was re-suspended in LB containing 20% glycerol and aliquots were frozen at −80°C.

### Murine model of GI tract colonization and systemic dissemination

The murine model of GI tract colonization and systemic dissemination by *P. aeruginosa* was performed as previously described [15]. In this model, around 1000 times more cfu of *P. aeruginosa* PA14 were recovered from the ceca (10^8^/cecum) that the number of mutants in the bank (3×10^5^) while an average of ∼10^7^ cfu/spleen were recovered after induction of neutropenia.

### DNA preparation for high-throughput sequencing (Figure S11)

*Mme*I is a type-IIS restriction-modification enzyme that cuts the double-stranded DNA 20 bp away from the recognition site, thus 16 bp outside of the Tn insertion. It produces sticky-end DNA fragments with a two-nucleotide 3′overhang. The transposons inserted into the PA14 genome contained *Mme*I restriction sites on both ends of the Tn, allowing the *Mme*I enzyme fragments of the genes on both sides of the Tn insertion for sequencing. Moreover, P7 Illumina sequences were also present just adjacent to the *Mme*I recognition sites on both ends of the Tn. These features allowed the high throughput sequencing to proceed to identify the gene interrupted and place of insertion for each individual Tn insertion.

### Digestion with MmeI

After RNase treatment, all DNA samples were digested with *Mme*I (NEB) according to manufacturer's instructions for one hour, then heat-inactivated for 20 minutes at 80°C, purified (QIAquick, PCR purification kit Qiagen) and concentrated using a Speed-vac to a final volume of 30 µL.

### Gel extraction of the transposon carrying the adjacent genomic DNA

Digested DNA samples were run on a 1% agarose gel and the 1.2–1.5 kb band was cut out, and gel extracted (Qiaquik Gel Extraction Kit, Qiagen) per manufacturer's instructions. This allowed recovery of all transposons containing adjacent genomic DNA (i.e. 1, 349 bp of transposon with additional 2×16 bp of genomic DNA).

### Creation of double stranded adaptors

A pair of single-stranded complementary oligonucleotides (LIB_AdaptT: 5′-TTCCCTACACGACGCTCTTCCGATCTNN-3′ and LIB_AdaptB: 5′-AGATCGGAAGAGCGTCGTGTAGGG-3′) was mixed and heated to 100°C, then slowly cooled down in a water bath to obtain double-stranded adaptors with two-nucleotide 5′overhangs.

### Ligation of the adaptors to the transposons

Sticky adaptors (0.44 µM) were ligated to 1 µg of gel-extracted DNA samples with T4 DNA ligase (NEB) in 50 µL during overnight reactions at 16°C, heat-inactivated at 65°C for 10 minutes, then purified (Qiaquick PCR purification Kit, Qiagen) and quantified by Nanodrop analysis.

### PCR amplification

Amplification by PCR of both ends of all extracted transposons was performed using a forward primer [LIB_PCR_5: 5′-CAAGCAGAAGACGGCATACGAAGACCGGGGACTTATCATCCAACCTGT-3′, which anneals to the P7 Illumina sequence of the transposon] and a reverse primer [LIB_PCR_3: 5′-AATGATACGGCGACCACCGAACACTCTTTCCCTACACGACGCTCTTCCGATCT-3′, which anneals to the P5 adaptor], and the HiFi DNA Polymerase (KAPA Biosystems). This PCR step enriches for fragments containing the P7 sequence, the end of the transposon, the genomic DNA junction sequence, and the P5 adaptor and generated the Illumina libraries for sequencing.

After confirmation of the presence of a 125 bp product on an 1% agarose gel, samples were purified, quantified and sent for sequencing (Harvard Biopolymers Facility).

### Bioinformatic analysis

We obtained 83, 46, and 21 million sequencing reads for the LB input, the ceca (colonization output), and the spleens (dissemination output) from the Illumina sequencing respectively. All bioinformatics were performed in CLC Genomics Workbench 5.5.1 (CLC bio).

### Trimming

All sequences were trimmed to eliminate those reads with quality scores less than 0.05 and/or sequences with ambiguous nucleotides. We then trimmed the sequence “(AGACCGGGGACTTATCATCCAACCTGTTA”) that corresponded to the end of the transposon from our reads. Finally, sequences shorter than 14 nucleotides were removed. At the end, 57, 39 and 12 million reads for the input, the cecum, and the spleen samples, respectively, remained.

### Alignment on the PA14 genome

We downloaded the PA14 genome from www.pseudomonas.com, which currently contains 5,977 annotated genes. All trimmed sequences from the 3 samples were mapped on the annotated genome, from −120 nucleotides (to include promoter regions) to the end of each ORF, including the final 20% of the gene. Notably, these insertions may not abrogate gene function. Ambiguous reads were excluded, and no mismatches were allowed for the mapping. With these stringent conditions, we were able to map 20.2, 18.3 and 6.5 millions of reads for the input, the cecum and spleen outputs, respectively. There were between 10 and 40 inserts for every kb of DNA. As the median number of Tn inserts in an identified ORF was obviously dependent of the size of the gene, the reads per kilobase per million reads (RPKM) was used for the overall comparative analyses.

### Statistical analysis of bioinformatics data

The fold changes under all conditions were determined and the results for all genes were analyzed for statistically-significant differences in their occurrence using the On Proportions function of CLC with corrected p-values calculated by the Bonferroni False-Discovery Rate method. Only differences in Tn insertion frequencies generating p-values less than 0.05 were considered significant.

### Analysis by operons

Using the results from an RNA sequencing study [34], we identified operons associated with a decreases fitness for colonization of their Tn insertions, defined as operons of at least 4 genes with a fold change of reads greater than 10 between the LB and the cecum, or operons with fewer genes but with at least one gene associated with a fold change in sequencing reads greater than 1000 between the LB and the ceca. Only genes with more than 10 reads in the LB were considered. Once such an operon was detected, we looked individually for the evolution of each gene in this operon from the LB environment to the ceca and from the ceca to the spleens.

#### Construction of the *P. aeruginosa* PA14 *pilA* depleted and complemented strains

The *pilA* deletion mutant (PA14ΔpilA) was derived from the wild-type PA14 using the replacement vector pEX18Tet as described previously [53]. Complementation was achieved by cloning the *pilA* gene into mini-CTX1 [54].

### *In vivo* competition experiments

To assess the *in vivo* fitness of PA14 strains with Tn interruptions in specific genes, we recovered PA14 strains with identified Tn insertions from the PA14 Non-Redundant Transposon Insertion Mutant Set [16]. The GI tract was decontaminated using penicillin-streptomycin drinking water. We used 4 mice for each competition. After 5 days, the drinking water was replaced with penicillin water containing a 1∶1 ratio of the wild-type PA14 and a single PA14 Tn mutant (5×10^7^ CFU/ml for each strain). After 6 days, mice were given sterile drinking water with penicillin alone and 24 h later the mice were euthanized and the ceca harvested. Serial dilutions were plated on LB agar without antibiotics. After overnight growth, 48 colonies from each mouse were individually streaked on LB agar with and without gentamicin to evaluate the final ratio of PA14 mutants (gentamicin resistant) and wild-type PA14 (gentamicin susceptible). This ratio was further confirmed by sub-culturing 100 separate colonies that grew on the LB agar plates onto LB-gentamicin plates and analysis by PCR of each one of these colonies.

### Circos plot figures

Circos figures in the main text (Figures 2A, 3, 5A–B and 6) and in the supporting information figures (Figures S3, S4, S5) were drawn following the instruction provided in http://www.circos.ca/. All the data used to generate the Circos figures are presented in Table S10.

## Supporting Information

Figure S1 Properties of the *P. aeruginosa* PA14 Tn-mutant bank grown overnight in LB.
(PPTX)

Figure S2 Schematic for the genomic scale analysis of *P. aeruginosa* PA14 fitness.
(PPTX)

Figure S3 Detailed analysis of the fitness for mucosal colonization of the genes involved in Type IVa pilus production. Selection for fitness for cecal colonization (green circle) of Tn inserts in genes involved in the production of Type IVa pili. Bars pointing outward from the central circular black line have a positive fitness for colonization, bars pointing toward the center have a negative fitness for cecal colonization and include Tn insertions that do not lead to a defect in piliation (*pilK*, *pilU* and *pilT*) or have a growth defect in LB (*pilF*). The light and dark blue chromosomal regions in the outermost circle are magnified 60× in relation to the rest of the bacterial genes to highlight the regions of interest.
(PPTX)

Figure S4 Fitness for systemic dissemination of the Tn insertions in genes for Type IVa pili. All the Tn insertions shown have a negative fitness for systemic dissemination. The light and dark blue chromosomal regions in the outermost circle are magnified 60× in relation to the rest of the bacterial genes to highlight the regions of interest.
(PPTX)

Figure S5 Reduced *in vivo* fitness of the Tn insertions in the genes encoding for the VFs of *P. aeruginosa*. Details of the genes. Each circle is as defined in Figure 3. The light and dark blue chromosomal regions in the outermost circle are magnified 60× in relation to the rest of the bacterial genes to highlight the regions of interest.
(PPTX)

Figure S6 A. Detailed analysis of the genes and operons important for GI tract colonization identifies a new T1SS (PA14_40230-250) with its probable secreted factor encoded by PA14_40260 and a truncated T2SS. Selection for fitness for cecal colonization (green circle) of Tn inserts in genes within an operon with features of a T1SS and a truncated T2SS similar to HplR-X in *P. aeruginosa* strain PA01 [35]. All of the genes had a negative fitness for cecal colonization. The light and dark blue chromosomal regions in the outermost circle are magnified 60× in relation to the rest of the bacterial genes to highlight the regions of interest. B. Detailed analysis of the exopolysaccharide/LPS genes and operons important for GI tract colonization. Selection for fitness for cecal colonization (green circle) of Tn insertions in the genes encoding for the LPS O-side chain and three different extracellular polysaccharides, Pel, Psl and alginate. All of the genes had a negative fitness for cecal colonization except the Tn mutants in the *algR* and *algZ*, regulatory genes that are also involved in the positive regulation of expression of the Type IVa pili. Increased fitness of these Tn inserts whose loss decreases Type IVa pilus production likely reflects the overall increased fitness for colonization of Type IVa pilus-negative clones. The light and dark blue chromosomal regions in the outermost circle are magnified 60× in relation to the rest of the bacterial genes to highlight the regions of interest.
(PPTX)

Figure S7 Fitness changes in Tn insertions in genes involved in nutrient utilization. Each circle is as defined in Figure 3. The light and dark blue chromosomal regions in the outermost circle are magnified 60× in relation to the rest of the bacterial genes to highlight the regions of interest.
(PPTX)

Figure S8 A. Discordance for selection of Tn insertions in the genes *hutD*, *mdcB* and *glgX* versus the remainder of the genes within their operons. Each circle is as defined in Figure 3. The light and dark blue chromosomal regions in the outermost circle are magnified 60× in relation to the rest of the bacterial genes to highlight the regions of interest. B. *In-vivo* loss of fitness for the Tn insertions in two prophages of *P. aeruginosa* strain PA14 encoded by operons of un-annotated genes. Each circle is as defined in Figure 3. The light and dark blue chromosomal regions in the outermost circle are magnified 60× in relation to the rest of the bacterial genes to highlight the regions of interest.
(PPTX)

Figure S9 Potential targets for immunotherapy. These Tn insertions have no defect in GI tract colonization (A) but are not able to disseminate to the spleen during jneutropenia (B). The light and dark blue chromosomal regions in the outermost circle are magnified 60× in relation to the rest of the bacterial genes to highlight the regions of interest.
(PPTX)

Figure S10 Map of pSAM-DGm used to create the Tn-insertion library. The two recognition sites for the MmeI enzyme are represented by the two small orange rectangles at the beginning and end of the mariner-based transposon. Gm = Gentamicin.
(PPTX)

Figure S11 Principle for the preparation of the saturated Tn-insertion library for high-throughput sequencing.
(PPTX)

Table S1 Genes identified as essential in *P. aeruginosa* strain PA14.
(XLS)

Table S2 Tn-insertions into *P. aeruginosa* PA14 genes with more than 1,000 sequencing reads after growth in LB.
(DOC)

Table S3 Genes (N = 129) identified with minimal changes (<2-fold) in the number of sequencing reads when comparing sequencing reads obtained from bacteria grown in LB to those from bacteria recovered from the cecum (minimum 10 sequencing reads in cecum).
(DOC)

Table S4 Tn-insertionss in genes in *P. aeruginosa* strain PA14 with a positive phenotype for colonization.
(DOC)

Table S5 Tn-insertions into genes in *P. aeruginosa* strain PA14 unable to colonize the murine GI tract.
(DOC)

Table S6 Nine operons with unannotated genes found in strain PA14 and no other sequenced *P. aeruginosa* strains that all have a have a reduce fitness for colonization.
(DOC)

Table S7 Tn-insertions in genes within operons of unannotated genes found in strain PA14 and other sequenced *P. aeruginosa* strains that have a reduce fitness for colonization.
(DOC)

Table S8 Tn-insertions in genes within operons of unannotated genes found in all sequenced *P. aeruginosa* strains and in other bacteria that have a reduce fitness for colonization.
(DOC)

Table S9 Tn-insertions into genes of *P. aeruginosa* strain PA14 capable of systemic dissemination in neutropenic mice.
(DOC)

Table S10 Raw data used to draw the Circos figures.
(XLS)

Text S1 Requirements for respiration and energy generation, ion pumps and redox reactions and potential targets for immunotherapy by *P. aeruginosa* during infection.
(DOC)
