# Supplementary material for: A Comprehensive Analysis of In Vitro and In Vivo Genetic Fitness of Pseudomonas aeruginosa Using High-Throughput Sequencing of Transposon Libraries
Source: PLoS Pathog. 2013 Sep 5;9(9):e1003582. doi: 10.1371/journal.ppat.1003582 (PMC3764216; doi:10.1371/journal.ppat.1003582)
Supplement: Figure S1 — Properties of the P. aeruginosa PA14 Tn-mutant bank grown overnight in LB. (PPTX) [file ppat.1003582.s001.pptx]

## Slide 1
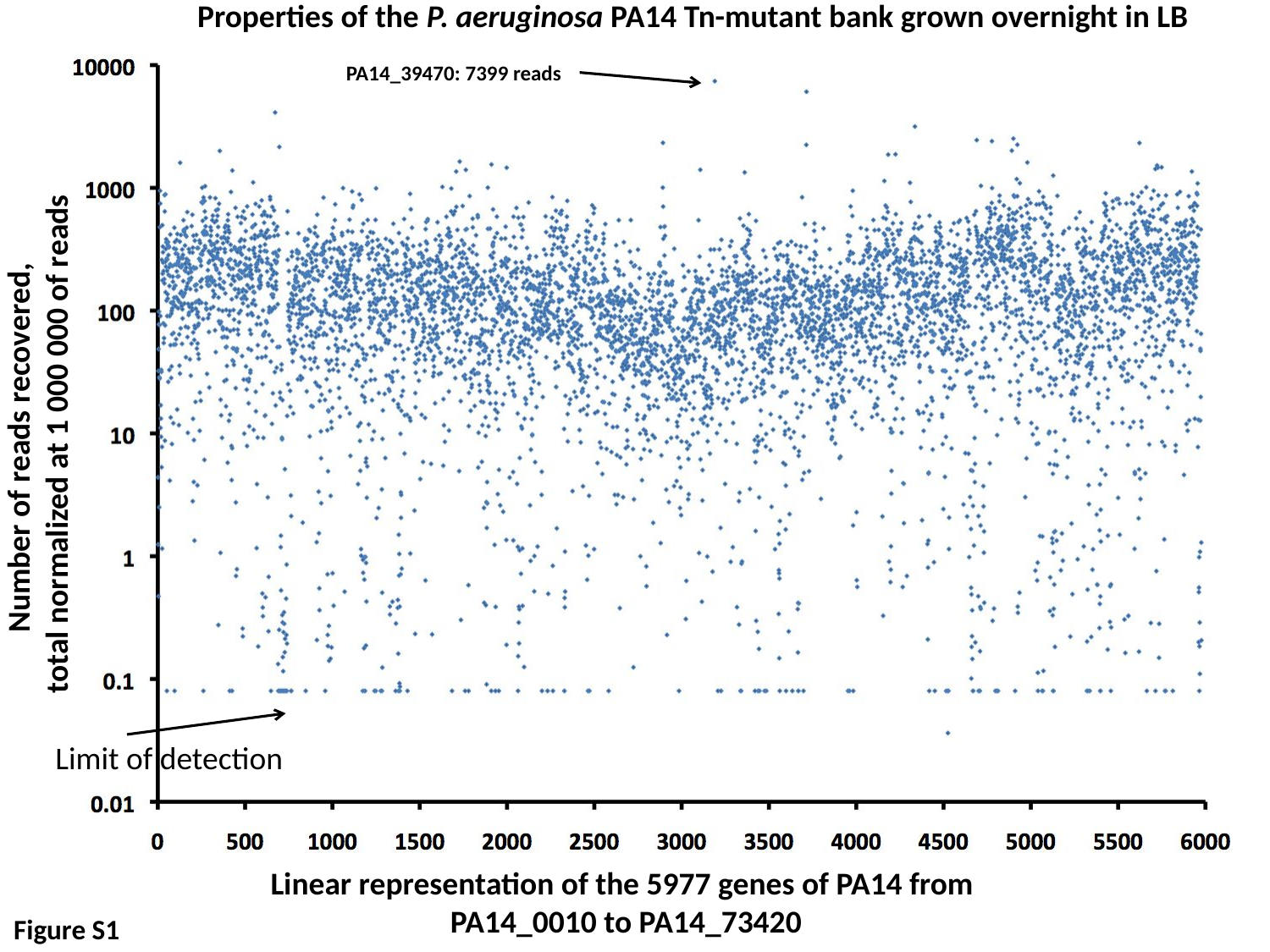

Properties of the P. aeruginosa PA14 Tn-mutant bank grown overnight in LB
PA14_39470: 7399 reads
Number of reads recovered,
total normalized at 1 000 000 of reads
Limit of detection
Linear representation of the 5977 genes of PA14 from
 PA14_0010 to PA14_73420
Figure S1
