# Supplementary material for: A Comprehensive Analysis of In Vitro and In Vivo Genetic Fitness of Pseudomonas aeruginosa Using High-Throughput Sequencing of Transposon Libraries
Source: PLoS Pathog. 2013 Sep 5;9(9):e1003582. doi: 10.1371/journal.ppat.1003582 (PMC3764216; doi:10.1371/journal.ppat.1003582)
Supplement: Figure S2 — Schematic for the genomic scale analysis of P. aeruginosa PA14 fitness. (PPTX) [file ppat.1003582.s002.pptx]

## Slide 1
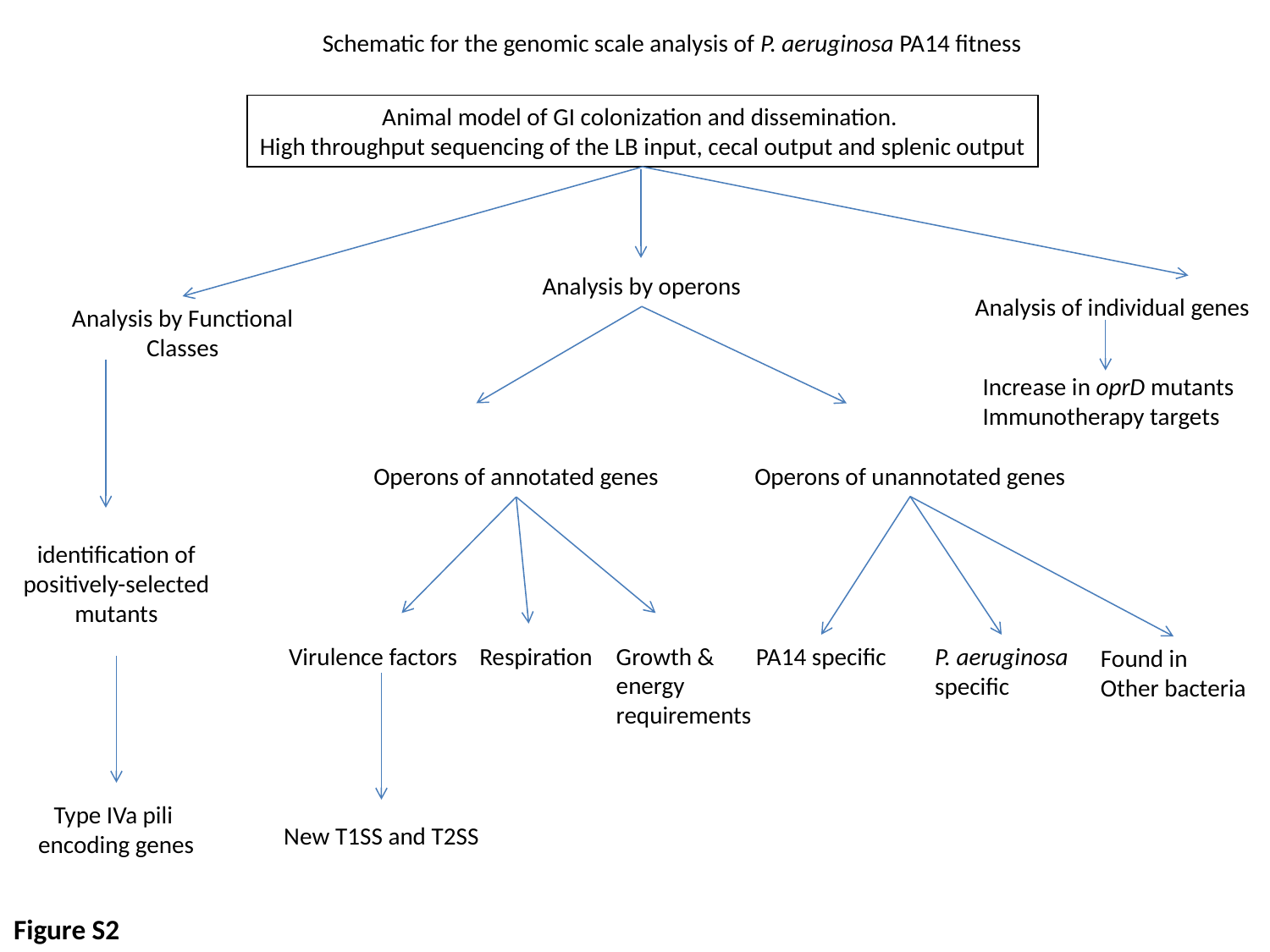

Schematic for the genomic scale analysis of P. aeruginosa PA14 fitness
Animal model of GI colonization and dissemination.
High throughput sequencing of the LB input, cecal output and splenic output
Analysis by operons
Analysis of individual genes
Analysis by Functional Classes
Increase in oprD mutants
Immunotherapy targets
Operons of unannotated genes
Operons of annotated genes
identification of positively-selected mutants
Virulence factors
Respiration
Growth & energy requirements
PA14 specific
P. aeruginosa
specific
Found in
Other bacteria
Type IVa pili
encoding genes
New T1SS and T2SS
Figure S2
