# Supplementary figures and images for: A Comprehensive Analysis of In Vitro and In Vivo Genetic Fitness of Pseudomonas aeruginosa Using High-Throughput Sequencing of Transposon Libraries
Source: PLoS Pathog. 2013 Sep 5;9(9):e1003582. doi: 10.1371/journal.ppat.1003582 (PMC3764216; doi:10.1371/journal.ppat.1003582)

## Slide 1
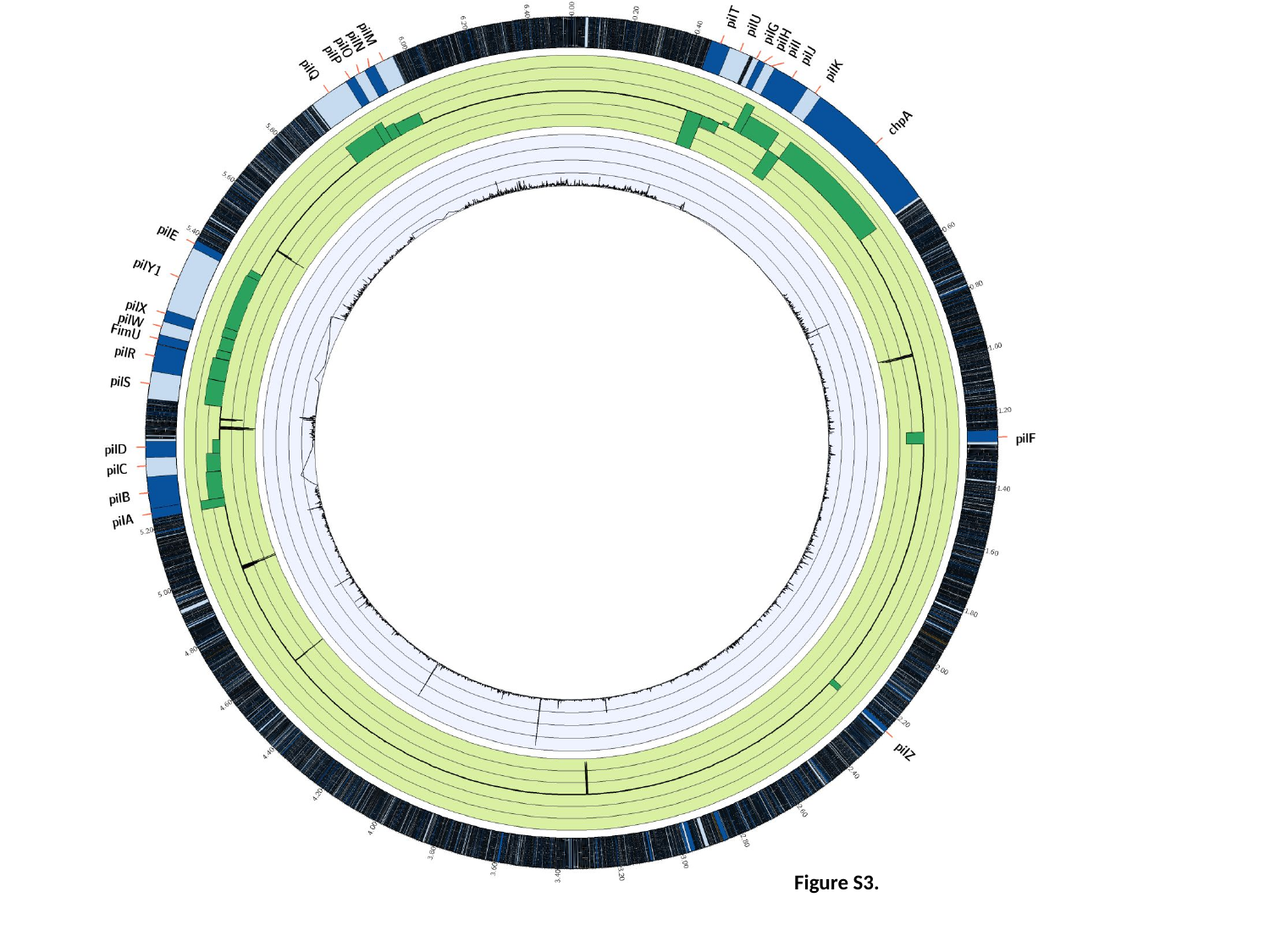

Figure S3.

Supplement: Figure S3 — Detailed analysis of the fitness for mucosal colonization of the genes involved in Type IVa pilus production. Selection for fitness for cecal colonization (green circle) of Tn inserts in genes involved in the production of Type IVa pili. Bars pointing outward from the central circular black line have a positive fitness for colonization, bars pointing toward the center have a negative fitness for cecal colonization and include Tn insertions that do not lead to a defect in piliation (pilK, pilU and pilT) or have a growth defect in LB (pilF). The light and dark blue chromosomal regions in the outermost circle are magnified 60× in relation to the rest of the bacterial genes to highlight the regions of interest. (PPTX) [file ppat.1003582.s003.pptx]

## Slide 1
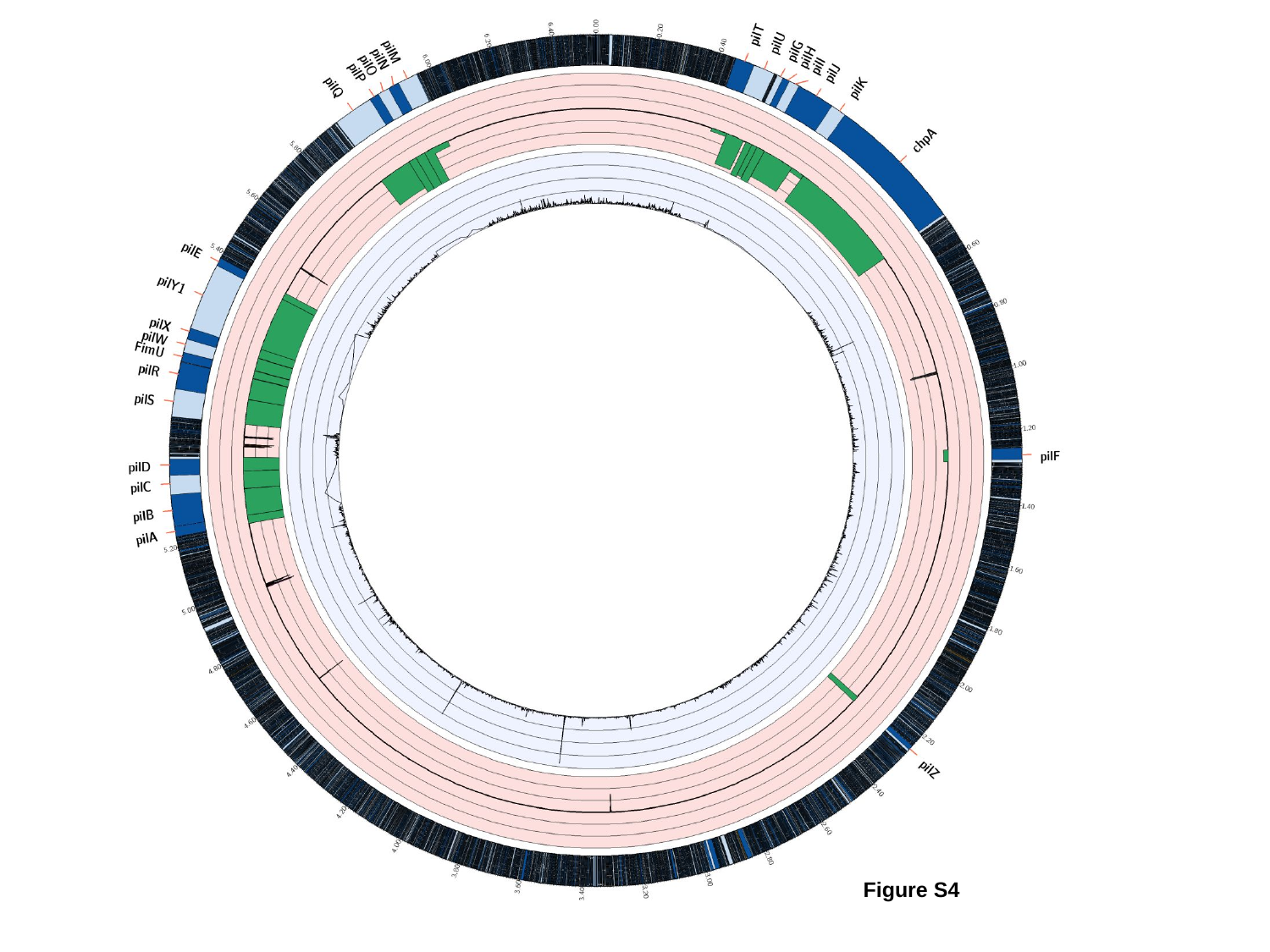

Figure S4

Supplement: Figure S4 — Fitness for systemic dissemination of the Tn insertions in genes for Type IVa pili. All the Tn insertions shown have a negative fitness for systemic dissemination. The light and dark blue chromosomal regions in the outermost circle are magnified 60× in relation to the rest of the bacterial genes to highlight the regions of interest. (PPTX) [file ppat.1003582.s004.pptx]

## Slide 1
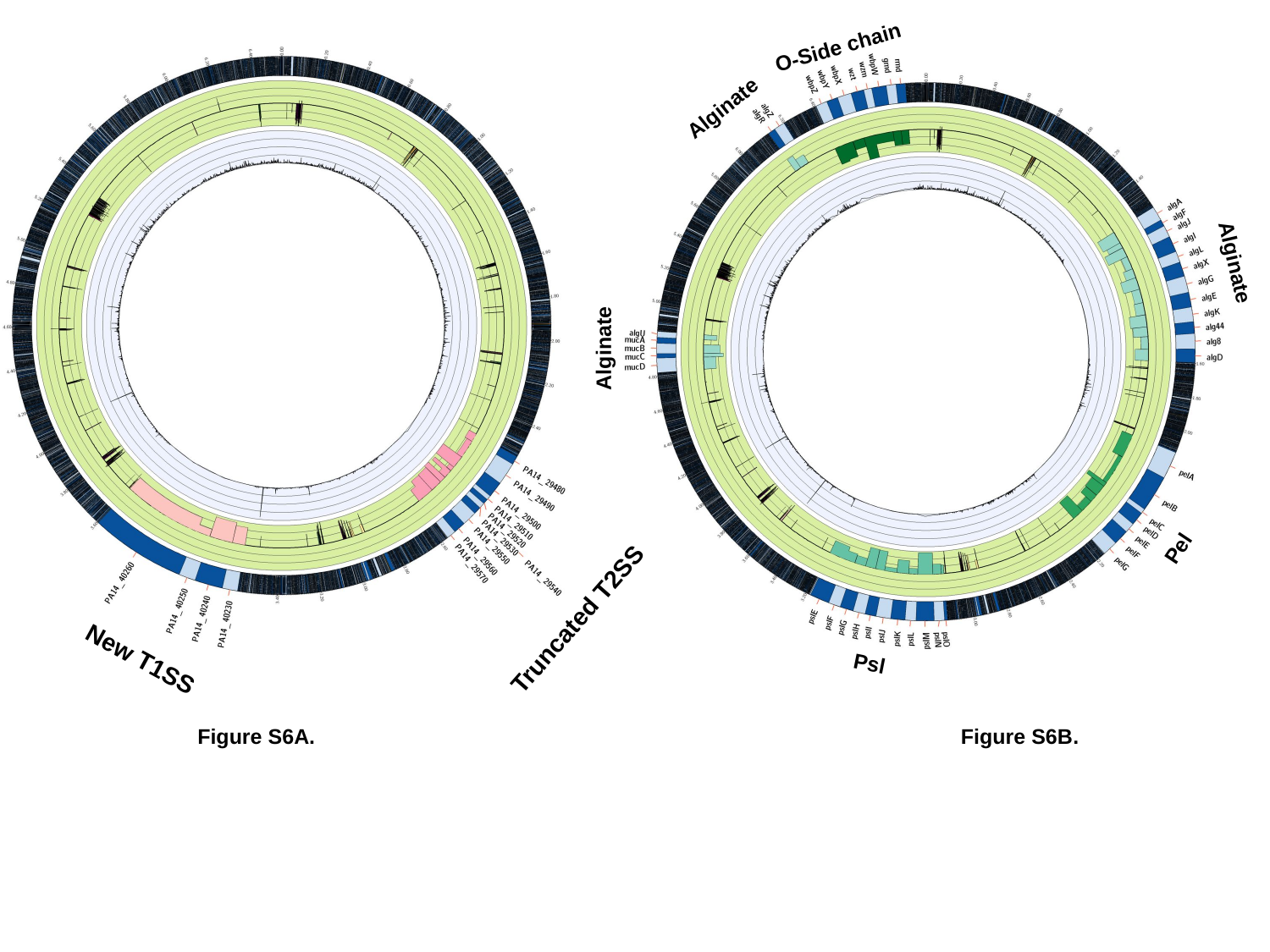

O-Side chain
Alginate
Alginate
Alginate
Pel
Truncated T2SS
New T1SS
Psl
Figure S6A.
Figure S6B.

Supplement: Figure S6 — A. Detailed analysis of the genes and operons important for GI tract colonization identifies a new T1SS (PA14_40230-250) with its probable secreted factor encoded by PA14_40260 and a truncated T2SS. Selection for fitness for cecal colonization (green circle) of Tn inserts in genes within an operon with features of a T1SS and a truncated T2SS similar to HplR-X in P. aeruginosa strain PA01 [35]. All of the genes had a negative fitness for cecal colonization. The light and dark blue chromosomal regions in the outermost circle are magnified 60× in relation to the rest of the bacterial genes to highlight the regions of interest. B. Detailed analysis of the exopolysaccharide/LPS genes and operons important for GI tract colonization. Selection for fitness for cecal colonization (green circle) of Tn insertions in the genes encoding for the LPS O-side chain and three different extracellular polysaccharides, Pel, Psl and alginate. All of the genes had a negative fitness for cecal colonization except the Tn mutants in the algR and algZ, regulatory genes that are also involved in the positive regulation of expression of the Type IVa pili. Increased fitness of these Tn inserts whose loss decreases Type IVa pilus production likely reflects the overall increased fitness for colonization of Type IVa pilus-negative clones. The light and dark blue chromosomal regions in the outermost circle are magnified 60× in relation to the rest of the bacterial genes to highlight the regions of interest. (PPTX) [file ppat.1003582.s006.pptx]

## Slide 1
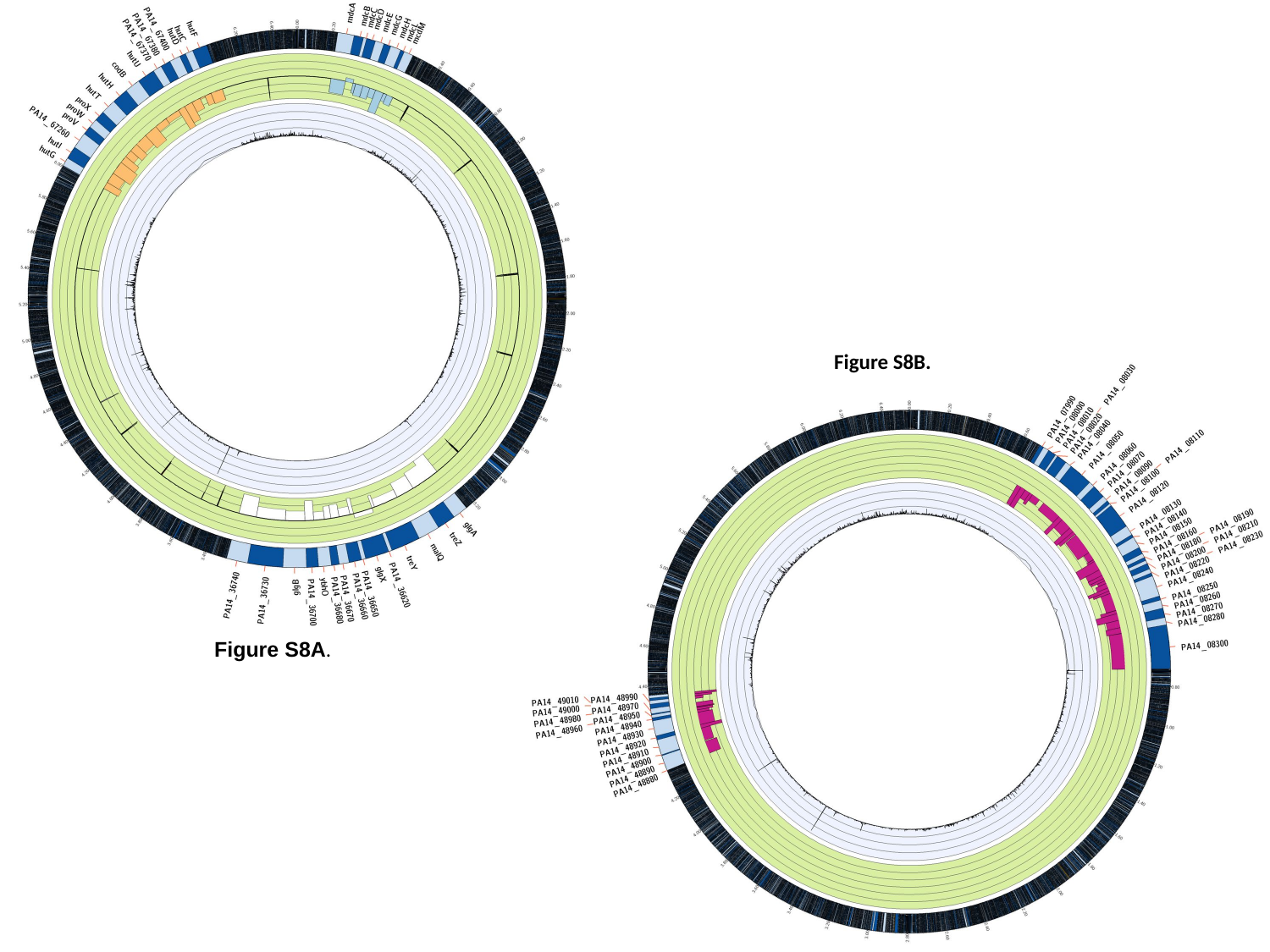

Figure S8B.
Figure S8A.

Supplement: Figure S8 — A. Discordance for selection of Tn insertions in the genes hutD, mdcB and glgX versus the remainder of the genes within their operons. Each circle is as defined in Figure 3. The light and dark blue chromosomal regions in the outermost circle are magnified 60× in relation to the rest of the bacterial genes to highlight the regions of interest. B. In-vivo loss of fitness for the Tn insertions in two prophages of P. aeruginosa strain PA14 encoded by operons of un-annotated genes. Each circle is as defined in Figure 3. The light and dark blue chromosomal regions in the outermost circle are magnified 60× in relation to the rest of the bacterial genes to highlight the regions of interest. (PPTX) [file ppat.1003582.s008.pptx]

## Slide 1
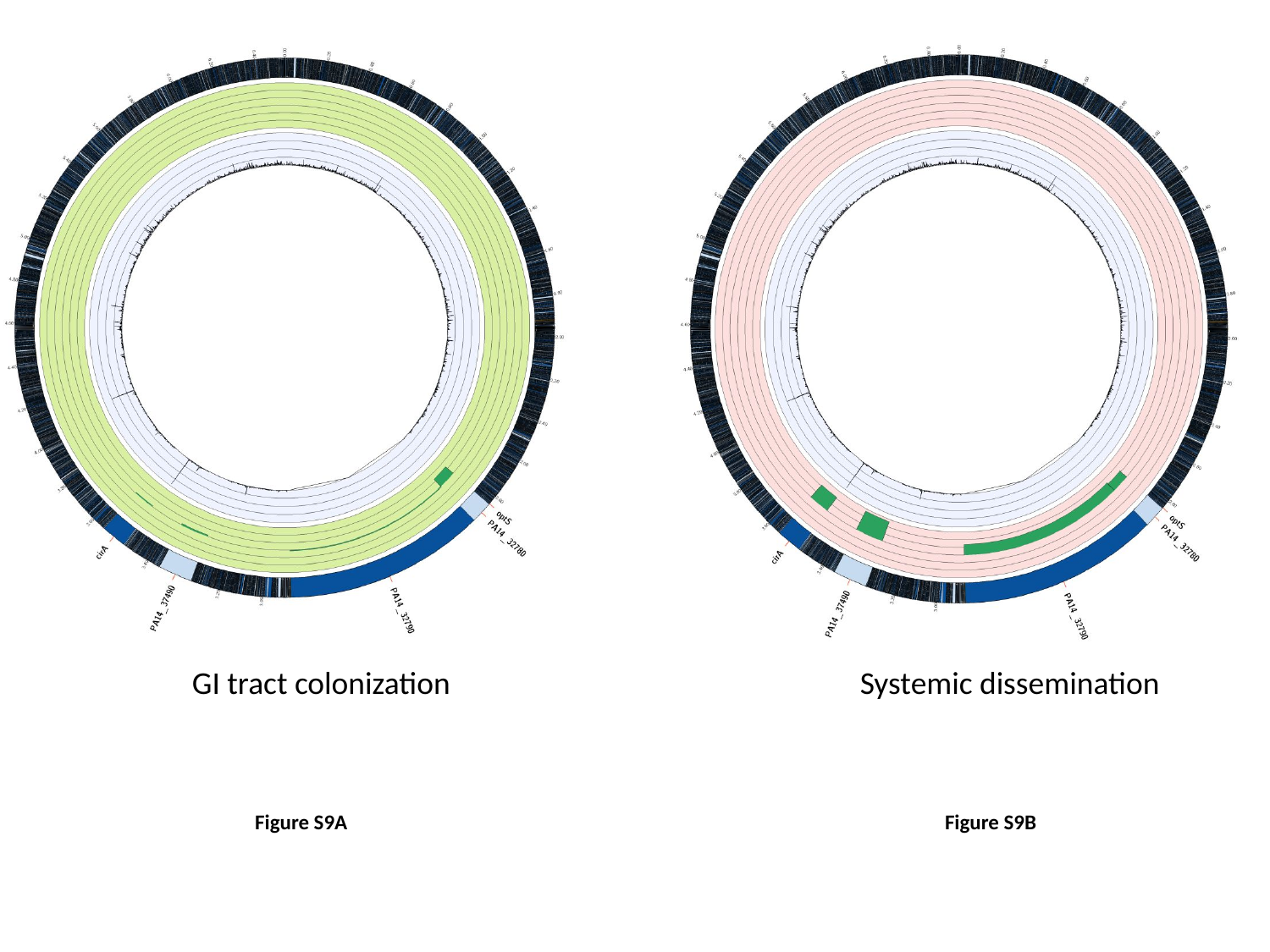

GI tract colonization
Systemic dissemination
Figure S9A
Figure S9B

Supplement: Figure S9 — Potential targets for immunotherapy. These Tn insertions have no defect in GI tract colonization (A) but are not able to disseminate to the spleen during jneutropenia (B). The light and dark blue chromosomal regions in the outermost circle are magnified 60× in relation to the rest of the bacterial genes to highlight the regions of interest. (PPTX) [file ppat.1003582.s009.pptx]

## Slide 1
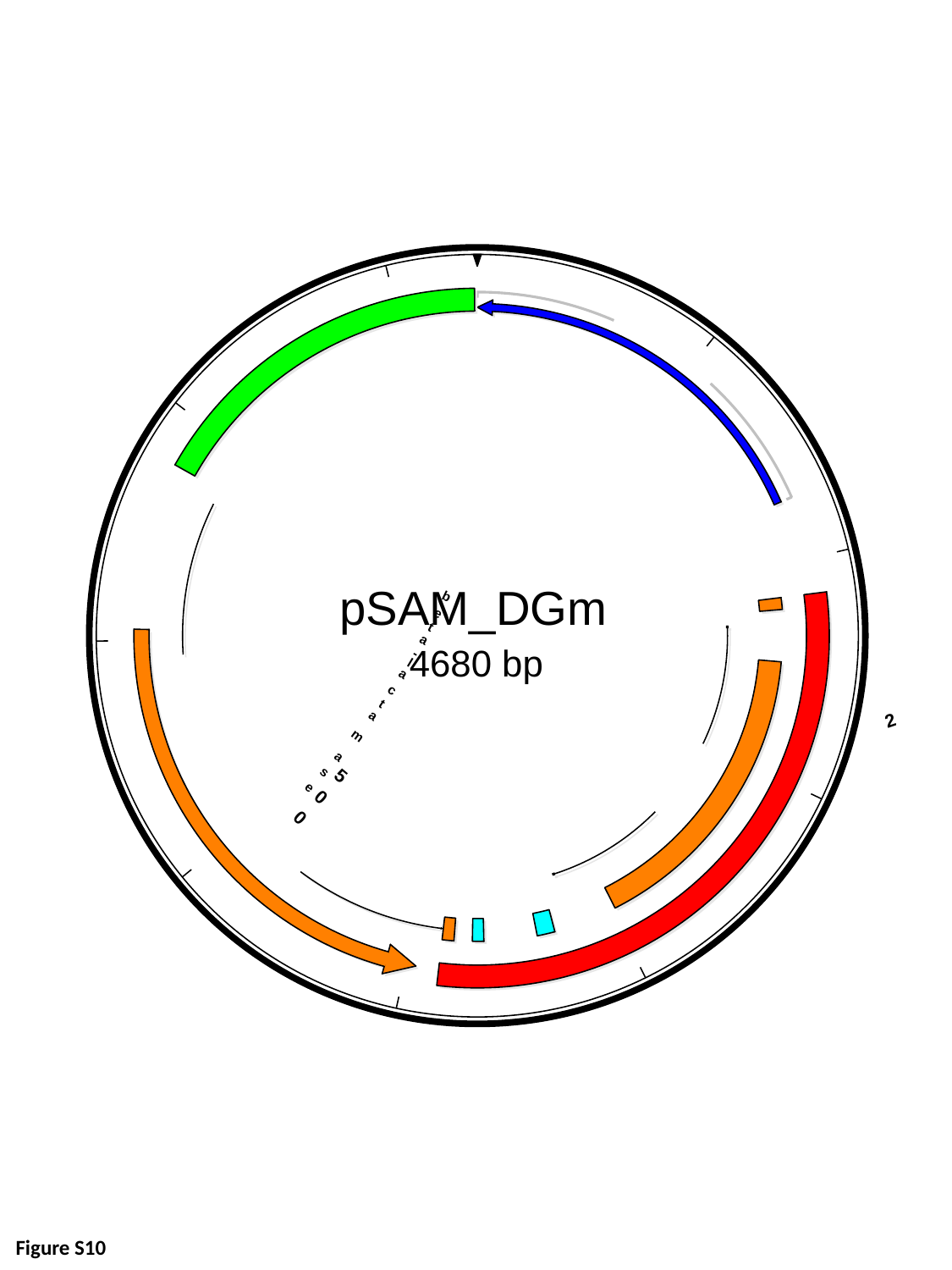

Figure S10

Supplement: Figure S10 — Map of pSAM-DGm used to create the Tn-insertion library. The two recognition sites for the MmeI enzyme are represented by the two small orange rectangles at the beginning and end of the mariner-based transposon. Gm = Gentamicin. (PPTX) [file ppat.1003582.s010.pptx]
