# Supplementary material for: A Comprehensive Analysis of In Vitro and In Vivo Genetic Fitness of Pseudomonas aeruginosa Using High-Throughput Sequencing of Transposon Libraries
Source: PLoS Pathog. 2013 Sep 5;9(9):e1003582. doi: 10.1371/journal.ppat.1003582 (PMC3764216; doi:10.1371/journal.ppat.1003582)
Supplement: Figure S5 — Reduced in vivo fitness of the Tn insertions in the genes encoding for the VFs of P. aeruginosa. Details of the genes. Each circle is as defined in Figure 3. The light and dark blue chromosomal regions in the outermost circle are magnified 60× in relation to the rest of the bacterial genes to highlight the regions of interest. (PPTX) [file ppat.1003582.s005.pptx]

## Slide 1
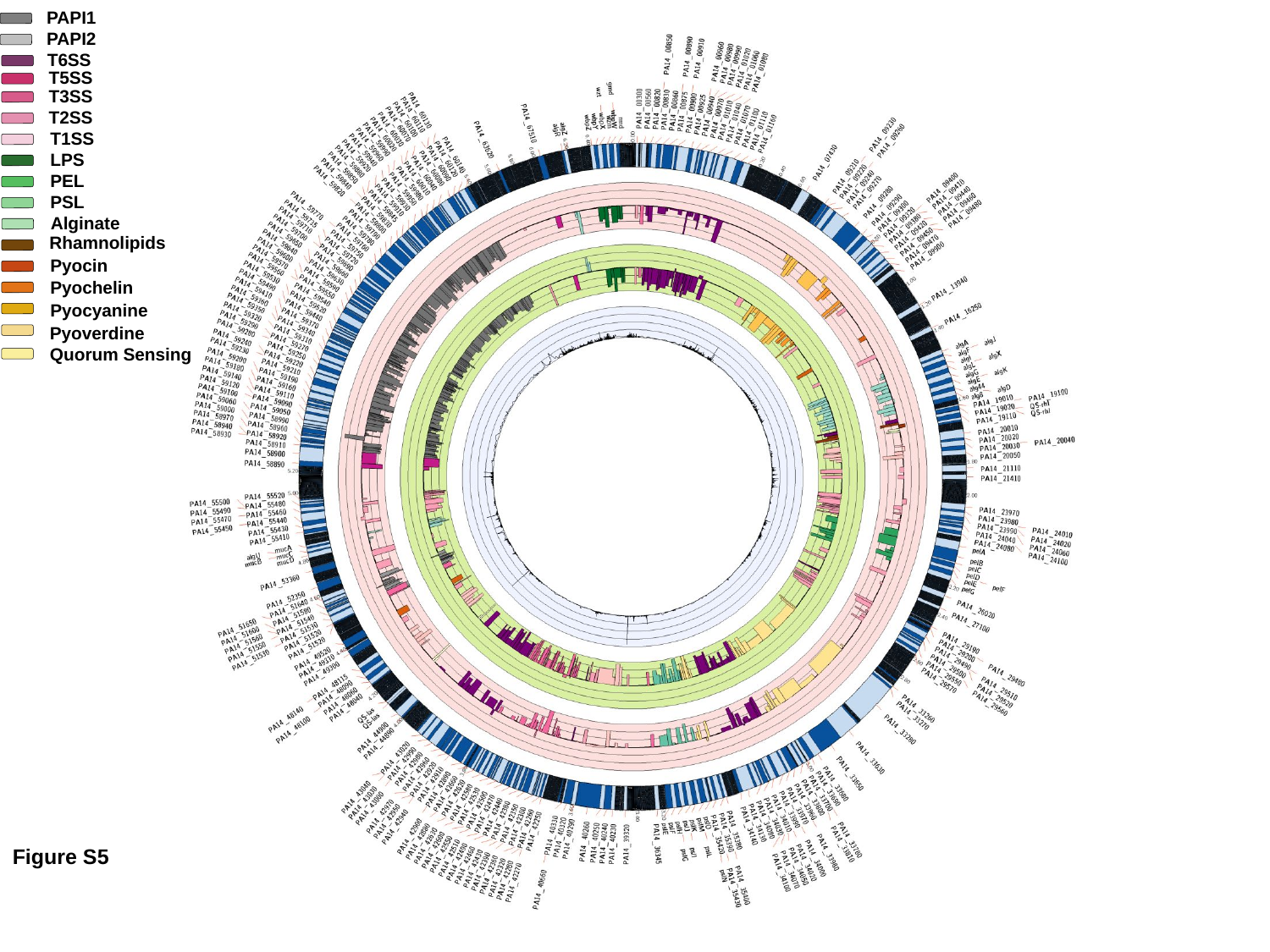

PAPI1
PAPI2
T6SS
T5SS
T3SS
T2SS
T1SS
LPS
PEL
PSL
Alginate
Rhamnolipids
Pyocin
Pyochelin
Pyocyanine
Pyoverdine
Quorum Sensing
Figure S5
