# Supplementary material for: A Comprehensive Analysis of In Vitro and In Vivo Genetic Fitness of Pseudomonas aeruginosa Using High-Throughput Sequencing of Transposon Libraries
Source: PLoS Pathog. 2013 Sep 5;9(9):e1003582. doi: 10.1371/journal.ppat.1003582 (PMC3764216; doi:10.1371/journal.ppat.1003582)
Supplement: Figure S7 — Fitness changes in Tn insertions in genes involved in nutrient utilization. Each circle is as defined in Figure 3. The light and dark blue chromosomal regions in the outermost circle are magnified 60× in relation to the rest of the bacterial genes to highlight the regions of interest. (PPTX) [file ppat.1003582.s007.pptx]

## Slide 1
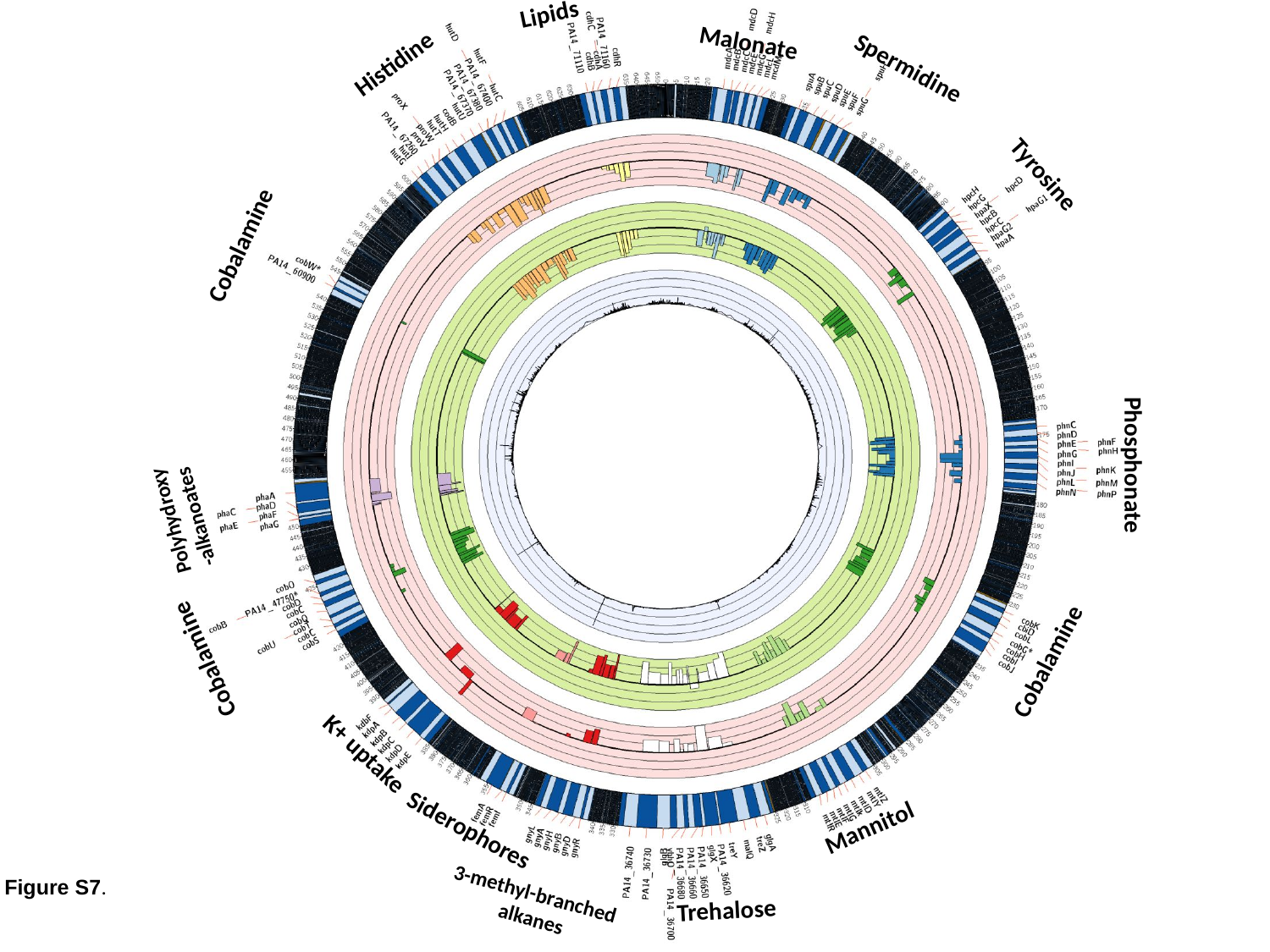

Lipids
Malonate
Histidine
Spermidine
Tyrosine
Cobalamine
Phosphonate
Polyhydroxy
-alkanoates
Cobalamine
Cobalamine
K+ uptake
Mannitol
Siderophores
Figure S7.
3-methyl-branched
 alkanes
Trehalose
