# Supplementary material for: A Comprehensive Analysis of In Vitro and In Vivo Genetic Fitness of Pseudomonas aeruginosa Using High-Throughput Sequencing of Transposon Libraries
Source: PLoS Pathog. 2013 Sep 5;9(9):e1003582. doi: 10.1371/journal.ppat.1003582 (PMC3764216; doi:10.1371/journal.ppat.1003582)
Supplement: Figure S11 — Principle for the preparation of the saturated Tn-insertion library for high-throughput sequencing. (PPTX) [file ppat.1003582.s011.pptx]

## Slide 1
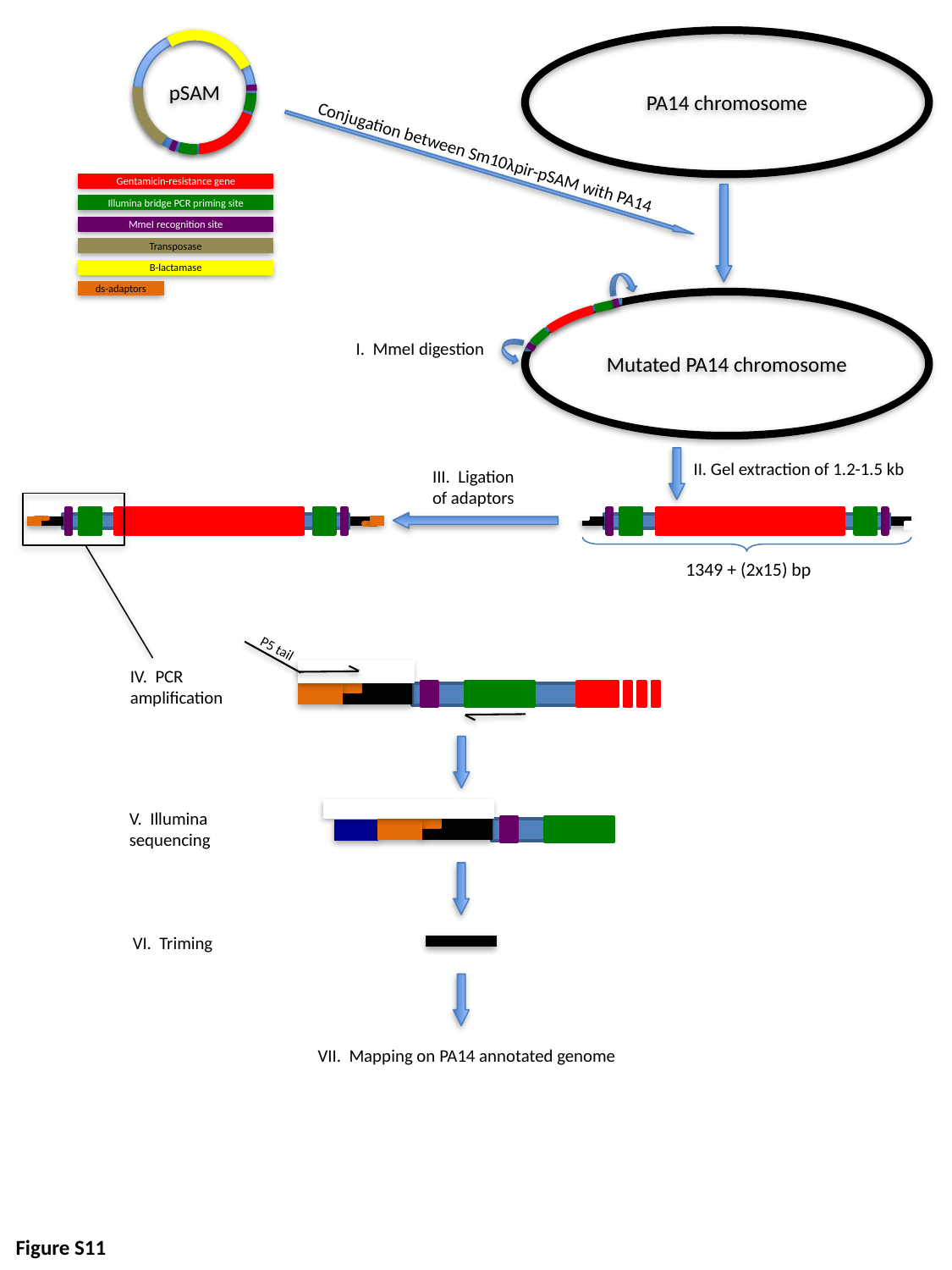

PA14 chromosome
pSAM
Conjugation between Sm10λpir-pSAM with PA14
Gentamicin-resistance gene
Illumina bridge PCR priming site
MmeI recognition site
Transposase
Β-lactamase
ds-adaptors
Mutated PA14 chromosome
I. MmeI digestion
II. Gel extraction of 1.2-1.5 kb
III. Ligation
of adaptors
1349 + (2x15) bp
P5 tail
IV. PCR
amplification
V. Illumina
sequencing
VI. Triming
VII. Mapping on PA14 annotated genome
Figure S11
