# Supplementary material for: A Comprehensive Analysis of In Vitro and In Vivo Genetic Fitness of Pseudomonas aeruginosa Using High-Throughput Sequencing of Transposon Libraries
Source: PLoS Pathog. 2013 Sep 5;9(9):e1003582. doi: 10.1371/journal.ppat.1003582 (PMC3764216; doi:10.1371/journal.ppat.1003582)
Supplement: Table S2 — Tn-insertions into P. aeruginosa PA14 genes with more than 1,000 sequencing reads after growth in LB. (DOC) [file ppat.1003582.s013.doc]

| Table S2: Tn-insertions into *P. aeruginosa* PA14 genes with more than 1,000 sequencing reads after growth in LB | | | |  |  |  |
| --- | --- | --- | --- | --- | --- | --- |
| ID | Gene name | Product Name | Functional Class | Subcellular Localization | Normalized means | Percent AT |
| PA14_03166 | PA14_03166 | hypothetical protein | Hypothetical, unclassified, unknown | Unknown [Class 3] | 1002.5 | 47 |
| PA14_23350 | orfA | hypothetical protein | Cell wall / LPS / capsule | Cytoplasmic Membrane [Class 3] | 1004.9 | 43 |
| PA14_35710 | PA14_35710 | hypothetical protein | Hypothetical, unclassified, unknown | Unknown [Class 3] | 1005.4 | 49 |
| PA14_69510 | PA14_69510 | Hypothetical protein | Hypothetical, unclassified, unknown | Cytoplasmic Membrane [Class 3] | 1011.3 | 43 |
| PA14_69480 | algZ | DNA binding-protein | Transcriptional regulators | Cytoplasmic [Class 3] | 1018.3 | 37 |
| PA14_03340 | PA14_03340 | hypothetical protein | Hypothetical, unclassified, unknown | Cytoplasmic [Class 3] | 1031.9 | 41 |
| PA14_57970 | PA14_57970 | hypothetical protein | Putative enzymes | Cytoplasmic [Class 3] | 1038.8 | 37 |
| PA14_73150 | PA14_73150 | hypothetical protein | Transport of small molecules | Cytoplasmic [Class 3] | 1087.1 | 35 |
| PA14_60480 | PA14_60480 | hypothetical protein | Hypothetical, unclassified, unknown | Unknown [Class 3] | 1092.2 | 39 |
| PA14_53070 | hpd | 4-hydroxyphenylpyruvate dioxygenase | Amino acid biosynthesis and metabolism | Cytoplasmic [Class 3] | 1100.6 | 38 |
| PA14_06875 | rsmY | rsmYregulatoryRNA | Non-coding RNA gene | Unknown [Class 3] | 1108.2 | 45 |
| PA14_51340 | mvfR | Transcriptional regulator MvfR | Transcriptional regulators | Cytoplasmic [Class 3] | 1138.9 | 37 |
| PA14_60280 | fimU | type 4 fimbrial biogenesis protein FimU | Motility & Attachment | Unknown [Class 3] | 1178.9 | 45 |
| PA14_62790 | PA14_62790 | tRNA-Met | Non-coding RNA gene | Unknown [Class 3] | 1259.5 | 38 |
| PA14_41570 | oprF | major porin and structural outer membrane porin OprF precursor | Membrane proteins | Outer Membrane [Class 3] | 1337.3 | 40 |
| PA14_72770 | PA14_72770 | hypothetical protein | Hypothetical, unclassified, unknown | Cytoplasmic [Class 3] | 1358.9 | 33 |
| PA14_21210 | PA14_21210 |  |  |  | 1359.0 | 37 |
| PA14_05300 | PA14_05300 | TonB domain-containing protein | Membrane proteins | Unknown [Class 3] | 1385.3 | 32 |
| PA14_21850 | PA14_21850 | putative transcriptional regulator | Transcriptional regulators | Unknown [Class 3] | 1402.5 | 30 |
| PA14_38380 | amrR | putative transcriptional regulator | Transcriptional regulators | Cytoplasmic [Class 3] | 1404.5 | 33 |
| PA14_70180 | rpmG | 50S ribosomal protein L33 | Translation, post-translational modification, degradation | Cytoplasmic [Class 3] | 1426.1 | 49 |
| PA14_24650 | rmf | ribosome modulation factor | Translation, post-translational modification, degradation | Unknown [Class 3] | 1460.6 | 44 |
| PA14_70350 | PA14_70350 | hypothetical protein | Fatty acid and phospholipid metabolism | Cytoplasmic [Class 3] | 1462.9 | 34 |
| PA14_70640 | rubA1 | Rubredoxin 1 | Carbon compound catabolism | Cytoplasmic [Class 3] | 1471.1 | 40 |
| PA14_70300 | PA14_70300 | putative enzyme | Energy metabolism | Cytoplasmic [Class 3] | 1523.7 | 37 |
| PA14_23570 | PA14_23570 | tRNA-Ala | Non-coding RNA gene | Unknown [Class 3] | 1550.1 | 40 |
| PA14_01510 | PA14_01510 | hypothetical protein | Hypothetical, unclassified, unknown | Unknown [Class 3] | 1602.1 | 38 |
| PA14_61020 | ankB | hypothetical protein | Two-component regulatory systems | Unknown [Class 3] | 1610.8 | 31 |
| PA14_21480 | PA14_21480 | hypothetical protein | Hypothetical, unclassified, unknown | Unknown [Class 3] | 1637.7 | 53 |
| PA14_51580 | PA14_51580 | hypothetical protein | Hypothetical, unclassified, unknown | Unknown [Class 3] | 1867.3 | 43 |
| PA14_52060 | PA14_52060 | hypothetical protein | Hypothetical, unclassified, unknown | Periplasmic [Class 3] | 1877.2 | 40 |
| PA14_04410 | ptsP | phosphoenolpyruvate-protein phosphotransferase PtsP | Transport of small molecules | Cytoplasmic [Class 3] | 2000.7 | 33 |
| PA14_59970 | PA14_59970 | hypothetical protein | Hypothetical, unclassified, unknown | Unknown [Class 3] | 2010.4 | 45 |
| PA14_08640 | PA14_08640 | hypothetical protein | Hypothetical, unclassified, unknown | Unknown [Class 3] | 2155.7 | 39 |
| PA14_45950 | rsaL | regulatory protein RsaL | Transcriptional regulators | Unknown [Class 3] | 2240.3 | 39 |
| PA14_60320 | pilE | type 4 fimbrial biogenesis protein PilE | Motility & Attachment | Fimbrial [Class 3] ; Extracellular [Class 3] | 2247.1 | 48 |
| PA14_69020 | PA14_69020 | hypothetical protein | Hypothetical, unclassified, unknown | Cytoplasmic [Class 3] | 2319.9 | 41 |
| PA14_35720 | PA14_35720 | hypothetical protein | Hypothetical, unclassified, unknown | Cytoplasmic [Class 3] | 2327.4 | 46 |
| PA14_58740 | PA14_58740 | hypothetical protein | Hypothetical, unclassified, unknown | Unknown [Class 3] | 2401.9 | 42 |
| PA14_57690 | PA14_57690 | hypothetical protein | Hypothetical, unclassified, unknown | Unknown [Class 3] | 2449.8 | 32 |
| PA14_60060 | PA14_60060 | hypothetical protein | Hypothetical, unclassified, unknown | Unknown [Class 3] | 2520.0 | 47 |
| PA14_53420 | btuE | glutathione peroxidase | Putative enzymes | Periplasmic [Class 3] | 3159.8 | 35 |
| PA14_08370 | vfr | cAMP-regulatory protein | Transcriptional regulators | Cytoplasmic [Class 3] | 4112.3 | 39 |
| PA14_45960 | lasR | transcriptional regulator LasR | Transcriptional regulators | Cytoplasmic [Class 3] | 6067.5 | 41 |
| PA14_39470 | PA14_39470 | hypothetical protein | Hypothetical, unclassified, unknown | Cytoplasmic [Class 3] | 7399.3 | 57 |
|  |  |  |  |  | Average= | 40.3 |
