# Supplementary material for: A Comprehensive Analysis of In Vitro and In Vivo Genetic Fitness of Pseudomonas aeruginosa Using High-Throughput Sequencing of Transposon Libraries
Source: PLoS Pathog. 2013 Sep 5;9(9):e1003582. doi: 10.1371/journal.ppat.1003582 (PMC3764216; doi:10.1371/journal.ppat.1003582)
Supplement: Table S3 — Genes (N = 129) identified with minimal changes (<2-fold) in the number of sequencing reads when comparing sequencing reads obtained from bacteria grown in LB to those from bacteria recovered from the cecum (minimum 10 sequencing reads in cecum). (DOC) [file ppat.1003582.s014.doc]

| Table S3: Genes (N=129) identified with minimal changes (<2-fold) in the number of sequencing reads when comparing sequencing reads obtained from bacteria grown in LB to those from bacteria recovered from the cecum (minimum 10 sequencing reads in cecum) | | | | |  |  |
| --- | --- | --- | --- | --- | --- | --- |
| ID | Gene name | Product Name | Functional Class | Subcellular Localization | Ratio Cecum/LB | Ratio Spleen/Cecum |
| PA14_32790 | PA14_32790 | hypothetical protein | Hypothetical, unclassified, unknown | Outer Membrane [Class 3] | 1.2 | 0.0 |
| PA14_65030 | PA14_65030 | hypothetical protein | Membrane proteins | Outer Membrane [Class 3] | 0.8 | 0.0 |
| PA14_39650 | cirA | putative TonB-dependent receptor | Transport of small molecules | Outer Membrane [Class 3] | 0.7 | 0.0 |
| PA14_32740 | optS | TonB-dependent receptor | Transport of small molecules | Outer Membrane [Class 3] | 0.6 | 0.0 |
| PA14_37490 | PA14_37490 | putative TonB-dependent receptor | Transport of small molecules | Outer Membrane [Class 3] | 0.6 | 0.0 |
| PA14_45100 | muiA | hypothetical protein | Transport of small molecules | Outer Membrane [Class 3] | 0.5 | 0.0 |
| PA14_64920 | PA14_64920 | putative methyl-accepting chemotaxis protein | Chemotaxis | Outer Membrane [Class 3] | 1.4 | 0.0 |
| PA14_06000 | clpA | putative ClpA/B protease ATP binding subunit | Translation, post-translational modification, degradation | Cytoplasmic [Class 3] | 1.9 | 0.0 |
| PA14_15230 | xseA | exodeoxyribonuclease VII large subunit | DNA replication, recombination, modification and repair | Cytoplasmic [Class 3] | 1.9 | 0.0 |
| PA14_21370 | fadD1 | long-chain-fatty-acid--CoA ligase | Fatty acid and phospholipid metabolism | Cytoplasmic [Class 3] | 1.9 | 0.0 |
| PA14_18850 | PA14_18850 | adenylosuccinate lyase | Carbon compound catabolism | Cytoplasmic [Class 3] | 1.9 | 0.0 |
| PA14_42300 | pscG | type III export protein PscG | Protein secretion/export apparatus | Cytoplasmic [Class 3] | 1.8 | 0.0 |
| PA14_42720 | masA | enolase-phosphatase | Amino acid biosynthesis and metabolism | Cytoplasmic [Class 3] | 1.7 | 0.0 |
| PA14_51890 | PA14_51890 | hypothetical protein | Hypothetical, unclassified, unknown | Cytoplasmic [Class 3] | 1.6 | 0.0 |
| PA14_70180 | rpmG | 50S ribosomal protein L33 | Translation, post-translational modification, degradation | Cytoplasmic [Class 3] | 1.5 | 0.0 |
| PA14_17050 | map | methionine aminopeptidase | Translation, post-translational modification, degradation | Cytoplasmic [Class 3] | 1.5 | 0.0 |
| PA14_01400 | elaA | hypothetical protein | Transport of small molecules | Cytoplasmic [Class 3] | 1.4 | 0.0 |
| PA14_57710 | cysN | bifunctional sulfate adenylyltransferase subunit 1/adenylylsulfate kinase protein | Amino acid biosynthesis and metabolism | Cytoplasmic [Class 3] | 1.4 | 0.0 |
| PA14_05320 | pilG | twitching motility protein PilG | Two-component regulatory systems | Cytoplasmic [Class 3] | 1.3 | 0.0 |
| PA14_68580 | pckA | phosphoenolpyruvate carboxykinase | Energy metabolism | Cytoplasmic [Class 3] | 1.3 | 0.0 |
| PA14_41220 | lon | Lon protease | Translation, post-translational modification, degradation | Cytoplasmic [Class 3] | 1.3 | 0.0 |
| PA14_68360 | PA14_68360 | putative beta-ketoacyl synthase | Fatty acid and phospholipid metabolism | Cytoplasmic [Class 3] | 1.3 | 0.0 |
| PA14_49590 | PA14_49590 | transcriptional regulator | Transcriptional regulators | Cytoplasmic [Class 3] | 1.2 | 0.0 |
| PA14_21690 | lhr1 | putative ATP-dependent DNA helicase | DNA replication, recombination, modification and repair | Cytoplasmic [Class 3] | 1.2 | 0.0 |
| PA14_08370 | vfr | cAMP-regulatory protein | Transcriptional regulators | Cytoplasmic [Class 3] | 1.2 | 0.0 |
| PA14_11050 | moaF | hypothetical protein | Hypothetical, unclassified, unknown | Cytoplasmic [Class 3] | 1.2 | 0.0 |
| PA14_08680 | tufB | elongation factor Tu | Translation, post-translational modification, degradation | Cytoplasmic [Class 3] | 1.1 | 0.0 |
| PA14_00450 | trpB | tryptophan synthase subunit beta | Amino acid biosynthesis and metabolism | Cytoplasmic [Class 3] | 1.1 | 0.0 |
| PA14_05330 | pilH | twitching motility protein PilH | Two-component regulatory systems | Cytoplasmic [Class 3] | 1.1 | 0.0 |
| PA14_03770 | speB1 | agmatinase | Amino acid biosynthesis and metabolism | Cytoplasmic [Class 3] | 1.0 | 0.0 |
| PA14_08040 | gpI | putative phage tail protein | Related to phage, transposon, or plasmid | Cytoplasmic [Class 3] | 1.0 | 0.0 |
| PA14_42140 | PA14_42140 | transglutaminase-like domain-containing protein | Amino acid biosynthesis and metabolism | Cytoplasmic [Class 3] | 1.0 | 0.0 |
| PA14_22460 | PA14_22460 | alpha/beta family hydrolase | Putative enzymes | Cytoplasmic [Class 3] | 0.9 | 0.0 |
| PA14_34680 | PA14_34680 | putative oxidoreductase | Amino acid biosynthesis and metabolism | Cytoplasmic [Class 3] | 0.9 | 0.0 |
| PA14_52700 | aruF | arginine/ornithine succinyltransferase AI subunit | Amino acid biosynthesis and metabolism | Cytoplasmic [Class 3] | 0.9 | 0.0 |
| PA14_30200 | cspD | cold-shock protein CspD | Transcriptional regulators | Cytoplasmic [Class 3] | 0.9 | 0.0 |
| PA14_32100 | xylY | toluate 1,2-dioxygenase beta subunit | Carbon compound catabolism | Cytoplasmic [Class 3] | 0.8 | 0.0 |
| PA14_04930 | rpoH | RNA polymerase factor sigma-32 | Transcriptional regulators | Cytoplasmic [Class 3] | 0.8 | 0.0 |
| PA14_27590 | PA14_27590 | hypothetical protein | Hypothetical, unclassified, unknown | Cytoplasmic [Class 3] | 0.7 | 0.0 |
| PA14_44070 | gltA | type II citrate synthase | Energy metabolism | Cytoplasmic [Class 3] | 0.7 | 0.0 |
| PA14_43480 | PA14_43480 | GntR family transcriptional regulator | Transcriptional regulators | Cytoplasmic [Class 3] | 0.7 | 0.0 |
| PA14_24590 | PA14_24590 | hypothetical protein | Hypothetical, unclassified, unknown | Cytoplasmic [Class 3] | 0.7 | 0.0 |
| PA14_66260 | ilvE | branched-chain amino acid aminotransferase | Amino acid biosynthesis and metabolism | Cytoplasmic [Class 3] | 0.7 | 0.0 |
| PA14_12450 | PA14_12450 | putative acyl-CoA dehydrogenase | Fatty acid and phospholipid metabolism | Cytoplasmic [Class 3] | 0.7 | 0.0 |
| PA14_48730 | PA14_48730 | hypothetical protein | Hypothetical, unclassified, unknown | Cytoplasmic [Class 3] | 0.7 | 0.0 |
| PA14_70420 | rph | ribonuclease PH | Transcription, RNA processing and degradation | Cytoplasmic [Class 3] | 0.6 | 0.0 |
| PA14_31270 | PA14_31270 | hypothetical protein | Hypothetical, unclassified, unknown | Cytoplasmic [Class 3] | 0.6 | 0.0 |
| PA14_09890 | ppiC2 | peptidyl-prolyl cis-trans isomerase C2 | Chaperones & heat shock proteins | Cytoplasmic [Class 3] | 0.6 | 0.0 |
| PA14_69450 | hemC | porphobilinogen deaminase | Central intermediary metabolism | Cytoplasmic [Class 3] | 0.6 | 0.0 |
| PA14_51900 | proS | prolyl-tRNA synthetase | Translation, post-translational modification, degradation | Cytoplasmic [Class 3] | 0.6 | 0.0 |
| PA14_17570 | PA14_17570 | hypothetical protein | Translation, post-translational modification, degradation | Cytoplasmic [Class 3] | 0.6 | 0.0 |
| PA14_20740 | flgA | flagellar basal body P-ring biosynthesis protein FlgA | Motility & Attachment | Cytoplasmic [Class 3] | 0.6 | 0.0 |
| PA14_30650 | gacA | response regulator GacA | Transcriptional regulators | Cytoplasmic [Class 3] | 0.6 | 0.0 |
| PA14_51330 | nadA | quinolinate synthetase | Biosynthesis of cofactors, prosthetic groups and carriers | Cytoplasmic [Class 3] | 0.6 | 0.0 |
| PA14_18160 | PA14_18160 | putative oxidoreductase | Energy metabolism | Cytoplasmic [Class 3] | 0.5 | 0.0 |
| PA14_47100 | ilvA2 | threonine dehydratase | Amino acid biosynthesis and metabolism | Cytoplasmic [Class 3] | 0.5 | 0.0 |
| PA14_43270 | ybbB | tRNA 2-selenouridine synthase | Putative enzymes | Cytoplasmic [Class 3] | 0.5 | 0.0 |
| PA14_71460 | glyA1 | serine hydroxymethyltransferase | Amino acid biosynthesis and metabolism | Cytoplasmic [Class 3] | 0.5 | 0.0 |
| PA14_63150 | pmrA | two-component response regulator | Two-component regulatory systems | Cytoplasmic [Class 3] | 0.5 | 0.0 |
| PA14_43760 | PA14_43760 | hypothetical protein | Hypothetical, unclassified, unknown | Cytoplasmic [Class 3] | 0.5 | 0.0 |
| PA14_45180 | PA14_45180 | putative oxidoreductase | Putative enzymes | Cytoplasmic [Class 3] | 0.5 | 0.0 |
| PA14_50530 | braD | branched-chain amino acid transport protein BraD | Membrane proteins | Cytoplasmic Membrane [Class 3] | 1.8 | 0.0 |
| PA14_61370 | PA14_61370 | hypothetical protein | Hypothetical, unclassified, unknown | Cytoplasmic Membrane [Class 3] | 1.3 | 0.0 |
| PA14_56520 | PA14_56520 | hypothetical protein | Hypothetical, unclassified, unknown | Cytoplasmic Membrane [Class 3] | 1.2 | 0.0 |
| PA14_67810 | ctpA | putative carboxyl-terminal protease | Related to phage, transposon, or plasmid | Cytoplasmic Membrane [Class 3] | 1.0 | 0.1 |
| PA14_65540 | fimX | hypothetical protein | Motility & Attachment | Cytoplasmic Membrane [Class 3] | 0.8 | 0.0 |
| PA14_62520 | PA14_62520 | hypothetical protein | Hypothetical, unclassified, unknown | Cytoplasmic Membrane [Class 3] | 0.8 | 0.0 |
| PA14_69850 | betT | putative choline transporter | Transport of small molecules | Cytoplasmic Membrane [Class 3] | 0.7 | 0.0 |
| PA14_68300 | arcD | arginine/ornithine antiporter | Amino acid biosynthesis and metabolism | Cytoplasmic Membrane [Class 3] | 0.7 | 0.0 |
| PA14_50200 | fleS | two-component sensor | Two-component regulatory systems | Cytoplasmic Membrane [Class 3] | 0.7 | 0.0 |
| PA14_21750 | PA14_21750 | hypothetical protein | Fatty acid and phospholipid metabolism | Cytoplasmic Membrane [Class 3] | 0.7 | 0.0 |
| PA14_29850 | nuoN | NADH dehydrogenase subunit N | Energy metabolism | Cytoplasmic Membrane [Class 3] | 0.7 | 0.0 |
| PA14_37850 | yejE | putative permease of ABC transporter | Transport of small molecules | Cytoplasmic Membrane [Class 3] | 0.6 | 0.0 |
| PA14_45970 | PA14_45970 | putative cation-transporting P-type ATPase | Transport of small molecules | Cytoplasmic Membrane [Class 3] | 0.6 | 0.0 |
| PA14_12160 | PA14_12160 | putative murein transglycosylase | Putative enzymes | Cytoplasmic Membrane [Class 3] | 0.6 | 0.0 |
| PA14_17640 | potA | polyamine transport protein PotA | Transport of small molecules | Cytoplasmic Membrane [Class 3] | 0.6 | 0.0 |
| PA14_36360 | PA14_36360 | hypothetical protein | Membrane proteins | Cytoplasmic Membrane [Class 3] | 0.6 | 0.0 |
| PA14_12080 | sltB1 | soluble lytic transglycosylase B | Cell wall / LPS / capsule | Cytoplasmic Membrane [Class 3] | 0.5 | 0.0 |
| PA14_39750 | PA14_39750 | putative amino acid permease | Transport of small molecules | Cytoplasmic Membrane [Class 3] | 0.5 | 0.0 |
| PA14_44950 | ygfU | putative transporter | Transport of small molecules | Cytoplasmic Membrane [Class 3] | 0.5 | 0.0 |
| PA14_67970 | PA14_67970 | hypothetical protein | Putative enzymes | Cytoplasmic Membrane [Class 3] | 0.5 | 0.0 |
| PA14_69300 | PA14_69300 | hypothetical protein | Transport of small molecules | Cytoplasmic Membrane [Class 3] | 0.5 | 0.0 |
| PA14_53360 | plcH | hemolytic phospholipase C precursor | Secreted Factors (toxins, enzymes, alginate) | Extracellular [Class 3] | 0.7 | 0.0 |
| PA14_50340 | flgL | flagellar hook-associated protein FlgL | Motility & Attachment | Flagellar [Class 3] ; Extracellular [Class 3] | 0.9 | 0.0 |
| PA14_50290 | fliC | flagellin type B | Motility & Attachment | Flagellar [Class 3] ; Extracellular [Class 3] | 0.6 | 0.0 |
| PA14_50480 | flgB | flagellar basal body rod protein FlgB | Motility & Attachment | Flagellar [Class 3] ; Periplasmic [Class 3] | 0.9 | 0.0 |
| PA14_07250 | PA14_07250 | hypothetical protein | Putative enzymes | Periplasmic [Class 3] | 0.7 | 0.0 |
| PA14_49740 | PA14_49740 | hypothetical protein | Hypothetical, unclassified, unknown | Unknown [Class 3] | 2.0 | 0.0 |
| PA14_45830 | PA14_45830 | hypothetical protein | Motility & Attachment | Unknown [Class 3] | 1.8 | 0.0 |
| PA14_06200 | PA14_06200 | hypothetical protein | Biosynthesis of cofactors, prosthetic groups and carriers | Unknown [Class 3] | 1.8 | 0.0 |
| PA14_13140 | PA14_13140 | hypothetical protein | Hypothetical, unclassified, unknown | Unknown [Class 3] | 1.7 | 0.0 |
| PA14_18250 | fruI | phosphotransferase system transporter enzyme I, FruI | Transport of small molecules | Unknown [Class 3] | 1.6 | 0.0 |
| PA14_31170 | PA14_31170 | hypothetical protein | Hypothetical, unclassified, unknown | Unknown [Class 3] | 1.5 | 0.0 |
| PA14_31440 | PA14_31440 | hypothetical protein | Hypothetical, unclassified, unknown | Unknown [Class 3] | 1.5 | 0.0 |
| PA14_35790 | PA14_35790 | Putative homospermidine synthase | Putative enzymes | Unknown [Class 3] | 1.5 | 0.0 |
| PA14_53860 | PA14_53860 | hypothetical protein | Hypothetical, unclassified, unknown | Unknown [Class 3] | 1.4 | 0.0 |
| PA14_40690 | PA14_40690 | hypothetical protein | Hypothetical, unclassified, unknown | Unknown [Class 3] | 1.2 | 0.0 |
| PA14_51950 | PA14_51950 | hypothetical protein | Hypothetical, unclassified, unknown | Unknown [Class 3] | 1.2 | 0.0 |
| PA14_37980 | PA14_37980 | putative Fe2+-dicitrate sensor, membrane component | Transport of small molecules | Unknown [Class 3] | 1.1 | 0.0 |
| PA14_62270 | phuW | hypothetical protein | Hypothetical, unclassified, unknown | Unknown [Class 3] | 1.0 | 0.0 |
| PA14_59960 | PA14_59960 | Putative protein-disulfide isomerase | Translation, post-translational modification, degradation | Unknown [Class 3] | 1.0 | 0.0 |
| PA14_30860 | trbG | TrbG-like protein | Related to phage, transposon, or plasmid | Unknown [Class 3] | 1.0 | 0.0 |
| PA14_15420 | PA14_15420 | hypothetical protein | Hypothetical, unclassified, unknown | Unknown [Class 3] | 1.0 | 0.0 |
| PA14_01560 | PA14_01560 | hypothetical protein | Transport of small molecules | Unknown [Class 3] | 1.0 | 0.0 |
| PA14_01220 | PA14_01220 | Hypothetical protein | Hypothetical, unclassified, unknown | Unknown [Class 3] | 1.0 | 0.0 |
| PA14_32820 | PA14_32820 | hypothetical protein | Hypothetical, unclassified, unknown | Unknown [Class 3] | 1.0 | 0.0 |
| PA14_24245 | PA14_24245 | hypothetical protein | Hypothetical, unclassified, unknown | Unknown [Class 3] | 0.9 | 0.0 |
| PA14_58040 | PA14_58040 | hypothetical protein | Hypothetical, unclassified, unknown | Unknown [Class 3] | 0.9 | 0.0 |
| PA14_51670 | PA14_51670 | hypothetical protein | Hypothetical, unclassified, unknown | Unknown [Class 3] | 0.9 | 0.0 |
| PA14_56930 | PA14_56930 | hypothetical protein | Hypothetical, unclassified, unknown | Unknown [Class 3] | 0.9 | 0.0 |
| PA14_24150 | PA14_24150 | hypothetical protein | Hypothetical, unclassified, unknown | Unknown [Class 3] | 0.9 | 0.0 |
| PA14_55140 | PA14_55140 | hypothetical protein | Hypothetical, unclassified, unknown | Unknown [Class 3] | 0.9 | 0.0 |
| PA14_39620 | PA14_39620 | hypothetical protein | Hypothetical, unclassified, unknown | Unknown [Class 3] | 0.9 | 0.0 |
| PA14_39420 | PA14_39420 | hypothetical protein | Hypothetical, unclassified, unknown | Unknown [Class 3] | 0.8 | 0.0 |
| PA14_08320 | PA14_08320 | hypothetical protein | Hypothetical, unclassified, unknown | Unknown [Class 3] | 0.8 | 0.0 |
| PA14_22260 | PA14_22260 | hypothetical protein | Hypothetical, unclassified, unknown | Unknown [Class 3] | 0.8 | 0.0 |
| PA14_44080 | PA14_44080 | hypothetical protein | Hypothetical, unclassified, unknown | Unknown [Class 3] | 0.8 | 0.0 |
| PA14_22880 | PA14_22880 | hypothetical protein | Hypothetical, unclassified, unknown | Unknown [Class 3] | 0.7 | 0.0 |
| PA14_67410 | PA14_67410 | hypothetical protein | Hypothetical, unclassified, unknown | Unknown [Class 3] | 0.7 | 0.0 |
| PA14_67180 | PA14_67180 | hypothetical protein | Hypothetical, unclassified, unknown | Unknown [Class 3] | 0.7 | 0.0 |
| PA14_28790 | PA14_28790 | hypothetical protein | Hypothetical, unclassified, unknown | Unknown [Class 3] | 0.6 | 0.0 |
| PA14_32640 | PA14_32640 | hypothetical protein | Hypothetical, unclassified, unknown | Unknown [Class 3] | 0.6 | 0.0 |
| PA14_38420 | PA14_38420 | hypothetical protein | Hypothetical, unclassified, unknown | Unknown [Class 3] | 0.6 | 0.0 |
| PA14_55930 | PA14_55930 | hypothetical protein | Protein secretion/export apparatus | Unknown [Class 3] | 0.6 | 0.0 |
| PA14_20060 | PA14_20060 | hypothetical protein | Hypothetical, unclassified, unknown | Unknown [Class 3] | 0.6 | 0.0 |
| PA14_28940 | PA14_28940 | hypothetical protein | Hypothetical, unclassified, unknown | Unknown [Class 3] | 0.5 | 0.0 |
| PA14_62640 | PA14_62640 | hypothetical protein | Hypothetical, unclassified, unknown | Unknown [Class 3] | 0.5 | 0.0 |
| PA14_50750 | PA14_50750 | hypothetical protein | Hypothetical, unclassified, unknown | Unknown [Class 3] | 0.5 | 0.0 |
| PA14_48150 | PA14_48150 | hypothetical protein | Hypothetical, unclassified, unknown | Unknown [Class 3] | 0.5 | 0.0 |
