# Supplementary material for: A Comprehensive Analysis of In Vitro and In Vivo Genetic Fitness of Pseudomonas aeruginosa Using High-Throughput Sequencing of Transposon Libraries
Source: PLoS Pathog. 2013 Sep 5;9(9):e1003582. doi: 10.1371/journal.ppat.1003582 (PMC3764216; doi:10.1371/journal.ppat.1003582)
Supplement: Table S4 — Tn-insertionss in genes in P. aeruginosa strain PA14 with a positive phenotype for colonization. (DOC) [file ppat.1003582.s015.doc]

| Table S4: Tn-inserts in genes in *P. aeruginosa* strain PA14 with a positive phenotype for colonization | | | |  |  |
| --- | --- | --- | --- | --- | --- |
| ID | Gene name | Product Name | Functional Class | Subcellular Localization | Ratio of Cecum:LB |
| PA14_30050 | aceA | isocitrate lyase | Energy metabolism | Cytoplasmic [Class 3] | 6.8 |
| PA14_69470 | algR* | alginate biosynthesis regulatory protein AlgR | Two-component regulatory systems | Cytoplasmic [Class 3] | 82.0 |
| PA14_69480 | algZ* | alginate biosynthesis protein AlgZ/FimS | Secreted Factors (toxins, enzymes, alginate) | Cytoplasmic Membrane [Class 3] | 19.5 |
| PA14_52720 | argD | bifunctional N-succinyldiaminopimelate-aminotransferase/acetylornithine transaminase protein | Amino acid biosynthesis and metabolism | Cytoplasmic [Class 3] | 30.0 |
| PA14_03020 | aroE | shikimate 5-dehydrogenase | Amino acid biosynthesis and metabolism | Cytoplasmic [Class 3] | 2.7 |
| PA14_16660 | cadA | putative metal-transporting P-type ATPase | Transport of small molecules | Cytoplasmic Membrane [Class 3] | 2.2 |
| PA14_45610 | cheZ | chemotaxis protein CheZ | Chemotaxis | Cytoplasmic [Class 3] | 3.2 |
| PA14_05390 | chpA | ChpA | Chemotaxis | Cytoplasmic [Class 3] | 51.1 |
| PA14_26140 | cifR | CifR | Transcriptional regulators | Cytoplasmic [Class 3] | 2.2 |
| PA14_60280 | fimU | type 4 fimbrial biogenesis protein FimU | Motility & Attachment | Unknown [Class 3] | 15.2 |
| PA14_50220 | fleQ | transcriptional regulator FleQ | Transcriptional regulators | Cytoplasmic [Class 3] | 11.4 |
| PA14_50450 | flgE | flagellar hook protein FlgE | Motility & Attachment | Extracellular [Class 3] | 4.8 |
| PA14_50440 | flgF | flagellar basal body rod protein FlgF | Motility & Attachment | Periplasmic [Class 3] | 2.3 |
| PA14_50420 | flgH | flagellar basal body L-ring protein | Motility & Attachment | Flagellar [Class 3] ; Outer Membrane [Class 3] | 5.1 |
| PA14_50410 | flgI | flagellar basal body P-ring protein | Motility & Attachment | Periplasmic [Class 3] | 2.8 |
| PA14_50380 | flgJ | flagellar rod assembly protein/muramidase FlgJ | Motility & Attachment | Unknown [Class 3] | 3.4 |
| PA14_45680 | flhA | flagellar biosynthesis protein FlhA | Motility & Attachment | Flagellar [Class 3] ; Cytoplasmic Membrane [Class 3] | 7.0 |
| PA14_45720 | flhB | flagellar biosynthesis protein FlhB | Motility & Attachment | Flagellar [Class 3] ; Cytoplasmic Membrane [Class 3] | 2.6 |
| PA14_45630 | fliA | flagellar biosynthesis sigma factor | Transcriptional regulators | Cytoplasmic [Class 3] | 10.2 |
| PA14_50140 | fliF | flagellar MS-ring protein | Motility & Attachment | Cytoplasmic Membrane [Class 3] | 2.7 |
| PA14_50100 | fliI | flagellum-specific ATP synthase | Motility & Attachment | Cytoplasmic [Class 3] | 4.9 |
| PA14_45800 | fliM | flagellar motor switch protein FliM | Motility & Attachment | Flagellar [Class 3] ; Cytoplasmic Membrane [Class 3] | 6.5 |
| PA14_45740 | fliR | flagellar biosynthesis protein FliR | Motility & Attachment | Flagellar [Class 3] ; Cytoplasmic Membrane [Class 3] | 15.9 |
| PA14_36630 | glgX | putative glycosyl hydrolase | Carbon compound catabolism | Cytoplasmic [Class 3] | 4.0 |
| PA14_17220 | lpxB | lipid-A-disaccharide synthase | Cell wall / LPS / capsule | Cytoplasmic [Class 3] | 158.4 |
| PA14_02560 | mdcB | triphosphoribosyl-dephospho-CoA synthase | Biosynthesis of cofactors, prosthetic groups and carriers | Unknown [Class 3] | 3.3 |
| PA14_11845 | mpl | murein tripeptide ligase | Cell wall / LPS / capsule | Cytoplasmic [Class 3] | 2.1 |
| PA14_51340 | mvfR | Transcriptional regulator MvfR | Transcriptional regulators | Cytoplasmic [Class 3] | 6.5 |
| PA14_06770 | nirQ | regulatory protein NirQ | Energy metabolism | Cytoplasmic [Class 3] | 2.5 |
| PA14_10440 | opdL | putative porin | Transport of small molecules | Outer Membrane [Class 3] | 8.8 |
| PA14_51880 | oprD | Basic amino acid, basic peptide and imipenem outer membrane porin OprD precursor | Transport of small molecules | Outer Membrane [Class 3] | 816.8 |
| PA14_00970 | PA14_00970 | hypothetical protein | Hypothetical, unclassified, unknown | Cytoplasmic [Class 3] | 2.9 |
| PA14_01110 | PA14_01110 | Hypothetical protein | Hypothetical, unclassified, unknown | Cytoplasmic [Class 3] | 2.7 |
| PA14_01470 | PA14_01470 | putative transcriptional regulator | Transcriptional regulators | Cytoplasmic [Class 3] | 2.1 |
| PA14_02460 | PA14_02460 | putative NAD(P) transhydrogenase, subunit alpha part 2 | Energy metabolism | Cytoplasmic Membrane [Class 3] | 2.9 |
| PA14_05250 | PA14_05250 | dihydroorotase | Energy metabolism | Cytoplasmic [Class 3] | 15.8 |
| PA14_05860 | PA14_05860 | hypothetical protein | Fatty acid and phospholipid metabolism | Cytoplasmic [Class 3] | 2.5 |
| PA14_10530 | PA14_10530 | GntR family transcriptional regulator | Transcriptional regulators | Cytoplasmic [Class 3] | 6.7 |
| PA14_12550 | PA14_12550 | hypothetical protein | Hypothetical, unclassified, unknown | Unknown [Class 3] | 4.2 |
| PA14_14430 | PA14_14430 | hypothetical protein | Hypothetical, unclassified, unknown | Unknown [Class 3] | 2.8 |
| PA14_16110 | PA14_16110 | hypothetical protein | Hypothetical, unclassified, unknown | Unknown [Class 3] | 2.1 |
| PA14_20110 | PA14_20110 | hypothetical protein | Antibiotic resistance and susceptibility | Cytoplasmic Membrane [Class 3] | 5.2 |
| PA14_26730 | PA14_26730 | putative short-chain dehydrogenase | Putative enzymes | Cytoplasmic [Class 3] | 3.7 |
| PA14_27800 | PA14_27800 | putative two-component sensor | Two-component regulatory systems | Cytoplasmic Membrane [Class 3] | 3.5 |
| PA14_31930 | PA14_31930 | hypothetical protein | Hypothetical, unclassified, unknown | Cytoplasmic Membrane [Class 3] | 2.0 |
| PA14_32370 | PA14_32370 | putative DNA damage-inducible gene | DNA replication, recombination, modification and repair | Cytoplasmic [Class 3] | 2.3 |
| PA14_37760 | PA14_37760 | putative MFS transporter | Transport of small molecules | Cytoplasmic Membrane [Class 3] | 4.8 |
| PA14_44230 | PA14_44230 | hypothetical protein | Hypothetical, unclassified, unknown | Unknown [Class 3] | 4.6 |
| PA14_45890 | PA14_45890 | putative RND efflux transporter | Antibiotic resistance and susceptibility | Cytoplasmic Membrane [Class 3] | 2.3 |
| PA14_46540 | PA14_46540 | hypothetical protein | Hypothetical, unclassified, unknown | Cytoplasmic [Class 3] | 3.9 |
| PA14_47130 | PA14_47130 | hypothetical protein | Hypothetical, unclassified, unknown | Unknown [Class 3] | 4.8 |
| PA14_50510 | PA14_50510 | hypothetical protein | Hypothetical, unclassified, unknown | Unknown [Class 3] | 11.8 |
| PA14_51910 | PA14_51910 | hypothetical protein | Hypothetical, unclassified, unknown | Unknown [Class 3] | 4.7 |
| PA14_52520 | PA14_52520 | hypothetical protein | Hypothetical, unclassified, unknown | Cytoplasmic Membrane [Class 3] | 63.7 |
| PA14_53820 | PA14_53820 | hypothetical protein | Hypothetical, unclassified, unknown | Cytoplasmic [Class 3] | 10.5 |
| PA14_54120 | PA14_54120 | acyl carrier protein phosphodiesterase | Putative enzymes | Cytoplasmic Membrane [Class 3] | 3.2 |
| PA14_56960 | PA14_56960 | hypothetical protein | Hypothetical, unclassified, unknown | Cytoplasmic Membrane [Class 3] | 2.2 |
| PA14_58650 | PA14_58650 | putative chemotaxis transducer | Chemotaxis | Cytoplasmic Membrane [Class 3] | 12.2 |
| PA14_58740 | PA14_58740 | hypothetical protein | Hypothetical, unclassified, unknown | Unknown [Class 3] | 2.2 |
| PA14_60240 | PA14_60240 | hypothetical protein | Hypothetical, unclassified, unknown | Cytoplasmic Membrane [Class 3] | 2.3 |
| PA14_66160 | PA14_66160 | putative glycosyl transferase | Cell wall / LPS / capsule | Cytoplasmic [Class 3] | 119.6 |
| PA14_69270 | PA14_69270 | hypothetical protein | Biosynthesis of cofactors, prosthetic groups and carriers | Cytoplasmic [Class 3] | 9.6 |
| PA14_69770 | PA14_69770 | hypothetical protein | Hypothetical, unclassified, unknown | Unknown [Class 3] | 2.9 |
| PA14_54290 | pdxJ | pyridoxine 5'-phosphate synthase | Biosynthesis of cofactors, prosthetic groups and carriers | Cytoplasmic [Class 3] | 22.3 |
| PA14_58730 | pilA | type IV pilin structural subunit | Motility & Attachment | Fimbrial [Class 3] ; Extracellular [Class 3] | 99.8 |
| PA14_58750 | pilB | type 4 fimbrial biogenesis protein PilB | Motility & Attachment | Cytoplasmic [Class 3] | 18.3 |
| PA14_58760 | pilC | type 4 fimbrial biogenesis protein pilC | Motility & Attachment | Cytoplasmic Membrane [Class 3] | 13.8 |
| PA14_60320 | pilE | type 4 fimbrial biogenesis protein PilE | Motility & Attachment | Fimbrial [Class 3] ; Extracellular [Class 3] | 11.7 |
| PA14_05340 | pilI | twitching motility protein PilI | Chemotaxis | Cytoplasmic [Class 3] | 354.5 |
| PA14_05360 | pilJ | twitching motility protein PilJ | Chemotaxis | Outer Membrane [Class 3] | 51.5 |
| PA14_66660 | pilM | type 4 fimbrial biogenesis protein PilM | Motility & Attachment | Cytoplasmic Membrane [Class 3] | 9.4 |
| PA14_66650 | pilN | type 4 fimbrial biogenesis protein PilN | Motility & Attachment | Cytoplasmic Membrane [Class 3] | 10.9 |
| PA14_66640 | pilO | type 4 fimbrial biogenesis protein PilO | Motility & Attachment | Cytoplasmic Membrane [Class 3] | 15.2 |
| PA14_66630 | pilP | type 4 fimbrial biogenesis protein PilP | Motility & Attachment | Cytoplasmic Membrane [Class 3] | 57.4 |
| PA14_66620 | pilQ | type 4 fimbrial biogenesis outer membrane protein PilQ precursor | Motility & Attachment | Outer Membrane [Class 3] | 47.7 |
| PA14_60260 | pilR | two-component response regulator PilR | Two-component regulatory systems | Cytoplasmic [Class 3] | 25.0 |
| PA14_60250 | pilS | two-component sensor PilS | Two-component regulatory systems | Cytoplasmic Membrane [Class 3] | 27.6 |
| PA14_60290 | pilW | type 4 fimbrial biogenesis protein PilW | Motility & Attachment | Unknown [Class 3] | 12.6 |
| PA14_60300 | pilX | type 4 fimbrial biogenesis protein PilX | Motility & Attachment | Unknown [Class 3] | 14.4 |
| PA14_60310 | pilY1 | type 4 fimbrial biogenesis protein PilY1 | Motility & Attachment | Outer Membrane [Class 3] | 14.7 |
| PA14_25770 | pilZ | type 4 fimbrial biogenesis protein PilZ | Motility & Attachment | Unknown [Class 3] | 8.5 |
| PA14_58560 | piuB | oxidoreductase | Putative enzymes | Cytoplasmic Membrane [Class 3] | 5.0 |
| PA14_38800 | pqqC | pyrroloquinoline quinone biosynthesis protein PqqC | Biosynthesis of cofactors, prosthetic groups and carriers | Cytoplasmic [Class 3] | 4.5 |
| PA14_42350 | pscC | Type III secretion outer membrane protein PscC precursor | Protein secretion/export apparatus | Outer Membrane [Class 3] | 2.5 |
| PA14_60450 | rpmA | 50S ribosomal protein L27 | Translation, post-translational modification, degradation | Cytoplasmic [Class 3] | 5.6 |
| PA14_57940 | rpoN* | RNA polymerase factor sigma-54 | Transcriptional regulators | Cytoplasmic [Class 3] | 42.6 |
| PA14_64790 | vanK | putative MFS transporter | Transport of small molecules | Cytoplasmic Membrane [Class 3] | 7.3 |
| PA14_61860 | yadF | putative carbonic anhydrase | Carbon compound catabolism | Cytoplasmic [Class 3] | 11.9 |
| PA14_52140 | yaiL | hypothetical protein | Hypothetical, unclassified, unknown | Cytoplasmic [Class 3] | 21.8 |
|  | *: Regulator of the biogenesis of the type IVa pili | |  |  |  |
