# Supplementary material for: A Comprehensive Analysis of In Vitro and In Vivo Genetic Fitness of Pseudomonas aeruginosa Using High-Throughput Sequencing of Transposon Libraries
Source: PLoS Pathog. 2013 Sep 5;9(9):e1003582. doi: 10.1371/journal.ppat.1003582 (PMC3764216; doi:10.1371/journal.ppat.1003582)
Supplement: Table S5 — Tn-insertions into genes in P. aeruginosa strain PA14 unable to colonize the murine GI tract. (DOC) [file ppat.1003582.s016.doc]

| Table S5: Tn-insertions into genes in P. aeruginosa strain PA14 unable to colonize the murine GI tract | | | | |
| --- | --- | --- | --- | --- |
| ID | Gene name | Product Name | Function Class | Subcellular Localization |
| PA14_35100 | arsC | arsenate reductase | Adaptation, Protection | Cytoplasmic [Class 3] |
| PA14_35080 | PA14_49410 | cold-shock protein | Adaptation, Protection | Cytoplasmic [Class 3] |
| PA14_15445 | PA14_12300 | hypothetical protein | Adaptation, Protection | Cytoplasmic Membrane [Class 3] |
| PA14_15475 | PA14_12330 | hypothetical protein | Adaptation, Protection | Cytoplasmic [Class 3] |
| PA14_00710 | PA14_25040 | hypothetical protein | Adaptation, Protection | Unknown [Class 3] |
| PA14_12300 | PA14_27450 | hypothetical protein | Adaptation, Protection | Cytoplasmic [Class 3] |
| PA14_12330 | PA14_48750 | hypothetical protein | Adaptation, Protection | Cytoplasmic [Class 3] |
| PA14_25040 | PA14_63360 | hypothetical protein | Adaptation, Protection | Cytoplasmic [Class 3] |
| PA14_27450 | PA14_66090 | hypothetical protein | Adaptation, Protection | Cytoplasmic [Class 3] |
| PA14_48750 | osmC | osmotically inducible protein OsmC | Adaptation, Protection | Cytoplasmic [Class 3] |
| PA14_49410 | arsH | putative arsenical resistance protein | Adaptation, Protection | Cytoplasmic [Class 3] |
| PA14_63360 | PA14_68670 | putative carboxypeptidase | Adaptation, Protection | Cytoplasmic [Class 3] |
| PA14_66090 | merT | putative mercuric transport protein | Adaptation, Protection | Cytoplasmic [Class 3] |
| PA14_68670 | merE | putative mercury resistance protein | Adaptation, Protection | Cytoplasmic [Class 3] |
| PA14_33270 | pvdG | PvdG | Adaptation, Protection | Cytoplasmic [Class 3] |
| PA14_57530 | sspA | stringent starvation protein A | Adaptation, Protection | Cytoplasmic [Class 3] |
| PA14_31810 | tpx | thiol peroxidase | Adaptation, Protection | Periplasmic [Class 3] |
| PA14_68330 | aroQ1 | 3-dehydroquinate dehydratase | Amino acid biosynthesis and metabolism | Cytoplasmic [Class 3] |
| PA14_68350 | aroB | 3-dehydroquinate synthase | Amino acid biosynthesis and metabolism | Cytoplasmic [Class 3] |
| PA14_66600 | PA14_16960 | amino acid transporter LysE | Amino acid biosynthesis and metabolism | Cytoplasmic Membrane [Class 3] |
| PA14_42760 | trpD | anthranilate phosphoribosyltransferase | Amino acid biosynthesis and metabolism | Cytoplasmic [Class 3] |
| PA14_00290 | arcA | arginine deiminase | Amino acid biosynthesis and metabolism | Cytoplasmic [Class 3] |
| PA14_41920 | arcC | carbamate kinase | Amino acid biosynthesis and metabolism | Cytoplasmic [Class 3] |
| PA14_66610 | PA14_36370 | carboxylate-amine ligase | Amino acid biosynthesis and metabolism | Cytoplasmic [Class 3] |
| PA14_64090 | aroC | chorismate synthase | Amino acid biosynthesis and metabolism | Cytoplasmic [Class 3] |
| PA14_51270 | iscS | cysteine desulfurase | Amino acid biosynthesis and metabolism | Cytoplasmic [Class 3] |
| PA14_62940 | dapF | diaminopimelate epimerase | Amino acid biosynthesis and metabolism | Cytoplasmic [Class 3] |
| PA14_16950 | dapB | dihydrodipicolinate reductase | Amino acid biosynthesis and metabolism | Cytoplasmic [Class 3] |
| PA14_49380 | glnS | glutaminyl-tRNA synthetase | Amino acid biosynthesis and metabolism | Cytoplasmic [Class 3] |
| PA14_69690 | gcvT | glycine cleavage system aminomethyltransferase T | Amino acid biosynthesis and metabolism | Cytoplasmic [Class 3] |
| PA14_68860 | gcvH1 | glycine cleavage system protein H | Amino acid biosynthesis and metabolism | Unknown [Class 3] |
| PA14_68870 | obgE | GTPase ObgE | Amino acid biosynthesis and metabolism | Cytoplasmic [Class 3] |
| PA14_41380 | hisC2 | histidinol-phosphate aminotransferase | Amino acid biosynthesis and metabolism | Cytoplasmic [Class 3] |
| PA14_23290 | ilvG | hypothetical protein | Amino acid biosynthesis and metabolism | Cytoplasmic [Class 3] |
| PA14_46120 | PA14_12730 | hypothetical protein | Amino acid biosynthesis and metabolism | Periplasmic [Class 3] |
| PA14_14730 | PA14_15910 | hypothetical protein | Amino acid biosynthesis and metabolism | Unknown [Class 3] |
| PA14_37830 | PA14_47840 | hypothetical protein | Amino acid biosynthesis and metabolism | Cytoplasmic [Class 3] |
| PA14_37590 | PA14_63120 | hypothetical protein | Amino acid biosynthesis and metabolism | Cytoplasmic [Class 3] |
| PA14_60445 | PA14_63370 | hypothetical protein | Amino acid biosynthesis and metabolism | Unknown [Class 3] |
| PA14_12730 | PA14_65050 | hypothetical protein | Amino acid biosynthesis and metabolism | Cytoplasmic [Class 3] |
| PA14_15910 | kynB | kynurenine formamidase | Amino acid biosynthesis and metabolism | Cytoplasmic [Class 3] |
| PA14_16960 | PA14_64850 | ornithine cyclodeaminase | Amino acid biosynthesis and metabolism | Cytoplasmic [Class 3] |
| PA14_36370 | aroF-1 | phospho-2-dehydro-3-deoxyheptonate aldolase | Amino acid biosynthesis and metabolism | Cytoplasmic [Class 3] |
| PA14_47840 | ptpA | phosphotyrosine protein phosphatase | Amino acid biosynthesis and metabolism | Cytoplasmic [Class 3] |
| PA14_48570 | yciV | PHP domain-containing protein | Amino acid biosynthesis and metabolism | Cytoplasmic [Class 3] |
| PA14_63120 | PA14_48570 | putative 2-isopropylmalate synthase | Amino acid biosynthesis and metabolism | Cytoplasmic [Class 3] |
| PA14_63370 | dapA | putative dihydrodipicolinate synthase | Amino acid biosynthesis and metabolism | Cytoplasmic [Class 3] |
| PA14_64850 | PA14_70100 | putative oxidoreductase | Amino acid biosynthesis and metabolism | Cytoplasmic [Class 3] |
| PA14_65050 | iscS | putative pyridoxal-phosphate dependent enzyme | Amino acid biosynthesis and metabolism | Cytoplasmic [Class 3] |
| PA14_70100 | proC | pyrroline-5-carboxylate reductase | Amino acid biosynthesis and metabolism | Cytoplasmic [Class 3] |
| PA14_05150 | aroE | shikimate 5-dehydrogenase | Amino acid biosynthesis and metabolism | Cytoplasmic [Class 3] |
| PA14_25540 | aroK | shikimate kinase | Amino acid biosynthesis and metabolism | Cytoplasmic [Class 3] |
| PA14_16090 | dapE | succinyl-diaminopimelate desuccinylase | Amino acid biosynthesis and metabolism | Cytoplasmic [Class 3] |
| PA14_08350 | dapD | tetrahydrodipicolinate succinylase | Amino acid biosynthesis and metabolism | Unknown [Class 3] |
| PA14_22820 | thrC | Threonine synthase | Amino acid biosynthesis and metabolism | Cytoplasmic [Class 3] |
| PA14_10790 | ampC | beta-lactamase | Antibiotic resistance and susceptibility | Periplasmic [Class 3] |
| PA14_39190 | PA14_18180 | hypothetical protein | Antibiotic resistance and susceptibility | Unknown [Class 3] |
| PA14_18180 | PA14_20250 | hypothetical protein | Antibiotic resistance and susceptibility | Unknown [Class 3] |
| PA14_20250 | PA14_23670 | hypothetical protein | Antibiotic resistance and susceptibility | Unknown [Class 3] |
| PA14_23670 | PA14_36280 | putative antibiotic biosynthesis monooxygenase | Antibiotic resistance and susceptibility | Cytoplasmic [Class 3] |
| PA14_29830 | PA14_29830 | putative methyltransferase | Antibiotic resistance and susceptibility | Cytoplasmic [Class 3] |
| PA14_36280 | PA14_38020 | putative ntibiotic biosynthesis monooxygenase | Antibiotic resistance and susceptibility | Unknown [Class 3] |
| PA14_38020 | PA14_68110 | putative transcriptional regulator | Antibiotic resistance and susceptibility | Unknown [Class 3] |
| PA14_68110 | str | streptomycin 3''-phosphotransferase | Antibiotic resistance and susceptibility | Cytoplasmic [Class 3] |
| PA14_40450 | bacA | undecaprenyl pyrophosphate phosphatase | Antibiotic resistance and susceptibility | Cytoplasmic Membrane [Class 3] |
| PA14_06540 | pabC | 4-amino-4-deoxychorismate lyase | Biosynthesis of cofactors, prosthetic groups and carriers | Cytoplasmic [Class 3] |
| PA14_06570 | ipk | 4-diphosphocytidyl-2-C-methyl-D-erythritol kinase | Biosynthesis of cofactors, prosthetic groups and carriers | Unknown [Class 3] |
| PA14_06510 | ubiA | 4-hydroxybenzoate octaprenyltransferase | Biosynthesis of cofactors, prosthetic groups and carriers | Cytoplasmic Membrane [Class 3] |
| PA14_58780 | ribH | 6,7-dimethyl-8-ribityllumazine synthase | Biosynthesis of cofactors, prosthetic groups and carriers | Cytoplasmic [Class 3] |
| PA14_49820 | bioF | 8-amino-7-oxononanoate synthase | Biosynthesis of cofactors, prosthetic groups and carriers | Cytoplasmic [Class 3] |
| PA14_47760 | cobU | adenosylcobinamide kinase/adenosylcobinamide-phosphate guanylyltransferase | Biosynthesis of cofactors, prosthetic groups and carriers | Cytoplasmic [Class 3] |
| PA14_26460 | cobO | cob(I)yrinic acid a,c-diamide adenosyltransferase | Biosynthesis of cofactors, prosthetic groups and carriers | Cytoplasmic [Class 3] |
| PA14_25920 | cobB | cobalamin biosynthetic protein | Biosynthesis of cofactors, prosthetic groups and carriers | Cytoplasmic [Class 3] |
| PA14_47790 | cobB | cobalamin biosynthetic protein | Biosynthesis of cofactors, prosthetic groups and carriers | Cytoplasmic [Class 3] |
| PA14_47650 | cobS | cobalamin synthase | Biosynthesis of cofactors, prosthetic groups and carriers | Cytoplasmic Membrane [Class 3] |
| PA14_47670 | cobK | cobalt-precorrin-6x reductase | Biosynthesis of cofactors, prosthetic groups and carriers | Cytoplasmic [Class 3] |
| PA14_47680 | pqqA | coenzyme PQQ synthesis protein PqqA | Biosynthesis of cofactors, prosthetic groups and carriers | Unknown [Class 3] |
| PA14_07170 | pvdN | cysteine desulfurase activity | Biosynthesis of cofactors, prosthetic groups and carriers | Unknown [Class 3] |
| PA14_07590 | coaE | Dephospho-CoA kinase | Biosynthesis of cofactors, prosthetic groups and carriers | Cytoplasmic [Class 3] |
| PA14_42850 | epd | D-erythrose 4-phosphate dehydrogenase | Biosynthesis of cofactors, prosthetic groups and carriers | Cytoplasmic [Class 3] |
| PA14_62570 | folB | dihydroneopterin aldolase | Biosynthesis of cofactors, prosthetic groups and carriers | Cytoplasmic [Class 3] |
| PA14_69420 | bioD | dithiobiotin synthetase | Biosynthesis of cofactors, prosthetic groups and carriers | Cytoplasmic [Class 3] |
| PA14_61750 | folE | GTP cyclohydrolase I | Biosynthesis of cofactors, prosthetic groups and carriers | Cytoplasmic [Class 3] |
| PA14_12120 | nemO | heme oxygenase | Biosynthesis of cofactors, prosthetic groups and carriers | Unknown [Class 3] |
| PA14_12130 | PA14_25930 | hypothetical protein | Biosynthesis of cofactors, prosthetic groups and carriers | Unknown [Class 3] |
| PA14_44970 | PA14_55710 | hypothetical protein | Biosynthesis of cofactors, prosthetic groups and carriers | Unknown [Class 3] |
| PA14_13240 | PA14_60920 | hypothetical protein | Biosynthesis of cofactors, prosthetic groups and carriers | Cytoplasmic [Class 3] |
| PA14_13250 | PA14_64940 | hypothetical protein | Biosynthesis of cofactors, prosthetic groups and carriers | Cytoplasmic [Class 3] |
| PA14_61670 | lipB | lipoate-protein ligase B | Biosynthesis of cofactors, prosthetic groups and carriers | Cytoplasmic [Class 3] |
| PA14_12020 | lis | lipoyl synthase | Biosynthesis of cofactors, prosthetic groups and carriers | Cytoplasmic [Class 3] |
| PA14_55580 | moaA2 | molybdenum cofactor biosynthesis protein A | Biosynthesis of cofactors, prosthetic groups and carriers | Cytoplasmic [Class 3] |
| PA14_01430 | moeB | molybdopterin biosynthesis protein MoeB | Biosynthesis of cofactors, prosthetic groups and carriers | Cytoplasmic [Class 3] |
| PA14_13680 | moaE | molybdopterin converting factor, large subunit | Biosynthesis of cofactors, prosthetic groups and carriers | Cytoplasmic [Class 3] |
| PA14_13690 | moaD | molybdopterin converting factor, small subunit | Biosynthesis of cofactors, prosthetic groups and carriers | Unknown [Class 3] |
| PA14_25930 | cobT | nicotinate-nucleotide--dimethylbenzimidazole phosphoribosyltransferase | Biosynthesis of cofactors, prosthetic groups and carriers | Cytoplasmic [Class 3] |
| PA14_25990 | nadD | nicotinic acid mononucleotide adenylyltransferase | Biosynthesis of cofactors, prosthetic groups and carriers | Cytoplasmic [Class 3] |
| PA14_26000 | cobM | precorrin-3 methylase | Biosynthesis of cofactors, prosthetic groups and carriers | Cytoplasmic [Class 3] |
| PA14_31510 | folk | putative 2-amino-4-hydroxy-6-hydroxymethyldihydropteridine pyrophosphokinase | Biosynthesis of cofactors, prosthetic groups and carriers | Cytoplasmic [Class 3] |
| PA14_55710 | bioC | putative biotin synthesis protein BioC | Biosynthesis of cofactors, prosthetic groups and carriers | Unknown [Class 3] |
| PA14_60920 | hemY | putative enzyme of heme biosynthesis | Biosynthesis of cofactors, prosthetic groups and carriers | Cytoplasmic Membrane [Class 3] |
| PA14_61680 | PA14_25990 | putative magnesium chelatase | Biosynthesis of cofactors, prosthetic groups and carriers | Cytoplasmic [Class 3] |
| PA14_64940 | PA14_26000 | putative magnesium chelatase | Biosynthesis of cofactors, prosthetic groups and carriers | Cytoplasmic [Class 3] |
| PA14_25710 | PA14_61680 | putative methyl transferase | Biosynthesis of cofactors, prosthetic groups and carriers | Cytoplasmic [Class 3] |
| PA14_50800 | PA14_13690 | putative methyltransferase | Biosynthesis of cofactors, prosthetic groups and carriers | Cytoplasmic [Class 3] |
| PA14_38825 | PA14_01430 | putative short chain dehydrogenase | Biosynthesis of cofactors, prosthetic groups and carriers | Cytoplasmic [Class 3] |
| PA14_38820 | PA14_31510 | putative short-chain dehydrogenase | Biosynthesis of cofactors, prosthetic groups and carriers | Cytoplasmic [Class 3] |
| PA14_38790 | pdxH | pyridoxamine 5'-phosphate oxidase | Biosynthesis of cofactors, prosthetic groups and carriers | Cytoplasmic [Class 3] |
| PA14_33720 | pqqB | pyrroloquinoline quinone biosynthesis protein PqqB | Biosynthesis of cofactors, prosthetic groups and carriers | Cytoplasmic [Class 3] |
| PA14_11410 | pqqD | pyrroloquinoline quinone biosynthesis protein PqqD | Biosynthesis of cofactors, prosthetic groups and carriers | Cytoplasmic [Class 3] |
| PA14_11430 | ribC | riboflavin synthase subunit alpha | Biosynthesis of cofactors, prosthetic groups and carriers | Cytoplasmic [Class 3] |
| PA14_12400 | PA14_13680 | short chain dehydrogenase | Biosynthesis of cofactors, prosthetic groups and carriers | Cytoplasmic [Class 3] |
| PA14_11460 | thiS | sulfur carrier protein ThiS | Biosynthesis of cofactors, prosthetic groups and carriers | Unknown [Class 3] |
| PA14_04970 | thiL | thiamine monophosphate kinase | Biosynthesis of cofactors, prosthetic groups and carriers | Unknown [Class 3] |
| PA14_70730 | thiE | thiamine-phosphate pyrophosphorylase | Biosynthesis of cofactors, prosthetic groups and carriers | Unknown [Class 3] |
| PA14_66920 | ubiB | ubiquinone biosynthesis protein UbiB | Biosynthesis of cofactors, prosthetic groups and carriers | Cytoplasmic Membrane [Class 3] |
| PA14_66900 | ubiE | ubiquinone/menaquinone biosynthesis methyltransferase | Biosynthesis of cofactors, prosthetic groups and carriers | Cytoplasmic [Class 3] |
| PA14_64660 | ureF | urease accessory protein UreF | Biosynthesis of cofactors, prosthetic groups and carriers | Unknown [Class 3] |
| PA14_10270 | hpcG | 2-oxo-hept-3-ene-1,7-dioate hydratase | Carbon compound catabolism | Cytoplasmic [Class 3] |
| PA14_38590 | pcaB | 3-carboxy-cis,cis-muconate cycloisomerase | Carbon compound catabolism | Cytoplasmic [Class 3] |
| PA14_32220 | bdhA | 3-hydroxybutyrate dehydrogenase | Carbon compound catabolism | Cytoplasmic [Class 3] |
| PA14_32230 | pcaD | beta-ketoadipate enol-lactone hydrolase | Carbon compound catabolism | Unknown [Class 3] |
| PA14_36570 | gnyH | gamma-carboxygeranoyl-CoA hydratase | Carbon compound catabolism | Cytoplasmic [Class 3] |
| PA14_38470 | pcaC | gamma-carboxymuconolactone decarboxylase | Carbon compound catabolism | Unknown [Class 3] |
| PA14_10590 | glgA | glycogen synthase | Carbon compound catabolism | Cytoplasmic [Class 3] |
| PA14_10570 | acoX | hypothetical protein | Carbon compound catabolism | Unknown [Class 3] |
| PA14_02610 | PA14_12690 | hypothetical protein | Carbon compound catabolism | Unknown [Class 3] |
| PA14_02620 | PA14_71570 | hypothetical protein | Carbon compound catabolism | Cytoplasmic [Class 3] |
| PA14_34360 | yajR | major facilitator transporter | Carbon compound catabolism | Cytoplasmic [Class 3] |
| PA14_57330 | mtlD | mannitol dehydrogenase | Carbon compound catabolism | Cytoplasmic [Class 3] |
| PA14_57340 | catB | muconate cycloisomerase I | Carbon compound catabolism | Cytoplasmic [Class 3] |
| PA14_12690 | catC | muconolactone delta-isomerase | Carbon compound catabolism | Cytoplasmic [Class 3] |
| PA14_37550 | mdcG | phosphoribosyl-dephospho-CoA transferase | Carbon compound catabolism | Cytoplasmic [Class 3] |
| PA14_53790 | pcaG | protocatechuate 3,4-dioxygenase, alpha subunit | Carbon compound catabolism | Cytoplasmic [Class 3] |
| PA14_54670 | hpcH | putative 2,4-dihydroxyhept-2-ene-1,7-dioic acid aldolase | Carbon compound catabolism | Cytoplasmic [Class 3] |
| PA14_71570 | PA14_54670 | putative 3-hydroxyisobutyrate dehydrogenase | Carbon compound catabolism | Cytoplasmic [Class 3] |
| PA14_02830 | pcaJ | putative CoA transferase, subunit B | Carbon compound catabolism | Cytoplasmic [Class 3] |
| PA14_02850 | mdcH | putative epsilon subunit of malonate decarboxylase | Carbon compound catabolism | Cytoplasmic [Class 3] |
| PA14_02840 | PA14_53790 | putative haloacid dehalogenase | Carbon compound catabolism | Cytoplasmic [Class 3] |
| PA14_01910 | PA14_37550 | putative ring-hydroxylating dioxygenase small subunit | Carbon compound catabolism | Cytoplasmic [Class 3] |
| PA14_02770 | soxG | sarcosine oxidase gamma subunit | Carbon compound catabolism | Cytoplasmic [Class 3] |
| PA14_71510 | murC | UDP-N-acetylmuramate--L-alanine ligase | Carbon compound catabolism | Cytoplasmic [Class 3] |
| PA14_09195 | murG | undecaprenyldiphospho-muramoylpentapeptide beta-N- acetylglucosaminyltransferase | Carbon compound catabolism | Cytoplasmic Membrane [Class 3] |
| PA14_57290 | gidB | 16S rRNA methyltransferase GidB | Cell division | Unknown [Class 3] |
| PA14_04910 | ftsE | cell division ATP-binding protein FtsE | Cell division | Cytoplasmic Membrane [Class 3] |
| PA14_57275 | ftsA | cell division protein FtsA | Cell division | Cytoplasmic [Class 3] |
| PA14_73360 | rrmJ | cell division protein FtsJ | Cell division | Cytoplasmic [Class 3] |
| PA14_17280 | ftsZ | cell division protein FtsZ | Cell division | Cytoplasmic [Class 3] |
| PA14_22010 | zipA | cell division protein ZipA | Cell division | Cytoplasmic Membrane [Class 3] |
| PA14_17330 | minE | cell division topological specificity factor MinE | Cell division | Cytoplasmic [Class 3] |
| PA14_25150 | soj | chromosome partitioning protein Soj | Cell division | Cytoplasmic Membrane [Class 3] |
| PA14_45520 | parB | chromosome partitioning protein Spo0J | Cell division | Cytoplasmic [Class 3] |
| PA14_66760 | mesJ | hypothetical protein | Cell division | Cytoplasmic [Class 3] |
| PA14_73330 | PA14_17330 | hypothetical protein | Cell division | Unknown [Class 3] |
| PA14_62870 | PA14_25150 | hypothetical protein | Cell division | Unknown [Class 3] |
| PA14_73350 | PA14_66760 | hypothetical protein | Cell division | Cytoplasmic Membrane [Class 3] |
| PA14_44670 | PA14_45520 | putative plasmid partitioning protein | Cell division | Cytoplasmic Membrane [Class 3] |
| PA14_22050 | kdsA | 2-dehydro-3-deoxyphosphooctonate aldolase | Cell wall / LPS / capsule | Cytoplasmic [Class 3] |
| PA14_17310 | waaA | 3-deoxy-D-manno-octulosonic-acid transferase | Cell wall / LPS / capsule | Cytoplasmic [Class 3] |
| PA14_25530 | kdsB | 3-deoxy-manno-octulosonate cytidylyltransferase | Cell wall / LPS / capsule | Cytoplasmic [Class 3] |
| PA14_61740 | rhlG | beta-ketoacyl reductase | Cell wall / LPS / capsule | Cytoplasmic [Class 3] |
| PA14_69660 | rmlC | dTDP-4-dehydrorhamnose 3,5-epimerase | Cell wall / LPS / capsule | Unknown [Class 3] |
| PA14_17180 | rmlD | dTDP-4-dehydrorhamnose reductase | Cell wall / LPS / capsule | Cytoplasmic [Class 3] |
| PA14_25510 | murI | glutamate racemase | Cell wall / LPS / capsule | Cytoplasmic [Class 3] |
| PA14_57450 | waaF | heptosyltransferase II | Cell wall / LPS / capsule | Cytoplasmic [Class 3] |
| PA14_57380 | PA14_11250 | hypothetical protein | Cell wall / LPS / capsule | Cytoplasmic [Class 3] |
| PA14_58130 | wapQ | hypothetical protein | Cell wall / LPS / capsule | Cytoplasmic [Class 3] |
| PA14_58120 | htrB | lipid A biosynthesis lauroyl acyltransferase | Cell wall / LPS / capsule | Cytoplasmic Membrane [Class 3] |
| PA14_25550 | lppL | Lipopeptide LppL precursor | Cell wall / LPS / capsule | Unknown [Class 3] |
| PA14_57390 | waaP | lipopolysaccharide kinase WaaP | Cell wall / LPS / capsule | Cytoplasmic [Class 3] |
| PA14_61660 | lolB | outer membrane lipoprotein LolB | Cell wall / LPS / capsule | Unknown [Class 3] |
| PA14_63030 | omlA | outer membrane lipoprotein OmlA precursor | Cell wall / LPS / capsule | Outer Membrane [Class 3] |
| PA14_23410 | pbpA | penicillin-binding protein 2 | Cell wall / LPS / capsule | Cytoplasmic Membrane [Class 3] |
| PA14_07790 | mraY | phospho-N-acetylmuramoyl-pentapeptide- transferase | Cell wall / LPS / capsule | Cytoplasmic Membrane [Class 3] |
| PA14_11250 | orfJ | putative glycosyl transferase | Cell wall / LPS / capsule | Unknown [Class 3] |
| PA14_17150 | prmF | putative glycosyl transferase | Cell wall / LPS / capsule | Cytoplasmic Membrane [Class 3] |
| PA14_48790 | PA14_48790 | putative lipoprotein | Cell wall / LPS / capsule | Unknown [Class 3] |
| PA14_12060 | PA14_07790 | putative nucleotidyl transferase | Cell wall / LPS / capsule | Cytoplasmic [Class 3] |
| PA14_18360 | PA14_17150 | putative outer membrane antigen | Cell wall / LPS / capsule | Outer Membrane [Class 3] |
| PA14_20270 | slt | putative soluble lytic transglycosylase | Cell wall / LPS / capsule | Periplasmic [Class 3] |
| PA14_68210 | rodA | rod shape-determining protein | Cell wall / LPS / capsule | Cytoplasmic Membrane [Class 3] |
| PA14_68190 | mreC | rod shape-determining protein MreC | Cell wall / LPS / capsule | Unknown [Class 3] |
| PA14_12070 | mreD | rod shape-determining protein MreD | Cell wall / LPS / capsule | Cytoplasmic Membrane [Class 3] |
| PA14_25000 | mraW | S-adenosyl-methyltransferase MraW | Cell wall / LPS / capsule | Cytoplasmic [Class 3] |
| PA14_65960 | lpxK | tetraacyldisaccharide 4'-kinase | Cell wall / LPS / capsule | Cytoplasmic [Class 3] |
| PA14_66250 | lpxD | UDP-3-O-[3-hydroxymyristoyl] glucosamine N-acyltransferase | Cell wall / LPS / capsule | Cytoplasmic [Class 3] |
| PA14_66220 | murB | UDP-N-acetylenolpyruvoylglucosamine reductase | Cell wall / LPS / capsule | Cytoplasmic [Class 3] |
| PA14_66200 | murF | UDP-N-acetylmuramoylalanyl-D-glutamyl-2, 6-diaminopimelate--D-alanyl-D-alanyl ligase | Cell wall / LPS / capsule | Cytoplasmic [Class 3] |
| PA14_37965 | pgl | 6-phosphogluconolactonase | Central intermediary metabolism | Unknown [Class 3] |
| PA14_37950 | cynT | carbonate dehydratase | Central intermediary metabolism | Cytoplasmic [Class 3] |
| PA14_67490 | PA14_68480 | chorismate mutase | Central intermediary metabolism | Periplasmic [Class 3] |
| PA14_04580 | cynS | cyanate hydratase | Central intermediary metabolism | Cytoplasmic [Class 3] |
| PA14_70680 | pvdH | diaminobutyrate--2-oxoglutarate aminotransferase | Central intermediary metabolism | Cytoplasmic [Class 3] |
| PA14_73170 | folA | dihydrofolate reductase | Central intermediary metabolism | Unknown [Class 3] |
| PA14_55130 | fbp | fructose-1,6-bisphosphatase | Central intermediary metabolism | Cytoplasmic [Class 3] |
| PA14_36330 | glmS | glucosamine--fructose-6-phosphate aminotransferase | Central intermediary metabolism | Cytoplasmic [Class 3] |
| PA14_36320 | yliJ | glutathione S-transferase | Central intermediary metabolism | Cytoplasmic [Class 3] |
| PA14_66950 | hprA | glycerate dehydrogenase | Central intermediary metabolism | Cytoplasmic [Class 3] |
| PA14_10650 | glcE | glycolate oxidase FAD binding subunit | Central intermediary metabolism | Cytoplasmic [Class 3] |
| PA14_61210 | hcnA | hydrogen cyanide synthase HcnA | Central intermediary metabolism | Unknown [Class 3] |
| PA14_34180 | hcnB | hydrogen cyanide synthase HcnB | Central intermediary metabolism | Cytoplasmic [Class 3] |
| PA14_11260 | hpaG1 | hypothetical protein | Central intermediary metabolism | Cytoplasmic [Class 3] |
| PA14_36890 | PA14_45470 | hypothetical protein | Central intermediary metabolism | Cytoplasmic [Class 3] |
| PA14_45470 | ubiC | hypothetical protein | Central intermediary metabolism | Cytoplasmic [Class 3] |
| PA14_48020 | pchB | isochorismate-pyruvate lyase | Central intermediary metabolism | Unknown [Class 3] |
| PA14_48620 | gloA2 | lactoylglutathione lyase | Central intermediary metabolism | Cytoplasmic [Class 3] |
| PA14_64520 | msuE | NADH-dependent FMN reductase MsuE | Central intermediary metabolism | Unknown [Class 3] |
| PA14_66170 | hisE | phosphoribosyl-ATP pyrophosphatase | Central intermediary metabolism | Cytoplasmic [Class 3] |
| PA14_67260 | PA14_64520 | putative bacterioferritin | Central intermediary metabolism | Cytoplasmic [Class 3] |
| PA14_68480 | PA14_66170 | putative carbamoyl transferase | Central intermediary metabolism | Cytoplasmic [Class 3] |
| PA14_09220 | PA14_48620 | putative clavaminic acid synthetase | Central intermediary metabolism | Unknown [Class 3] |
| PA14_09290 | PA14_11260 | putative epimerase | Central intermediary metabolism | Cytoplasmic [Class 3] |
| PA14_23080 | PA14_67260 | putative histidine/phenylalanine ammonia-lyase | Central intermediary metabolism | Cytoplasmic [Class 3] |
| PA14_33500 | PA14_48020 | putative L-malate dehydrogenase | Central intermediary metabolism | Cytoplasmic [Class 3] |
| PA14_07910 | PA14_36890 | putative metallothionein | Central intermediary metabolism | Unknown [Class 3] |
| PA14_19590 | ssuF | putative molybdopterin-binding protein | Central intermediary metabolism | Unknown [Class 3] |
| PA14_62830 | pchG | pyochelin biosynthetic protein PchG | Central intermediary metabolism | Cytoplasmic [Class 3] |
| PA14_70720 | rpe | ribulose-phosphate 3-epimerase | Central intermediary metabolism | Cytoplasmic [Class 3] |
| PA14_64350 | tpiA | triosephosphate isomerase | Central intermediary metabolism | Cytoplasmic [Class 3] |
| PA14_64370 | ureD | urease accessory protein | Central intermediary metabolism | Cytoplasmic [Class 3] |
| PA14_64335 | ureB | urease subunit beta | Central intermediary metabolism | Cytoplasmic [Class 3] |
| PA14_27755 | ureA | urease subunit gamma | Central intermediary metabolism | Cytoplasmic [Class 3] |
| PA14_41230 | clpX | ATP-dependent protease ATP-binding subunit ClpX | Chaperones & heat shock proteins | Cytoplasmic [Class 3] |
| PA14_37000 | cupA5 | chaperone CupA5 | Chaperones & heat shock proteins | Periplasmic [Class 3] |
| PA14_64080 | groES | co-chaperonin GroES | Chaperones & heat shock proteins | Cytoplasmic [Class 3] |
| PA14_57020 | ppiC1 | peptidyl-prolyl cis-trans isomerase C1 | Chaperones & heat shock proteins | Cytoplasmic [Class 3] |
| PA14_38700 | dipZ | thiol:disulfide interchange protein precursor | Chaperones & heat shock proteins | Cytoplasmic Membrane [Class 3] |
| PA14_02190 | cheD | hypothetical protein | Chemotaxis | Cytoplasmic [Class 3] |
| PA14_20760 | chpC | putative chemotaxis protein | Chemotaxis | Unknown [Class 3] |
| PA14_45500 | chpE | putative chemotaxis protein | Chemotaxis | Cytoplasmic Membrane [Class 3] |
| PA14_05410 | PA14_20750 | putative chemotaxis protein | Chemotaxis | Cytoplasmic [Class 3] |
| PA14_05430 | cheR | putative chemotaxis protein methyltransferase | Chemotaxis | Cytoplasmic [Class 3] |
| PA14_20750 | PA14_39560 | putative chemotaxis transducer | Chemotaxis | Cytoplasmic Membrane [Class 3] |
| PA14_39560 | cheW | putative purine-binding chemotaxis protein | Chemotaxis | Cytoplasmic [Class 3] |
| PA14_17760 | aspP | adenosine diphosphate sugar pyrophosphatase | DNA replication, recombination, modification and repair | Cytoplasmic [Class 3] |
| PA14_65710 | dnaA | chromosomal replication initiation protein | DNA replication, recombination, modification and repair | Cytoplasmic [Class 3] |
| PA14_00010 | holB | DNA polymerase III subunit delta' | DNA replication, recombination, modification and repair | Cytoplasmic [Class 3] |
| PA14_25760 | PA14_22380 | DNA polymerase III subunit epsilon | DNA replication, recombination, modification and repair | Cytoplasmic [Class 3] |
| PA14_41210 | radC | DNA repair protein RadC | DNA replication, recombination, modification and repair | Cytoplasmic [Class 3] |
| PA14_28720 | hupB | DNA-binding protein HU | DNA replication, recombination, modification and repair | Cytoplasmic [Class 3] |
| PA14_23340 | PA14_14150 | GIY-YIG nuclease superfamily protein | DNA replication, recombination, modification and repair | Unknown [Class 3] |
| PA14_25160 | ruvA | Holliday junction DNA helicase RuvA | DNA replication, recombination, modification and repair | Cytoplasmic [Class 3] |
| PA14_40950 | ruvC | Holliday junction resolvase | DNA replication, recombination, modification and repair | Cytoplasmic [Class 3] |
| PA14_14150 | PA14_15860 | hypothetical protein | DNA replication, recombination, modification and repair | Cytoplasmic [Class 3] |
| PA14_15860 | PA14_67120 | hypothetical protein | DNA replication, recombination, modification and repair | Cytoplasmic [Class 3] |
| PA14_18210 | ihfA | integration host factor subunit alpha | DNA replication, recombination, modification and repair | Cytoplasmic [Class 3] |
| PA14_22380 | ihfB | integration host factor subunit beta | DNA replication, recombination, modification and repair | Cytoplasmic [Class 3] |
| PA14_67120 | lexA | LexA repressor | DNA replication, recombination, modification and repair | Cytoplasmic [Class 3] |
| PA14_70230 | nudC | NADH pyrophosphatase | DNA replication, recombination, modification and repair | Cytoplasmic [Class 3] |
| PA14_44610 | ada | putative methylated-DNA--protein-cysteine methyltransferase | DNA replication, recombination, modification and repair | Cytoplasmic [Class 3] |
| PA14_17540 | yhhF | putative methyltransferase | DNA replication, recombination, modification and repair | Unknown [Class 3] |
| PA14_41060 | recR | recombination protein RecR | DNA replication, recombination, modification and repair | Cytoplasmic [Class 3] |
| PA14_17230 | recX | recombination regulator RecX | DNA replication, recombination, modification and repair | Cytoplasmic [Class 3] |
| PA14_18700 | rnhA | ribonuclease H | DNA replication, recombination, modification and repair | Unknown [Class 3] |
| PA14_51790 | rnhB | ribonuclease HII | DNA replication, recombination, modification and repair | Cytoplasmic [Class 3] |
| PA14_51800 | rnt | ribonuclease T | DNA replication, recombination, modification and repair | Cytoplasmic [Class 3] |
| PA14_09200 | ssb | single-stranded DNA-binding protein | DNA replication, recombination, modification and repair | Cytoplasmic [Class 3] |
| PA14_04860 | PA14_18210 | TatD family deoxyribonuclease | DNA replication, recombination, modification and repair | Cytoplasmic [Class 3] |
| PA14_73310 | ccoO | cbb3-type cytochrome c oxidase subunit II | Energy metabolism | Cytoplasmic [Class 3] |
| PA14_73230 | cioB | CioB, cyanide insensitive terminal oxidase | Energy metabolism | Cytoplasmic Membrane [Class 3] |
| PA14_73290 | ccmG | cytochrome C biogenesis protein CcmG | Energy metabolism | Cytoplasmic Membrane [Class 3] |
| PA14_73250 | exaB | cytochrome c550 | Energy metabolism | Unknown [Class 3] |
| PA14_73320 | ccmH | cytochrome C-type biogenesis protein CcmH | Energy metabolism | Cytoplasmic Membrane [Class 3] |
| PA14_45300 | cyoC | cytochrome o ubiquinol oxidase subunit III | Energy metabolism | Cytoplasmic Membrane [Class 3] |
| PA14_45290 | lpdG | dihydrolipoamide dehydrogenase | Energy metabolism | Cytoplasmic [Class 3] |
| PA14_44350 | etfA | electron transfer flavoprotein alpha-subunit | Energy metabolism | Unknown [Class 3] |
| PA14_44400 | etfB | electron transfer flavoprotein beta-subunit | Energy metabolism | Unknown [Class 3] |
| PA14_13040 | rnfB | electron transport complex protein RnfB | Energy metabolism | Cytoplasmic [Class 3] |
| PA14_47180 | rnfE | electron transport complex RsxE subunit | Energy metabolism | Cytoplasmic Membrane [Class 3] |
| PA14_30400 | atpB | F0F1 ATP synthase subunit A | Energy metabolism | Cytoplasmic Membrane [Class 3] |
| PA14_17320 | atpF | F0F1 ATP synthase subunit B | Energy metabolism | Cytoplasmic Membrane [Class 3] |
| PA14_25880 | atpC | F0F1 ATP synthase subunit epsilon | Energy metabolism | Cytoplasmic [Class 3] |
| PA14_25860 | atpG | F0F1 ATP synthase subunit gamma | Energy metabolism | Cytoplasmic [Class 3] |
| PA14_38850 | atpI | F0F1 ATP synthase subunit I | Energy metabolism | Cytoplasmic Membrane [Class 3] |
| PA14_38840 | fdxA | ferredoxin I | Energy metabolism | Cytoplasmic [Class 3] |
| PA14_63550 | napF | ferredoxin protein NapF | Energy metabolism | Cytoplasmic [Class 3] |
| PA14_17490 | fdhE | formate dehydrogenase accessory protein FdhE | Energy metabolism | Cytoplasmic [Class 3] |
| PA14_61060 | glpD | glycerol-3-phosphate dehydrogenase | Energy metabolism | Cytoplasmic [Class 3] |
| PA14_37090 | PA14_42120 | hypothetical protein | Energy metabolism | Unknown [Class 3] |
| PA14_17930 | PA14_56260 | hypothetical protein | Energy metabolism | Cytoplasmic [Class 3] |
| PA14_43970 | PA14_66000 | hypothetical protein | Energy metabolism | Cytoplasmic [Class 3] |
| PA14_01600 | PA14_66010 | hypothetical protein | Energy metabolism | Cytoplasmic [Class 3] |
| PA14_49220 | ppa | inorganic pyrophosphatase | Energy metabolism | Cytoplasmic [Class 3] |
| PA14_13810 | nqrC | Na(+)-translocating NADH-quinone reductase subunit C | Energy metabolism | Unknown [Class 3] |
| PA14_06730 | exaC | NAD+ dependent acetaldehyde dehydrogenase | Energy metabolism | Cytoplasmic [Class 3] |
| PA14_06710 | nuoE | NADH dehydrogenase subunit E | Energy metabolism | Cytoplasmic [Class 3] |
| PA14_41540 | nuoH | NADH dehydrogenase subunit H | Energy metabolism | Cytoplasmic Membrane [Class 3] |
| PA14_06810 | nuoI | NADH dehydrogenase subunit I | Energy metabolism | Cytoplasmic [Class 3] |
| PA14_20180 | nuoJ | NADH dehydrogenase subunit J | Energy metabolism | Cytoplasmic Membrane [Class 3] |
| PA14_25320 | nuoM | NADH dehydrogenase subunit M | Energy metabolism | Cytoplasmic Membrane [Class 3] |
| PA14_29980 | norC | nitric-oxide reductase subunit C | Energy metabolism | Cytoplasmic Membrane [Class 3] |
| PA14_29930 | nosF | NosF protein | Energy metabolism | Cytoplasmic [Class 3] |
| PA14_29920 | eno | phosphopyruvate hydratase | Energy metabolism | Cytoplasmic [Class 3] |
| PA14_29900 | gbsA | putative aldehyde dehydrogenase | Energy metabolism | Cytoplasmic [Class 3] |
| PA14_29860 | mmsA | putative aldehyde dehydrogenase | Energy metabolism | Cytoplasmic [Class 3] |
| PA14_07030 | PA14_58880 | putative ATPase | Energy metabolism | Cytoplasmic [Class 3] |
| PA14_29030 | nirC | putative c-type cytochrome precursor | Energy metabolism | Periplasmic [Class 3] |
| PA14_29040 | PA14_57560 | putative cytochrome b | Energy metabolism | Cytoplasmic Membrane [Class 3] |
| PA14_32420 | PA14_07030 | putative cytochrome c' | Energy metabolism | Unknown [Class 3] |
| PA14_34250 | PA14_44390 | putative cytochrome c oxidase subunit | Energy metabolism | Cytoplasmic Membrane [Class 3] |
| PA14_42120 | ccoP | putative cytochrome c oxidase, cbb3-type, subunit III | Energy metabolism | Unknown [Class 3] |
| PA14_44390 | PA14_57540 | putative cytochrome c1 precursor | Energy metabolism | Unknown [Class 3] |
| PA14_45190 | PA14_29040 | putative ferredoxin | Energy metabolism | Cytoplasmic [Class 3] |
| PA14_56260 | PA14_29030 | putative FMN oxidoreductase | Energy metabolism | Cytoplasmic [Class 3] |
| PA14_57540 | PA14_45190 | putative glycerol kinase | Energy metabolism | Cytoplasmic [Class 3] |
| PA14_57560 | PA14_34250 | putative glycerophosphoryl diester phosphodiesterase | Energy metabolism | Periplasmic [Class 3] |
| PA14_58880 | fnr-2 | putative oxidoreductase | Energy metabolism | Cytoplasmic [Class 3] |
| PA14_66000 | PA14_32420 | putative oxidoreductase | Energy metabolism | Cytoplasmic [Class 3] |
| PA14_66010 | yhfP | putative oxidoreductase | Energy metabolism | Cytoplasmic [Class 3] |
| PA14_11690 | trxA | putative thioredoxin | Energy metabolism | Cytoplasmic [Class 3] |
| PA14_18930 | nirD | putative transcriptional regulator | Energy metabolism | Cytoplasmic [Class 3] |
| PA14_18890 | nirD | putative transcriptional regulator | Energy metabolism | Cytoplasmic [Class 3] |
| PA14_04310 | narJ | respiratory nitrate reductase delta chain | Energy metabolism | Cytoplasmic [Class 3] |
| PA14_44020 | rpiA | ribose-5-phosphate isomerase A | Energy metabolism | Cytoplasmic [Class 3] |
| PA14_44060 | sdhB | succinate dehydrogenase iron-sulfur subunit | Energy metabolism | Cytoplasmic Membrane [Class 3] |
| PA14_43940 | sdhC | succinate dehydrogenase, cytochrome b556 subunit | Energy metabolism | Cytoplasmic Membrane [Class 3] |
| PA14_69200* | sucD | succinyl-CoA synthetase subunit alpha | Energy metabolism | Cytoplasmic [Class 3] |
| PA14_40830 | dsrE | sulfur transfer complex subunit TusD | Energy metabolism | Cytoplasmic [Class 3] |
| PA14_64100 | fabZ | (3R)-hydroxymyristoyl-ACP dehydratase | Fatty acid and phospholipid metabolism | Cytoplasmic [Class 3] |
| PA14_06450 | fabG | 3-ketoacyl-ACP reductase | Fatty acid and phospholipid metabolism | Cytoplasmic [Class 3] |
| PA14_25360 | fabH2 | 3-oxoacyl-(acyl carrier protein) synthase III | Fatty acid and phospholipid metabolism | Cytoplasmic [Class 3] |
| PA14_38630 | atoB | acetyl-CoA acetyltransferase | Fatty acid and phospholipid metabolism | Cytoplasmic [Class 3] |
| PA14_63270 | PA14_42090 | acetyl-CoA acetyltransferase | Fatty acid and phospholipid metabolism | Cytoplasmic [Class 3] |
| PA14_20950 | accB | acetyl-CoA carboxylase biotin carboxyl carrier protein subunit | Fatty acid and phospholipid metabolism | Unknown [Class 3] |
| PA14_17190 | accC | acetyl-CoA carboxylase biotin carboxylase subunit | Fatty acid and phospholipid metabolism | Cytoplasmic [Class 3] |
| PA14_04460 | phaA | acyl-CoA thiolase | Fatty acid and phospholipid metabolism | Cytoplasmic [Class 3] |
| PA14_60360 | PA14_41950 | enoyl-CoA hydratase | Fatty acid and phospholipid metabolism | Cytoplasmic [Class 3] |
| PA14_00060 | PA14_43440 | enoyl-CoA hydratase | Fatty acid and phospholipid metabolism | Cytoplasmic [Class 3] |
| PA14_00960 | PA14_13320 | hypothetical protein | Fatty acid and phospholipid metabolism | Unknown [Class 3] |
| PA14_01550 | PA14_29720 | hypothetical protein | Fatty acid and phospholipid metabolism | Unknown [Class 3] |
| PA14_13320 | PA14_40060 | hypothetical protein | Fatty acid and phospholipid metabolism | Cytoplasmic Membrane [Class 3] |
| PA14_16560 | PA14_43300 | hypothetical protein | Fatty acid and phospholipid metabolism | Extracellular [Class 3] |
| PA14_16640 | PA14_43600 | hypothetical protein | Fatty acid and phospholipid metabolism | Unknown [Class 3] |
| PA14_19430 | PA14_48800 | hypothetical protein | Fatty acid and phospholipid metabolism | Cytoplasmic [Class 3] |
| PA14_25820 | PA14_58210 | hypothetical protein | Fatty acid and phospholipid metabolism | Unknown [Class 3] |
| PA14_26550 | PA14_64010 | hypothetical protein | Fatty acid and phospholipid metabolism | Unknown [Class 3] |
| PA14_29720 | PA14_64610 | hypothetical protein | Fatty acid and phospholipid metabolism | Unknown [Class 3] |
| PA14_31750 | yfiM | hypothetical protein | Fatty acid and phospholipid metabolism | Cytoplasmic Membrane [Class 3] |
| PA14_37370 | lspA | lipoprotein signal peptidase | Fatty acid and phospholipid metabolism | Cytoplasmic Membrane [Class 3] |
| PA14_40060 | pssA | phosphatidylserine synthase | Fatty acid and phospholipid metabolism | Cytoplasmic Membrane [Class 3] |
| PA14_40310 | lgt | prolipoprotein diacylglyceryl transferase | Fatty acid and phospholipid metabolism | Cytoplasmic Membrane [Class 3] |
| PA14_41950 | PA14_40310 | putative acyl carrier protein | Fatty acid and phospholipid metabolism | Cytoplasmic [Class 3] |
| PA14_42090 | PA14_65820 | putative acyl-CoA dehydrogenase | Fatty acid and phospholipid metabolism | Cytoplasmic [Class 3] |
| PA14_42940 | PA14_19430 | putative acyl-CoA thiolase | Fatty acid and phospholipid metabolism | Cytoplasmic [Class 3] |
| PA14_43290 | PA14_00060 | putative acyltransferase | Fatty acid and phospholipid metabolism | Cytoplasmic Membrane [Class 3] |
| PA14_43300 | PA14_31750 | putative acyltransferase | Fatty acid and phospholipid metabolism | Unknown [Class 3] |
| PA14_43440 | PA14_37370 | putative esterase | Fatty acid and phospholipid metabolism | Cytoplasmic [Class 3] |
| PA14_43600 | PA14_00960 | putative lipoprotein | Fatty acid and phospholipid metabolism | Cytoplasmic Membrane [Class 3] |
| PA14_48800 | PA14_01550 | putative lipoprotein | Fatty acid and phospholipid metabolism | Unknown [Class 3] |
| PA14_58210 | PA14_16560 | putative lipoprotein | Fatty acid and phospholipid metabolism | Unknown [Class 3] |
| PA14_61390 | PA14_16640 | putative lipoprotein | Fatty acid and phospholipid metabolism | Unknown [Class 3] |
| PA14_64010 | PA14_25820 | putative lipoprotein | Fatty acid and phospholipid metabolism | Unknown [Class 3] |
| PA14_64610 | PA14_26550 | putative lipoprotein | Fatty acid and phospholipid metabolism | Unknown [Class 3] |
| PA14_65820 | PA14_42940 | putative lipoprotein | Fatty acid and phospholipid metabolism | Unknown [Class 3] |
| PA14_67460 | PA14_43290 | putative lipoprotein | Fatty acid and phospholipid metabolism | Unknown [Class 3] |
| PA14_70060 | PA14_61390 | putative lipoprotein | Fatty acid and phospholipid metabolism | Unknown [Class 3] |
| PA14_26010 | PA14_67460 | putative lipoprotein | Fatty acid and phospholipid metabolism | Unknown [Class 3] |
| PA14_62120 | PA14_70060 | putative lipoprotein | Fatty acid and phospholipid metabolism | Unknown [Class 3] |
| PA14_06350 | apbE | putative thiamine biosynthesis lipoprotein | Fatty acid and phospholipid metabolism | Unknown [Class 3] |
| PA14_07710 | PA14_66990 | 16S ribosomal RNA methyltransferase RsmE | Hypothetical, unclassified, unknown | Cytoplasmic [Class 3] |
| PA14_41610 | PA14_21530 | ankyrin domain-containing protein | Hypothetical, unclassified, unknown | Periplasmic [Class 3] |
| PA14_50280 | apaG | ApaG | Hypothetical, unclassified, unknown | Unknown [Class 3] |
| PA14_09350 | PA14_48920 | bacteriophage protein | Hypothetical, unclassified, unknown | Cytoplasmic Membrane [Class 3] |
| PA14_55510 | PA14_03090 | CBS domain-containing protein | Hypothetical, unclassified, unknown | Unknown [Class 3] |
| PA14_00160 | crfX | hypothetical protein | Hypothetical, unclassified, unknown | Unknown [Class 3] |
| PA14_00520 | flaG | hypothetical protein | Hypothetical, unclassified, unknown | Unknown [Class 3] |
| PA14_00650 | fptB | hypothetical protein | Hypothetical, unclassified, unknown | Cytoplasmic Membrane [Class 3] |
| PA14_00670 | hxcP | hypothetical protein | Hypothetical, unclassified, unknown | Cytoplasmic Membrane [Class 3] |
| PA14_00720 | PA14_00520 | hypothetical protein | Hypothetical, unclassified, unknown | Unknown [Class 3] |
| PA14_00900 | PA14_00650 | hypothetical protein | Hypothetical, unclassified, unknown | Unknown [Class 3] |
| PA14_01010 | PA14_00670 | hypothetical protein | Hypothetical, unclassified, unknown | Cytoplasmic [Class 3] |
| PA14_01060 | PA14_00720 | hypothetical protein | Hypothetical, unclassified, unknown | Unknown [Class 3] |
| PA14_01350 | PA14_00900 | hypothetical protein | Hypothetical, unclassified, unknown | Unknown [Class 3] |
| PA14_02010 | PA14_01010 | hypothetical protein | Hypothetical, unclassified, unknown | Cytoplasmic [Class 3] |
| PA14_02100 | PA14_01060 | hypothetical protein | Hypothetical, unclassified, unknown | Unknown [Class 3] |
| PA14_02960 | PA14_01350 | hypothetical protein | Hypothetical, unclassified, unknown | Unknown [Class 3] |
| PA14_03090 | PA14_02010 | hypothetical protein | Hypothetical, unclassified, unknown | Unknown [Class 3] |
| PA14_03110 | PA14_02100 | hypothetical protein | Hypothetical, unclassified, unknown | Cytoplasmic [Class 3] |
| PA14_03420 | PA14_02960 | hypothetical protein | Hypothetical, unclassified, unknown | Cytoplasmic [Class 3] |
| PA14_03520 | PA14_03110 | hypothetical protein | Hypothetical, unclassified, unknown | Unknown [Class 3] |
| PA14_04060 | PA14_03420 | Hypothetical protein | Hypothetical, unclassified, unknown | Unknown [Class 3] |
| PA14_04070 | PA14_03520 | hypothetical protein | Hypothetical, unclassified, unknown | Unknown [Class 3] |
| PA14_04180 | PA14_04060 | hypothetical protein | Hypothetical, unclassified, unknown | Unknown [Class 3] |
| PA14_04430 | PA14_04070 | hypothetical protein | Hypothetical, unclassified, unknown | Cytoplasmic [Class 3] |
| PA14_04520 | PA14_04180 | hypothetical protein | Hypothetical, unclassified, unknown | Unknown [Class 3] |
| PA14_04850 | PA14_04430 | hypothetical protein | Hypothetical, unclassified, unknown | Unknown [Class 3] |
| PA14_05600 | PA14_04520 | hypothetical protein | Hypothetical, unclassified, unknown | Cytoplasmic Membrane [Class 3] |
| PA14_05630 | PA14_04850 | hypothetical protein | Hypothetical, unclassified, unknown | Unknown [Class 3] |
| PA14_05775 | PA14_05600 | hypothetical protein | Hypothetical, unclassified, unknown | Unknown [Class 3] |
| PA14_05970 | PA14_05630 | hypothetical protein | Hypothetical, unclassified, unknown | Cytoplasmic Membrane [Class 3] |
| PA14_06010 | PA14_05775 | hypothetical protein | Hypothetical, unclassified, unknown | Unknown [Class 3] |
| PA14_06040 | PA14_05970 | hypothetical protein | Hypothetical, unclassified, unknown | Unknown [Class 3] |
| PA14_06090 | PA14_06010 | hypothetical protein | Hypothetical, unclassified, unknown | Unknown [Class 3] |
| PA14_06150 | PA14_06040 | hypothetical protein | Hypothetical, unclassified, unknown | Unknown [Class 3] |
| PA14_06390 | PA14_06090 | hypothetical protein | Hypothetical, unclassified, unknown | Cytoplasmic Membrane [Class 3] |
| PA14_06900 | PA14_06150 | hypothetical protein | Hypothetical, unclassified, unknown | Cytoplasmic [Class 3] |
| PA14_07200 | PA14_06390 | hypothetical protein | Hypothetical, unclassified, unknown | Cytoplasmic Membrane [Class 3] |
| PA14_07330 | PA14_06900 | hypothetical protein | Hypothetical, unclassified, unknown | Unknown [Class 3] |
| PA14_07355 | PA14_07200 | hypothetical protein | Hypothetical, unclassified, unknown | Unknown [Class 3] |
| PA14_07410 | PA14_07330 | hypothetical protein | Hypothetical, unclassified, unknown | Cytoplasmic Membrane [Class 3] |
| PA14_07460 | PA14_07355 | hypothetical protein | Hypothetical, unclassified, unknown | Unknown [Class 3] |
| PA14_07550 | PA14_07410 | hypothetical protein | Hypothetical, unclassified, unknown | Unknown [Class 3] |
| PA14_08000 | PA14_07460 | hypothetical protein | Hypothetical, unclassified, unknown | Unknown [Class 3] |
| PA14_08100 | PA14_07550 | hypothetical protein | Hypothetical, unclassified, unknown | Cytoplasmic [Class 3] |
| PA14_08180 | PA14_08000 | hypothetical protein | Hypothetical, unclassified, unknown | Unknown [Class 3] |
| PA14_08220 | PA14_08100 | hypothetical protein | Hypothetical, unclassified, unknown | Unknown [Class 3] |
| PA14_08230 | PA14_08180 | hypothetical protein | Hypothetical, unclassified, unknown | Unknown [Class 3] |
| PA14_10080 | PA14_08220 | hypothetical protein | Hypothetical, unclassified, unknown | Cytoplasmic [Class 3] |
| PA14_10380 | PA14_08230 | hypothetical protein | Hypothetical, unclassified, unknown | Unknown [Class 3] |
| PA14_10400 | PA14_10080 | hypothetical protein | Hypothetical, unclassified, unknown | Unknown [Class 3] |
| PA14_10780 | PA14_10380 | hypothetical protein | Hypothetical, unclassified, unknown | Unknown [Class 3] |
| PA14_10950 | PA14_10400 | hypothetical protein | Hypothetical, unclassified, unknown | Unknown [Class 3] |
| PA14_11010 | PA14_10780 | hypothetical protein | Hypothetical, unclassified, unknown | Cytoplasmic [Class 3] |
| PA14_11160 | PA14_10950 | hypothetical protein | Hypothetical, unclassified, unknown | Cytoplasmic [Class 3] |
| PA14_11490 | PA14_11010 | hypothetical protein | Hypothetical, unclassified, unknown | Unknown [Class 3] |
| PA14_11520 | PA14_11160 | hypothetical protein | Hypothetical, unclassified, unknown | Cytoplasmic Membrane [Class 3] |
| PA14_11880 | PA14_11490 | hypothetical protein | Hypothetical, unclassified, unknown | Unknown [Class 3] |
| PA14_11920 | PA14_11520 | hypothetical protein | Hypothetical, unclassified, unknown | Cytoplasmic Membrane [Class 3] |
| PA14_12030 | PA14_11880 | hypothetical protein | Hypothetical, unclassified, unknown | Unknown [Class 3] |
| PA14_12050 | PA14_11920 | hypothetical protein | Hypothetical, unclassified, unknown | Cytoplasmic Membrane [Class 3] |
| PA14_12110 | PA14_12030 | hypothetical protein | Hypothetical, unclassified, unknown | Cytoplasmic [Class 3] |
| PA14_12640 | PA14_12110 | hypothetical protein | Hypothetical, unclassified, unknown | Cytoplasmic [Class 3] |
| PA14_12740 | PA14_12640 | hypothetical protein | Hypothetical, unclassified, unknown | Unknown [Class 3] |
| PA14_12910 | PA14_12740 | hypothetical protein | Hypothetical, unclassified, unknown | Unknown [Class 3] |
| PA14_13370 | PA14_12910 | hypothetical protein | Hypothetical, unclassified, unknown | Unknown [Class 3] |
| PA14_13380 | PA14_13370 | hypothetical protein | Hypothetical, unclassified, unknown | Cytoplasmic [Class 3] |
| PA14_13630 | PA14_13380 | hypothetical protein | Hypothetical, unclassified, unknown | Unknown [Class 3] |
| PA14_13710 | PA14_13630 | hypothetical protein | Hypothetical, unclassified, unknown | Cytoplasmic Membrane [Class 3] |
| PA14_13720 | PA14_13710 | hypothetical protein | Hypothetical, unclassified, unknown | Unknown [Class 3] |
| PA14_13880 | PA14_13720 | hypothetical protein | Hypothetical, unclassified, unknown | Cytoplasmic [Class 3] |
| PA14_13910 | PA14_13880 | hypothetical protein | Hypothetical, unclassified, unknown | Unknown [Class 3] |
| PA14_13950 | PA14_13910 | hypothetical protein | Hypothetical, unclassified, unknown | Unknown [Class 3] |
| PA14_13960 | PA14_13950 | hypothetical protein | Hypothetical, unclassified, unknown | Unknown [Class 3] |
| PA14_14220 | PA14_13960 | hypothetical protein | Hypothetical, unclassified, unknown | Unknown [Class 3] |
| PA14_14320 | PA14_14320 | hypothetical protein | Hypothetical, unclassified, unknown | Cytoplasmic Membrane [Class 3] |
| PA14_14330 | PA14_14330 | hypothetical protein | Hypothetical, unclassified, unknown | Unknown [Class 3] |
| PA14_14450 | PA14_14450 | hypothetical protein | Hypothetical, unclassified, unknown | Unknown [Class 3] |
| PA14_14560 | PA14_14560 | hypothetical protein | Hypothetical, unclassified, unknown | Unknown [Class 3] |
| PA14_14810 | PA14_14810 | hypothetical protein | Hypothetical, unclassified, unknown | Unknown [Class 3] |
| PA14_14900 | PA14_14900 | hypothetical protein | Hypothetical, unclassified, unknown | Unknown [Class 3] |
| PA14_14975 | PA14_14975 | hypothetical protein | Hypothetical, unclassified, unknown | Cytoplasmic Membrane [Class 3] |
| PA14_15090 | PA14_15090 | hypothetical protein | Hypothetical, unclassified, unknown | Cytoplasmic Membrane [Class 3] |
| PA14_15110 | PA14_15110 | hypothetical protein | Hypothetical, unclassified, unknown | Cytoplasmic Membrane [Class 3] |
| PA14_15410 | PA14_15410 | hypothetical protein | Hypothetical, unclassified, unknown | Unknown [Class 3] |
| PA14_15490 | PA14_15490 | hypothetical protein | Hypothetical, unclassified, unknown | Cytoplasmic Membrane [Class 3] |
| PA14_15750 | PA14_15750 | hypothetical protein | Hypothetical, unclassified, unknown | Unknown [Class 3] |
| PA14_16020 | PA14_16020 | hypothetical protein | Hypothetical, unclassified, unknown | Unknown [Class 3] |
| PA14_16210 | PA14_16210 | hypothetical protein | Hypothetical, unclassified, unknown | Cytoplasmic [Class 3] |
| PA14_16270 | PA14_16270 | hypothetical protein | Hypothetical, unclassified, unknown | Unknown [Class 3] |
| PA14_16290 | PA14_16290 | hypothetical protein | Hypothetical, unclassified, unknown | Cytoplasmic [Class 3] |
| PA14_16300 | PA14_16300 | hypothetical protein | Hypothetical, unclassified, unknown | Unknown [Class 3] |
| PA14_16770 | PA14_16770 | hypothetical protein | Hypothetical, unclassified, unknown | Cytoplasmic [Class 3] |
| PA14_18100 | PA14_18100 | hypothetical protein | Hypothetical, unclassified, unknown | Unknown [Class 3] |
| PA14_19010 | PA14_19010 | hypothetical protein | Hypothetical, unclassified, unknown | Unknown [Class 3] |
| PA14_19330 | PA14_19330 | hypothetical protein | Hypothetical, unclassified, unknown | Cytoplasmic [Class 3] |
| PA14_19600 | PA14_19600 | hypothetical protein | Hypothetical, unclassified, unknown | Unknown [Class 3] |
| PA14_19610 | PA14_19610 | hypothetical protein | Hypothetical, unclassified, unknown | Unknown [Class 3] |
| PA14_19750 | PA14_19750 | hypothetical protein | Hypothetical, unclassified, unknown | Unknown [Class 3] |
| PA14_19860 | PA14_19860 | hypothetical protein | Hypothetical, unclassified, unknown | Cytoplasmic [Class 3] |
| PA14_19950 | PA14_19950 | hypothetical protein | Hypothetical, unclassified, unknown | Unknown [Class 3] |
| PA14_19960 | PA14_19960 | hypothetical protein | Hypothetical, unclassified, unknown | Unknown [Class 3] |
| PA14_20460 | PA14_20460 | hypothetical protein | Hypothetical, unclassified, unknown | Cytoplasmic Membrane [Class 3] |
| PA14_20530 | PA14_20530 | hypothetical protein | Hypothetical, unclassified, unknown | Cytoplasmic [Class 3] |
| PA14_21380 | PA14_21380 | hypothetical protein | Hypothetical, unclassified, unknown | Cytoplasmic [Class 3] |
| PA14_21460 | PA14_21460 | hypothetical protein | Hypothetical, unclassified, unknown | Cytoplasmic [Class 3] |
| PA14_21470 | PA14_21470 | hypothetical protein | Hypothetical, unclassified, unknown | Unknown [Class 3] |
| PA14_21510 | PA14_21510 | hypothetical protein | Hypothetical, unclassified, unknown | Unknown [Class 3] |
| PA14_21530 | PA14_21560 | hypothetical protein | Hypothetical, unclassified, unknown | Unknown [Class 3] |
| PA14_21560 | PA14_21570 | hypothetical protein | Hypothetical, unclassified, unknown | Unknown [Class 3] |
| PA14_21570 | PA14_21830 | hypothetical protein | Hypothetical, unclassified, unknown | Cytoplasmic Membrane [Class 3] |
| PA14_21830 | PA14_21860 | hypothetical protein | Hypothetical, unclassified, unknown | Unknown [Class 3] |
| PA14_21860 | PA14_22180 | hypothetical protein | Hypothetical, unclassified, unknown | Unknown [Class 3] |
| PA14_22180 | PA14_22190 | hypothetical protein | Hypothetical, unclassified, unknown | Unknown [Class 3] |
| PA14_22190 | PA14_22220 | hypothetical protein | Hypothetical, unclassified, unknown | Unknown [Class 3] |
| PA14_22220 | PA14_22230 | hypothetical protein | Hypothetical, unclassified, unknown | Unknown [Class 3] |
| PA14_22230 | PA14_22480 | hypothetical protein | Hypothetical, unclassified, unknown | Unknown [Class 3] |
| PA14_22480 | PA14_22510 | hypothetical protein | Hypothetical, unclassified, unknown | Unknown [Class 3] |
| PA14_22510 | PA14_22720 | hypothetical protein | Hypothetical, unclassified, unknown | Cytoplasmic Membrane [Class 3] |
| PA14_22720 | PA14_22770 | hypothetical protein | Hypothetical, unclassified, unknown | Cytoplasmic Membrane [Class 3] |
| PA14_22770 | PA14_24410 | hypothetical protein | Hypothetical, unclassified, unknown | Unknown [Class 3] |
| PA14_24410 | PA14_24630 | hypothetical protein | Hypothetical, unclassified, unknown | Unknown [Class 3] |
| PA14_24630 | PA14_24740 | hypothetical protein | Hypothetical, unclassified, unknown | Unknown [Class 3] |
| PA14_24740 | PA14_24770 | hypothetical protein | Hypothetical, unclassified, unknown | Unknown [Class 3] |
| PA14_24770 | PA14_24850 | hypothetical protein | Hypothetical, unclassified, unknown | Unknown [Class 3] |
| PA14_24850 | PA14_24980 | hypothetical protein | Hypothetical, unclassified, unknown | Cytoplasmic [Class 3] |
| PA14_24980 | PA14_25140 | hypothetical protein | Hypothetical, unclassified, unknown | Cytoplasmic [Class 3] |
| PA14_25140 | PA14_25370 | hypothetical protein | Hypothetical, unclassified, unknown | Unknown [Class 3] |
| PA14_25370 | PA14_25410 | hypothetical protein | Hypothetical, unclassified, unknown | Unknown [Class 3] |
| PA14_25410 | PA14_25520 | hypothetical protein | Hypothetical, unclassified, unknown | Unknown [Class 3] |
| PA14_25520 | PA14_26300 | hypothetical protein | Hypothetical, unclassified, unknown | Cytoplasmic Membrane [Class 3] |
| PA14_26300 | PA14_26580 | hypothetical protein | Hypothetical, unclassified, unknown | Unknown [Class 3] |
| PA14_26580 | PA14_26780 | hypothetical protein | Hypothetical, unclassified, unknown | Cytoplasmic Membrane [Class 3] |
| PA14_26780 | PA14_26990 | hypothetical protein | Hypothetical, unclassified, unknown | Cytoplasmic Membrane [Class 3] |
| PA14_26990 | PA14_27290 | hypothetical protein | Hypothetical, unclassified, unknown | Unknown [Class 3] |
| PA14_27290 | PA14_27490 | hypothetical protein | Hypothetical, unclassified, unknown | Unknown [Class 3] |
| PA14_27490 | PA14_27660 | hypothetical protein | Hypothetical, unclassified, unknown | Unknown [Class 3] |
| PA14_27660 | PA14_27710 | hypothetical protein | Hypothetical, unclassified, unknown | Cytoplasmic [Class 3] |
| PA14_27710 | PA14_27930 | hypothetical protein | Hypothetical, unclassified, unknown | Cytoplasmic [Class 3] |
| PA14_27930 | PA14_28070 | hypothetical protein | Hypothetical, unclassified, unknown | Cytoplasmic [Class 3] |
| PA14_28070 | PA14_28090 | hypothetical protein | Hypothetical, unclassified, unknown | Cytoplasmic Membrane [Class 3] |
| PA14_28090 | PA14_28110 | hypothetical protein | Hypothetical, unclassified, unknown | Unknown [Class 3] |
| PA14_28110 | PA14_28120 | hypothetical protein | Hypothetical, unclassified, unknown | Unknown [Class 3] |
| PA14_28120 | PA14_28140 | hypothetical protein | Hypothetical, unclassified, unknown | Cytoplasmic Membrane [Class 3] |
| PA14_28140 | PA14_28200 | hypothetical protein | Hypothetical, unclassified, unknown | Unknown [Class 3] |
| PA14_28200 | PA14_28220 | hypothetical protein | Hypothetical, unclassified, unknown | Cytoplasmic Membrane [Class 3] |
| PA14_28220 | PA14_28230 | hypothetical protein | Hypothetical, unclassified, unknown | Unknown [Class 3] |
| PA14_28230 | PA14_28260 | hypothetical protein | Hypothetical, unclassified, unknown | Cytoplasmic [Class 3] |
| PA14_28260 | PA14_28330 | hypothetical protein | Hypothetical, unclassified, unknown | Cytoplasmic Membrane [Class 3] |
| PA14_28330 | PA14_28360 | hypothetical protein | Hypothetical, unclassified, unknown | Cytoplasmic Membrane [Class 3] |
| PA14_28360 | PA14_28370 | hypothetical protein | Hypothetical, unclassified, unknown | Cytoplasmic Membrane [Class 3] |
| PA14_28370 | PA14_28500 | hypothetical protein | Hypothetical, unclassified, unknown | Cytoplasmic [Class 3] |
| PA14_28500 | PA14_28540 | hypothetical protein | Hypothetical, unclassified, unknown | Unknown [Class 3] |
| PA14_28540 | PA14_28560 | hypothetical protein | Hypothetical, unclassified, unknown | Unknown [Class 3] |
| PA14_28560 | PA14_28580 | hypothetical protein | Hypothetical, unclassified, unknown | Unknown [Class 3] |
| PA14_28580 | PA14_28850 | hypothetical protein | Hypothetical, unclassified, unknown | Unknown [Class 3] |
| PA14_28850 | PA14_28870 | hypothetical protein | Hypothetical, unclassified, unknown | Unknown [Class 3] |
| PA14_28870 | PA14_28950 | hypothetical protein | Hypothetical, unclassified, unknown | Unknown [Class 3] |
| PA14_28950 | PA14_28960 | hypothetical protein | Hypothetical, unclassified, unknown | Cytoplasmic [Class 3] |
| PA14_28960 | PA14_29190 | hypothetical protein | Hypothetical, unclassified, unknown | Unknown [Class 3] |
| PA14_29190 | PA14_29200 | hypothetical protein | Hypothetical, unclassified, unknown | Unknown [Class 3] |
| PA14_29200 | PA14_29650 | hypothetical protein | Hypothetical, unclassified, unknown | Cytoplasmic Membrane [Class 3] |
| PA14_29650 | PA14_29680 | hypothetical protein | Hypothetical, unclassified, unknown | Unknown [Class 3] |
| PA14_29680 | PA14_29750 | hypothetical protein | Hypothetical, unclassified, unknown | Cytoplasmic Membrane [Class 3] |
| PA14_29750 | PA14_30140 | hypothetical protein | Hypothetical, unclassified, unknown | Unknown [Class 3] |
| PA14_30140 | PA14_30950 | hypothetical protein | Hypothetical, unclassified, unknown | Unknown [Class 3] |
| PA14_30950 | PA14_30980 | hypothetical protein | Hypothetical, unclassified, unknown | Unknown [Class 3] |
| PA14_30980 | PA14_31130 | hypothetical protein | Hypothetical, unclassified, unknown | Unknown [Class 3] |
| PA14_31130 | PA14_31200 | hypothetical protein | Hypothetical, unclassified, unknown | Cytoplasmic [Class 3] |
| PA14_31200 | PA14_31250 | hypothetical protein | Hypothetical, unclassified, unknown | Unknown [Class 3] |
| PA14_31250 | PA14_31360 | hypothetical protein | Hypothetical, unclassified, unknown | Unknown [Class 3] |
| PA14_31360 | PA14_31450 | hypothetical protein | Hypothetical, unclassified, unknown | Unknown [Class 3] |
| PA14_31450 | PA14_31740 | hypothetical protein | Hypothetical, unclassified, unknown | Cytoplasmic Membrane [Class 3] |
| PA14_31740 | PA14_32350 | hypothetical protein | Hypothetical, unclassified, unknown | Unknown [Class 3] |
| PA14_32350 | PA14_32810 | hypothetical protein | Hypothetical, unclassified, unknown | Unknown [Class 3] |
| PA14_32810 | PA14_32890 | hypothetical protein | Hypothetical, unclassified, unknown | Unknown [Class 3] |
| PA14_32890 | PA14_33060 | hypothetical protein | Hypothetical, unclassified, unknown | Cytoplasmic [Class 3] |
| PA14_33060 | PA14_33120 | hypothetical protein | Hypothetical, unclassified, unknown | Unknown [Class 3] |
| PA14_33120 | PA14_33160 | hypothetical protein | Hypothetical, unclassified, unknown | Unknown [Class 3] |
| PA14_33160 | PA14_33200 | hypothetical protein | Hypothetical, unclassified, unknown | Cytoplasmic Membrane [Class 3] |
| PA14_33200 | PA14_33220 | hypothetical protein | Hypothetical, unclassified, unknown | Unknown [Class 3] |
| PA14_33220 | PA14_33230 | hypothetical protein | Hypothetical, unclassified, unknown | Unknown [Class 3] |
| PA14_33230 | PA14_33240 | hypothetical protein | Hypothetical, unclassified, unknown | Cytoplasmic [Class 3] |
| PA14_33240 | PA14_33250 | hypothetical protein | Hypothetical, unclassified, unknown | Unknown [Class 3] |
| PA14_33250 | PA14_33300 | hypothetical protein | Hypothetical, unclassified, unknown | Unknown [Class 3] |
| PA14_33300 | PA14_33580 | hypothetical protein | Hypothetical, unclassified, unknown | Unknown [Class 3] |
| PA14_33580 | PA14_33590 | hypothetical protein | Hypothetical, unclassified, unknown | Cytoplasmic Membrane [Class 3] |
| PA14_33590 | PA14_33930 | hypothetical protein | Hypothetical, unclassified, unknown | Cytoplasmic Membrane [Class 3] |
| PA14_33930 | PA14_34070 | hypothetical protein | Hypothetical, unclassified, unknown | Cytoplasmic [Class 3] |
| PA14_34070 | PA14_34110 | hypothetical protein | Hypothetical, unclassified, unknown | Unknown [Class 3] |
| PA14_34110 | PA14_34140 | hypothetical protein | Hypothetical, unclassified, unknown | Cytoplasmic [Class 3] |
| PA14_34140 | PA14_34170 | hypothetical protein | Hypothetical, unclassified, unknown | Unknown [Class 3] |
| PA14_34170 | PA14_34720 | hypothetical protein | Hypothetical, unclassified, unknown | Unknown [Class 3] |
| PA14_34720 | PA14_34740 | hypothetical protein | Hypothetical, unclassified, unknown | Unknown [Class 3] |
| PA14_34740 | PA14_34940 | hypothetical protein | Hypothetical, unclassified, unknown | Unknown [Class 3] |
| PA14_34940 | PA14_35010 | hypothetical protein | Hypothetical, unclassified, unknown | Unknown [Class 3] |
| PA14_35010 | PA14_35030 | hypothetical protein | Hypothetical, unclassified, unknown | Unknown [Class 3] |
| PA14_35030 | PA14_35160 | hypothetical protein | Hypothetical, unclassified, unknown | Unknown [Class 3] |
| PA14_35160 | PA14_35760 | hypothetical protein | Hypothetical, unclassified, unknown | Cytoplasmic [Class 3] |
| PA14_35760 | PA14_35770 | hypothetical protein | Hypothetical, unclassified, unknown | Cytoplasmic Membrane [Class 3] |
| PA14_35770 | PA14_35930 | Hypothetical protein | Hypothetical, unclassified, unknown | Unknown [Class 3] |
| PA14_35930 | PA14_36010 | hypothetical protein | Hypothetical, unclassified, unknown | Unknown [Class 3] |
| PA14_36010 | PA14_36150 | hypothetical protein | Hypothetical, unclassified, unknown | Cytoplasmic Membrane [Class 3] |
| PA14_36020 | PA14_36190 | hypothetical protein | Hypothetical, unclassified, unknown | Unknown [Class 3] |
| PA14_36150 | PA14_36250 | hypothetical protein | Hypothetical, unclassified, unknown | Unknown [Class 3] |
| PA14_36190 | PA14_36350 | hypothetical protein | Hypothetical, unclassified, unknown | Cytoplasmic [Class 3] |
| PA14_36250 | PA14_36375 | hypothetical protein | Hypothetical, unclassified, unknown | Cytoplasmic [Class 3] |
| PA14_36350 | PA14_36450 | hypothetical protein | Hypothetical, unclassified, unknown | Unknown [Class 3] |
| PA14_36375 | PA14_36480 | hypothetical protein | Hypothetical, unclassified, unknown | Unknown [Class 3] |
| PA14_36450 | PA14_36490 | hypothetical protein | Hypothetical, unclassified, unknown | Unknown [Class 3] |
| PA14_36480 | PA14_36520 | hypothetical protein | Hypothetical, unclassified, unknown | Cytoplasmic [Class 3] |
| PA14_36490 | PA14_36620 | hypothetical protein | Hypothetical, unclassified, unknown | Unknown [Class 3] |
| PA14_36520 | PA14_36650 | hypothetical protein | Hypothetical, unclassified, unknown | Unknown [Class 3] |
| PA14_36620 | PA14_36770 | hypothetical protein | Hypothetical, unclassified, unknown | Unknown [Class 3] |
| PA14_36650 | PA14_36820 | hypothetical protein | Hypothetical, unclassified, unknown | Unknown [Class 3] |
| PA14_36770 | PA14_36850 | hypothetical protein | Hypothetical, unclassified, unknown | Unknown [Class 3] |
| PA14_36820 | PA14_36860 | hypothetical protein | Hypothetical, unclassified, unknown | Unknown [Class 3] |
| PA14_36850 | PA14_36900 | hypothetical protein | Hypothetical, unclassified, unknown | Cytoplasmic Membrane [Class 3] |
| PA14_36860 | PA14_36930 | hypothetical protein | Hypothetical, unclassified, unknown | Unknown [Class 3] |
| PA14_36900 | PA14_37150 | hypothetical protein | Hypothetical, unclassified, unknown | Cytoplasmic [Class 3] |
| PA14_36930 | PA14_37210 | hypothetical protein | Hypothetical, unclassified, unknown | Cytoplasmic [Class 3] |
| PA14_37150 | PA14_37410 | hypothetical protein | Hypothetical, unclassified, unknown | Cytoplasmic [Class 3] |
| PA14_37210 | PA14_37820 | hypothetical protein | Hypothetical, unclassified, unknown | Unknown [Class 3] |
| PA14_37410 | PA14_38000 | hypothetical protein | Hypothetical, unclassified, unknown | Unknown [Class 3] |
| PA14_37820 | PA14_38050 | hypothetical protein | Hypothetical, unclassified, unknown | Unknown [Class 3] |
| PA14_38000 | PA14_38060 | hypothetical protein | Hypothetical, unclassified, unknown | Unknown [Class 3] |
| PA14_38050 | PA14_38190 | hypothetical protein | Hypothetical, unclassified, unknown | Cytoplasmic [Class 3] |
| PA14_38060 | PA14_38290 | hypothetical protein | Hypothetical, unclassified, unknown | Cytoplasmic [Class 3] |
| PA14_38190 | PA14_38310 | hypothetical protein | Hypothetical, unclassified, unknown | Unknown [Class 3] |
| PA14_38290 | PA14_38370 | hypothetical protein | Hypothetical, unclassified, unknown | Cytoplasmic [Class 3] |
| PA14_38310 | PA14_38710 | hypothetical protein | Hypothetical, unclassified, unknown | Unknown [Class 3] |
| PA14_38370 | PA14_38720 | hypothetical protein | Hypothetical, unclassified, unknown | Cytoplasmic [Class 3] |
| PA14_38710 | PA14_38880 | hypothetical protein | Hypothetical, unclassified, unknown | Extracellular [Class 3] |
| PA14_38720 | PA14_38920 | hypothetical protein | Hypothetical, unclassified, unknown | Cytoplasmic Membrane [Class 3] |
| PA14_38880 | PA14_39070 | hypothetical protein | Hypothetical, unclassified, unknown | Unknown [Class 3] |
| PA14_38920 | PA14_39080 | hypothetical protein | Hypothetical, unclassified, unknown | Unknown [Class 3] |
| PA14_39070 | PA14_39090 | hypothetical protein | Hypothetical, unclassified, unknown | Unknown [Class 3] |
| PA14_39080 | PA14_39220 | hypothetical protein | Hypothetical, unclassified, unknown | Extracellular [Class 3] |
| PA14_39090 | PA14_39240 | hypothetical protein | Hypothetical, unclassified, unknown | Unknown [Class 3] |
| PA14_39220 | PA14_39500 | hypothetical protein | Hypothetical, unclassified, unknown | Periplasmic [Class 3] |
| PA14_39240 | PA14_39630 | hypothetical protein | Hypothetical, unclassified, unknown | Cytoplasmic Membrane [Class 3] |
| PA14_39500 | PA14_39670 | hypothetical protein | Hypothetical, unclassified, unknown | Unknown [Class 3] |
| PA14_39630 | PA14_40010 | hypothetical protein | Hypothetical, unclassified, unknown | Cytoplasmic [Class 3] |
| PA14_39670 | PA14_40050 | hypothetical protein | Hypothetical, unclassified, unknown | Unknown [Class 3] |
| PA14_40010 | PA14_40110 | hypothetical protein | Hypothetical, unclassified, unknown | Unknown [Class 3] |
| PA14_40050 | PA14_40280 | hypothetical protein | Hypothetical, unclassified, unknown | Unknown [Class 3] |
| PA14_40110 | PA14_40330 | hypothetical protein | Hypothetical, unclassified, unknown | Cytoplasmic Membrane [Class 3] |
| PA14_40280 | PA14_40560 | hypothetical protein | Hypothetical, unclassified, unknown | Unknown [Class 3] |
| PA14_40330 | PA14_40610 | hypothetical protein | Hypothetical, unclassified, unknown | Unknown [Class 3] |
| PA14_40560 | PA14_40650 | hypothetical protein | Hypothetical, unclassified, unknown | Unknown [Class 3] |
| PA14_40610 | PA14_40700 | hypothetical protein | Hypothetical, unclassified, unknown | Cytoplasmic [Class 3] |
| PA14_40650 | PA14_40710 | hypothetical protein | Hypothetical, unclassified, unknown | Unknown [Class 3] |
| PA14_40700 | PA14_40750 | hypothetical protein | Hypothetical, unclassified, unknown | Cytoplasmic Membrane [Class 3] |
| PA14_40710 | PA14_40780 | hypothetical protein | Hypothetical, unclassified, unknown | Cytoplasmic [Class 3] |
| PA14_40750 | PA14_40930 | hypothetical protein | Hypothetical, unclassified, unknown | Cytoplasmic Membrane [Class 3] |
| PA14_40780 | PA14_41300 | hypothetical protein | Hypothetical, unclassified, unknown | Unknown [Class 3] |
| PA14_40930 | PA14_41730 | hypothetical protein | Hypothetical, unclassified, unknown | Cytoplasmic [Class 3] |
| PA14_41300 | PA14_41960 | hypothetical protein | Hypothetical, unclassified, unknown | Unknown [Class 3] |
| PA14_41730 | PA14_42000 | hypothetical protein | Hypothetical, unclassified, unknown | Unknown [Class 3] |
| PA14_41960 | PA14_42150 | hypothetical protein | Hypothetical, unclassified, unknown | Cytoplasmic [Class 3] |
| PA14_42000 | PA14_42410 | hypothetical protein | Hypothetical, unclassified, unknown | Unknown [Class 3] |
| PA14_42150 | PA14_42710 | hypothetical protein | Hypothetical, unclassified, unknown | Cytoplasmic Membrane [Class 3] |
| PA14_42410 | PA14_42830 | hypothetical protein | Hypothetical, unclassified, unknown | Cytoplasmic Membrane [Class 3] |
| PA14_42710 | PA14_42840 | hypothetical protein | Hypothetical, unclassified, unknown | Cytoplasmic [Class 3] |
| PA14_42830 | PA14_42950 | hypothetical protein | Hypothetical, unclassified, unknown | Cytoplasmic [Class 3] |
| PA14_42840 | PA14_43040 | hypothetical protein | Hypothetical, unclassified, unknown | Cytoplasmic [Class 3] |
| PA14_42950 | PA14_43230 | hypothetical protein | Hypothetical, unclassified, unknown | Unknown [Class 3] |
| PA14_43040 | PA14_43310 | hypothetical protein | Hypothetical, unclassified, unknown | Unknown [Class 3] |
| PA14_43230 | PA14_43520 | hypothetical protein | Hypothetical, unclassified, unknown | Unknown [Class 3] |
| PA14_43310 | PA14_44170 | hypothetical protein | Hypothetical, unclassified, unknown | Unknown [Class 3] |
| PA14_43520 | PA14_44430 | hypothetical protein | Hypothetical, unclassified, unknown | Unknown [Class 3] |
| PA14_44170 | PA14_44480 | hypothetical protein | Hypothetical, unclassified, unknown | Unknown [Class 3] |
| PA14_44430 | PA14_44620 | hypothetical protein | Hypothetical, unclassified, unknown | Unknown [Class 3] |
| PA14_44480 | PA14_44640 | hypothetical protein | Hypothetical, unclassified, unknown | Unknown [Class 3] |
| PA14_44620 | PA14_44930 | hypothetical protein | Hypothetical, unclassified, unknown | Unknown [Class 3] |
| PA14_44640 | PA14_45400 | hypothetical protein | Hypothetical, unclassified, unknown | Unknown [Class 3] |
| PA14_44760 | PA14_46160 | hypothetical protein | Hypothetical, unclassified, unknown | Unknown [Class 3] |
| PA14_44930 | PA14_46340 | hypothetical protein | Hypothetical, unclassified, unknown | Unknown [Class 3] |
| PA14_45400 | PA14_46390 | hypothetical protein | Hypothetical, unclassified, unknown | Unknown [Class 3] |
| PA14_46160 | PA14_46430 | hypothetical protein | Hypothetical, unclassified, unknown | Unknown [Class 3] |
| PA14_46340 | PA14_46740 | hypothetical protein | Hypothetical, unclassified, unknown | Cytoplasmic [Class 3] |
| PA14_46390 | PA14_46770 | hypothetical protein | Hypothetical, unclassified, unknown | Cytoplasmic [Class 3] |
| PA14_46430 | PA14_46780 | hypothetical protein | Hypothetical, unclassified, unknown | Unknown [Class 3] |
| PA14_46740 | PA14_46830 | hypothetical protein | Hypothetical, unclassified, unknown | Unknown [Class 3] |
| PA14_46770 | PA14_47350 | hypothetical protein | Hypothetical, unclassified, unknown | Cytoplasmic Membrane [Class 3] |
| PA14_46780 | PA14_47420 | hypothetical protein | Hypothetical, unclassified, unknown | Cytoplasmic [Class 3] |
| PA14_46830 | PA14_48230 | hypothetical protein | Hypothetical, unclassified, unknown | Unknown [Class 3] |
| PA14_47350 | PA14_48320 | hypothetical protein | Hypothetical, unclassified, unknown | Cytoplasmic [Class 3] |
| PA14_47420 | PA14_48400 | hypothetical protein | Hypothetical, unclassified, unknown | Unknown [Class 3] |
| PA14_48230 | PA14_48410 | hypothetical protein | Hypothetical, unclassified, unknown | Unknown [Class 3] |
| PA14_48320 | PA14_48510 | hypothetical protein | Hypothetical, unclassified, unknown | Unknown [Class 3] |
| PA14_48400 | PA14_48860 | hypothetical protein | Hypothetical, unclassified, unknown | Cytoplasmic [Class 3] |
| PA14_48410 | PA14_49090 | hypothetical protein | Hypothetical, unclassified, unknown | Cytoplasmic [Class 3] |
| PA14_48510 | PA14_49290 | hypothetical protein | Hypothetical, unclassified, unknown | Cytoplasmic Membrane [Class 3] |
| PA14_48860 | PA14_49310 | hypothetical protein | Hypothetical, unclassified, unknown | Unknown [Class 3] |
| PA14_48920 | PA14_49330 | hypothetical protein | Hypothetical, unclassified, unknown | Unknown [Class 3] |
| PA14_49090 | PA14_49500 | hypothetical protein | Hypothetical, unclassified, unknown | Unknown [Class 3] |
| PA14_49290 | PA14_49720 | hypothetical protein | Hypothetical, unclassified, unknown | Unknown [Class 3] |
| PA14_49310 | PA14_49730 | hypothetical protein | Hypothetical, unclassified, unknown | Unknown [Class 3] |
| PA14_49330 | PA14_49860 | hypothetical protein | Hypothetical, unclassified, unknown | Unknown [Class 3] |
| PA14_49500 | PA14_49920 | hypothetical protein | Hypothetical, unclassified, unknown | Unknown [Class 3] |
| PA14_49720 | PA14_50000 | hypothetical protein | Hypothetical, unclassified, unknown | Unknown [Class 3] |
| PA14_49730 | PA14_50030 | hypothetical protein | Hypothetical, unclassified, unknown | Cytoplasmic [Class 3] |
| PA14_49860 | PA14_50320 | hypothetical protein | Hypothetical, unclassified, unknown | Cytoplasmic [Class 3] |
| PA14_49920 | PA14_50500 | hypothetical protein | Hypothetical, unclassified, unknown | Cytoplasmic [Class 3] |
| PA14_50000 | PA14_50620 | hypothetical protein | Hypothetical, unclassified, unknown | Unknown [Class 3] |
| PA14_50030 | PA14_50870 | hypothetical protein | Hypothetical, unclassified, unknown | Cytoplasmic Membrane [Class 3] |
| PA14_50320 | PA14_50890 | hypothetical protein | Hypothetical, unclassified, unknown | Unknown [Class 3] |
| PA14_50500 | PA14_50910 | hypothetical protein | Hypothetical, unclassified, unknown | Unknown [Class 3] |
| PA14_50620 | PA14_51590 | hypothetical protein | Hypothetical, unclassified, unknown | Cytoplasmic Membrane [Class 3] |
| PA14_50870 | PA14_51850 | hypothetical protein | Hypothetical, unclassified, unknown | Unknown [Class 3] |
| PA14_50890 | PA14_52290 | hypothetical protein | Hypothetical, unclassified, unknown | Unknown [Class 3] |
| PA14_50910 | PA14_52440 | hypothetical protein | Hypothetical, unclassified, unknown | Unknown [Class 3] |
| PA14_51190 | PA14_52490 | hypothetical protein | Hypothetical, unclassified, unknown | Unknown [Class 3] |
| PA14_51590 | PA14_52640 | hypothetical protein | Hypothetical, unclassified, unknown | Cytoplasmic [Class 3] |
| PA14_51850 | PA14_52730 | hypothetical protein | Hypothetical, unclassified, unknown | Unknown [Class 3] |
| PA14_52290 | PA14_52910 | hypothetical protein | Hypothetical, unclassified, unknown | Cytoplasmic [Class 3] |
| PA14_52440 | PA14_53160 | hypothetical protein | Hypothetical, unclassified, unknown | Unknown [Class 3] |
| PA14_52490 | PA14_53680 | hypothetical protein | Hypothetical, unclassified, unknown | Unknown [Class 3] |
| PA14_52640 | PA14_53690 | hypothetical protein | Hypothetical, unclassified, unknown | Unknown [Class 3] |
| PA14_52730 | PA14_53810 | hypothetical protein | Hypothetical, unclassified, unknown | Cytoplasmic [Class 3] |
| PA14_52910 | PA14_53870 | hypothetical protein | Hypothetical, unclassified, unknown | Cytoplasmic Membrane [Class 3] |
| PA14_53160 | PA14_54230 | hypothetical protein | Hypothetical, unclassified, unknown | Unknown [Class 3] |
| PA14_53680 | PA14_54550 | hypothetical protein | Hypothetical, unclassified, unknown | Cytoplasmic Membrane [Class 3] |
| PA14_53690 | PA14_54680 | hypothetical protein | Hypothetical, unclassified, unknown | Cytoplasmic Membrane [Class 3] |
| PA14_53810 | PA14_54720 | hypothetical protein | Hypothetical, unclassified, unknown | Unknown [Class 3] |
| PA14_53870 | PA14_54730 | hypothetical protein | Hypothetical, unclassified, unknown | Cytoplasmic Membrane [Class 3] |
| PA14_54230 | PA14_54750 | hypothetical protein | Hypothetical, unclassified, unknown | Unknown [Class 3] |
| PA14_54550 | PA14_55240 | hypothetical protein | Hypothetical, unclassified, unknown | Unknown [Class 3] |
| PA14_54680 | PA14_55260 | hypothetical protein | Hypothetical, unclassified, unknown | Unknown [Class 3] |
| PA14_54720 | PA14_55840 | hypothetical protein | Hypothetical, unclassified, unknown | Unknown [Class 3] |
| PA14_54730 | PA14_56180 | hypothetical protein | Hypothetical, unclassified, unknown | Unknown [Class 3] |
| PA14_54750 | PA14_57040 | hypothetical protein | Hypothetical, unclassified, unknown | Cytoplasmic [Class 3] |
| PA14_55240 | PA14_57250 | hypothetical protein | Hypothetical, unclassified, unknown | Unknown [Class 3] |
| PA14_55260 | PA14_57820 | hypothetical protein | Hypothetical, unclassified, unknown | Unknown [Class 3] |
| PA14_55840 | PA14_57920 | hypothetical protein | Hypothetical, unclassified, unknown | Unknown [Class 3] |
| PA14_56180 | PA14_58800 | hypothetical protein | Hypothetical, unclassified, unknown | Unknown [Class 3] |
| PA14_57040 | PA14_58820 | hypothetical protein | Hypothetical, unclassified, unknown | Unknown [Class 3] |
| PA14_57130 | PA14_58850 | hypothetical protein | Hypothetical, unclassified, unknown | Cytoplasmic [Class 3] |
| PA14_57250 | PA14_58860 | hypothetical protein | Hypothetical, unclassified, unknown | Cytoplasmic Membrane [Class 3] |
| PA14_57820 | PA14_59510 | hypothetical protein | Hypothetical, unclassified, unknown | Unknown [Class 3] |
| PA14_57920 | PA14_59670 | hypothetical protein | Hypothetical, unclassified, unknown | Unknown [Class 3] |
| PA14_58800 | PA14_59870 | hypothetical protein | Hypothetical, unclassified, unknown | Cytoplasmic Membrane [Class 3] |
| PA14_58820 | PA14_59890 | hypothetical protein | Hypothetical, unclassified, unknown | Cytoplasmic Membrane [Class 3] |
| PA14_58850 | PA14_60010 | hypothetical protein | Hypothetical, unclassified, unknown | Cytoplasmic Membrane [Class 3] |
| PA14_58860 | PA14_60090 | hypothetical protein | Hypothetical, unclassified, unknown | Unknown [Class 3] |
| PA14_59510 | PA14_60200 | hypothetical protein | Hypothetical, unclassified, unknown | Unknown [Class 3] |
| PA14_59670 | PA14_60520 | hypothetical protein | Hypothetical, unclassified, unknown | Cytoplasmic [Class 3] |
| PA14_59870 | PA14_60540 | hypothetical protein | Hypothetical, unclassified, unknown | Unknown [Class 3] |
| PA14_59890 | PA14_61000 | hypothetical protein | Hypothetical, unclassified, unknown | Cytoplasmic Membrane [Class 3] |
| PA14_60010 | PA14_61340 | hypothetical protein | Hypothetical, unclassified, unknown | Unknown [Class 3] |
| PA14_60090 | PA14_61350 | hypothetical protein | Hypothetical, unclassified, unknown | Unknown [Class 3] |
| PA14_60200 | PA14_61430 | hypothetical protein | Hypothetical, unclassified, unknown | Cytoplasmic [Class 3] |
| PA14_60520 | PA14_61450 | hypothetical protein | Hypothetical, unclassified, unknown | Unknown [Class 3] |
| PA14_60540 | PA14_61890 | hypothetical protein | Hypothetical, unclassified, unknown | Unknown [Class 3] |
| PA14_61000 | PA14_61910 | hypothetical protein | Hypothetical, unclassified, unknown | Unknown [Class 3] |
| PA14_61340 | PA14_61980 | hypothetical protein | Hypothetical, unclassified, unknown | Cytoplasmic [Class 3] |
| PA14_61350 | PA14_62190 | hypothetical protein | Hypothetical, unclassified, unknown | Extracellular [Class 3] |
| PA14_61430 | PA14_62240 | hypothetical protein | Hypothetical, unclassified, unknown | Cytoplasmic [Class 3] |
| PA14_61450 | PA14_62670 | hypothetical protein | Hypothetical, unclassified, unknown | Unknown [Class 3] |
| PA14_61890 | PA14_62890 | hypothetical protein | Hypothetical, unclassified, unknown | Cytoplasmic Membrane [Class 3] |
| PA14_61910 | PA14_63220 | hypothetical protein | Hypothetical, unclassified, unknown | Unknown [Class 3] |
| PA14_61980 | PA14_63300 | hypothetical protein | Hypothetical, unclassified, unknown | Cytoplasmic [Class 3] |
| PA14_62190 | PA14_63320 | hypothetical protein | Hypothetical, unclassified, unknown | Unknown [Class 3] |
| PA14_62240 | PA14_63430 | Hypothetical protein | Hypothetical, unclassified, unknown | Unknown [Class 3] |
| PA14_62670 | PA14_63660 | hypothetical protein | Hypothetical, unclassified, unknown | Cytoplasmic [Class 3] |
| PA14_62890 | PA14_63680 | hypothetical protein | Hypothetical, unclassified, unknown | Cytoplasmic [Class 3] |
| PA14_63220 | PA14_63740 | hypothetical protein | Hypothetical, unclassified, unknown | Unknown [Class 3] |
| PA14_63300 | PA14_63770 | hypothetical protein | Hypothetical, unclassified, unknown | Unknown [Class 3] |
| PA14_63320 | PA14_63820 | hypothetical protein | Hypothetical, unclassified, unknown | Unknown [Class 3] |
| PA14_63430 | PA14_63940 | hypothetical protein | Hypothetical, unclassified, unknown | Cytoplasmic [Class 3] |
| PA14_63660 | PA14_64260 | hypothetical protein | Hypothetical, unclassified, unknown | Unknown [Class 3] |
| PA14_63680 | PA14_64530 | hypothetical protein | Hypothetical, unclassified, unknown | Unknown [Class 3] |
| PA14_63740 | PA14_64560 | hypothetical protein | Hypothetical, unclassified, unknown | Periplasmic [Class 3] |
| PA14_63770 | PA14_65590 | hypothetical protein | Hypothetical, unclassified, unknown | Unknown [Class 3] |
| PA14_63820 | PA14_65700 | hypothetical protein | Hypothetical, unclassified, unknown | Unknown [Class 3] |
| PA14_63940 | PA14_66190 | hypothetical protein | Hypothetical, unclassified, unknown | Cytoplasmic [Class 3] |
| PA14_64260 | PA14_66890 | hypothetical protein | Hypothetical, unclassified, unknown | Unknown [Class 3] |
| PA14_64530 | PA14_66910 | hypothetical protein | Hypothetical, unclassified, unknown | Unknown [Class 3] |
| PA14_64560 | PA14_67200 | hypothetical protein | Hypothetical, unclassified, unknown | Unknown [Class 3] |
| PA14_65590 | PA14_67620 | hypothetical protein | Hypothetical, unclassified, unknown | Unknown [Class 3] |
| PA14_65700 | PA14_67940 | hypothetical protein | Hypothetical, unclassified, unknown | Unknown [Class 3] |
| PA14_66190 | PA14_67960 | hypothetical protein | Hypothetical, unclassified, unknown | Unknown [Class 3] |
| PA14_66890 | PA14_68840 | hypothetical protein | Hypothetical, unclassified, unknown | Unknown [Class 3] |
| PA14_66910 | PA14_69010 | hypothetical protein | Hypothetical, unclassified, unknown | Unknown [Class 3] |
| PA14_66990 | PA14_69030 | hypothetical protein | Hypothetical, unclassified, unknown | Cytoplasmic [Class 3] |
| PA14_67200 | PA14_69050 | hypothetical protein | Hypothetical, unclassified, unknown | Unknown [Class 3] |
| PA14_67620 | PA14_69280 | hypothetical protein | Hypothetical, unclassified, unknown | Cytoplasmic [Class 3] |
| PA14_67940 | PA14_69580 | hypothetical protein | Hypothetical, unclassified, unknown | Unknown [Class 3] |
| PA14_67960 | PA14_69600 | hypothetical protein | Hypothetical, unclassified, unknown | Unknown [Class 3] |
| PA14_68840 | PA14_69820 | hypothetical protein | Hypothetical, unclassified, unknown | Unknown [Class 3] |
| PA14_69010 | PA14_70220 | hypothetical protein | Hypothetical, unclassified, unknown | Unknown [Class 3] |
| PA14_69030 | PA14_71080 | hypothetical protein | Hypothetical, unclassified, unknown | Unknown [Class 3] |
| PA14_69050 | PA14_71190 | hypothetical protein | Hypothetical, unclassified, unknown | Unknown [Class 3] |
| PA14_69280 | PA14_71350 | hypothetical protein | Hypothetical, unclassified, unknown | Unknown [Class 3] |
| PA14_69580 | PA14_71360 | hypothetical protein | Hypothetical, unclassified, unknown | Unknown [Class 3] |
| PA14_69600 | PA14_71370 | hypothetical protein | Hypothetical, unclassified, unknown | Unknown [Class 3] |
| PA14_69820 | PA14_71380 | hypothetical protein | Hypothetical, unclassified, unknown | Unknown [Class 3] |
| PA14_70220 | PA14_71760 | hypothetical protein | Hypothetical, unclassified, unknown | Unknown [Class 3] |
| PA14_71080 | PA14_72060 | hypothetical protein | Hypothetical, unclassified, unknown | Unknown [Class 3] |
| PA14_71190 | PA14_72090 | hypothetical protein | Hypothetical, unclassified, unknown | Unknown [Class 3] |
| PA14_71350 | PA14_72150 | hypothetical protein | Hypothetical, unclassified, unknown | Unknown [Class 3] |
| PA14_71360 | PA14_72210 | hypothetical protein | Hypothetical, unclassified, unknown | Cytoplasmic [Class 3] |
| PA14_71370 | PA14_72350 | hypothetical protein | Hypothetical, unclassified, unknown | Unknown [Class 3] |
| PA14_71380 | PA14_72360 | hypothetical protein | Hypothetical, unclassified, unknown | Periplasmic [Class 3] |
| PA14_71760 | PA14_72520 | hypothetical protein | Hypothetical, unclassified, unknown | Cytoplasmic [Class 3] |
| PA14_72060 | PA14_72830 | hypothetical protein | Hypothetical, unclassified, unknown | Unknown [Class 3] |
| PA14_72090 | PA14_72920 | hypothetical protein | Hypothetical, unclassified, unknown | Cytoplasmic [Class 3] |
| PA14_72150 | PA14_72990 | hypothetical protein | Hypothetical, unclassified, unknown | Unknown [Class 3] |
| PA14_72210 | PA14_73100 | hypothetical protein | Hypothetical, unclassified, unknown | Unknown [Class 3] |
| PA14_72350 | pcr3 | hypothetical protein | Hypothetical, unclassified, unknown | T3SS [Class 3] ; Extracellular [Class 3] |
| PA14_72360 | pcr4 | hypothetical protein | Hypothetical, unclassified, unknown | Cytoplasmic [Class 3] |
| PA14_72520 | pslO | hypothetical protein | Hypothetical, unclassified, unknown | Cytoplasmic Membrane [Class 3] |
| PA14_72830 | ycgL | hypothetical protein | Hypothetical, unclassified, unknown | Cytoplasmic [Class 3] |
| PA14_72920 | ycgN | hypothetical protein | Hypothetical, unclassified, unknown | Cytoplasmic [Class 3] |
| PA14_72990 | yeaG | hypothetical protein | Hypothetical, unclassified, unknown | Cytoplasmic [Class 3] |
| PA14_73100 | yhhW | hypothetical protein | Hypothetical, unclassified, unknown | Cytoplasmic [Class 3] |
| PA14_42520 | yybH | hypothetical protein | Hypothetical, unclassified, unknown | Unknown [Class 3] |
| PA14_42510 | ppnK | inorganic polyphosphate/ATP-NAD kinase | Hypothetical, unclassified, unknown | Cytoplasmic [Class 3] |
| PA14_24500 | PA14_14220 | nucleoid-associated protein NdpA | Hypothetical, unclassified, unknown | Cytoplasmic [Class 3] |
| PA14_50720 | PA14_36020 | paraquat-inducible protein B | Hypothetical, unclassified, unknown | Outer Membrane [Class 3] |
| PA14_50680 | pelC | putative lipoprotein | Hypothetical, unclassified, unknown | Unknown [Class 3] |
| PA14_24220 | phaC | putative monovalent cation/H+ antiporter subunit C | Hypothetical, unclassified, unknown | Cytoplasmic Membrane [Class 3] |
| PA14_35550 | phaG | putative monovalent cation/H+ antiporter subunit G | Hypothetical, unclassified, unknown | Cytoplasmic Membrane [Class 3] |
| PA14_27630 | PA14_57130 | putative nucleotide-binding protein | Hypothetical, unclassified, unknown | Cytoplasmic [Class 3] |
| PA14_27640 | rebB1 | putative protein associated with synthesis and assembly of refractile inclusion bodies | Hypothetical, unclassified, unknown | Unknown [Class 3] |
| PA14_47450 | rebB2 | putative protein associated with synthesis and assembly of refractile inclusion bodies | Hypothetical, unclassified, unknown | Unknown [Class 3] |
| PA14_47410 | PA14_44760 | putative xanthine dehydrogenase accessory factor X | Hypothetical, unclassified, unknown | Cytoplasmic [Class 3] |
| PA14_50790 | PA14_12050 | rRNA large subunit methyltransferase | Hypothetical, unclassified, unknown | Cytoplasmic [Class 3] |
| PA14_10220 | ygiM | SH3 domain-containing protein | Hypothetical, unclassified, unknown | Unknown [Class 3] |
| PA14_48650 | PA14_51190 | thiolase | Hypothetical, unclassified, unknown | Unknown [Class 3] |
| PA14_47110 | PA14_00160 | TPR repeat-containing protein | Hypothetical, unclassified, unknown | Unknown [Class 3] |
| PA14_23900 | PA14_40540 | CBS domain-containing protein | Membrane proteins | Cytoplasmic Membrane [Class 3] |
| PA14_13450 | cvpA | hypothetical protein | Membrane proteins | Cytoplasmic Membrane [Class 3] |
| PA14_25450 | lolE | hypothetical protein | Membrane proteins | Cytoplasmic Membrane [Class 3] |
| PA14_09540 | mexG | hypothetical protein | Membrane proteins | Cytoplasmic Membrane [Class 3] |
| PA14_11270 | PA14_00550 | hypothetical protein | Membrane proteins | Cytoplasmic Membrane [Class 3] |
| PA14_00550 | PA14_03590 | hypothetical protein | Membrane proteins | Cytoplasmic Membrane [Class 3] |
| PA14_02980 | PA14_04330 | hypothetical protein | Membrane proteins | Cytoplasmic Membrane [Class 3] |
| PA14_03590 | PA14_07010 | hypothetical protein | Membrane proteins | Cytoplasmic Membrane [Class 3] |
| PA14_04330 | PA14_08470 | hypothetical protein | Membrane proteins | Cytoplasmic Membrane [Class 3] |
| PA14_07010 | PA14_08500 | hypothetical protein | Membrane proteins | Cytoplasmic [Class 3] |
| PA14_08470 | PA14_09370 | hypothetical protein | Membrane proteins | Cytoplasmic Membrane [Class 3] |
| PA14_08500 | PA14_14200 | hypothetical protein | Membrane proteins | Cytoplasmic Membrane [Class 3] |
| PA14_09370 | PA14_18310 | hypothetical protein | Membrane proteins | Cytoplasmic Membrane [Class 3] |
| PA14_14200 | PA14_18320 | hypothetical protein | Membrane proteins | Cytoplasmic Membrane [Class 3] |
| PA14_18310 | PA14_19390 | hypothetical protein | Membrane proteins | Cytoplasmic Membrane [Class 3] |
| PA14_18320 | PA14_25940 | hypothetical protein | Membrane proteins | Cytoplasmic Membrane [Class 3] |
| PA14_19390 | PA14_26340 | hypothetical protein | Membrane proteins | Cytoplasmic Membrane [Class 3] |
| PA14_20700 | PA14_29660 | hypothetical protein | Membrane proteins | Cytoplasmic Membrane [Class 3] |
| PA14_25940 | PA14_36700 | hypothetical protein | Membrane proteins | Cytoplasmic Membrane [Class 3] |
| PA14_26340 | PA14_36940 | hypothetical protein | Membrane proteins | Cytoplasmic Membrane [Class 3] |
| PA14_29660 | PA14_38950 | hypothetical protein | Membrane proteins | Cytoplasmic Membrane [Class 3] |
| PA14_36700 | PA14_39180 | hypothetical protein | Membrane proteins | Cytoplasmic Membrane [Class 3] |
| PA14_36940 | PA14_42100 | hypothetical protein | Membrane proteins | Cytoplasmic Membrane [Class 3] |
| PA14_38950 | PA14_42910 | hypothetical protein | Membrane proteins | Cytoplasmic [Class 3] |
| PA14_39180 | PA14_44460 | hypothetical protein | Membrane proteins | Cytoplasmic Membrane [Class 3] |
| PA14_40540 | PA14_48660 | hypothetical protein | Membrane proteins | Cytoplasmic Membrane [Class 3] |
| PA14_42100 | PA14_49900 | hypothetical protein | Membrane proteins | Outer Membrane [Class 3] |
| PA14_42910 | PA14_63720 | hypothetical protein | Membrane proteins | Cytoplasmic Membrane [Class 3] |
| PA14_44460 | PA14_65160 | hypothetical protein | Membrane proteins | Cytoplasmic Membrane [Class 3] |
| PA14_48660 | PA14_65260 | hypothetical protein | Membrane proteins | Cytoplasmic Membrane [Class 3] |
| PA14_49900 | PA14_72320 | hypothetical protein | Membrane proteins | Cytoplasmic Membrane [Class 3] |
| PA14_63720 | PA14_72790 | hypothetical protein | Membrane proteins | Cytoplasmic Membrane [Class 3] |
| PA14_64690 | tonB2 | hypothetical protein | Membrane proteins | Periplasmic [Class 3] |
| PA14_65160 | yedI | hypothetical protein | Membrane proteins | Cytoplasmic Membrane [Class 3] |
| PA14_65260 | yjgQ | hypothetical protein | Membrane proteins | Cytoplasmic Membrane [Class 3] |
| PA14_72320 | yjiJ | hypothetical protein | Membrane proteins | Cytoplasmic Membrane [Class 3] |
| PA14_72790 | ygdE | multidrug efflux system protein MdtI | Membrane proteins | Cytoplasmic Membrane [Class 3] |
| PA14_02490 | oprG | outer membrane protein OprG precursor | Membrane proteins | Outer Membrane [Class 3] |
| PA14_65580 | PA14_20700 | putative glycosyltransferase | Membrane proteins | Cytoplasmic [Class 3] |
| PA14_40490 | ybcI | putative membrane-bound metal-dependent hydrolase | Membrane proteins | Cytoplasmic Membrane [Class 3] |
| PA14_44530 | PA14_02980 | putative porin | Membrane proteins | Outer Membrane [Class 3] |
| PA14_14510 | fecR | putative transmembrane sensor | Membrane proteins | Unknown [Class 3] |
| PA14_46180 | PA14_64690 | putative transmembrane sensor | Membrane proteins | Periplasmic [Class 3] |
| PA14_11060 | cupB4 | chaperone CupB4 | Motility & Attachment | Periplasmic [Class 3] |
| PA14_11090 | wspD | CheW domain-containing protein | Motility & Attachment | Cytoplasmic [Class 3] |
| PA14_50430 | fliH | flagellar assembly protein H | Motility & Attachment | Cytoplasmic [Class 3] |
| PA14_50110 | flgG | flagellar basal body rod protein FlgG | Motility & Attachment | Flagellar [Class 3] ; Extracellular [Class 3] |
| PA14_45810 | fliL | flagellar basal body-associated protein FliL | Motility & Attachment | Cytoplasmic Membrane [Class 3] |
| PA14_45790 | fliP | flagellar biosynthesis protein FliP | Motility & Attachment | Flagellar [Class 3] ; Cytoplasmic Membrane [Class 3] |
| PA14_45770 | fliN | flagellar motor switch protein | Motility & Attachment | Cytoplasmic Membrane [Class 3] |
| PA14_33530 | PA14_45410 | FlhB domain-containing protein | Motility & Attachment | Unknown [Class 3] |
| PA14_45410 | PA14_33530 | hypothetical protein | Motility & Attachment | Unknown [Class 3] |
| PA14_59320 | wspE | putative chemotaxis sensor/effector fusion protein | Motility & Attachment | Cytoplasmic [Class 3] |
| PA14_16460 | cupB1 | putative fimbrial subunit CupB1 | Motility & Attachment | Fimbrial [Class 3] ; Extracellular [Class 3] |
| PA14_16470 | pilS2 | type IV B pilus protein | Motility & Attachment | Fimbrial [Class 3] ; Extracellular [Class 3] |
| PA14_07470 | PA14_08570 | 16S ribosomal RNA | Non-coding RNA gene | No localization assigned and computational localization prediction using PSORTB not performed [] |
| PA14_08570 | PA14_55631 | 23S ribosomal RNA | Non-coding RNA gene | No localization assigned and computational localization prediction using PSORTB not performed [] |
| PA14_08650 | PA14_62060 | 23S ribosomal RNA | Non-coding RNA gene | No localization assigned and computational localization prediction using PSORTB not performed [] |
| PA14_21800 | PA14_52550 | tRNA-Arg | Non-coding RNA gene | No localization assigned and computational localization prediction using PSORTB not performed [] |
| PA14_23490 | PA14_23490 | tRNA-Asn | Non-coding RNA gene | No localization assigned and computational localization prediction using PSORTB not performed [] |
| PA14_23580 | PA14_24130 | tRNA-Asp | Non-coding RNA gene | No localization assigned and computational localization prediction using PSORTB not performed [] |
| PA14_24130 | PA14_41200 | tRNA-Asp | Non-coding RNA gene | No localization assigned and computational localization prediction using PSORTB not performed [] |
| PA14_24870 | PA14_30720 | tRNA-Cys | Non-coding RNA gene | No localization assigned and computational localization prediction using PSORTB not performed [] |
| PA14_27190 | PA14_23580 | tRNA-Glu | Non-coding RNA gene | No localization assigned and computational localization prediction using PSORTB not performed [] |
| PA14_28190 | PA14_41330 | tRNA-His | Non-coding RNA gene | No localization assigned and computational localization prediction using PSORTB not performed [] |
| PA14_28740 | PA14_41320 | tRNA-Leu | Non-coding RNA gene | No localization assigned and computational localization prediction using PSORTB not performed [] |
| PA14_30720 | PA14_62800 | tRNA-Leu | Non-coding RNA gene | No localization assigned and computational localization prediction using PSORTB not performed [] |
| PA14_41200 | PA14_65210 | tRNA-Leu | Non-coding RNA gene | No localization assigned and computational localization prediction using PSORTB not performed [] |
| PA14_41320 | PA14_51660 | tRNA-Lys | Non-coding RNA gene | No localization assigned and computational localization prediction using PSORTB not performed [] |
| PA14_41330 | PA14_07470 | tRNA-Met | Non-coding RNA gene | No localization assigned and computational localization prediction using PSORTB not performed [] |
| PA14_51230 | PA14_61830 | tRNA-Met | Non-coding RNA gene | No localization assigned and computational localization prediction using PSORTB not performed [] |
| PA14_51660 | PA14_68030 | tRNA-Phe | Non-coding RNA gene | No localization assigned and computational localization prediction using PSORTB not performed [] |
| PA14_52550 | PA14_24870 | tRNA-Pro | Non-coding RNA gene | No localization assigned and computational localization prediction using PSORTB not performed [] |
| PA14_52560 | PA14_28740 | tRNA-Pro | Non-coding RNA gene | No localization assigned and computational localization prediction using PSORTB not performed [] |
| PA14_55631 | PA14_63460 | tRNA-Sec | Non-coding RNA gene | No localization assigned and computational localization prediction using PSORTB not performed [] |
| PA14_58710 | PA14_27190 | tRNA-Ser | Non-coding RNA gene | No localization assigned and computational localization prediction using PSORTB not performed [] |
| PA14_61830 | PA14_51230 | tRNA-Ser | Non-coding RNA gene | No localization assigned and computational localization prediction using PSORTB not performed [] |
| PA14_62060 | PA14_52560 | tRNA-Ser | Non-coding RNA gene | No localization assigned and computational localization prediction using PSORTB not performed [] |
| PA14_62800 | PA14_58710 | tRNA-Thr | Non-coding RNA gene | No localization assigned and computational localization prediction using PSORTB not performed [] |
| PA14_63460 | PA14_68150 | tRNA-Thr | Non-coding RNA gene | No localization assigned and computational localization prediction using PSORTB not performed [] |
| PA14_65210 | PA14_08650 | tRNA-Tyr | Non-coding RNA gene | No localization assigned and computational localization prediction using PSORTB not performed [] |
| PA14_68030 | PA14_21800 | tRNA-Val | Non-coding RNA gene | No localization assigned and computational localization prediction using PSORTB not performed [] |
| PA14_68150 | PA14_28190 | tRNA-Val | Non-coding RNA gene | No localization assigned and computational localization prediction using PSORTB not performed [] |
| PA14_07700 | purA | adenylosuccinate synthetase | Nucleotide biosynthesis and metabolism | Cytoplasmic [Class 3] |
| PA14_15680 | purH | bifunctional phosphoribosylaminoimidazolecarboxamide formyltransferase/IMP cyclohydrolase | Nucleotide biosynthesis and metabolism | Cytoplasmic [Class 3] |
| PA14_70260 | dut | deoxyuridine 5'-triphosphate nucleotidohydrolase | Nucleotide biosynthesis and metabolism | Unknown [Class 3] |
| PA14_70440 | apaH | diadenosine tetraphosphatase | Nucleotide biosynthesis and metabolism | Cytoplasmic [Class 3] |
| PA14_61460 | pyrD | dihydroorotate dehydrogenase 2 | Nucleotide biosynthesis and metabolism | Cytoplasmic Membrane [Class 3] |
| PA14_41430 | ygdP | dinucleoside polyphosphate hydrolase | Nucleotide biosynthesis and metabolism | Cytoplasmic [Class 3] |
| PA14_14820 | gmk | guanylate kinase | Nucleotide biosynthesis and metabolism | Cytoplasmic [Class 3] |
| PA14_00800 | cumB | hypothetical protein | Nucleotide biosynthesis and metabolism | Cytoplasmic [Class 3] |
| PA14_05050 | miaE | hypothetical protein | Nucleotide biosynthesis and metabolism | Cytoplasmic [Class 3] |
| PA14_32340 | PA14_00800 | hypothetical protein | Nucleotide biosynthesis and metabolism | Unknown [Class 3] |
| PA14_64930 | PA14_64930 | hypothetical protein | Nucleotide biosynthesis and metabolism | Unknown [Class 3] |
| PA14_65230 | hpt | hypoxanthine-guanine phosphoribosyltransferase | Nucleotide biosynthesis and metabolism | Cytoplasmic [Class 3] |
| PA14_71620 | ndk | nucleoside diphosphate kinase | Nucleotide biosynthesis and metabolism | Extracellular [Class 3] |
| PA14_64200 | pyrE | orotate phosphoribosyltransferase | Nucleotide biosynthesis and metabolism | Cytoplasmic [Class 3] |
| PA14_24640 | purE | phosphoribosylaminoimidazole carboxylase, catalytic subunit | Nucleotide biosynthesis and metabolism | Unknown [Class 3] |
| PA14_70370 | PA14_32340 | putative deaminase | Nucleotide biosynthesis and metabolism | Cytoplasmic [Class 3] |
| PA14_17080 | PA14_05050 | putative deoxyribonucleotide triphosphate pyrophosphatase | Nucleotide biosynthesis and metabolism | Cytoplasmic [Class 3] |
| PA14_25740 | tmk | thymidylate kinase | Nucleotide biosynthesis and metabolism | Cytoplasmic [Class 3] |
| PA14_44710 | pyrH | uridylate kinase | Nucleotide biosynthesis and metabolism | Cytoplasmic [Class 3] |
| PA14_04390 | xdhA | xanthine dehydrogenase | Nucleotide biosynthesis and metabolism | Cytoplasmic [Class 3] |
| PA14_55890 | xcpU | general secretion pathway outer membrane protein H precursor | Protein secretion/export apparatus | Cytoplasmic Membrane [Class 3] |
| PA14_20720 | xcpV | general secretion pathway protein I | Protein secretion/export apparatus | Cytoplasmic Membrane [Class 3] |
| PA14_04900 | xcpW | general secretion pathway protein J | Protein secretion/export apparatus | Cytoplasmic Membrane [Class 3] |
| PA14_29520 | xcpZ | general secretion pathway protein M | Protein secretion/export apparatus | Cytoplasmic Membrane [Class 3] |
| PA14_29540 | hxcV | HxcV putative pseudopilin | Protein secretion/export apparatus | Unknown [Class 3] |
| PA14_55520 | flgN | hypothetical protein | Protein secretion/export apparatus | Cytoplasmic [Class 3] |
| PA14_55500 | PA14_55860 | hypothetical protein | Protein secretion/export apparatus | Cytoplasmic Membrane [Class 3] |
| PA14_55480 | PA14_55880 | hypothetical protein | Protein secretion/export apparatus | Cytoplasmic Membrane [Class 3] |
| PA14_55460 | PA14_55940 | hypothetical protein | Protein secretion/export apparatus | Cytoplasmic Membrane [Class 3] |
| PA14_54350 | yqjC | hypothetical protein | Protein secretion/export apparatus | Unknown [Class 3] |
| PA14_01040 | secF | preprotein translocase subunit SecF | Protein secretion/export apparatus | Cytoplasmic Membrane [Class 3] |
| PA14_40240 | secY | preprotein translocase subunit SecY | Protein secretion/export apparatus | Cytoplasmic Membrane [Class 3] |
| PA14_55860 | PA14_40240 | putative ATP-binding/permease fusion ABC transporter | Protein secretion/export apparatus | Cytoplasmic Membrane [Class 3] |
| PA14_55880 | PA14_01040 | putative secretion protein | Protein secretion/export apparatus | Cytoplasmic [Class 3] |
| PA14_55940 | cpaF2 | putative type II secretion system protein | Protein secretion/export apparatus | Cytoplasmic [Class 3] |
| PA14_59860 | hplW | putative type II secretion system protein | Protein secretion/export apparatus | Unknown [Class 3] |
| PA14_42480 | hxcX | putative type II secretion system protein | Protein secretion/export apparatus | Cytoplasmic Membrane [Class 3] |
| PA14_42550 | PA14_59860 | putative type III effector Hop protein | Protein secretion/export apparatus | Unknown [Class 3] |
| PA14_42340 | pcrG | regulator in type III secretion | Protein secretion/export apparatus | Unknown [Class 3] |
| PA14_42310 | tatC | sec-independent protein translocase TatC | Protein secretion/export apparatus | Cytoplasmic Membrane [Class 3] |
| PA14_42280 | xcpP | secretion protein XcpP | Protein secretion/export apparatus | Cytoplasmic Membrane [Class 3] |
| PA14_42580 | lepB | signal peptidase I | Protein secretion/export apparatus | Cytoplasmic Membrane [Class 3] |
| PA14_42600 | ftsY | signal recognition particle receptor FtsY | Protein secretion/export apparatus | Cytoplasmic [Class 3] |
| PA14_42610 | pscO | translocation protein in type III secretion | Protein secretion/export apparatus | Cytoplasmic [Class 3] |
| PA14_42620 | pscP | translocation protein in type III secretion | Protein secretion/export apparatus | T3SS [Class 3] ; Extracellular [Class 3] |
| PA14_42640 | pscT | translocation protein in type III secretion | Protein secretion/export apparatus | Flagellar [Class 3] ; Cytoplasmic Membrane [Class 3] |
| PA14_14650 | hplU | type II secretion system protein | Protein secretion/export apparatus | Cytoplasmic Membrane [Class 3] |
| PA14_09050 | hxcU | type II secretion system protein | Protein secretion/export apparatus | Unknown [Class 3] |
| PA14_66980 | hxcZ | type II secretion system protein | Protein secretion/export apparatus | Unknown [Class 3] |
| PA14_23980 | pscD | type III export protein PscD | Protein secretion/export apparatus | Cytoplasmic [Class 3] |
| PA14_24040 | pscF | type III export protein PscF | Protein secretion/export apparatus | Extracellular [Class 3] |
| PA14_24050 | pscI | type III export protein PscI | Protein secretion/export apparatus | Unknown [Class 3] |
| PA14_24060 | popN | Type III secretion outer membrane protein PopN precursor | Protein secretion/export apparatus | Outer Membrane [Class 3] |
| PA14_24100 | pscQ | type III secretion system protein | Protein secretion/export apparatus | Unknown [Class 3] |
| PA14_07040 | pscR | type III secretion system protein | Protein secretion/export apparatus | Flagellar [Class 3] ; Cytoplasmic Membrane [Class 3] |
| PA14_70140 | PA14_72980 | G3E family GTPase | Putative enzymes | Cytoplasmic Membrane [Class 3] |
| PA14_10920 | PA14_35880 | gamma-aminobutyraldehyde dehydrogenase | Putative enzymes | No localization assigned and computational localization prediction using PSORTB not performed [Class 3] |
| PA14_45020 | dad | hypothetical protein | Putative enzymes | Unknown [Class 3] |
| PA14_65690 | icc | hypothetical protein | Putative enzymes | Cytoplasmic [Class 3] |
| PA14_24430 | moxR | hypothetical protein | Putative enzymes | Cytoplasmic [Class 3] |
| PA14_00770 | PA14_00770 | hypothetical protein | Putative enzymes | Cytoplasmic [Class 3] |
| PA14_04380 | PA14_04380 | hypothetical protein | Putative enzymes | Cytoplasmic [Class 3] |
| PA14_05160 | PA14_05160 | hypothetical protein | Putative enzymes | Cytoplasmic [Class 3] |
| PA14_06430 | PA14_06430 | hypothetical protein | Putative enzymes | Unknown [Class 3] |
| PA14_09580 | PA14_09580 | hypothetical protein | Putative enzymes | Cytoplasmic [Class 3] |
| PA14_09950 | PA14_12890 | hypothetical protein | Putative enzymes | Cytoplasmic [Class 3] |
| PA14_10130 | PA14_13330 | hypothetical protein | Putative enzymes | Extracellular [Class 3] |
| PA14_12890 | PA14_17550 | hypothetical protein | Putative enzymes | Cytoplasmic [Class 3] |
| PA14_13330 | PA14_28340 | hypothetical protein | Putative enzymes | Unknown [Class 3] |
| PA14_15620 | PA14_29000 | hypothetical protein | Putative enzymes | Unknown [Class 3] |
| PA14_17550 | PA14_29340 | hypothetical protein | Putative enzymes | Unknown [Class 3] |
| PA14_23950 | PA14_31660 | hypothetical protein | Putative enzymes | Cytoplasmic [Class 3] |
| PA14_28340 | PA14_31730 | hypothetical protein | Putative enzymes | Cytoplasmic Membrane [Class 3] |
| PA14_29000 | PA14_31850 | hypothetical protein | Putative enzymes | Cytoplasmic [Class 3] |
| PA14_29340 | PA14_36110 | hypothetical protein | Putative enzymes | Cytoplasmic [Class 3] |
| PA14_31640 | PA14_36540 | hypothetical protein | Putative enzymes | Cytoplasmic [Class 3] |
| PA14_31660 | PA14_40220 | hypothetical protein | Putative enzymes | Cytoplasmic [Class 3] |
| PA14_31730 | PA14_44320 | hypothetical protein | Putative enzymes | Cytoplasmic [Class 3] |
| PA14_31850 | PA14_46150 | hypothetical protein | Putative enzymes | Cytoplasmic [Class 3] |
| PA14_32600 | PA14_50760 | hypothetical protein | Putative enzymes | Cytoplasmic [Class 3] |
| PA14_35880 | PA14_58080 | hypothetical protein | Putative enzymes | Cytoplasmic [Class 3] |
| PA14_35900 | PA14_65630 | hypothetical protein | Putative enzymes | Unknown [Class 3] |
| PA14_35950 | PA14_65670 | hypothetical protein | Putative enzymes | Unknown [Class 3] |
| PA14_35980 | PA14_67820 | hypothetical protein | Putative enzymes | Cytoplasmic Membrane [Class 3] |
| PA14_36110 | PA14_72430 | hypothetical protein | Putative enzymes | Cytoplasmic [Class 3] |
| PA14_36540 | yycJ | hypothetical protein | Putative enzymes | Cytoplasmic [Class 3] |
| PA14_39250 | PA14_42820 | isochorismatase family hydrolase | Putative enzymes | Cytoplasmic [Class 3] |
| PA14_40180 | PA14_15620 | oxidoreductase | Putative enzymes | Cytoplasmic [Class 3] |
| PA14_40220 | PA14_23950 | oxidoreductase | Putative enzymes | Cytoplasmic [Class 3] |
| PA14_41400 | PA14_53230 | oxidoreductase | Putative enzymes | Cytoplasmic [Class 3] |
| PA14_42820 | PA14_47440 | putative 2-hydroxyacid dehydrogenase | Putative enzymes | Cytoplasmic [Class 3] |
| PA14_43180 | PA14_35980 | putative acyl-CoA dehydrogenase | Putative enzymes | Unknown [Class 3] |
| PA14_43630 | PA14_44590 | putative acyl-CoA dehydrogenase | Putative enzymes | Cytoplasmic [Class 3] |
| PA14_44320 | aldH | putative aldehyde dehydrogenase | Putative enzymes | Cytoplasmic [Class 3] |
| PA14_44590 | PA14_57570 | putative cytochrome c reductase, iron-sulfur subun | Putative enzymes | Cytoplasmic Membrane [Class 3] |
| PA14_46150 | PA14_35900 | Putative dehydrogenase | Putative enzymes | Unknown [Class 3] |
| PA14_46890 | PA14_35950 | Putative Dehydrogenase | Putative enzymes | Cytoplasmic [Class 3] |
| PA14_47060 | PA14_39250 | putative double-glycine peptidase | Putative enzymes | Unknown [Class 3] |
| PA14_47340 | PA14_47340 | putative HIT family protein | Putative enzymes | Cytoplasmic [Class 3] |
| PA14_47440 | PA14_48210 | putative hydrolase | Putative enzymes | Cytoplasmic Membrane [Class 3] |
| PA14_48210 | PA14_43630 | putative lipase | Putative enzymes | Cytoplasmic Membrane [Class 3] |
| PA14_50610 | PA14_63830 | putative N-hydroxyarylamine O-acetyltransferase | Putative enzymes | Cytoplasmic [Class 3] |
| PA14_50760 | glxR | putative oxidoreductase | Putative enzymes | Cytoplasmic [Class 3] |
| PA14_52890 | PA14_09950 | putative oxidoreductase | Putative enzymes | Unknown [Class 3] |
| PA14_53230 | PA14_40180 | putative oxidoreductase | Putative enzymes | Cytoplasmic [Class 3] |
| PA14_53700 | sixA | putative phosphohistidine phosphatase SixA | Putative enzymes | Unknown [Class 3] |
| PA14_57570 | PA14_31640 | putative ring-cleaving dioxygenase | Putative enzymes | Cytoplasmic [Class 3] |
| PA14_58080 | PA14_46890 | putative short-chain dehydrogenase | Putative enzymes | Unknown [Class 3] |
| PA14_63830 | PA14_63890 | putative short-chain dehydrogenase | Putative enzymes | Cytoplasmic [Class 3] |
| PA14_63890 | pslI | putative transferase | Putative enzymes | Cytoplasmic Membrane [Class 3] |
| PA14_65630 | PA14_52890 | ring-cleaving dioxygenase | Putative enzymes | Cytoplasmic [Class 3] |
| PA14_65670 | PA14_53700 | ring-cleaving dioxygenase | Putative enzymes | Unknown [Class 3] |
| PA14_67820 | PA14_10130 | short chain dehydrogenase | Putative enzymes | Cytoplasmic [Class 3] |
| PA14_72430 | PA14_43180 | short chain dehydrogenase | Putative enzymes | Unknown [Class 3] |
| PA14_72980 | PA14_47060 | short chain dehydrogenase | Putative enzymes | Cytoplasmic [Class 3] |
| PA14_35640 | PA14_50610 | short chain dehydrogenase | Putative enzymes | Cytoplasmic [Class 3] |
| PA14_43620 | ydgB | short chain dehydrogenase | Putative enzymes | Cytoplasmic [Class 3] |
| PA14_22780 | PA14_32600 | thiol:disulfide interchange protein | Putative enzymes | Cytoplasmic Membrane [Class 3] |
| PA14_19640 | PA14_41400 | UDP-2,3-diacylglucosamine hydrolase | Putative enzymes | Cytoplasmic [Class 3] |
| PA14_51250 | yciI | YciI-like protein | Putative enzymes | Unknown [Class 3] |
| PA14_08150 | traG | conjugal transfer coupling protein TraG | Related to phage, transposon, or plasmid | Cytoplasmic Membrane [Class 3] |
| PA14_08090 | hol | hypothetical protein | Related to phage, transposon, or plasmid | Cytoplasmic Membrane [Class 3] |
| PA14_65310 | PA14_03180 | hypothetical protein | Related to phage, transposon, or plasmid | Cytoplasmic [Class 3] |
| PA14_07990 | PA14_03300 | hypothetical protein | Related to phage, transposon, or plasmid | Unknown [Class 3] |
| PA14_03180 | PA14_31000 | hypothetical protein | Related to phage, transposon, or plasmid | Cytoplasmic Membrane [Class 3] |
| PA14_03300 | PA14_48960 | hypothetical protein | Related to phage, transposon, or plasmid | Cytoplasmic Membrane [Class 3] |
| PA14_08060 | PA14_49010 | hypothetical protein | Related to phage, transposon, or plasmid | Unknown [Class 3] |
| PA14_08260 | PA14_58970 | hypothetical protein | Related to phage, transposon, or plasmid | Cytoplasmic [Class 3] |
| PA14_13890 | PA14_63450 | hypothetical protein | Related to phage, transposon, or plasmid | Unknown [Class 3] |
| PA14_31000 | traK | oriT-binding protein, TraK | Related to phage, transposon, or plasmid | Unknown [Class 3] |
| PA14_31080 | PA14_31080 | putative conjugal transfer protein | Related to phage, transposon, or plasmid | Unknown [Class 3] |
| PA14_31100 | trbB | putative conjugal transfer protein | Related to phage, transposon, or plasmid | Cytoplasmic [Class 3] |
| PA14_48960 | PA14_13890 | putative integrase protein | Related to phage, transposon, or plasmid | Cytoplasmic [Class 3] |
| PA14_49010 | PA14_08260 | putative minor tail protein L | Related to phage, transposon, or plasmid | Unknown [Class 3] |
| PA14_51620 | gpD | putative phage late control gene D protein | Related to phage, transposon, or plasmid | Cytoplasmic [Class 3] |
| PA14_51630 | gpFII | putative phage tail tube protein | Related to phage, transposon, or plasmid | Unknown [Class 3] |
| PA14_58970 | PA14_31100 | putative plasmid partitioning protein | Related to phage, transposon, or plasmid | Cytoplasmic Membrane [Class 3] |
| PA14_59570 | tnpR | putative resolvase, essential for transposition | Related to phage, transposon, or plasmid | Cytoplasmic [Class 3] |
| PA14_63450 | PA14_08060 | putative tail fiber assembly protein | Related to phage, transposon, or plasmid | Unknown [Class 3] |
| PA14_15430 | tpnC | putative tpnA repressor protein | Related to phage, transposon, or plasmid | Cytoplasmic [Class 3] |
| PA14_35750 | PA14_59570 | putative transposase | Related to phage, transposon, or plasmid | Unknown [Class 3] |
| PA14_30960 | hfq | RNA-binding protein Hfq | Related to phage, transposon, or plasmid | Cytoplasmic [Class 3] |
| PA14_15500 | PA14_51620 | transposase | Related to phage, transposon, or plasmid | Unknown [Class 3] |
| PA14_30940 | PA14_51630 | transposase | Related to phage, transposon, or plasmid | Unknown [Class 3] |
| PA14_30930 | trbC | TrbC-like protein | Related to phage, transposon, or plasmid | Cytoplasmic Membrane [Class 3] |
| PA14_18430 | algJ | alginate o-acetyltransferase AlgJ | Secreted Factors (toxins, enzymes, alginate) | Cytoplasmic Membrane [Class 3] |
| PA14_43070 | PA14_56560 | hypothetical protein | Secreted Factors (toxins, enzymes, alginate) | Cytoplasmic [Class 3] |
| PA14_54410 | pyoS3I | immunity protein S3I structureal gene | Secreted Factors (toxins, enzymes, alginate) | Cytoplasmic [Class 3] |
| PA14_56560 | mucB | negative regulator for alginate biosynthesis MucB | Secreted Factors (toxins, enzymes, alginate) | Periplasmic [Class 3] |
| PA14_39970 | phzA2 | phenazine biosynthesis protein | Secreted Factors (toxins, enzymes, alginate) | Cytoplasmic [Class 3] |
| PA14_09470 | phzB1 | phenazine biosynthesis protein | Secreted Factors (toxins, enzymes, alginate) | Cytoplasmic [Class 3] |
| PA14_09440 | phzE1 | phenazine biosynthesis protein PhzE | Secreted Factors (toxins, enzymes, alginate) | Cytoplasmic [Class 3] |
| PA14_09410 | pvcC | pyoverdine biosynthesis protein PvcC | Secreted Factors (toxins, enzymes, alginate) | Cytoplasmic [Class 3] |
| PA14_39880 | phzG2 | pyridoxamine 5'-phosphate oxidase | Secreted Factors (toxins, enzymes, alginate) | Cytoplasmic [Class 3] |
| PA14_35400 | phzG1 | pyrodoxamine 5'-phosphate oxidase | Secreted Factors (toxins, enzymes, alginate) | Cytoplasmic [Class 3] |
| PA14_49510 | hcpD | secreted protein Hcp | Secreted Factors (toxins, enzymes, alginate) | Extracellular [Class 3] |
| PA14_27690 | rimM | 16S rRNA-processing protein RimM | Transcription, RNA processing and degradation | Cytoplasmic [Class 3] |
| PA14_64190 | ligT | 2'-5' RNA ligase | Transcription, RNA processing and degradation | Unknown [Class 3] |
| PA14_62900 | fis | DNA-binding protein Fis | Transcription, RNA processing and degradation | Cytoplasmic [Class 3] |
| PA14_27110 | rpoZ | DNA-directed RNA polymerase subunit omega | Transcription, RNA processing and degradation | Unknown [Class 3] |
| PA14_30150 | PA14_07970 | hypothetical protein | Transcription, RNA processing and degradation | Unknown [Class 3] |
| PA14_62770 | PA14_49060 | hypothetical protein | Transcription, RNA processing and degradation | Cytoplasmic [Class 3] |
| PA14_11450 | fecI | putative RNA polymerase sigma factor | Transcription, RNA processing and degradation | Cytoplasmic [Class 3] |
| PA14_07970 | sun | putative tRNA and rRNA cytosine-C5-methylases | Transcription, RNA processing and degradation | Cytoplasmic [Class 3] |
| PA14_49060 | rnc | ribonuclease III | Transcription, RNA processing and degradation | Cytoplasmic [Class 3] |
| PA14_15980 | rrmA | rRNA methyltransferase | Transcription, RNA processing and degradation | Cytoplasmic [Class 3] |
| PA14_54330 | nusB | transcription antitermination protein NusB | Transcription, RNA processing and degradation | Unknown [Class 3] |
| PA14_70450 | greA | transcription elongation factor GreA | Transcription, RNA processing and degradation | Cytoplasmic [Class 3] |
| PA14_49390 | nusA | transcription elongation factor NusA | Transcription, RNA processing and degradation | Cytoplasmic [Class 3] |
| PA14_00180 | trmD | tRNA (guanine-N(1)-)-methyltransferase | Transcription, RNA processing and degradation | Cytoplasmic [Class 3] |
| PA14_15990 | truB | tRNA pseudouridine synthase B | Transcription, RNA processing and degradation | Cytoplasmic [Class 3] |
| PA14_62730 | mnmA | tRNA-specific 2-thiouridylase MnmA | Transcription, RNA processing and degradation | Cytoplasmic [Class 3] |
| PA14_05290 | PA14_19800 | AraC family transcriptional regulator | Transcriptional regulators | Unknown [Class 3] |
| PA14_20290 | PA14_34450 | AraC family transcriptional regulator | Transcriptional regulators | Cytoplasmic [Class 3] |
| PA14_10800 | PA14_35140 | AraC family transcriptional regulator | Transcriptional regulators | Cytoplasmic [Class 3] |
| PA14_16670 | PA14_38040 | AraC family transcriptional regulator | Transcriptional regulators | Cytoplasmic [Class 3] |
| PA14_32200 | PA14_30770 | AsnC family transcriptional regulator | Transcriptional regulators | Cytoplasmic [Class 3] |
| PA14_63170 | PA14_58510 | AsnC family transcriptional regulator | Transcriptional regulators | Cytoplasmic [Class 3] |
| PA14_41870 | pyrR | bifunctional pyrimidine regulatory protein PyrR uracil phosphoribosyltransferase | Transcriptional regulators | Cytoplasmic [Class 3] |
| PA14_20730 | rsmA | carbon storage regulator | Transcriptional regulators | Unknown [Class 3] |
| PA14_38930 | algZ | DNA binding-protein | Transcriptional regulators | Cytoplasmic [Class 3] |
| PA14_38430 | PA14_32710 | ECF subfamily RNA polymerase sigma-70 factor | Transcriptional regulators | Cytoplasmic [Class 3] |
| PA14_37580 | PA14_39800 | ECF subfamily RNA polymerase sigma-70 factor | Transcriptional regulators | Cytoplasmic [Class 3] |
| PA14_15450 | PA14_44690 | GntR family transcriptional regulator | Transcriptional regulators | Cytoplasmic [Class 3] |
| PA14_15480 | algH | hypothetical protein | Transcriptional regulators | Unknown [Class 3] |
| PA14_60860 | flgM | hypothetical protein | Transcriptional regulators | Unknown [Class 3] |
| PA14_06690 | PA14_01480 | hypothetical protein | Transcriptional regulators | Cytoplasmic [Class 3] |
| PA14_01480 | PA14_01520 | hypothetical protein | Transcriptional regulators | Unknown [Class 3] |
| PA14_01500 | PA14_37080 | hypothetical protein | Transcriptional regulators | Cytoplasmic [Class 3] |
| PA14_01520 | PA14_40210 | hypothetical protein | Transcriptional regulators | Cytoplasmic [Class 3] |
| PA14_01840 | PA14_43720 | hypothetical protein | Transcriptional regulators | Cytoplasmic [Class 3] |
| PA14_02910 | PA14_02910 | IclR family transcriptional regulator | Transcriptional regulators | Cytoplasmic [Class 3] |
| PA14_06260 | PA14_06260 | LysR family transcriptional regulator | Transcriptional regulators | Cytoplasmic [Class 3] |
| PA14_08630 | PA14_10830 | LysR family transcriptional regulator | Transcriptional regulators | Cytoplasmic [Class 3] |
| PA14_10830 | PA14_15210 | LysR family transcriptional regulator | Transcriptional regulators | Cytoplasmic [Class 3] |
| PA14_11180 | PA14_15240 | LysR family transcriptional regulator | Transcriptional regulators | Cytoplasmic [Class 3] |
| PA14_14310 | PA14_16380 | LysR family transcriptional regulator | Transcriptional regulators | Cytoplasmic [Class 3] |
| PA14_14710 | PA14_18200 | LysR family transcriptional regulator | Transcriptional regulators | Cytoplasmic [Class 3] |
| PA14_15210 | PA14_19670 | LysR family transcriptional regulator | Transcriptional regulators | Cytoplasmic [Class 3] |
| PA14_15240 | PA14_31630 | LysR family transcriptional regulator | Transcriptional regulators | Cytoplasmic [Class 3] |
| PA14_16380 | PA14_31780 | LysR family transcriptional regulator | Transcriptional regulators | Cytoplasmic [Class 3] |
| PA14_18200 | PA14_33440 | LysR family transcriptional regulator | Transcriptional regulators | Cytoplasmic [Class 3] |
| PA14_19670 | PA14_36180 | LysR family transcriptional regulator | Transcriptional regulators | Cytoplasmic [Class 3] |
| PA14_19800 | PA14_40440 | LysR family transcriptional regulator | Transcriptional regulators | Cytoplasmic [Class 3] |
| PA14_21550 | PA14_47270 | LysR family transcriptional regulator | Transcriptional regulators | Cytoplasmic [Class 3] |
| PA14_21970 | PA14_68550 | LysR family transcriptional regulator | Transcriptional regulators | Cytoplasmic [Class 3] |
| PA14_22860 | phnR | LysR family transcriptional regulator | Transcriptional regulators | Cytoplasmic [Class 3] |
| PA14_23590 | PA14_27530 | MarR family transcriptional regulator | Transcriptional regulators | Cytoplasmic [Class 3] |
| PA14_26150 | PA14_43140 | MarR family transcriptional regulator | Transcriptional regulators | Unknown [Class 3] |
| PA14_26600 | PA14_61620 | MerR family transcriptional regulator | Transcriptional regulators | Cytoplasmic [Class 3] |
| PA14_27530 | PA14_08630 | pantothenate kinase | Transcriptional regulators | Cytoplasmic [Class 3] |
| PA14_27900 | lrp | putative leucine-responsive regulatory protein | Transcriptional regulators | Cytoplasmic [Class 3] |
| PA14_28080 | PA14_36880 | putative ompetence-damaged protein | Transcriptional regulators | Unknown [Class 3] |
| PA14_28730 | PA14_14710 | putative Rrf2 family protein | Transcriptional regulators | Unknown [Class 3] |
| PA14_29260 | cueR | putative transcriptional regulator | Transcriptional regulators | Cytoplasmic [Class 3] |
| PA14_30770 | glpR | putative transcriptional regulator | Transcriptional regulators | Cytoplasmic [Class 3] |
| PA14_31630 | nirG | putative transcriptional regulator | Transcriptional regulators | Cytoplasmic [Class 3] |
| PA14_31780 | PA14_01500 | putative transcriptional regulator | Transcriptional regulators | Unknown [Class 3] |
| PA14_32710 | PA14_11180 | putative transcriptional regulator | Transcriptional regulators | Cytoplasmic [Class 3] |
| PA14_32970 | PA14_14310 | putative transcriptional regulator | Transcriptional regulators | Unknown [Class 3] |
| PA14_33170 | PA14_22860 | putative transcriptional regulator | Transcriptional regulators | Cytoplasmic [Class 3] |
| PA14_33440 | PA14_23590 | putative transcriptional regulator | Transcriptional regulators | Cytoplasmic [Class 3] |
| PA14_33840 | PA14_26150 | putative transcriptional regulator | Transcriptional regulators | Cytoplasmic [Class 3] |
| PA14_33920 | PA14_27900 | putative transcriptional regulator | Transcriptional regulators | Cytoplasmic [Class 3] |
| PA14_34450 | PA14_28080 | putative transcriptional regulator | Transcriptional regulators | Unknown [Class 3] |
| PA14_34730 | PA14_28730 | putative transcriptional regulator | Transcriptional regulators | Cytoplasmic [Class 3] |
| PA14_35140 | PA14_29260 | putative transcriptional regulator | Transcriptional regulators | Cytoplasmic [Class 3] |
| PA14_35210 | PA14_33170 | putative transcriptional regulator | Transcriptional regulators | Cytoplasmic [Class 3] |
| PA14_36180 | PA14_33840 | putative transcriptional regulator | Transcriptional regulators | Cytoplasmic [Class 3] |
| PA14_36880 | PA14_33920 | putative transcriptional regulator | Transcriptional regulators | Cytoplasmic [Class 3] |
| PA14_37080 | PA14_40150 | putative transcriptional regulator | Transcriptional regulators | Unknown [Class 3] |
| PA14_37990 | PA14_40600 | putative transcriptional regulator | Transcriptional regulators | Cytoplasmic [Class 3] |
| PA14_38040 | PA14_42970 | putative transcriptional regulator | Transcriptional regulators | Cytoplasmic [Class 3] |
| PA14_39800 | PA14_45150 | putative transcriptional regulator | Transcriptional regulators | Cytoplasmic [Class 3] |
| PA14_40150 | PA14_45250 | putative transcriptional regulator | Transcriptional regulators | Cytoplasmic [Class 3] |
| PA14_40210 | PA14_47240 | putative transcriptional regulator | Transcriptional regulators | Cytoplasmic [Class 3] |
| PA14_40440 | PA14_47520 | putative transcriptional regulator | Transcriptional regulators | Cytoplasmic [Class 3] |
| PA14_40600 | PA14_47820 | putative transcriptional regulator | Transcriptional regulators | Cytoplasmic [Class 3] |
| PA14_42970 | PA14_48190 | putative transcriptional regulator | Transcriptional regulators | Cytoplasmic [Class 3] |
| PA14_43140 | PA14_48770 | putative transcriptional regulator | Transcriptional regulators | Cytoplasmic [Class 3] |
| PA14_43720 | PA14_63880 | putative transcriptional regulator | Transcriptional regulators | Cytoplasmic [Class 3] |
| PA14_44690 | PA14_67550 | putative transcriptional regulator | Transcriptional regulators | Cytoplasmic [Class 3] |
| PA14_45150 | PA14_71330 | putative transcriptional regulator | Transcriptional regulators | Cytoplasmic [Class 3] |
| PA14_45250 | pobR | putative transcriptional regulator | Transcriptional regulators | Cytoplasmic [Class 3] |
| PA14_46660 | sfnR | putative transcriptional regulator | Transcriptional regulators | Cytoplasmic [Class 3] |
| PA14_46810 | vanR | putative transcriptional regulator | Transcriptional regulators | Cytoplasmic [Class 3] |
| PA14_47240 | cadR | putative transcriptional regulator CadR | Transcriptional regulators | Cytoplasmic [Class 3] |
| PA14_47270 | merR | putative transcriptional regulator MerR | Transcriptional regulators | Cytoplasmic [Class 3] |
| PA14_47520 | nfxB | putative transcriptional regulator NfxB | Transcriptional regulators | Cytoplasmic [Class 3] |
| PA14_47820 | gnyR | Regulatory gene of gnyRDBHAL cluster, GnyR | Transcriptional regulators | Cytoplasmic [Class 3] |
| PA14_48190 | PA14_01840 | RNA polymerase ECF-subfamily sigma-70 factor | Transcriptional regulators | Cytoplasmic [Class 3] |
| PA14_48770 | PA14_21550 | RNA polymerase ECF-subfamily sigma-70 factor | Transcriptional regulators | Cytoplasmic [Class 3] |
| PA14_49150 | PA14_46660 | RNA polymerase ECF-subfamily sigma-70 factor | Transcriptional regulators | Cytoplasmic [Class 3] |
| PA14_49700 | PA14_46810 | RNA polymerase ECF-subfamily sigma-70 factor | Transcriptional regulators | Cytoplasmic [Class 3] |
| PA14_53720 | PA14_26600 | RNA polymerase sigma factor | Transcriptional regulators | Cytoplasmic Membrane [Class 3] |
| PA14_55250 | PA14_37990 | RNA polymerase sigma factor | Transcriptional regulators | Cytoplasmic [Class 3] |
| PA14_55540 | PA14_64700 | RNA polymerase sigma factor | Transcriptional regulators | Cytoplasmic [Class 3] |
| PA14_58510 | rpoS | RNA polymerase sigma factor RpoS | Transcriptional regulators | Cytoplasmic [Class 3] |
| PA14_61620 | PA14_35210 | TetR family transcriptional regulator | Transcriptional regulators | Cytoplasmic [Class 3] |
| PA14_63880 | PA14_21970 | transcriptional regulator | Transcriptional regulators | Cytoplasmic [Class 3] |
| PA14_64700 | PA14_32970 | transcriptional regulator | Transcriptional regulators | Cytoplasmic [Class 3] |
| PA14_67550 | PA14_49150 | transcriptional regulator | Transcriptional regulators | Cytoplasmic [Class 3] |
| PA14_68550 | PA14_49700 | transcriptional regulator | Transcriptional regulators | Cytoplasmic [Class 3] |
| PA14_71330 | PA14_53720 | transcriptional regulator | Transcriptional regulators | Cytoplasmic [Class 3] |
| PA14_42490 | PA14_55250 | transcriptional regulator | Transcriptional regulators | Cytoplasmic [Class 3] |
| PA14_47310 | ampR | transcriptional regulator AmpR | Transcriptional regulators | Cytoplasmic [Class 3] |
| PA14_03070 | catR | transcriptional regulator CatR | Transcriptional regulators | Cytoplasmic [Class 3] |
| PA14_07950 | cysB | transcriptional regulator CysB | Transcriptional regulators | Cytoplasmic [Class 3] |
| PA14_07960 | merD | transcriptional regulator MerD | Transcriptional regulators | Unknown [Class 3] |
| PA14_05270 | pcrR | transcriptional regulator protein PcrR | Transcriptional regulators | Unknown [Class 3] |
| PA14_17480 | prtN | transcriptional regulator PrtN | Transcriptional regulators | Unknown [Class 3] |
| PA14_52570 | prtR | transcriptional regulator PrtR | Transcriptional regulators | Cytoplasmic [Class 3] |
| PA14_34150 | PA14_55540 | transmembrane sensor | Transcriptional regulators | Cytoplasmic Membrane [Class 3] |
| PA14_64820 | PA14_34730 | XRE family transcriptional regulator | Transcriptional regulators | Cytoplasmic [Class 3] |
| PA14_66750 | rpsJ | 30S ribosomal protein S10 | Translation, post-translational modification, degradation | Cytoplasmic [Class 3] |
| PA14_41360 | rpsK | 30S ribosomal protein S11 | Translation, post-translational modification, degradation | Cytoplasmic [Class 3] |
| PA14_00200 | rpsN | 30S ribosomal protein S14 | Translation, post-translational modification, degradation | Cytoplasmic [Class 3] |
| PA14_07800 | rpsO | 30S ribosomal protein S15 | Translation, post-translational modification, degradation | Cytoplasmic [Class 3] |
| PA14_67100 | rpsQ | 30S ribosomal protein S17 | Translation, post-translational modification, degradation | Cytoplasmic [Class 3] |
| PA14_00190 | rpsF | 30S ribosomal protein S6 | Translation, post-translational modification, degradation | Cytoplasmic [Class 3] |
| PA14_17100 | rpsG | 30S ribosomal protein S7 | Translation, post-translational modification, degradation | Cytoplasmic [Class 3] |
| PA14_00090 | rpsH | 30S ribosomal protein S8 | Translation, post-translational modification, degradation | Cytoplasmic [Class 3] |
| PA14_14890 | rplA | 50S ribosomal protein L1 | Translation, post-translational modification, degradation | Cytoplasmic [Class 3] |
| PA14_30240 | rplK | 50S ribosomal protein L11 | Translation, post-translational modification, degradation | Cytoplasmic [Class 3] |
| PA14_16530 | rplQ | 50S ribosomal protein L17 | Translation, post-translational modification, degradation | Cytoplasmic [Class 3] |
| PA14_28590 | rplB | 50S ribosomal protein L2 | Translation, post-translational modification, degradation | Cytoplasmic [Class 3] |
| PA14_19050 | rplT | 50S ribosomal protein L20 | Translation, post-translational modification, degradation | Cytoplasmic [Class 3] |
| PA14_65320 | rplU | 50S ribosomal protein L21 | Translation, post-translational modification, degradation | Cytoplasmic [Class 3] |
| PA14_39710 | rplV | 50S ribosomal protein L22 | Translation, post-translational modification, degradation | Cytoplasmic [Class 3] |
| PA14_00240 | rpmC | 50S ribosomal protein L29 | Translation, post-translational modification, degradation | Cytoplasmic [Class 3] |
| PA14_12840 | rplC | 50S ribosomal protein L3 | Translation, post-translational modification, degradation | Cytoplasmic [Class 3] |
| PA14_12850 | rpmE | 50S ribosomal protein L31 | Translation, post-translational modification, degradation | Cytoplasmic [Class 3] |
| PA14_21820 | rpmE2 | 50S ribosomal protein L31 type B | Translation, post-translational modification, degradation | Unknown [Class 3] |
| PA14_34850 | rpmF | 50S ribosomal protein L32 | Translation, post-translational modification, degradation | Cytoplasmic [Class 3] |
| PA14_42790 | rplE | 50S ribosomal protein L5 | Translation, post-translational modification, degradation | Cytoplasmic [Class 3] |
| PA14_44200 | PA14_12840 | acetyltransferase | Translation, post-translational modification, degradation | Cytoplasmic [Class 3] |
| PA14_44270 | PA14_12850 | acetyltransferase | Translation, post-translational modification, degradation | Unknown [Class 3] |
| PA14_49870 | argS | arginyl-tRNA synthetase | Translation, post-translational modification, degradation | Cytoplasmic [Class 3] |
| PA14_60350 | cysS | cysteinyl-tRNA synthetase | Translation, post-translational modification, degradation | Cytoplasmic [Class 3] |
| PA14_70480 | dtD | D-tyrosyl-tRNA(Tyr) deacylase | Translation, post-translational modification, degradation | Unknown [Class 3] |
| PA14_49340 | tufA | elongation factor Tu | Translation, post-translational modification, degradation | Cytoplasmic [Class 3] |
| PA14_04650 | glyS | glycyl-tRNA synthetase subunit beta | Translation, post-translational modification, degradation | Cytoplasmic [Class 3] |
| PA14_41390 | hisS | histidyl-tRNA synthetase | Translation, post-translational modification, degradation | Cytoplasmic [Class 3] |
| PA14_72200 | djlA | hypothetical protein | Translation, post-translational modification, degradation | Cytoplasmic Membrane [Class 3] |
| PA14_61790 | PA14_00240 | hypothetical protein | Translation, post-translational modification, degradation | Cytoplasmic [Class 3] |
| PA14_08730 | PA14_44200 | hypothetical protein | Translation, post-translational modification, degradation | Cytoplasmic [Class 3] |
| PA14_08880 | PA14_70480 | hypothetical protein | Translation, post-translational modification, degradation | Cytoplasmic [Class 3] |
| PA14_08850 | rsmC | hypothetical protein | Translation, post-translational modification, degradation | Cytoplasmic [Class 3] |
| PA14_08970 | yeaZ | hypothetical protein | Translation, post-translational modification, degradation | Cytoplasmic Membrane [Class 3] |
| PA14_08720 | yhbY | hypothetical protein | Translation, post-translational modification, degradation | Cytoplasmic [Class 3] |
| PA14_09130 | lysS | lysyl-tRNA synthetase | Translation, post-translational modification, degradation | Cytoplasmic [Class 3] |
| PA14_28680 | fmt | methionyl-tRNA formyltransferase | Translation, post-translational modification, degradation | Periplasmic [Class 3] |
| PA14_60460 | metG | methionyl-tRNA synthetase | Translation, post-translational modification, degradation | Cytoplasmic [Class 3] |
| PA14_08900 | PA14_42790 | N5-glutamine S-adenosyl-L-methionine-dependent methyltransferase | Translation, post-translational modification, degradation | Cytoplasmic [Class 3] |
| PA14_08930 | pcpS | PcpS | Translation, post-translational modification, degradation | Cytoplasmic [Class 3] |
| PA14_66710 | prfH | peptide chain release factor-like protein | Translation, post-translational modification, degradation | Cytoplasmic [Class 3] |
| PA14_17700 | def | peptide deformylase | Translation, post-translational modification, degradation | Cytoplasmic [Class 3] |
| PA14_25630 | PA14_49870 | peptide deformylase | Translation, post-translational modification, degradation | Cytoplasmic [Class 3] |
| PA14_65180 | ppiB | peptidyl-prolyl cis-trans isomerase B | Translation, post-translational modification, degradation | Cytoplasmic [Class 3] |
| PA14_08810 | PA14_60350 | peptidyl-prolyl cis-trans isomerase, FkbP-type | Translation, post-translational modification, degradation | Cytoplasmic [Class 3] |
| PA14_08990 | pth | peptidyl-tRNA hydrolase | Translation, post-translational modification, degradation | Cytoplasmic [Class 3] |
| PA14_08840 | pfpI | protease PfpI | Translation, post-translational modification, degradation | Cytoplasmic [Class 3] |
| PA14_09090 | mapB | putative methionine aminopeptidase | Translation, post-translational modification, degradation | Cytoplasmic [Class 3] |
| PA14_08980 | PA14_21820 | putative peptidyl-prolyl cis-trans isomerase, FkbP-type | Translation, post-translational modification, degradation | Cytoplasmic [Class 3] |
| PA14_62720 | nrdG | putative radical-activating enzyme | Translation, post-translational modification, degradation | Cytoplasmic [Class 3] |
| PA14_08940 | PA14_34850 | putative tRNA synthase | Translation, post-translational modification, degradation | Cytoplasmic [Class 3] |
| PA14_61220 | frr | ribosome recycling factor | Translation, post-translational modification, degradation | Cytoplasmic [Class 3] |
| PA14_30330 | serS | seryl-tRNA synthetase | Translation, post-translational modification, degradation | Cytoplasmic [Class 3] |
| PA14_23840 | PA14_44270 | sulfur transfer protein SirA | Translation, post-translational modification, degradation | Cytoplasmic [Class 3] |
| PA14_08830 | infA | translation initiation factor IF-1 | Translation, post-translational modification, degradation | Cytoplasmic [Class 3] |
| PA14_16710 | miaA | tRNA delta(2)-isopentenylpyrophosphate transferase | Translation, post-translational modification, degradation | Cytoplasmic [Class 3] |
| PA14_62880 | truA | tRNA pseudouridine synthase A | Translation, post-translational modification, degradation | Cytoplasmic [Class 3] |
| PA14_57100 | PA14_11310 | ABC transporter ATP-binding protein | Transport of small molecules | Cytoplasmic Membrane [Class 3] |
| PA14_52780 | ssuB | ABC transporter ATP-binding protein | Transport of small molecules | Cytoplasmic Membrane [Class 3] |
| PA14_18680 | PA14_13590 | ABC transporter permease | Transport of small molecules | Cytoplasmic Membrane [Class 3] |
| PA14_45380 | PA14_21150 | ABC transporter permease | Transport of small molecules | Cytoplasmic Membrane [Class 3] |
| PA14_45370 | PA14_67040 | ABC transporter permease | Transport of small molecules | Cytoplasmic Membrane [Class 3] |
| PA14_45350 | PA14_68080 | ABC transporter permease | Transport of small molecules | Cytoplasmic Membrane [Class 3] |
| PA14_03650 | ssuC | ABC transporter permease | Transport of small molecules | Cytoplasmic Membrane [Class 3] |
| PA14_31990 | ssuC | ABC transporter permease | Transport of small molecules | Cytoplasmic Membrane [Class 3] |
| PA14_47430 | PA14_46930 | amino acid ABC transporter membrane protein | Transport of small molecules | Cytoplasmic Membrane [Class 3] |
| PA14_25500 | PA14_47950 | amino acid ABC transporter membrane protein | Transport of small molecules | Cytoplasmic Membrane [Class 3] |
| PA14_02510 | PA14_34800 | amino acid transporter LysE | Transport of small molecules | Cytoplasmic Membrane [Class 3] |
| PA14_55340 | aotQ | arginine/ornithine transport protein AotQ | Transport of small molecules | Cytoplasmic Membrane [Class 3] |
| PA14_10170 | hisZ | ATP phosphoribosyltransferase regulatory subunit | Transport of small molecules | Cytoplasmic [Class 3] |
| PA14_10160 | phnN | ATP-binding component of phosphonate transport | Transport of small molecules | Cytoplasmic [Class 3] |
| PA14_63710 | rbsB | binding protein component precursor of ABC ribose transporter | Transport of small molecules | Periplasmic [Class 3] |
| PA14_26240 | czcB | cobalt/zinc/cadmium efflux RND transporter, membrane fusion protein, CzcB famil | Transport of small molecules | Cytoplasmic Membrane [Class 3] |
| PA14_26230 | ccmA | cytochrome c biogenesis protein CcmA | Transport of small molecules | Cytoplasmic Membrane [Class 3] |
| PA14_65250 | PA14_00070 | D,D-heptose 1,7-bisphosphate phosphatase | Transport of small molecules | Cytoplasmic [Class 3] |
| PA14_43405 | fepD | ferric enterobactin transport protein FepD | Transport of small molecules | Cytoplasmic Membrane [Class 3] |
| PA14_43380 | oprB | glucose/carbohydrate outer membrane porin OprB precursor | Transport of small molecules | Outer Membrane [Class 3] |
| PA14_43370 | ccmB | heme exporter protein CcmB | Transport of small molecules | Cytoplasmic Membrane [Class 3] |
| PA14_25430 | ccmC | heme exporter protein CcmC | Transport of small molecules | Cytoplasmic Membrane [Class 3] |
| PA14_34270 | hisQ | histidine transport system permease protein | Transport of small molecules | Cytoplasmic Membrane [Class 3] |
| PA14_34280 | bfd | hypothetical protein | Transport of small molecules | Unknown [Class 3] |
| PA14_09530 | exbD | hypothetical protein | Transport of small molecules | Unknown [Class 3] |
| PA14_40420 | lolC | hypothetical protein | Transport of small molecules | Cytoplasmic Membrane [Class 3] |
| PA14_37260 | metQ-l | hypothetical protein | Transport of small molecules | Cytoplasmic Membrane [Class 3] |
| PA14_23030 | PA14_00850 | hypothetical protein | Transport of small molecules | Cytoplasmic Membrane [Class 3] |
| PA14_51710 | PA14_11530 | hypothetical protein | Transport of small molecules | Periplasmic [Class 3] |
| PA14_00070 | PA14_18800 | hypothetical protein | Transport of small molecules | Unknown [Class 3] |
| PA14_00850 | PA14_26160 | hypothetical protein | Transport of small molecules | Cytoplasmic Membrane [Class 3] |
| PA14_00860 | PA14_29820 | hypothetical protein | Transport of small molecules | Cytoplasmic Membrane [Class 3] |
| PA14_01690 | PA14_30370 | hypothetical protein | Transport of small molecules | Cytoplasmic [Class 3] |
| PA14_11310 | PA14_36780 | hypothetical protein | Transport of small molecules | Cytoplasmic Membrane [Class 3] |
| PA14_11530 | PA14_37290 | hypothetical protein | Transport of small molecules | Cytoplasmic [Class 3] |
| PA14_12940 | PA14_37310 | hypothetical protein | Transport of small molecules | Cytoplasmic [Class 3] |
| PA14_13590 | PA14_40130 | hypothetical protein | Transport of small molecules | Unknown [Class 3] |
| PA14_14370 | PA14_43570 | hypothetical protein | Transport of small molecules | Cytoplasmic Membrane [Class 3] |
| PA14_16310 | PA14_57990 | hypothetical protein | Transport of small molecules | Cytoplasmic Membrane [Class 3] |
| PA14_16870 | fepB | iron-enterobactin transporter periplasmic binding protein | Transport of small molecules | Periplasmic [Class 3] |
| PA14_16880 | PA14_69310 | LysE family efflux protein | Transport of small molecules | Cytoplasmic Membrane [Class 3] |
| PA14_18800 | PA14_34710 | major facilitator transporter | Transport of small molecules | Cytoplasmic Membrane [Class 3] |
| PA14_21150 | modC | molybdenum transport protein ModC | Transport of small molecules | Cytoplasmic [Class 3] |
| PA14_21780 | oprL | Peptidoglycan associated lipoprotein OprL precursor | Transport of small molecules | Outer Membrane [Class 3] |
| PA14_25490 | hisJ | periplasmic histidine-binding protein HisJ | Transport of small molecules | Periplasmic [Class 3] |
| PA14_26160 | phnL | phosphonate ABC transporter, ATP-binding protein | Transport of small molecules | Cytoplasmic Membrane [Class 3] |
| PA14_29820 | potC | polyamine transport protein PotC | Transport of small molecules | Cytoplasmic Membrane [Class 3] |
| PA14_30370 | trkA | potassium transporter peripheral membrane component | Transport of small molecules | Cytoplasmic Membrane [Class 3] |
| PA14_31800 | kdpB | potassium-transporting ATPase subunit B | Transport of small molecules | Cytoplasmic Membrane [Class 3] |
| PA14_33540 | kdpC | potassium-transporting ATPase subunit C | Transport of small molecules | Cytoplasmic Membrane [Class 3] |
| PA14_34710 | kdbF | potassium-transporting ATPase subunit F | Transport of small molecules | Cytoplasmic Membrane [Class 3] |
| PA14_34780 | PA14_64310 | putative ABC transport ATP-binding subunit | Transport of small molecules | Cytoplasmic Membrane [Class 3] |
| PA14_34800 | PA14_34780 | putative ABC transporter ATP-binding component | Transport of small molecules | Cytoplasmic Membrane [Class 3] |
| PA14_36780 | PA14_16870 | putative ABC transporter ATP-binding protein | Transport of small molecules | Cytoplasmic [Class 3] |
| PA14_37290 | PA14_14370 | putative ABC-transporter ATP-binding component | Transport of small molecules | Cytoplasmic Membrane [Class 3] |
| PA14_37310 | PA14_46440 | putative acetyltransferase | Transport of small molecules | Unknown [Class 3] |
| PA14_37420 | yhdW | putative amino acid-binding protein | Transport of small molecules | Periplasmic [Class 3] |
| PA14_37440 | PA14_43130 | putative aminotransferase | Transport of small molecules | Cytoplasmic [Class 3] |
| PA14_37840 | metN-1 | putative ATP-binding component of ABC transporter | Transport of small molecules | Cytoplasmic Membrane [Class 3] |
| PA14_40130 | PA14_00860 | putative ATP-binding component of ABC transporter | Transport of small molecules | Cytoplasmic Membrane [Class 3] |
| PA14_43130 | PA14_37840 | putative ATP-binding component of ABC transporter | Transport of small molecules | Cytoplasmic Membrane [Class 3] |
| PA14_43570 | PA14_64300 | putative ATP-binding component of ABC transporter | Transport of small molecules | Cytoplasmic Membrane [Class 3] |
| PA14_45060 | pchH | putative ATP-binding component of ABC transporter | Transport of small molecules | Cytoplasmic Membrane [Class 3] |
| PA14_46440 | yrbF | putative ATP-binding component of ABC transporter | Transport of small molecules | Cytoplasmic Membrane [Class 3] |
| PA14_46930 | PA14_71020 | putative BC-type proline/glycine betaine transport system, permease component | Transport of small molecules | Cytoplasmic Membrane [Class 3] |
| PA14_47900 | gtrB | putative glycosyl transferase | Transport of small molecules | Cytoplasmic Membrane [Class 3] |
| PA14_47950 | PA14_66110 | putative glycosyl transferase | Transport of small molecules | Cytoplasmic Membrane [Class 3] |
| PA14_48300 | czcD | putative metal transporter | Transport of small molecules | Cytoplasmic Membrane [Class 3] |
| PA14_54110 | PA14_16310 | putative MFS permease | Transport of small molecules | Cytoplasmic Membrane [Class 3] |
| PA14_57990 | PA14_37440 | putative MFS transporter | Transport of small molecules | Cytoplasmic Membrane [Class 3] |
| PA14_64290 | PA14_47900 | putative MFS transporter | Transport of small molecules | Cytoplasmic Membrane [Class 3] |
| PA14_64300 | PA14_48300 | putative MFS transporter | Transport of small molecules | Cytoplasmic Membrane [Class 3] |
| PA14_64310 | yaaU | putative MFS transporter | Transport of small molecules | Cytoplasmic Membrane [Class 3] |
| PA14_66110 | ampG | putative permease | Transport of small molecules | Cytoplasmic Membrane [Class 3] |
| PA14_67040 | PA14_01690 | putative permease of ABC transporter | Transport of small molecules | Cytoplasmic Membrane [Class 3] |
| PA14_68080 | PA14_16880 | putative permease of ABC transporter | Transport of small molecules | Cytoplasmic Membrane [Class 3] |
| PA14_69310 | PA14_33540 | putative permease of ABC transporter | Transport of small molecules | Cytoplasmic Membrane [Class 3] |
| PA14_71020 | PA14_64290 | putative permease of ABC transporter | Transport of small molecules | Cytoplasmic Membrane [Class 3] |
| PA14_09300 | phnG | putative phosphonate metabolism protein PhnG | Transport of small molecules | Unknown [Class 3] |
| PA14_20360 | opdO | putative porin | Transport of small molecules | Outer Membrane [Class 3] |
| PA14_20420 | trpB | putative secretion protein | Transport of small molecules | Cytoplasmic Membrane [Class 3] |
| PA14_20440 | PA14_31800 | putative sodium:alanine symporter | Transport of small molecules | Cytoplasmic Membrane [Class 3] |
| PA14_17620 | PA14_25490 | putative tolQ-type transport protein | Transport of small molecules | Cytoplasmic Membrane [Class 3] |
| PA14_39350 | PA14_37420 | putative transmembrane sensor protein | Transport of small molecules | Periplasmic [Class 3] |
| PA14_19580 | PA14_21780 | putative transporter | Transport of small molecules | Cytoplasmic Membrane [Class 3] |
| PA14_19510 | PA14_45060 | putative transporter | Transport of small molecules | Cytoplasmic Membrane [Class 3] |
| PA14_19570 | yigN | rhodanese-like domain-containing protein | Transport of small molecules | Unknown [Class 3] |
| PA14_51750 | mexH | RND efflux membrane fusion protein precursor | Transport of small molecules | Cytoplasmic Membrane [Class 3] |
| PA14_51740 | cysA | sulfate transport protein CysA | Transport of small molecules | Cytoplasmic Membrane [Class 3] |
| PA14_72970 | PA14_12940 | taurine ABC transporter ATP-binding protein | Transport of small molecules | Cytoplasmic Membrane [Class 3] |
| PA14_00170 | tolQ | TolQ protein | Transport of small molecules | Cytoplasmic Membrane [Class 3] |
| PA14_27420 | tolR | TolR protein | Transport of small molecules | Cytoplasmic Membrane [Class 3] |
| PA14_09740 | tonB | TonB protein | Transport of small molecules | Periplasmic [Class 3] |
| PA14_14100 | exbD1 | transport protein ExbD | Transport of small molecules | Cytoplasmic Membrane [Class 3] |
| PA14_67750 | exbD2 | transport protein ExbD | Transport of small molecules | Cytoplasmic Membrane [Class 3] |
| PA14_22670 | PA14_54110 | transporter | Transport of small molecules | Cytoplasmic Membrane [Class 3] |
| PA14_72580 | znuC | zinc transporter | Transport of small molecules | Cytoplasmic Membrane [Class 3] |
| PA14_02260 | PA14_06310 | ACT domain-containing protein | Two-component regulatory systems | Cytoplasmic [Class 3] |
| PA14_45620 | PA14_73020 | DksA/TraR family C4-type zinc finger protein | Two-component regulatory systems | Cytoplasmic [Class 3] |
| PA14_22760 | PA14_36990 | EAL domain-containing protein | Two-component regulatory systems | Cytoplasmic Membrane [Class 3] |
| PA14_31960 | PA14_02110 | GGDEF domain-containing protein | Two-component regulatory systems | Cytoplasmic [Class 3] |
| PA14_72720 | PA14_20800 | histidine phosphotransfer domain-containing protein | Two-component regulatory systems | Cytoplasmic [Class 3] |
| PA14_68680 | nasT | hypothetical protein | Two-component regulatory systems | Cytoplasmic [Class 3] |
| PA14_38910 | PA14_03470 | hypothetical protein | Two-component regulatory systems | Cytoplasmic [Class 3] |
| PA14_38900 | PA14_22370 | hypothetical protein | Two-component regulatory systems | Cytoplasmic [Class 3] |
| PA14_22940 | PA14_36920 | hypothetical protein | Two-component regulatory systems | Cytoplasmic [Class 3] |
| PA14_41490 | ybiI | hypothetical protein | Two-component regulatory systems | Unknown [Class 3] |
| PA14_02110 | exaD | putative sensor kinase | Two-component regulatory systems | Cytoplasmic Membrane [Class 3] |
| PA14_03470 | PA14_47390 | putative transmembrane sensor | Two-component regulatory systems | Cytoplasmic Membrane [Class 3] |
| PA14_06310 | cheY | putative two-component response regulator | Two-component regulatory systems | Cytoplasmic [Class 3] |
| PA14_09690 | cheY | putative two-component response regulator | Two-component regulatory systems | Cytoplasmic [Class 3] |
| PA14_20800 | cpxR | putative two-component response regulator | Two-component regulatory systems | Cytoplasmic [Class 3] |
| PA14_22370 | czrR | putative two-component response regulator | Two-component regulatory systems | Cytoplasmic [Class 3] |
| PA14_24350 | dctD | putative two-component response regulator | Two-component regulatory systems | Cytoplasmic [Class 3] |
| PA14_27940 | exaE | putative two-component response regulator | Two-component regulatory systems | Cytoplasmic [Class 3] |
| PA14_29730 | PA14_09690 | putative two-component response regulator | Two-component regulatory systems | Cytoplasmic [Class 3] |
| PA14_32570 | PA14_24350 | putative two-component response regulator | Two-component regulatory systems | Cytoplasmic [Class 3] |
| PA14_36920 | PA14_27940 | putative two-component response regulator | Two-component regulatory systems | Cytoplasmic [Class 3] |
| PA14_36990 | PA14_29730 | putative two-component response regulator | Two-component regulatory systems | Cytoplasmic [Class 3] |
| PA14_46360 | PA14_46360 | putative two-component response regulator | Two-component regulatory systems | Cytoplasmic [Class 3] |
| PA14_47390 | PA14_58300 | putative two-component response regulator | Two-component regulatory systems | Cytoplasmic [Class 3] |
| PA14_57170 | PA14_63210 | putative two-component response regulator | Two-component regulatory systems | Cytoplasmic [Class 3] |
| PA14_58300 | uhpA | putative two-component response regulator | Two-component regulatory systems | Cytoplasmic [Class 3] |
| PA14_63210 | PA14_57170 | putative two-component sensor | Two-component regulatory systems | Cytoplasmic Membrane [Class 3] |
| PA14_64580 | PA14_64580 | putative two-component sensor | Two-component regulatory systems | Cytoplasmic Membrane [Class 3] |
| PA14_73020 | gltR | two-component response regulator GltR | Two-component regulatory systems | Cytoplasmic [Class 3] |
| PA14_07840 | PA14_32570 | two-component sensor | Two-component regulatory systems | Cytoplasmic Membrane [Class 3] |
| PA14_64410 | envZ | two-component sensor EnvZ | Two-component regulatory systems | Cytoplasmic Membrane [Class 3] |
