# Supplementary material for: A Comprehensive Analysis of In Vitro and In Vivo Genetic Fitness of Pseudomonas aeruginosa Using High-Throughput Sequencing of Transposon Libraries
Source: PLoS Pathog. 2013 Sep 5;9(9):e1003582. doi: 10.1371/journal.ppat.1003582 (PMC3764216; doi:10.1371/journal.ppat.1003582)
Supplement: Table S6 — Nine operons with unannotated genes found in strain PA14 and no other sequenced P. aeruginosa strains that all have a have a reduce fitness for colonization. (DOC) [file ppat.1003582.s017.doc]

| Table S6: Nine operons with unannotated genes found in strain PA14 and no other sequenced *P. aeruginosa* strains that all have a have a reduce fitness for colonization | | | | |
| --- | --- | --- | --- | --- |
| ID | Results from BLAST alignment | Product Name | Function Class | Subcellular Localization |
| PA14_03330 | Acidithiobacillus ferrivorans SS3:histidinol phosphate phosphatase HisJ family, Identities = 779/867 (90%), Gaps = 0/867 (0%), , | hypothetical protein | Hypothetical, unclassified, unknown | Cytoplasmic [Class 3] |
| PA14_03340 | Identities = 817/927 (88%), Gaps = 0/927 (0%) | hypothetical protein | Hypothetical, unclassified, unknown | Cytoplasmic [Class 3] |
|  |  |  |  |  |
| PA14_13960 | Blastp: 100% multidrug efflux: GENE ID: 4380303 amrB | multidrug efflux protein | hypothetical protein | Hypothetical, unclassified, unknown | Unknown [Class 3] |
| PA14_13970 | 260/261 identities | hypothetical protein | Hypothetical, unclassified, unknown | Unknown [Class 3] |
|  |  |  |  |  |
| PA14_15570 | None |  |  |  |
| PA14_15580 |  | Type II restriction enzyme, methylase subunit | Putative enzymes | Cytoplasmic [Class 3] |
| PA14_15590 |  | hypothetical protein | Hypothetical, unclassified, unknown | Unknown [Class 3] |
| PA14_15600 |  | hypothetical protein | Hypothetical, unclassified, unknown | Cytoplasmic [Class 3] |
| PA14_15610 |  | hypothetical protein | Hypothetical, unclassified, unknown | Cytoplasmic [Class 3] |
|  |  |  |  |  |
| PA14_22180 |  | hypothetical protein | Hypothetical, unclassified, unknown | Unknown [Class 3] |
| PA14_22190 |  | hypothetical protein | Hypothetical, unclassified, unknown | Unknown [Class 3] |
|  |  |  |  |  |
| PA14_28850 | IS222 | hypothetical protein | Hypothetical, unclassified, unknown | Unknown [Class 3] |
| PA14_28870 | IS222 | hypothetical protein | Hypothetical, unclassified, unknown | Unknown [Class 3] |
|  |  |  |  |  |
| PA14_48230 | None | hypothetical protein | Hypothetical, unclassified, unknown | Unknown [Class 3] |
| PA14_48240 |  | putative outer membrane component of multidrug efflux pump | Antibiotic resistance and susceptibility | Outer Membrane [Class 3] |
| PA14_48280 |  | putative multidrug resistance efflux pump | Antibiotic resistance and susceptibility | Cytoplasmic Membrane [Class 3] |
| PA14_48300 |  | putative MFS transporter | Transport of small molecules | Cytoplasmic Membrane [Class 3] |
|  |  |  |  |  |
| PA14_55000 | None | ABC transporter periplasmic protein | Transport of small molecules | Unknown [Class 3] |
| PA14_55020 |  | ABC transporter permease/ABC-type Fe3+-siderophore transporter permease | Transport of small molecules | Cytoplasmic Membrane [Class 3] |
| PA14_55030 |  | ABC transporter permease/ABC-type Fe3+-siderophore transport system, permease component | Transport of small molecules | Cytoplasmic Membrane [Class 3] |
| PA14_55040 |  | putative ATP-binding component of ferric enterobactin transport/ABC-type cobalamin/Fe3+-siderophores transport systems, ATPase components | Transport of small molecules | Cytoplasmic Membrane [Class 3] |
|  |  |  |  |  |
| PA14_67180 | None | hypothetical protein | Hypothetical, unclassified, unknown | Unknown [Class 3] |
| PA14_67190 |  | hypothetical protein | Hypothetical, unclassified, unknown | Unknown [Class 3] |
| PA14_67200 |  | hypothetical protein | Hypothetical, unclassified, unknown | Unknown [Class 3] |
| PA14_67210 |  | hypothetical protein | Hypothetical, unclassified, unknown | Unknown [Class 3] |
| PA14_67220 |  | hypothetical protein | Hypothetical, unclassified, unknown | Extracellular [Class 3] |
| PA14_67230 |  | hypothetical protein | Hypothetical, unclassified, unknown | Cytoplasmic [Class 3] |
|  |  |  |  |  |
| PA14_72820 | None | hypothetical protein | Hypothetical, unclassified, unknown | Unknown [Class 3] |
| PA14_72830 |  | hypothetical protein | Hypothetical, unclassified, unknown | Unknown [Class 3] |
