# Supplementary material for: A Comprehensive Analysis of In Vitro and In Vivo Genetic Fitness of Pseudomonas aeruginosa Using High-Throughput Sequencing of Transposon Libraries
Source: PLoS Pathog. 2013 Sep 5;9(9):e1003582. doi: 10.1371/journal.ppat.1003582 (PMC3764216; doi:10.1371/journal.ppat.1003582)
Supplement: Table S7 — Tn-insertions in genes within operons of unannotated genes found in strain PA14 and other sequenced P. aeruginosa strains that have a reduce fitness for colonization. (DOC) [file ppat.1003582.s018.doc]

| Table S7: Tn-insertions in genes within operons of unannotated genes found in strain PA14 and other sequenced P. aeruginosa strains that have a reduce fitness for colonization | | | | | |
| --- | --- | --- | --- | --- | --- |
| ID | Operon number | Orthologs in other *P. aeruginosa* strains (www.pseudomonas.com) | Product Name | Functional Class | Subcellular Localization |
| PA14_00660 | 1 | 2192/3719/3907/PA7/PACS2/PA01 | RNA 2'-phosphotransferase-like protein | Putative enzymes | Unknown [Class 3] |
| PA14_00670 | 1 |  | hypothetical protein | Hypothetical, unclassified, unknown | Cytoplasmic [Class 3] |
| PA14_03190 | 2 | C3719/LESB58/PA01/PA7/2192 | hypothetical protein | Hypothetical, unclassified, unknown | Unknown [Class 3] |
| PA14_03200 | 2 |  | Hypothetical protein | Hypothetical, unclassified, unknown | Unknown [Class 3] |
| PA14_03210 | 2 |  | hypothetical protein | Hypothetical, unclassified, unknown | Unknown [Class 3] |
| PA14_03220 | 2 |  | Hypothetical protein | Hypothetical, unclassified, unknown | Cytoplasmic [Class 3] |
| PA14_03490 | 3 | C3719/LESB58/PACS2/PA01/PA7/2192/39016 | hypothetical protein | Carbon compound catabolism | Unknown [Class 3] |
| PA14_03510 | 3 |  | hypothetical protein | Hypothetical, unclassified, unknown | Unknown [Class 3] |
| PA14_03520 | 3 |  | hypothetical protein | Hypothetical, unclassified, unknown | Unknown [Class 3] |
| PA14_04560 | 4 | C3719/LESB58/PACS2/PA01/PA7/2192 | hypothetical protein | Hypothetical, unclassified, unknown | Unknown [Class 3] |
| PA14_04570 | 4 |  | hypothetical protein | Hypothetical, unclassified, unknown | Unknown [Class 3] |
| PA14_04830 | 5 | PA2192 | putative acetyltransferase | Transport of small molecules | Unknown [Class 3] |
| PA14_04840 | 5 |  | hypothetical protein | Putative enzymes | Unknown [Class 3] |
| PA14_04850 | 5 |  | hypothetical protein | Hypothetical, unclassified, unknown | Unknown [Class 3] |
| PA14_04860 | 5 |  | putative methyltransferase | DNA replication, recombination, modification and repair | Unknown [Class 3] |
| PA14_04870 | 5 |  | hypothetical protein | Protein secretion/export apparatus | Unknown [Class 3] |
| PA14_04890 | 5 |  | putative zinc protease | Putative enzymes | Periplasmic [Class 3] |
| PA14_07380 | 6 | C3719/LESB58/PACS2/PA01/PA7/2192/39016 | hypothetical protein | Hypothetical, unclassified, unknown | Cytoplasmic [Class 3] |
| PA14_07400 | 6 |  | hypothetical protein | Hypothetical, unclassified, unknown | Cytoplasmic [Class 3] |
| PA14_07410 | 6 |  | hypothetical protein | Hypothetical, unclassified, unknown | Unknown [Class 3] |
| PA14_07420 | 6 |  | hypothetical protein | Hypothetical, unclassified, unknown | Unknown [Class 3] |
| PA14_10050 | 7 | PA7 | hypothetical protein | Hypothetical, unclassified, unknown | Unknown [Class 3] |
| PA14_10070 | 7 |  | putative zinc-dependent oxidoreductase | Putative enzymes | Cytoplasmic [Class 3] |
| PA14_10080 | 7 |  | hypothetical protein | Hypothetical, unclassified, unknown | Unknown [Class 3] |
| PA14_12640 | 8 | C3719/LESB58/PACS2/PA01/PA7/2192/39016 | hypothetical protein | Hypothetical, unclassified, unknown | Unknown [Class 3] |
| PA14_12650 | 8 |  | hypothetical protein | Hypothetical, unclassified, unknown | Unknown [Class 3] |
| PA14_13290 | 9 | C3719/LESB58/PACS2/PA01/PA7/2192/39016 | putative protease | Translation, post-translational modification, degradation | Cytoplasmic [Class 3] |
| PA14_13300 | 9 |  | hypothetical protein | Translation, post-translational modification, degradation | Cytoplasmic [Class 3] |
| PA14_13320 | 9 |  | hypothetical protein | Fatty acid and phospholipid metabolism | Unknown [Class 3] |
| PA14_13330 | 9 |  | hypothetical protein | Putative enzymes | Extracellular [Class 3] |
| PA14_13340 | 9 |  | extracellular nuclease | Transcription, RNA processing and degradation | Unknown [Class 3] |
| PA14_13350 | 10 | C3719/LESB58/PACS2/PA01/2192/39016 | hypothetical protein | Hypothetical, unclassified, unknown | Cytoplasmic [Class 3] |
| PA14_13360 | 10 |  | hypothetical protein | Hypothetical, unclassified, unknown | Cytoplasmic [Class 3] |
| PA14_13370 | 10 |  | hypothetical protein | Hypothetical, unclassified, unknown | Cytoplasmic [Class 3] |
| PA14_13380 | 10 |  | hypothetical protein | Hypothetical, unclassified, unknown | Unknown [Class 3] |
| PA14_13390 | 10 |  | hypothetical protein | Hypothetical, unclassified, unknown | Unknown [Class 3] |
| PA14_13520 | 11 |  | putative outer membrane protein | Protein secretion/export apparatus | Outer Membrane [Class 3] |
| PA14_13530 | 11 | LESB58/PA01 | putative outer membrane protein | Protein secretion/export apparatus | Cytoplasmic Membrane [Class 3] |
| PA14_13560 | 11 |  | hypothetical protein | Protein secretion/export apparatus | Cytoplasmic Membrane [Class 3] |
| PA14_14170 | 12 | C3719/LESB58/PACS2/PA01/2192/39016 | hypothetical protein | Translation, post-translational modification, degradation | Periplasmic [Class 3] |
| PA14_14200 | 12 |  | hypothetical protein | Membrane proteins | Cytoplasmic Membrane [Class 3] |
| PA14_16560 | 13 | C3719/LESB58/PACS2/PA01/PA7/2192/39016 | putative lipoprotein | Fatty acid and phospholipid metabolism | Unknown [Class 3] |
| PA14_16580 | 13 |  | hypothetical protein | Hypothetical, unclassified, unknown | Cytoplasmic [Class 3] |
| PA14_16590 | 13 |  | hypothetical protein | Hypothetical, unclassified, unknown | Unknown [Class 3] |
| PA14_16600 | 13 |  | alpha/beta family hydrolase | Fatty acid and phospholipid metabolism | Unknown [Class 3] |
| PA14_16610 | 13 |  | hypothetical protein | Fatty acid and phospholipid metabolism | Unknown [Class 3] |
| PA14_16620 | 13 |  | hypothetical protein | Putative enzymes | Unknown [Class 3] |
| PA14_21120 | 14 | C3719/LESB58/PACS2/PA01/2192 | hypothetical protein | Hypothetical, unclassified, unknown | Unknown [Class 3] |
| PA14_21130 | 14 |  | putative outer membrane lipoprotein | Membrane proteins | Unknown [Class 3] |
| PA14_21140 | 14 |  | ABC transporter permease | Transport of small molecules | Cytoplasmic Membrane [Class 3] |
| PA14_21150 | 14 |  | ABC transporter permease | Transport of small molecules | Cytoplasmic Membrane [Class 3] |
| PA14_21160 | 14 |  | ABC transporter ATP-binding protein | Transport of small molecules | Cytoplasmic Membrane [Class 3] |
| PA14_21175 | 14 |  | hypothetical protein | Transport of small molecules | Periplasmic [Class 3] |
| PA14_21850 | 15 | LESB58/PA01 | putative transcriptional regulator | Transcriptional regulators | Unknown [Class 3] |
| PA14_21860 | 15 |  | hypothetical protein | Hypothetical, unclassified, unknown | Unknown [Class 3] |
| PA14_22500 | 15 |  | protein-disulfide isomerase | Translation, post-translational modification, degradation | Cytoplasmic [Class 3] |
| PA14_22510 | 15 |  | hypothetical protein | Hypothetical, unclassified, unknown | Unknown [Class 3] |
| PA14_22520 | 15 |  | hypothetical protein | Hypothetical, unclassified, unknown | Unknown [Class 3] |
| PA14_22530 | 15 |  | putative glutathione S-transferase | Translation, post-translational modification, degradation | Cytoplasmic [Class 3] |
| PA14_24370 | 16 | C3719/LESB58/PACS2/PA01/PA7/2192 | hypothetical protein | Hypothetical, unclassified, unknown | Unknown [Class 3] |
| PA14_24380 | 16 |  | hypothetical protein | Hypothetical, unclassified, unknown | Unknown [Class 3] |
| PA14_24390 | 16 |  | TPR domain-containing protein | Putative enzymes | Cytoplasmic Membrane [Class 3] |
| PA14_24400 | 16 |  | von Willebrand factor type A domain-containing protein | Protein secretion/export apparatus | Cytoplasmic Membrane [Class 3] |
| PA14_24410 | 16 |  | hypothetical protein | Hypothetical, unclassified, unknown | Unknown [Class 3] |
| PA14_24420 | 16 |  | hypothetical protein | Hypothetical, unclassified, unknown | Cytoplasmic [Class 3] |
| PA14_24430 | 16 |  | hypothetical protein | Putative enzymes | Cytoplasmic [Class 3] |
| PA14_26160 | 17 | 2192/PACS2/PA01/PA7 | hypothetical protein | Transport of small molecules | Cytoplasmic Membrane [Class 3] |
| PA14_26165 | 17 |  | hypothetical protein | Hypothetical, unclassified, unknown | Cytoplasmic Membrane [Class 3] |
| PA14_28490 | 18 | C3719/LESB58/PACS2/PA01/PA7/2192/39016 | hypothetical protein | Membrane proteins | Unknown [Class 3] |
| PA14_28500 | 18 |  | hypothetical protein | Hypothetical, unclassified, unknown | Cytoplasmic [Class 3] |
| PA14_31680 | 19 | C3719/LESB58/PACS2/PA01/PA7/2192/39016 | hypothetical protein | Cell wall / LPS / capsule | Outer Membrane [Class 3] |
| PA14_31690 | 19 |  | hypothetical protein | Hypothetical, unclassified, unknown | Outer Membrane [Class 3] |
| PA14_31700 | 19 |  | putative CDP-alcohol phosphatidyltransferase | Fatty acid and phospholipid metabolism | Cytoplasmic Membrane [Class 3] |
| PA14_31720 | 19 |  | hypothetical protein | Fatty acid and phospholipid metabolism | Cytoplasmic [Class 3] |
| PA14_31730 | 19 |  | hypothetical protein | Putative enzymes | Cytoplasmic Membrane [Class 3] |
| PA14_31740 | 19 |  | hypothetical protein | Hypothetical, unclassified, unknown | Cytoplasmic Membrane [Class 3] |
| PA14_31750 | 19 |  | putative acyltransferase | Fatty acid and phospholipid metabolism | Unknown [Class 3] |
| PA14_31760 | 19 |  | putative phosphatidate cytidylyltransferase | Fatty acid and phospholipid metabolism | Cytoplasmic Membrane [Class 3] |
| PA14_32860 | 20 | C3719/LESB58/PACS2/PA01/PA7/2192/39016 | hypothetical protein | Hypothetical, unclassified, unknown | Cytoplasmic [Class 3] |
| PA14_32880 | 20 |  | hypothetical protein | Hypothetical, unclassified, unknown | Unknown [Class 3] |
| PA14_32890 | 20 |  | hypothetical protein | Hypothetical, unclassified, unknown | Unknown [Class 3] |
| PA14_33190 | 21 | PA7/PACS2 | hypothetical protein | Hypothetical, unclassified, unknown | Cytoplasmic Membrane [Class 3] |
| PA14_33200 | 21 |  | hypothetical protein | Hypothetical, unclassified, unknown | Cytoplasmic Membrane [Class 3] |
| PA14_33220 | 21 |  | hypothetical protein | Hypothetical, unclassified, unknown | Unknown [Class 3] |
| PA14_34730 | 22 | C3719/LESB58/PACS2/PA01/2192/39016 | XRE family transcriptional regulator | Transcriptional regulators | Cytoplasmic [Class 3] |
| PA14_34740 | 22 |  | hypothetical protein | Hypothetical, unclassified, unknown | Unknown [Class 3] |
| PA14_35010 | 23 | C3719/LESB58/PACS2/PA01/2192/PA7 | hypothetical protein | Hypothetical, unclassified, unknown | Unknown [Class 3] |
| PA14_35030 | 23 |  | hypothetical protein | Hypothetical, unclassified, unknown | Unknown [Class 3] |
| PA14_35040 | 23 |  | hypothetical protein | Hypothetical, unclassified, unknown | Unknown [Class 3] |
| PA14_35050 | 23 |  | hypothetical protein | Putative enzymes | Cytoplasmic Membrane [Class 3] |
| PA14_35060 | 23 |  | hypothetical protein | Hypothetical, unclassified, unknown | Unknown [Class 3] |
| PA14_36480 | 24 | C3719/LESB58/PACS2/PA01/2192/39016/PA7 | hypothetical protein | Hypothetical, unclassified, unknown | Unknown [Class 3] |
| PA14_36490 | 24 |  | hypothetical protein | Hypothetical, unclassified, unknown | Unknown [Class 3] |
| PA14_36500 | 24 |  | hypothetical protein | Carbon compound catabolism | Cytoplasmic [Class 3] |
| PA14_36520 | 24 |  | hypothetical protein | Hypothetical, unclassified, unknown | Cytoplasmic [Class 3] |
| PA14_36760 | 25 | PA3907/PA7 | KU domain-containing protein | DNA replication, recombination, modification and repair | Cytoplasmic [Class 3] |
| PA14_36770 | 25 |  | hypothetical protein | Hypothetical, unclassified, unknown | Unknown [Class 3] |
| PA14_36780 | 25 |  | hypothetical protein | Transport of small molecules | Cytoplasmic Membrane [Class 3] |
| PA14_36790 | 25 |  | hypothetical protein | Hypothetical, unclassified, unknown | Unknown [Class 3] |
| PA14_36810 | 25 |  | hydroperoxidase II | Adaptation, Protection | Cytoplasmic [Class 3] |
| PA14_36820 | 25 |  | hypothetical protein | Hypothetical, unclassified, unknown | Unknown [Class 3] |
| PA14_36870 | 26 |  | putative short-chain dehydrogenase | Central intermediary metabolism | Cytoplasmic [Class 3] |
| PA14_36880 | 26 | C3719/LESB58/PACS2/PA01/PA7 | putative ompetence-damaged protein | Transcriptional regulators | Unknown [Class 3] |
| PA14_36890 | 26 |  | putative metallothionein | Central intermediary metabolism | Unknown [Class 3] |
| PA14_36900 | 26 |  | hypothetical protein | Hypothetical, unclassified, unknown | Cytoplasmic Membrane [Class 3] |
| PA14_36910 | 26 |  | ATP-dependent DNA ligase | DNA replication, recombination, modification and repair | Cytoplasmic [Class 3] |
| PA14_36920 | 26 |  | hypothetical protein | Two-component regulatory systems | Cytoplasmic [Class 3] |
| PA14_37090 | 27 | C3719/LESB58/PACS2/PA01/2192/39016 | putative aldehyde dehydrogenase | Energy metabolism | Cytoplasmic [Class 3] |
| PA14_37100 | 27 |  | putative dehydrogenase | Energy metabolism | Cytoplasmic Membrane [Class 3] |
| PA14_37120 | 27 |  | LysR family transcriptional regulator | Transcriptional regulators | Cytoplasmic [Class 3] |
| PA14_37130 | 27 |  | hypothetical protein | Hypothetical, unclassified, unknown | Cytoplasmic Membrane [Class 3] |
| PA14_37420 | 28 | C3719/LESB58/PACS2/PA01/2192 | putative transmembrane sensor protein | Transport of small molecules | Periplasmic [Class 3] |
| PA14_37430 | 28 |  | RNA polymerase sigma factor | Transcription, RNA processing and degradation | Cytoplasmic [Class 3] |
| PA14_37440 | 29 | C3719/LESB58/PACS2/PA01/2192 | putative MFS transporter | Transport of small molecules | Cytoplasmic Membrane [Class 3] |
| PA14_37460 | 29 |  | putative permease | Transport of small molecules | Cytoplasmic Membrane [Class 3] |
| PA14_37470 | 29 |  | putative flavin-dependent oxidoreductase | Energy metabolism | Cytoplasmic [Class 3] |
| PA14_37510 | 29 |  | hypothetical protein | Hypothetical, unclassified, unknown | Cytoplasmic [Class 3] |
| PA14_37520 | 29 |  | hypothetical protein | Hypothetical, unclassified, unknown | Unknown [Class 3] |
| PA14_37530 | 29 |  | putative hydrolase | Putative enzymes | Extracellular [Class 3] |
| PA14_37550 | 29 |  | putative ring-hydroxylating dioxygenase small subunit | Carbon compound catabolism | Cytoplasmic [Class 3] |
| PA14_37560 | 29 |  | asparagine synthetase, glutamine-hydrolysing | Amino acid biosynthesis and metabolism | Cytoplasmic [Class 3] |
| PA14_37570 | 29 |  | ring-hydroxylating dioxygenase, large terminal subunit | Carbon compound catabolism | Cytoplasmic [Class 3] |
| PA14_39520 | 30 | PA7/LESB58/PACS2/PA01/39016 | putative hydroxylase large subunit | Energy metabolism | Cytoplasmic Membrane [Class 3] |
| PA14_39530 | 30 |  | putative hydroxylase molybdopterin-containing subunit | Energy metabolism | Cytoplasmic [Class 3] |
| PA14_39540 | 30 |  | putative ferredoxin | Energy metabolism | Cytoplasmic [Class 3] |
| PA14_39560 | 30 |  | putative chemotaxis transducer | Chemotaxis | Cytoplasmic Membrane [Class 3] |
| PA14_39990 | 31 | C3719/LESB58/PACS2/PA01/2192/39016/PA7 | hypothetical protein | Fatty acid and phospholipid metabolism | Cytoplasmic Membrane [Class 3] |
| PA14_40010 | 31 |  | hypothetical protein | Hypothetical, unclassified, unknown | Cytoplasmic [Class 3] |
| PA14_40020 | 31 |  | hypothetical protein | Hypothetical, unclassified, unknown | Cytoplasmic Membrane [Class 3] |
| PA14_40030 | 32 | C3719/LESB58/PACS2/2192/39016/PA7 | hypothetical protein | Putative enzymes | Unknown [Class 3] |
| PA14_40040 | 32 |  | hypothetical protein | Central intermediary metabolism | Periplasmic [Class 3] |
| PA14_40050 | 32 |  | hypothetical protein | Hypothetical, unclassified, unknown | Unknown [Class 3] |
| PA14_40060 | 32 |  | hypothetical protein | Fatty acid and phospholipid metabolism | Cytoplasmic Membrane [Class 3] |
| PA14_40100 | 33 | PA01/2192/39016/PA7 | hypothetical protein | Hypothetical, unclassified, unknown | Unknown [Class 3] |
| PA14_40110 | 33 |  | hypothetical protein | Hypothetical, unclassified, unknown | Unknown [Class 3] |
| PA14_44190 | 34 | C3719/LESB58/PACS2/PA01/2192/39016/PA7 | putative sugar MFS transporter | Transport of small molecules | Cytoplasmic Membrane [Class 3] |
| PA14_44200 | 34 |  | hypothetical protein | Translation, post-translational modification, degradation | Cytoplasmic [Class 3] |
| PA14_44210 | 34 |  | putative Glycine/D-amino acid oxidase | Amino acid biosynthesis and metabolism | Cytoplasmic [Class 3] |
| PA14_46340 | 35 | C3719/LESB58/PA01/2192/39016 | hypothetical protein | Hypothetical, unclassified, unknown | Unknown [Class 3] |
| PA14_46360 | 35 |  | putative two-component response regulator | Two-component regulatory systems | Cytoplasmic [Class 3] |
| PA14_46380 | 36 | C3719/LESB58/PA01/2192/39016 | hypothetical protein | Hypothetical, unclassified, unknown | Unknown [Class 3] |
| PA14_46390 | 36 |  | hypothetical protein | Hypothetical, unclassified, unknown | Unknown [Class 3] |
| PA14_46430 | 37 | C3719/PA7/PA01/2192/39016 | hypothetical protein | Hypothetical, unclassified, unknown | Unknown [Class 3] |
| PA14_46440 | 37 |  | putative acetyltransferase | Transport of small molecules | Unknown [Class 3] |
| PA14_46810 | 38 | C3719/LESB58/PACS2/PA01/2192/39016/PA7 | RNA polymerase ECF-subfamily sigma-70 factor | Transcriptional regulators | Cytoplasmic [Class 3] |
| PA14_46820 | 38 |  | hypothetical protein | Hypothetical, unclassified, unknown | Cytoplasmic [Class 3] |
| PA14_47060 | 39 | C3719/LESB58/PACS2/PA01/2192/PA7 | short chain dehydrogenase | Putative enzymes | Cytoplasmic [Class 3] |
| PA14_47070 | 39 |  | hypothetical protein | Hypothetical, unclassified, unknown | Unknown [Class 3] |
| PA14_48170 | 40 | PACS2/PA01/2192/39016 | hypothetical protein | Hypothetical, unclassified, unknown | Unknown [Class 3] |
| PA14_48190 | 40 |  | putative transcriptional regulator | Transcriptional regulators | Cytoplasmic [Class 3] |
| PA14_48330 | 41 | C3719/LESB58/PACS2/PA01/2192/39016/PA7 | hypothetical protein | Hypothetical, unclassified, unknown | Unknown [Class 3] |
| PA14_48340 | 41 |  | hypothetical protein | Membrane proteins | Cytoplasmic Membrane [Class 3] |
| PA14_48350 | 41 |  | hypothetical protein | Membrane proteins | Cytoplasmic Membrane [Class 3] |
| PA14_48380 | 41 |  | hypothetical protein | Hypothetical, unclassified, unknown | Cytoplasmic Membrane [Class 3] |
| PA14_48590 | 42 |  | hypothetical protein | Hypothetical, unclassified, unknown | Cytoplasmic [Class 3] |
| PA14_48600 | 42 | C3719/LESB58/PACS2/PA01/2192/39016 | putative AMP-binding enzyme | Putative enzymes | Cytoplasmic [Class 3] |
| PA14_48610 | 42 |  | putative sparagine synthase | Amino acid biosynthesis and metabolism | Cytoplasmic [Class 3] |
| PA14_48620 | 42 |  | putative clavaminic acid synthetase | Central intermediary metabolism | Unknown [Class 3] |
| PA14_48630 | 42 |  | putative MFS transporter | Transport of small molecules | Cytoplasmic Membrane [Class 3] |
| PA14_48640 | 42 |  | hypothetical protein | Hypothetical, unclassified, unknown | Unknown [Class 3] |
| PA14_49090 | 43 | C3719/LESB58/PACS2/PA01/2192/39016/PA7 | hypothetical protein | Hypothetical, unclassified, unknown | Cytoplasmic [Class 3] |
| PA14_49100 | 43 |  | glutathione S-transferase | Putative enzymes | Cytoplasmic [Class 3] |
| PA14_50640 | 44 | LESB58/PACS2/PA01/2192/39016/PA7 | hypothetical protein | Hypothetical, unclassified, unknown | Unknown [Class 3] |
| PA14_50650 | 44 |  | hypothetical protein | Hypothetical, unclassified, unknown | Cytoplasmic [Class 3] |
| PA14_50660 | 44 |  | hypothetical protein | Putative enzymes | Cytoplasmic [Class 3] |
| PA14_50670 | 44 |  | hypothetical protein | Hypothetical, unclassified, unknown | Cytoplasmic Membrane [Class 3] |
| PA14_50890 | 45 | C3719/LESB58/PACS2/PA01/2192/39016/PA7 | hypothetical protein | Hypothetical, unclassified, unknown | Unknown [Class 3] |
| PA14_50900 | 45 |  | hypothetical protein | Hypothetical, unclassified, unknown | Unknown [Class 3] |
| PA14_50910 | 45 |  | hypothetical protein | Hypothetical, unclassified, unknown | Unknown [Class 3] |
| PA14_51190 | 46 | C3719/PACS2/2192/39016/PA7 | thiolase | Hypothetical, unclassified, unknown | Unknown [Class 3] |
| PA14_51200 | 46 |  | hypothetical protein | Hypothetical, unclassified, unknown | Unknown [Class 3] |
| PA14_52480 | 47 | C3719/LESB58/PA01/2192 | hypothetical protein | Hypothetical, unclassified, unknown | Unknown [Class 3] |
| PA14_52490 | 47 |  | hypothetical protein | Hypothetical, unclassified, unknown | Unknown [Class 3] |
| PA14_52500 | 47 |  | hypothetical protein | Hypothetical, unclassified, unknown | Cytoplasmic Membrane [Class 3] |
| PA14_52510 | 47 |  | hypothetical protein | Hypothetical, unclassified, unknown | Cytoplasmic Membrane [Class 3] |
| PA14_63340 | 48 | C3719/LESB58/PACS2/2192/39016/PA7 | putative lipoprotein | Fatty acid and phospholipid metabolism | Unknown [Class 3] |
| PA14_63350 | 48 |  | GNAT family acetyltransferase | Transport of small molecules | Unknown [Class 3] |
| PA14_63360 | 48 |  | hypothetical protein | Adaptation, Protection | Cytoplasmic [Class 3] |
| PA14_63370 | 48 |  | hypothetical protein | Amino acid biosynthesis and metabolism | Unknown [Class 3] |
| PA14_63380 | 48 |  | hypothetical protein | Hypothetical, unclassified, unknown | Cytoplasmic [Class 3] |
| PA14_63390 | 49 | C3719/39016 | putative adenylate kinase | Nucleotide biosynthesis and metabolism | Unknown [Class 3] |
| PA14_63410 | 49 |  | hypothetical protein | Hypothetical, unclassified, unknown | Cytoplasmic [Class 3] |
| PA14_63420 | 49 |  | hypothetical protein | Hypothetical, unclassified, unknown | Unknown [Class 3] |
| PA14_63430 | 49 |  | Hypothetical protein | Hypothetical, unclassified, unknown | Unknown [Class 3] |
| PA14_63740 | 50 | PACS2/39016 | hypothetical protein | Hypothetical, unclassified, unknown | Unknown [Class 3] |
| PA14_63770 | 50 |  | hypothetical protein | Hypothetical, unclassified, unknown | Unknown [Class 3] |
| PA14_63780 | 50 |  | hypothetical protein | Hypothetical, unclassified, unknown | Unknown [Class 3] |
| PA14_63910 | 51 |  | hypothetical protein | Membrane proteins | Cytoplasmic Membrane [Class 3] |
| PA14_63920 | 51 | C3719/LESB58/PACS2/PA01/2192/39016/PA7 | hypothetical protein | Hypothetical, unclassified, unknown | Unknown [Class 3] |
| PA14_63940 | 51 |  | hypothetical protein | Hypothetical, unclassified, unknown | Cytoplasmic [Class 3] |
| PA14_63960 | 51 |  | putative uter membrane protein precursor | Membrane proteins | Outer Membrane [Class 3] |
| PA14_71330 | 52 | C3719/LESB58/PACS2/PA01/2192/39016/PA7 | putative transcriptional regulator | Transcriptional regulators | Cytoplasmic [Class 3] |
| PA14_71340 | 52 |  | hypothetical protein | Hypothetical, unclassified, unknown | Unknown [Class 3] |
| PA14_71350 | 52 |  | hypothetical protein | Hypothetical, unclassified, unknown | Unknown [Class 3] |
| PA14_71360 | 52 |  | hypothetical protein | Hypothetical, unclassified, unknown | Unknown [Class 3] |
| PA14_71370 | 52 |  | hypothetical protein | Hypothetical, unclassified, unknown | Unknown [Class 3] |
| PA14_71380 | 53 | C3719/LESB58/PACS2/PA01/2192/39016/PA7 | hypothetical protein | Hypothetical, unclassified, unknown | Unknown [Class 3] |
| PA14_71390 | 53 |  | hypothetical protein | Membrane proteins | Cytoplasmic Membrane [Class 3] |
