# Supplementary material for: A Comprehensive Analysis of In Vitro and In Vivo Genetic Fitness of Pseudomonas aeruginosa Using High-Throughput Sequencing of Transposon Libraries
Source: PLoS Pathog. 2013 Sep 5;9(9):e1003582. doi: 10.1371/journal.ppat.1003582 (PMC3764216; doi:10.1371/journal.ppat.1003582)
Supplement: Table S8 — Tn-insertions in genes within operons of unannotated genes found in all sequenced P. aeruginosa strains and in other bacteria that have a reduce fitness for colonization. (DOC) [file ppat.1003582.s019.doc]

| Table S8: Tn-insertions in genes within operons of unannotated genes found in all sequenced *P. aeruginosa* strains and in other bacteria that have a reduce fitness for colonization | | | | | | |
| --- | --- | --- | --- | --- | --- | --- |
| ID | Operon number | Orthologs in other *P. aeruginosa* strains (www.pseudomonas.com) | Orthologs in other bacteria | Product Name | Functional Class | Subcellular Localization |
| PA14_01160 | 1 | C3719/LESB58/PACS2/PA01 | *P. fulva* | hypothetical protein | Hypothetical, unclassified, unknown | Cytoplasmic [Class 3] |
| PA14_01170 | 1 |  | *P. mendocina* | hypothetical protein | Hypothetical, unclassified, unknown | Unknown [Class 3] |
| PA14_01180 | 1 |  | *P. fluorescens* | hypothetical protein | Hypothetical, unclassified, unknown | Cytoplasmic [Class 3] |
| PA14_01190 | 1 |  | *P. brassicacearum* | 3-oxoacyl-(acyl carrier protein) synthase | Fatty acid and phospholipid metabolism | Cytoplasmic [Class 3] |
| PA14_01200 | 1 |  |  | Hypothetical protein | Hypothetical, unclassified, unknown | Unknown [Class 3] |
| PA14_01230 | 1 |  |  | Hypothetical protein | Hypothetical, unclassified, unknown | Cytoplasmic Membrane [Class 3] |
| PA14_01510 | 2 | C3719/LESB58/PACS2/PA01/ PA7/2192/39016 | *P. syringae* | hypothetical protein | Hypothetical, unclassified, unknown | Unknown [Class 3] |
| PA14_01520 | 2 |  | *P. brassicacearum* | hypothetical protein | Transcriptional regulators | Unknown [Class 3] |
| PA14_01670 | 3 | LESB58/PACS2/PA01/PA7/2192 | *P. syringae* | putative ATP-binding component of ABC transporter | Transport of small molecules | Cytoplasmic Membrane [Class 3] |
| PA14_01680 | 3 |  | *P. brassicacearum* | putative permease of ABC transporter | Transport of small molecules | Cytoplasmic Membrane [Class 3] |
| PA14_01690 | 3 |  | *P. putida/mendocina/ fluorescens* | putative permease of ABC transporter | Transport of small molecules | Cytoplasmic Membrane [Class 3] |
| PA14_01840 | 4 | C3719/LESB58/PACS2/PA01/ PA7/2192 | *P. brassicacearum/fulva/ entomophila* | RNA polymerase ECF-subfamily sigma-70 factor | Transcriptional regulators | Cytoplasmic [Class 3] |
| PA14_01860 | 4 |  | *P. putida/mendocina/ fluorescens* | putative transmembrane sensor | Transport of small molecules | Periplasmic [Class 3] |
| PA14_02930 | 5 | C3719/LESB58/PACS2/PA01/ 2192/39016 | *P. putida/mendocina/ fluorescens* | putative oxidoreductase | Putative enzymes | Periplasmic [Class 3] |
| PA14_02960 | 5 |  | *P. fulva* | hypothetical protein | Hypothetical, unclassified, unknown | Cytoplasmic [Class 3] |
| PA14_04370 | 6 | C3719/LESB58/PACS2/PA01/ PA7/2192/39016 | *P. stuzeri* | putative MFS transporter | Transport of small molecules | Cytoplasmic Membrane [Class 3] |
| PA14_04380 | 6 |  |  | hypothetical protein | Putative enzymes | Cytoplasmic [Class 3] |
| PA14_04780 | 7 | C3719/LESB58/PACS2/PA01/ PA7/2192 | *P. brassicacearum/stuzeri/ entomophila* | oxidoreductase | Energy metabolism | Unknown [Class 3] |
| PA14_04790 | 7 |  | *P. putida/mendocina/ fluorescens* | hypothetical protein | Hypothetical, unclassified, unknown | Cytoplasmic Membrane [Class 3] |
| PA14_04810 | 7 |  |  | putative aldehyde dehydrogenase | Energy metabolism | Cytoplasmic [Class 3] |
| PA14_06430 | 8 | C3719/LESB58/PACS2/PA01/ PA7/2192/39016 | *P. fluorescens* | hypothetical protein | Putative enzymes | Unknown [Class 3] |
| PA14_06450 | 8 |  |  | acetyl-CoA carboxylase biotin carboxylase subunit | Fatty acid and phospholipid metabolism | Cytoplasmic [Class 3] |
| PA14_06460 | 8 |  |  | hypothetical protein | Putative enzymes | Cytoplasmic [Class 3] |
| PA14_06480 | 8 |  |  | putative hydrolase | Putative enzymes | Cytoplasmic [Class 3] |
| PA14_12740 | 9 | C3719/LESB58/PACS2/PA01/PA7/2192/39016 | *P. brassicacearum/ entomophina/ fluorescens* | hypothetical protein | Hypothetical, unclassified, unknown | Unknown [Class 3] |
| PA14_12750 | 9 |  |  | hypothetical protein | Hypothetical, unclassified, unknown | Unknown [Class 3] |
| PA14_13580 | 10 | C3719/LESB58/PACS2/PA01/PA7/2192/39016 | *P. brassicacearum/fulva/*  *entomophila* | ABC transporter ATP-binding protein | Transport of small molecules | Cytoplasmic Membrane [Class 3] |
| PA14_13590 | 10 |  | *P. putida/fluorescens /syringae* | ABC transporter permease | Transport of small molecules | Cytoplasmic Membrane [Class 3] |
| PA14_13600 | 10 |  |  | putative binding protein component of ABC transporter | Transport of small molecules | Cytoplasmic Membrane [Class 3] |
| PA14_13610 | 10 |  |  | ABC transporter permease | Transport of small molecules | Cytoplasmic Membrane [Class 3] |
| PA14_19350 | 11 | C3719/LESB58/PACS2/PA01/PA7/2192/39016 | *P. brassicacearum/fulva/*  *entomophila* | hypothetical protein | Carbon compound catabolism | Cytoplasmic [Class 3] |
| PA14_19360 | 11 |  | *P. putida/fluorescens /syringae/mendocina/stuzeri* | GNAT family acetyltransferase | Putative enzymes | Cytoplasmic [Class 3] |
| PA14_21930 | 12 |  | *P. putida/fluorescens / syringae/mendocina* | putative permease of ABC transporter | Transport of small molecules | Cytoplasmic Membrane [Class 3] |
| PA14_21940 | 12 |  |  | hypothetical protein | Hypothetical, unclassified, unknown | Unknown [Class 3] |
| PA14_21960 | 12 |  |  | hypothetical protein | Transport of small molecules | Unknown [Class 3] |
| PA14_28200 | 13 | C3719/LESB58/PACS2/PA01/2192/39016 | *P. brassicacearum/fluorescens* | hypothetical protein | Hypothetical, unclassified, unknown | Unknown [Class 3] |
| PA14_28210 | 13 |  |  | hypothetical protein | Hypothetical, unclassified, unknown | Cytoplasmic Membrane [Class 3] |
| PA14_29680 | 14 | C3719/LESB58/PACS2/PA01/PA7/39016 | *P. mendocina* | hypothetical protein | Hypothetical, unclassified, unknown | Unknown [Class 3] |
| PA14_29690 | 14 |  |  | hypothetical protein | Hypothetical, unclassified, unknown | Cytoplasmic [Class 3] |
| PA14_29800 | 15 | C3719/LESB58/PACS2/PA01/PA7/2192/39016 | *P. fulva/syringae* | putative chemotaxis transducer | Chemotaxis | Cytoplasmic Membrane [Class 3] |
| PA14_29820 | 15 |  |  | hypothetical protein | Transport of small molecules | Cytoplasmic Membrane [Class 3] |
| PA14_31840 | 16 | C3719/LESB58/PACS2/PA01/PA7/2192/39016 | *P. brassicacearum/fulva/ entomophila* | hypothetical protein | Putative enzymes | Unknown [Class 3] |
| PA14_31850 | 16 |  | *P. putida/fluorescens /syringae/mendocina/ stuzeri* | hypothetical protein | Putative enzymes | Cytoplasmic [Class 3] |
| PA14_33570 | 17 | C3719/PACS2/PA01/PA7/  2192/39016 | *P. brassicacearum/fulva/ entomophila* | hypothetical protein | Hypothetical, unclassified, unknown | Unknown [Class 3] |
| PA14_33580 | 17 |  | *P. putida/fluorescens /syringae/mendocina* | hypothetical protein | Hypothetical, unclassified, unknown | Unknown [Class 3] |
| PA14_33590 | 17 |  |  | hypothetical protein | Hypothetical, unclassified, unknown | Cytoplasmic Membrane [Class 3] |
| PA14_33600 | 17 |  |  | hypothetical protein | Membrane proteins | Cytoplasmic Membrane [Class 3] |
| PA14_36540 | 18 | C3719/LESB58/PACS2/PA01/2192/39016/PA7 | *P. brassicacearum/ putida/fluorescens/mendocina* | hypothetical protein | Putative enzymes | Cytoplasmic [Class 3] |
| PA14_36550 | 18 |  |  | hypothetical protein | Hypothetical, unclassified, unknown | Cytoplasmic [Class 3] |
| PA14_39220 | 19 | C3719/LESB58/PACS2/2192/39016 | *P. brassicacearum/ putida/fluorescens/ entomophila* | hypothetical protein | Hypothetical, unclassified, unknown | Extracellular [Class 3] |
| PA14_39240 | 19 |  |  | hypothetical protein | Hypothetical, unclassified, unknown | Unknown [Class 3] |
| PA14_39250 | 19 |  |  | putative double-glycine peptidase | Putative enzymes | Unknown [Class 3] |
| PA14_39260 | 19 |  |  | hypothetical protein | Hypothetical, unclassified, unknown | Extracellular [Class 3] |
| PA14_40600 | 20 | C3719/LESB58/PACS2/PA01/2192/39016/PA7 | *P. fulva* | putative transcriptional regulator | Transcriptional regulators | Cytoplasmic [Class 3] |
| PA14_40610 | 20 |  |  | hypothetical protein | Hypothetical, unclassified, unknown | Unknown [Class 3] |
| PA14_40620 | 20 |  |  | putative MFS transporter | Transport of small molecules | Cytoplasmic Membrane [Class 3] |
| PA14_41110 | 21 | C3719/LESB58/PACS2/PA01/2192/39016/PA7 | *P. brassicacearum/stuzeri/*  *fluorescens /syringae/ mendocina* | putative solute-binding protein | Transport of small molecules | Periplasmic [Class 3] |
| PA14_41130 | 21 |  |  | putative binding protein component of ABC transporter | Transport of small molecules | Periplasmic [Class 3] |
| PA14_41140 | 21 |  |  | ABC transporter permease | Transport of small molecules | Cytoplasmic Membrane [Class 3] |
| PA14_41150 | 21 |  |  | putative permease of ABC transporter | Transport of small molecules | Cytoplasmic Membrane [Class 3] |
| PA14_41160 | 21 |  |  | putative ATP-binding component of ABC transporter | Transport of small molecules | Cytoplasmic Membrane [Class 3] |
| PA14_41170 | 21 |  |  | NADH-dependent enoyl-ACP reductase | Fatty acid and phospholipid metabolism | Cytoplasmic Membrane [Class 3] |
| PA14_43110 | 22 | C3719/LESB58/PACS2/PA01/2192/39016/PA7 | *P. syringae/fulva* | putative glutathione S-transferase | Central intermediary metabolism | Cytoplasmic [Class 3] |
| PA14_43130 | 22 |  | *P. mendocina/fluorescens/*  *stuzeri* | putative aminotransferase | Transport of small molecules | Cytoplasmic [Class 3] |
| PA14_43580 | 23 | C3719/LESB58/PACS2/PA01/2192/39016/PA7 | *P. mendocina* | putative transcriptional regulator | Transcriptional regulators | Cytoplasmic [Class 3] |
| PA14_43600 | 23 |  |  | hypothetical protein | Fatty acid and phospholipid metabolism | Unknown [Class 3] |
| PA14_45120 | 24 | C3719/LESB58/PACS2/PA01/2192/39016/PA7 | *P. fluorescens* | hypothetical protein | Hypothetical, unclassified, unknown | Cytoplasmic [Class 3] |
| PA14_45130 | 24 |  |  | putative transporter | Transport of small molecules | Cytoplasmic Membrane [Class 3] |
| PA14_45400 | 25 | C3719/LESB58/PACS2/PA01/2192/39016/PA7 | *P. syringae/brassicacearum/*  *fulva/entomophila* | hypothetical protein | Hypothetical, unclassified, unknown | Unknown [Class 3] |
| PA14_45410 | 25 |  | *P. putida/mendocina/*  *fluorescens/stuzeri* | FlhB domain-containing protein | Motility & Attachment | Unknown [Class 3] |
| PA14_46920 | 26 | C3719/LESB58/PACS2/PA01/2192/39016/PA7 | *P. syringae/brassicacearum/fulva/entomophila* | amino acid ABC transporter membrane protein | Transport of small molecules | Cytoplasmic Membrane [Class 3] |
| PA14_46930 | 26 |  | *P. putida/mendocina/fluorescens* | amino acid ABC transporter membrane protein | Transport of small molecules | Cytoplasmic Membrane [Class 3] |
| PA14_46950 | 26 |  |  | amino acid ABC transporter ATP binding protein | Transport of small molecules | Cytoplasmic Membrane [Class 3] |
| PA14_46980 | 27 | LESB58/PACS2/PA01/2192/39016/PA7 | *P. syringae/brassicacearum/fulva/entomophila* | putative two-component sensor | Two-component regulatory systems | Cytoplasmic Membrane [Class 3] |
| PA14_46990 | 27 |  | *P. putida/mendocina/*  *fluorescens* | putative two-component response regulator | Two-component regulatory systems | Cytoplasmic [Class 3] |
| PA14_47920 | 28 | C3719/LESB58/PACS2/PA01/2192/PA7 | *P. brassicacaerum* | amino acid ABC transporter periplasmic binding protein | Transport of small molecules | Periplasmic [Class 3] |
| PA14_47930 | 28 |  |  | hypothetical protein | Hypothetical, unclassified, unknown | Cytoplasmic [Class 3] |
| PA14_47940 | 28 |  |  | putative permease of ABC transporter | Transport of small molecules | Cytoplasmic Membrane [Class 3] |
| PA14_47950 | 28 |  |  | amino acid ABC transporter membrane protein | Transport of small molecules | Cytoplasmic Membrane [Class 3] |
| PA14_47960 | 28 |  |  | amino acid ABC transporter ATP binding protein | Transport of small molecules | Cytoplasmic Membrane [Class 3] |
| PA14_47970 | 28 |  |  | hypothetical protein | Central intermediary metabolism | Cytoplasmic [Class 3] |
| PA14_48000 | 28 |  |  | putative dihydrodipicolinate synthetase | Amino acid biosynthesis and metabolism | Cytoplasmic [Class 3] |
| PA14_48010 | 28 |  |  | putative semialdehyde dehydrogenase | Putative enzymes | Cytoplasmic [Class 3] |
| PA14_48780 | 29 | C3719/LESB58/PACS2/PA01/2192/39016 | *P. fluoresens/mendocina* | hypothetical protein | Transport of small molecules | Cytoplasmic [Class 3] |
| PA14_48790 | 29 |  |  | putative lipoprotein | Cell wall / LPS / capsule | Unknown [Class 3] |
| PA14_49780 | 30 | C3719/LESB58/PACS2/PA01/2192/39016 | *P. brassicacaerum* | fosfomycin resistance protein | Antibiotic resistance and susceptibility | Cytoplasmic [Class 3] |
| PA14_49790 | 30 |  |  | transcriptional regulator | Transcriptional regulators | Cytoplasmic [Class 3] |
| PA14_52850 | 31 |  |  | acyl-CoA lyase beta chain | Putative enzymes | Cytoplasmic [Class 3] |
| PA14_52870 | 31 | C3719/LESB58/PACS2/2192/39016/PA7 | *P. stuzeri* | hypothetical protein | Putative enzymes | Cytoplasmic [Class 3] |
| PA14_52880 | 31 |  |  | hypothetical protein | Putative enzymes | Cytoplasmic [Class 3] |
| PA14_52890 | 31 |  |  | ring-cleaving dioxygenase | Putative enzymes | Cytoplasmic [Class 3] |
| PA14_52900 | 31 |  |  | acyl-CoA dehydrogenase | Putative enzymes | Cytoplasmic [Class 3] |
| PA14_52910 | 31 |  |  | hypothetical protein | Hypothetical, unclassified, unknown | Cytoplasmic [Class 3] |
| PA14_54090 | 32 | C3719/LESB58/PACS2/PA01/2192/39016/PA7 | *P. fluorescens* | hypothetical protein | Putative enzymes | Cytoplasmic [Class 3] |
| PA14_54110 | 32 |  |  | transporter | Transport of small molecules | Cytoplasmic Membrane [Class 3] |
| PA14_54120 | 32 |  |  | acyl carrier protein phosphodiesterase | Putative enzymes | Cytoplasmic Membrane [Class 3] |
| PA14_54640 | 33 | C3719/LESB58/PACS2/PA01/2192/PA7 | *P. mendocina* | enoyl-CoA hydratase | Putative enzymes | Cytoplasmic [Class 3] |
| PA14_54660 | 33 |  |  | enoyl-CoA hydratase/isomerase | Putative enzymes | Cytoplasmic [Class 3] |
| PA14_54670 | 33 |  |  | putative 3-hydroxyisobutyrate dehydrogenase | Carbon compound catabolism | Cytoplasmic [Class 3] |
| PA14_56560 | 34 | C3719/LESB58/PACS2/PA01/2192/39016/PA7 | *P. syringae/brassicacearum/fulva/entomophila* | Putative hemolysin | Secreted Factors (toxins, enzymes, alginate) | Cytoplasmic [Class 3] |
| PA14_56570 | 34 |  | *P. putida/mendocina/fluorescens* | putative acyltransferase | Fatty acid and phospholipid metabolism | Cytoplasmic Membrane [Class 3] |
| PA14_61340 | 35 | C3719/LESB58/PACS2/39016/PA7 | *P. syringae/brassicacearum/fulva/entomophila* | hypothetical protein | Hypothetical, unclassified, unknown | Unknown [Class 3] |
| PA14_61350 | 35 |  | *P. putida/mendocina/fluorescens* | hypothetical protein | Hypothetical, unclassified, unknown | Unknown [Class 3] |
| PA14_61910 | 36 | C3719/LESB58/PACS2/PA01/2192/39016/PA7 | *P. mendocina* | hypothetical protein | Hypothetical, unclassified, unknown | Unknown [Class 3] |
| PA14_61920 | 36 |  |  | hypothetical protein | Hypothetical, unclassified, unknown | Unknown [Class 3] |
| PA14_61940 | 36 |  |  | hypothetical protein | Hypothetical, unclassified, unknown | Unknown [Class 3] |
| PA14_62180 | 37 | C3719/LESB58/PACS2/PA01/2192/39016/PA7 | *P. syringae/brassicacearum/ fulva/entomophila* | hypothetical protein | Hypothetical, unclassified, unknown | Unknown [Class 3] |
| PA14_62190 | 37 |  | *P. putida/mendocina/ fluorescens/stuzeri* | hypothetical protein | Hypothetical, unclassified, unknown | Extracellular [Class 3] |
| PA14_62660 | 38 | C3719/LESB58/PACS2/PA01/2192/39016/PA7 | *P. fulva/mendocina/stuzeri* | hypothetical protein | Hypothetical, unclassified, unknown | Unknown [Class 3] |
| PA14_62670 | 38 |  |  | hypothetical protein | Hypothetical, unclassified, unknown | Unknown [Class 3] |
| PA14_64000 | 39 |  |  | translation initiation factor Sui1 | Transcription, RNA processing and degradation | Unknown [Class 3] |
| PA14_64010 | 39 | C3719/LESB58/PACS2/PA01/2192/39016/PA7 | *P. syringae/brassicacearum/ fulva* | hypothetical protein | Fatty acid and phospholipid metabolism | Unknown [Class 3] |
| PA14_64030 | 39 |  | *P. putida/mendocina/fluorescens* | hypothetical protein | Hypothetical, unclassified, unknown | Unknown [Class 3] |
| PA14_64610 | 40 | C3719/LESB58/PACS2/PA01/2192/PA7 | *P. mendocina* | hypothetical protein | Fatty acid and phospholipid metabolism | Unknown [Class 3] |
| PA14_64620 | 40 |  |  | putative oxidoreductase | Energy metabolism | Unknown [Class 3] |
| PA14_68080 | 41 | C3719/LESB58/PACS2/PA01/2192/39016/PA7 | *P. syringae/brassicacearum/fulva/entomophila* | ABC transporter permease | Transport of small molecules | Cytoplasmic Membrane [Class 3] |
| PA14_68090 | 41 |  | *P. putida/mendocina/fluorescens/stuzeri* | amino acid ABC transporter membrane protein | Transport of small molecules | Cytoplasmic Membrane [Class 3] |
| PA14_69580 | 42 | C3719/LESB58/PACS2/PA01/2192/39016/PA7 | *P. entomophila/mendocina/ fulva* | hypothetical protein | Hypothetical, unclassified, unknown | Unknown [Class 3] |
| PA14_69590 | 42 |  |  | putative ABC-type amino acid transporter | Transport of small molecules | Unknown [Class 3] |
| PA14_70480 | 43 | C3719/LESB58/PACS2/PA01/2192/39016/PA7 | *P. syringae/brassicacearum/ entomophila* | hypothetical protein | Translation, post-translational modification, degradation | Cytoplasmic [Class 3] |
| PA14_70490 | 43 |  | *P. putida/mendocina/fluorescens* | putative lipoprotein | Fatty acid and phospholipid metabolism | Unknown [Class 3] |
| PA14_71000 | 43 |  |  | putative lycine betaine/L-proline ABC transporter, ATP-binding subunit | Transport of small molecules | Cytoplasmic Membrane [Class 3] |
| PA14_71020 | 44 | C3719/LESB58/PACS2/PA01/2192/39016/PA7 | *P. syringae/brassicacearum/ entomophila* | putative BC-type proline/glycine betaine transport system, permease component | Transport of small molecules | Cytoplasmic Membrane [Class 3] |
| PA14_71030 | 44 |  | *P. putida/mendocina/ fluorescens* | hypothetical protein | Transport of small molecules | Periplasmic [Class 3] |
| PA14_71190 | 45 | C3719/LESB58/PACS2/ PA01/2192/39016/PA7 | *P. brassicacearum/fluorescens* | hypothetical protein | Hypothetical, unclassified, unknown | Unknown [Class 3] |
| PA14_71200 | 45 |  |  | YjgF family translation initiation inhibitor | Translation, post-translational modification, degradation | Unknown [Class 3] |
| PA14_71210 | 45 |  |  | hypothetical protein | Transport of small molecules | Cytoplasmic [Class 3] |
| PA14_71760 | 46 | 39016/PACS2/PA7 | *P. brassicacearum/entomophila* | hypothetical protein | Hypothetical, unclassified, unknown | Unknown [Class 3] |
| PA14_71780 | 46 |  | *P. putida/stuzeri* | RpiR family transcriptional regulator | Transcriptional regulators | Cytoplasmic [Class 3] |
| PA14_72090 | 47 | C3719/LESB58/PACS2/PA01/2192/39016/PA7 | *P. syringae/brassicacearum/fulva/entomophila* | hypothetical protein | Hypothetical, unclassified, unknown | Unknown [Class 3] |
| PA14_72110 | 47 |  | *P. putida/mendocina/fluorescens/stuzeri* | hypothetical protein | Hypothetical, unclassified, unknown | Unknown [Class 3] |
| PA14_72650 | 48 | C3719/LESB58/PACS2/PA01/2192/39016/PA7 | *P. sbrassicacearum/fluorescens* | hypothetical protein | Transcriptional regulators | Cytoplasmic [Class 3] |
| PA14_72660 | 48 |  |  | hypothetical protein | Biosynthesis of cofactors, prosthetic groups and carriers | Cytoplasmic [Class 3] |
| PA14_72690 | 48 |  |  | putative glutamine synthetase | Amino acid biosynthesis and metabolism | Cytoplasmic [Class 3] |
| PA14_72700 | 48 |  |  | hypothetical protein | Biosynthesis of cofactors, prosthetic groups and carriers | Cytoplasmic [Class 3] |
| PA14_72710 | 48 |  |  | putative transporter | Transport of small molecules | Cytoplasmic Membrane [Class 3] |
| PA14_72850 | 49 | C3719/LESB58/PACS2/ PA01/2192/39016/PA7 | *P. fulva/fluorescens* | putative glutamine synthetase | Amino acid biosynthesis and metabolism | Cytoplasmic [Class 3] |
| PA14_72870 | 49 |  |  | aminotransferase | Biosynthesis of cofactors, prosthetic groups and carriers | Cytoplasmic [Class 3] |
| PA14_73000 | 50 | C3719/LESB58/PACS2/ PA01/39016/PA7 | *P. fulva/fluorescens/mendocina/ stuzeri* | hypothetical protein | Hypothetical, unclassified, unknown | Cytoplasmic [Class 3] |
| PA14_73010 | 50 |  |  | hypothetical protein | Biosynthesis of cofactors, prosthetic groups and carriers | Cytoplasmic [Class 3] |
| PA14_73020 | 50 |  |  | DksA/TraR family C4-type zinc finger protein | Two-component regulatory systems | Cytoplasmic [Class 3] |
| PA14_73100 | 51 | 2192/LESB58/PACS2/PA01/39016/PA7 | *P. fulva/brassicacaerum/ mendocina/stuzeri/putida* | hypothetical protein | Hypothetical, unclassified, unknown | Unknown [Class 3] |
| PA14_73110 | 51 |  |  | hypothetical protein | Transport of small molecules | Cytoplasmic Membrane [Class 3] |
