# Supplementary material for: A Comprehensive Analysis of In Vitro and In Vivo Genetic Fitness of Pseudomonas aeruginosa Using High-Throughput Sequencing of Transposon Libraries
Source: PLoS Pathog. 2013 Sep 5;9(9):e1003582. doi: 10.1371/journal.ppat.1003582 (PMC3764216; doi:10.1371/journal.ppat.1003582)
Supplement: Table S9 — Tn-insertions into genes of P. aeruginosa strain PA14 capable of systemic dissemination in neutropenic mice. (DOC) [file ppat.1003582.s020.doc]

| Table S9: Tn-insertions in *P. aeruginosa* strain PA14 genes capable of systemic dissemination in neutropenic mice | | | | |
| --- | --- | --- | --- | --- |
| ID | Gene name | Product Name | Function Class | Subcellular Localization |
| PA14_01710 | ahpC | alkyl hydroperoxide reductase subunit C | Adaptation, Protection | Cytoplasmic [Class 3] |
| PA14_69610 | cyaA | adenylate cyclase | Adaptation, Protection | Cytoplasmic [Class 3] |
| PA14_56220 | PA14_56220 | hypothetical protein | Adaptation, Protection | Cytoplasmic [Class 3] |
| PA14_66460 | PA14_66460 | hypothetical protein | Adaptation, Protection | Cytoplasmic [Class 3] |
| PA14_64460 | psiF | hypothetical protein | Adaptation, Protection | Periplasmic [Class 3] |
| PA14_33650 | pvdD | pyoverdine synthetase D | Adaptation, Protection | Cytoplasmic [Class 3] |
| PA14_33280 | pvdL | peptide synthase | Adaptation, Protection | Unknown [Class 3] |
| PA14_58000 | sodM | superoxide dismutase | Adaptation, Protection | Periplasmic [Class 3] |
| PA14_66290 | aceE | pyruvate dehydrogenase subunit E1 | Amino acid biosynthesis and metabolism | Cytoplasmic [Class 3] |
| PA14_68340 | arcB | ornithine carbamoyltransferase | Amino acid biosynthesis and metabolism | Cytoplasmic [Class 3] |
| PA14_25980 | aroF | phospho-2-dehydro-3-deoxyheptonate aldolase | Amino acid biosynthesis and metabolism | Unknown [Class 3] |
| PA14_19370 | asnB | asparagine synthetase, glutamine-hydrolysing | Amino acid biosynthesis and metabolism | Cytoplasmic [Class 3] |
| PA14_19370 | asnB | putative asparagine synthetase, glutamine-hydrolyzing | Amino acid biosynthesis and metabolism | Cytoplasmic [Class 3] |
| PA14_68850 | gcvP1 | glycine dehydrogenase | Amino acid biosynthesis and metabolism | Cytoplasmic [Class 3] |
| PA14_57770 | hisC1 | histidinol-phosphate aminotransferase | Amino acid biosynthesis and metabolism | Cytoplasmic [Class 3] |
| PA14_67920 | hisH | imidazole glycerol phosphate synthase subunit HisH | Amino acid biosynthesis and metabolism | Cytoplasmic [Class 3] |
| PA14_53070 | hpd | 4-hydroxyphenylpyruvate dioxygenase | Amino acid biosynthesis and metabolism | Cytoplasmic [Class 3] |
| PA14_23750 | leuC | isopropylmalate isomerase large subunit | Amino acid biosynthesis and metabolism | Cytoplasmic [Class 3] |
| PA14_39590 | metE | 5-methyltetrahydropteroyltriglutamate-- homocysteine S-methyltransferase | Amino acid biosynthesis and metabolism | Cytoplasmic [Class 3] |
| PA14_02730 | PA14_02730 | putative dihydrodipicolinate synthetase | Amino acid biosynthesis and metabolism | Cytoplasmic [Class 3] |
| PA14_03000 | PA14_03000 | hypothetical protein | Amino acid biosynthesis and metabolism | Cytoplasmic [Class 3] |
| PA14_05250 | PA14_05250 | pyrimidine ribonucleotide biosynthetic proces | Amino acid biosynthesis and metabolism | Cytoplasmic [Class 3] |
| PA14_44240 | PA14_44240 | putative glutamine synthetase | Amino acid biosynthesis and metabolism | Cytoplasmic [Class 3] |
| PA14_48470 | PA14_48470 | putative N-carbamoylputrescine amidohydrolase | Amino acid biosynthesis and metabolism | Cytoplasmic [Class 3] |
| PA14_65795 | PA14_65795 | hypothetical protein | Amino acid biosynthesis and metabolism | Cytoplasmic [Class 3] |
| PA14_06190 | psfA | putative glutathione S-transferase | Amino acid biosynthesis and metabolism | Cytoplasmic [Class 3] |
| PA14_54170 | putA | bifunctional proline dehydrogenase/pyrroline-5-carboxylate dehydrogenase | Amino acid biosynthesis and metabolism | Cytoplasmic [Class 3] |
| PA14_60830 | mexD | multidrug efflux RND transporter MexD | Antibiotic resistance and susceptibility | Cytoplasmic Membrane [Class 3] |
| PA14_16820 | PA14_16820 | putative efflux transmembrane protein | Antibiotic resistance and susceptibility | Cytoplasmic Membrane [Class 3] |
| PA14_38395 | PA14_38395 | periplasmic multidrug efflux lipoprotein precursor | Antibiotic resistance and susceptibility | Cytoplasmic Membrane [Class 3] |
| PA14_41280 | PA14_41280 | putative beta-lactamase | Antibiotic resistance and susceptibility | Cytoplasmic Membrane [Class 3] |
| PA14_45890 | PA14_45890 | putative RND efflux transporter | Antibiotic resistance and susceptibility | Cytoplasmic Membrane [Class 3] |
| PA14_57160 | apbA | 2-dehydropantoate 2-reductase | Biosynthesis of cofactors, prosthetic groups and carriers | Cytoplasmic [Class 3] |
| PA14_06500 | bioB | biotin synthase | Biosynthesis of cofactors, prosthetic groups and carriers | Cytoplasmic [Class 3] |
| PA14_47690 | cobQ | cobyric acid synthase | Biosynthesis of cofactors, prosthetic groups and carriers | Cytoplasmic [Class 3] |
| PA14_62850 | folP | dihydropteroate synthase | Biosynthesis of cofactors, prosthetic groups and carriers | Cytoplasmic [Class 3] |
| PA14_20140 | fpr | ferredoxin--NADP+ reductase | Biosynthesis of cofactors, prosthetic groups and carriers | Cytoplasmic [Class 3] |
| PA14_68730 | gshA | glutamate--cysteine ligase | Biosynthesis of cofactors, prosthetic groups and carriers | Cytoplasmic [Class 3] |
| PA14_61580 | hemH | ferrochelatase | Biosynthesis of cofactors, prosthetic groups and carriers | Cytoplasmic [Class 3] |
| PA14_14740 | iscU | scaffold protein | Biosynthesis of cofactors, prosthetic groups and carriers | Cytoplasmic [Class 3] |
| PA14_24890 | mobA | molybdopterin-guanine dinucleotide biosynthesis protein MobA | Biosynthesis of cofactors, prosthetic groups and carriers | Cytoplasmic [Class 3] |
| PA14_51330 | nadA | quinolinate synthetase | Biosynthesis of cofactors, prosthetic groups and carriers | Cytoplasmic [Class 3] |
| PA14_54450 | nadB | L-aspartate oxidase | Biosynthesis of cofactors, prosthetic groups and carriers | Cytoplasmic [Class 3] |
| PA14_03900 | spuC | putative aminotransferase | Biosynthesis of cofactors, prosthetic groups and carriers | Cytoplasmic [Class 3] |
| PA14_69150 | ubiD | hypothetical protein | Biosynthesis of cofactors, prosthetic groups and carriers | Cytoplasmic [Class 3] |
| PA14_32160 | antA | anthranilate dioxygenase large subunit | Carbon compound catabolism | Cytoplasmic [Class 3] |
| PA14_09550 | PA14_09550 | hypothetical protein | Carbon compound catabolism | Periplasmic [Class 3] |
| PA14_03050 | pobA | 4-hydroxybenzoate 3-monooxygenase | Carbon compound catabolism | Cytoplasmic [Class 3] |
| PA14_53480 | pta | phosphate acetyltransferase | Carbon compound catabolism | Cytoplasmic [Class 3] |
| PA14_45030 | ttuD | hypothetical protein | Carbon compound catabolism | Cytoplasmic [Class 3] |
| PA14_61860 | yadF | putative carbonic anhydrase | Carbon compound catabolism | Cytoplasmic [Class 3] |
| PA14_65390 | yjeF | hypothetical protein | Carbon compound catabolism | Unknown [Class 3] |
| PA14_58110 | maf | Maf-like protein | Cell division | Cytoplasmic [Class 3] |
| PA14_67790 | PA14_67790 | putative membrane-bound metallopeptidase | Cell division | Outer Membrane [Class 3] |
| PA14_12100 | dacC | D-ala-D-ala-carboxypeptidase | Cell wall / LPS / capsule | Cytoplasmic Membrane [Class 3] |
| PA14_09600 | ddlA | D-alanine-D-alanine ligase A | Cell wall / LPS / capsule | Cytoplasmic [Class 3] |
| PA14_36840 | glgP | glycogen phosphorylase | Cell wall / LPS / capsule | Cytoplasmic [Class 3] |
| PA14_12280 | lnt | apolipoprotein N-acyltransferase | Cell wall / LPS / capsule | Cytoplasmic Membrane [Class 3] |
| PA14_68120 | opmG | putative outer membrane protein precursor | Cell wall / LPS / capsule | Outer Membrane [Class 3] |
| PA14_08540 | PA14_08540 | hypothetical protein | Cell wall / LPS / capsule | Outer Membrane [Class 3] |
| PA14_23420 | PA14_23420 | putative zinc-binding dehydrogenase | Cell wall / LPS / capsule | Unknown [Class 3] |
| PA14_66160 | PA14_66160 | putative glycosyl transferase | Cell wall / LPS / capsule | Cytoplasmic [Class 3] |
| PA14_23360 | wzz | exopolysaccharide transport protein family | Cell wall / LPS / capsule | Cytoplasmic Membrane [Class 3] |
| PA14_06110 | accC | pyruvate carboxylase subunit A | Central intermediary metabolism | Cytoplasmic [Class 3] |
| PA14_46450 | aceK | bifunctional isocitrate dehydrogenase kinase/phosphatase protein | Central intermediary metabolism | Cytoplasmic [Class 3] |
| PA14_17400 | adhC | alcohol dehydrogenase class III | Central intermediary metabolism | Cytoplasmic [Class 3] |
| PA14_70270 | algC | phosphomannomutase AlgC | Central intermediary metabolism | Unknown [Class 3] |
| PA14_69810 | glnK | nitrogen regulatory protein P-II 2 | Central intermediary metabolism | Cytoplasmic Membrane [Class 3] |
| PA14_67500 | gloA3 | lactoylglutathione lyase | Central intermediary metabolism | Cytoplasmic [Class 3] |
| PA14_17960 | glpK | glycerol kinase | Central intermediary metabolism | Cytoplasmic [Class 3] |
| PA14_36310 | hcnC | hydrogen cyanide synthase HcnC | Central intermediary metabolism | Cytoplasmic [Class 3] |
| PA14_10640 | hpaG2 | hypothetical protein | Central intermediary metabolism | Cytoplasmic [Class 3] |
| PA14_41510 | nasA | assimilatory nitrate reductase | Central intermediary metabolism | Cytoplasmic [Class 3] |
| PA14_27580 | PA14_27580 | putative glutathione S-transferase | Central intermediary metabolism | Cytoplasmic [Class 3] |
| PA14_37340 | PA14_37340 | thiamine pyrophosphate protein | Central intermediary metabolism | Cytoplasmic Membrane [Class 3] |
| PA14_68490 | PA14_68490 | hypothetical protein | Central intermediary metabolism | Cytoplasmic [Class 3] |
| PA14_66840 | phaC2 | poly(3-hydroxyalkanoic acid) synthase 2 | Central intermediary metabolism | Cytoplasmic [Class 3] |
| PA14_05620 | sahH | S-adenosyl-L-homocysteine hydrolase | Central intermediary metabolism | Cytoplasmic [Class 3] |
| PA14_16050 | dsbC | thiol:disulfide interchange protein DsbC | Chaperones & heat shock proteins | Periplasmic [Class 3] |
| PA14_28920 | PA14_28920 | putative chaperone | Chaperones & heat shock proteins | Cytoplasmic [Class 3] |
| PA14_09890 | ppiC2 | peptidyl-prolyl cis-trans isomerase C2 | Chaperones & heat shock proteins | Cytoplasmic [Class 3] |
| PA14_05390 | chpA | ChpA | Chemotaxis | Cytoplasmic [Class 3] |
| PA14_26280 | PA14_26280 | putative chemotaxis transducer | Chemotaxis | Cytoplasmic Membrane [Class 3] |
| PA14_27000 | PA14_27000 | putative chemotaxis transducer | Chemotaxis | Cytoplasmic Membrane [Class 3] |
| PA14_29760 | PA14_29760 | putative chemotaxis transducer | Chemotaxis | Cytoplasmic Membrane [Class 3] |
| PA14_29800 | PA14_29800 | putative chemotaxis transducer | Chemotaxis | Cytoplasmic Membrane [Class 3] |
| PA14_30820 | PA14_30820 | putative methyl-accepting chemotaxis transducer | Chemotaxis | Cytoplasmic Membrane [Class 3] |
| PA14_46030 | PA14_46030 | putative chemotaxis transducer | Chemotaxis | Cytoplasmic Membrane [Class 3] |
| PA14_67010 | PA14_67010 | putative chemotaxis transducer | Chemotaxis | Unknown [Class 3] |
| PA14_56000 | pctA | chemotactic transducer PctA | Chemotaxis | Cytoplasmic Membrane [Class 3] |
| PA14_05340 | pilI | twitching motility protein PilI | Chemotaxis | Cytoplasmic [Class 3] |
| PA14_05360 | pilJ | twitching motility protein PilJ | Chemotaxis | Outer Membrane [Class 3] |
| PA14_42700 | alkA | DNA-3-methyladenine glycosidase II | DNA replication, recombination, modification and repair | Unknown [Class 3] |
| PA14_70390 | crc | catabolite repression control protein | DNA replication, recombination, modification and repair | Cytoplasmic [Class 3] |
| PA14_52310 | dinP | DNA polymerase IV | DNA replication, recombination, modification and repair | Cytoplasmic [Class 3] |
| PA14_17260 | dnaE | DNA polymerase III subunit alpha | DNA replication, recombination, modification and repair | Cytoplasmic [Class 3] |
| PA14_55610 | dnaE2 | error-prone DNA polymerase | DNA replication, recombination, modification and repair | Cytoplasmic [Class 3] |
| PA14_07530 | dnaG | DNA primase | DNA replication, recombination, modification and repair | Cytoplasmic [Class 3] |
| PA14_23260 | gyrA | DNA gyrase subunit A | DNA replication, recombination, modification and repair | Cytoplasmic [Class 3] |
| PA14_21400 | hrpA | putative ATP-dependent helicase | DNA replication, recombination, modification and repair | Cytoplasmic Membrane [Class 3] |
| PA14_70600 | hupA | HU family DNA-binding protein | DNA replication, recombination, modification and repair | Cytoplasmic [Class 3] |
| PA14_21690 | lhr1 | putative ATP-dependent DNA helicase | DNA replication, recombination, modification and repair | Cytoplasmic [Class 3] |
| PA14_44660 | ligA | NAD-dependent DNA ligase LigA | DNA replication, recombination, modification and repair | Cytoplasmic [Class 3] |
| PA14_36910 | ligD | ATP-dependent DNA ligase | DNA replication, recombination, modification and repair | Cytoplasmic [Class 3] |
| PA14_12630 | PA14_12630 | putative ATP-dependent helicase | DNA replication, recombination, modification and repair | Outer Membrane [Class 3] |
| PA14_28810 | PA14_28810 | putative DNA helicase | DNA replication, recombination, modification and repair | Unknown [Class 3] |
| PA14_28840 | PA14_28840 | putative helicase | DNA replication, recombination, modification and repair | Cytoplasmic Membrane [Class 3] |
| PA14_59180 | PA14_59180 | topoisomerase I - like protein | DNA replication, recombination, modification and repair | Cytoplasmic [Class 3] |
| PA14_59540 | PA14_59540 | DEAD-like helicases superfamily | DNA replication, recombination, modification and repair | Cytoplasmic [Class 3] |
| PA14_40120 | polB | DNA polymerase II | DNA replication, recombination, modification and repair | Cytoplasmic [Class 3] |
| PA14_55670 | recB | exodeoxyribonuclease V beta chain | DNA replication, recombination, modification and repair | Unknown [Class 3] |
| PA14_56080 | sbcB | exonuclease I | DNA replication, recombination, modification and repair | Cytoplasmic [Class 3] |
| PA14_55650 | sbcC | putative exonuclease | DNA replication, recombination, modification and repair | Cytoplasmic [Class 3] |
| PA14_00230 | smf | putative Rossmann fold nucleotide-binding protein | DNA replication, recombination, modification and repair | Unknown [Class 3] |
| PA14_25110 | topA | DNA topoisomerase I | DNA replication, recombination, modification and repair | Cytoplasmic [Class 3] |
| PA14_30050 | aceA | isocitrate lyase | Energy metabolism | Cytoplasmic [Class 3] |
| PA14_44290 | acnA | aconitate hydratase | Energy metabolism | Cytoplasmic [Class 3] |
| PA14_73300 | atpE | F0F1 ATP synthase subunit C | Energy metabolism | Cytoplasmic Membrane [Class 3] |
| PA14_13030 | cioA | CioA, cyanide insensitive terminal oxidase | Energy metabolism | Cytoplasmic Membrane [Class 3] |
| PA14_04750 | fdx1 | ferredoxin (4Fe-4S) | Energy metabolism | Unknown [Class 3] |
| PA14_30180 | idh | monomeric isocitrate dehydrogenase | Energy metabolism | Unknown [Class 3] |
| PA14_06830 | norB | nitric-oxide reductase subunit B | Energy metabolism | Cytoplasmic Membrane [Class 3] |
| PA14_29990 | nuoD | bifunctional NADH:ubiquinone oxidoreductase subunit C/D | Energy metabolism | Cytoplasmic [Class 3] |
| PA14_04140 | PA14_04140 | hypothetical protein | Energy metabolism | Cytoplasmic [Class 3] |
| PA14_16260 | PA14_16260 | putative FMN oxidoreductase | Energy metabolism | Cytoplasmic [Class 3] |
| PA14_19900 | PA14_19900 | putative pyruvate dehydrogenase E1 component, alpha subunit | Energy metabolism | Cytoplasmic [Class 3] |
| PA14_29050 | PA14_29050 | putative molybdopterin oxidoreductase | Energy metabolism | Cytoplasmic [Class 3] |
| PA14_32530 | PA14_32530 | putative cytochrome c | Energy metabolism | Periplasmic [Class 3] |
| #N/A | PA14_39640 | Cobalamin biosynthesis protein CobN | Energy metabolism | Unknown [Class 3] |
| PA14_65940 | PA14_65940 | putative oxidoreductase | Energy metabolism | Cytoplasmic [Class 3] |
| PA14_68440 | PA14_68440 | putative oxidoreductase | Energy metabolism | Cytoplasmic [Class 3] |
| PA14_71280 | PA14_71280 | putative ferredoxin | Energy metabolism | Cytoplasmic Membrane [Class 3] |
| PA14_46320 | pyc | putative pyruvate carboxylase | Energy metabolism | Cytoplasmic [Class 3] |
| PA14_18920 | rnfC | electron transport complex protein RnfC | Energy metabolism | Cytoplasmic [Class 3] |
| PA14_17270 | accA | acetyl-CoA carboxylase carboxyltransferase subunit alpha | Fatty acid and phospholipid metabolism | Cytoplasmic [Class 3] |
| PA14_22490 | acpD | azoreductase | Fatty acid and phospholipid metabolism | Cytoplasmic [Class 3] |
| PA14_10370 | choS | hypothetical protein | Fatty acid and phospholipid metabolism | Unknown [Class 3] |
| PA14_41650 | estX | putative esterase | Fatty acid and phospholipid metabolism | Unknown [Class 3] |
| PA14_21540 | fabH-2 | 3-oxoacyl-(acyl carrier protein) synthase III | Fatty acid and phospholipid metabolism | Cytoplasmic [Class 3] |
| PA14_06030 | PA14_06030 | putative acyltransferase | Fatty acid and phospholipid metabolism | Cytoplasmic Membrane [Class 3] |
| PA14_09660 | PA14_09660 | acyl-CoA synthetase | Fatty acid and phospholipid metabolism | Cytoplasmic [Class 3] |
| PA14_13110 | PA14_13110 | long-chain-fatty-acid--CoA ligase | Fatty acid and phospholipid metabolism | Cytoplasmic [Class 3] |
| PA14_19740 | PA14_19740 | enoyl-CoA hydratase | Fatty acid and phospholipid metabolism | Cytoplasmic [Class 3] |
| PA14_28310 | PA14_28310 | putative enoyl-CoA hydratase | Fatty acid and phospholipid metabolism | Cytoplasmic [Class 3] |
| PA14_31720 | PA14_31720 | hypothetical protein | Fatty acid and phospholipid metabolism | Cytoplasmic [Class 3] |
| PA14_36270 | PA14_36270 | putative dehydrogenase | Fatty acid and phospholipid metabolism | Cytoplasmic [Class 3] |
| PA14_40980 | PA14_40980 | enoyl-CoA hydratase | Fatty acid and phospholipid metabolism | Cytoplasmic [Class 3] |
| PA14_61360 | PA14_61360 | hypothetical protein | Fatty acid and phospholipid metabolism | Unknown [Class 3] |
| PA14_66040 | PA14_66040 | putative acyl-CoA dehydrogenase | Fatty acid and phospholipid metabolism | Cytoplasmic [Class 3] |
| PA14_70490 | PA14_70490 | putative lipoprotein | Fatty acid and phospholipid metabolism | Unknown [Class 3] |
| PA14_17860 | paaH | 3-hydroxy-acyl-CoA dehydrogenase | Fatty acid and phospholipid metabolism | Cytoplasmic [Class 3] |
| PA14_17860 | paaH | 3-hydroxy-acyl-CoA dehydrogenase | Fatty acid and phospholipid metabolism | Unknown [Class 3] |
| PA14_17880 | paaJ | acetyl-CoA acetyltransferase | Fatty acid and phospholipid metabolism | Cytoplasmic [Class 3] |
| PA14_54220 | icp | inhibitor of cysteine peptidase | Hypothetical, unclassified, unknown | Unknown [Class 3] |
| PA14_00320 | PA14_00320 | hypothetical protein | Hypothetical, unclassified, unknown | Unknown [Class 3] |
| PA14_00910 | PA14_00910 | hypothetical protein | Hypothetical, unclassified, unknown | Cytoplasmic Membrane [Class 3] |
| PA14_00940 | PA14_00940 | hypothetical protein | Hypothetical, unclassified, unknown | Cytoplasmic [Class 3] |
| PA14_01160 | PA14_01160 | hypothetical protein | Hypothetical, unclassified, unknown | Cytoplasmic [Class 3] |
| PA14_03190 | PA14_03190 | hypothetical protein | Hypothetical, unclassified, unknown | Unknown [Class 3] |
| PA14_03270 | PA14_03270 | hypothetical protein | Hypothetical, unclassified, unknown | Cytoplasmic [Class 3] |
| PA14_03285 | PA14_03285 | hypothetical protein | Hypothetical, unclassified, unknown | Cytoplasmic [Class 3] |
| PA14_03310 | PA14_03310 | hypothetical protein | Hypothetical, unclassified, unknown | Cytoplasmic [Class 3] |
| PA14_03320 | PA14_03320 | Hypothetical protein | Hypothetical, unclassified, unknown | Unknown [Class 3] |
| PA14_04530 | PA14_04530 | hypothetical protein | Hypothetical, unclassified, unknown | Unknown [Class 3] |
| PA14_04790 | PA14_04790 | hypothetical protein | Hypothetical, unclassified, unknown | Cytoplasmic Membrane [Class 3] |
| PA14_07430 | PA14_07430 | hypothetical protein | Hypothetical, unclassified, unknown | Unknown [Class 3] |
| PA14_07500 | PA14_07500 | hypothetical protein | Hypothetical, unclassified, unknown | Cytoplasmic Membrane [Class 3] |
| PA14_10820 | PA14_10820 | HDIG domain-containing protein | Hypothetical, unclassified, unknown | Cytoplasmic [Class 3] |
| PA14_11700 | PA14_11700 | hypothetical protein | Hypothetical, unclassified, unknown | Unknown [Class 3] |
| PA14_11890 | PA14_11890 | hypothetical protein | Hypothetical, unclassified, unknown | Cytoplasmic [Class 3] |
| PA14_11960 | PA14_11960 | hypothetical protein | Hypothetical, unclassified, unknown | Cytoplasmic Membrane [Class 3] |
| PA14_13350 | PA14_13350 | hypothetical protein | Hypothetical, unclassified, unknown | Cytoplasmic [Class 3] |
| PA14_13420 | PA14_13420 | hypothetical protein | Hypothetical, unclassified, unknown | Periplasmic [Class 3] |
| PA14_14000 | PA14_14000 | hypothetical protein | Hypothetical, unclassified, unknown | Unknown [Class 3] |
| PA14_14420 | PA14_14420 | hypothetical protein | Hypothetical, unclassified, unknown | Cytoplasmic Membrane [Class 3] |
| PA14_15360 | PA14_15360 | hypothetical protein | Hypothetical, unclassified, unknown | Unknown [Class 3] |
| PA14_16190 | PA14_16190 | hypothetical protein | Hypothetical, unclassified, unknown | Cytoplasmic Membrane [Class 3] |
| PA14_16330 | PA14_16330 | hypothetical protein | Hypothetical, unclassified, unknown | Unknown [Class 3] |
| PA14_16340 | PA14_16340 | hypothetical protein | Hypothetical, unclassified, unknown | Unknown [Class 3] |
| PA14_17580 | PA14_17580 | hypothetical protein | Hypothetical, unclassified, unknown | Cytoplasmic [Class 3] |
| PA14_18960 | PA14_18960 | hypothetical protein | Hypothetical, unclassified, unknown | Cytoplasmic [Class 3] |
| PA14_19410 | PA14_19410 | hypothetical protein | Hypothetical, unclassified, unknown | Cytoplasmic [Class 3] |
| PA14_21240 | PA14_21240 | hypothetical protein | Hypothetical, unclassified, unknown | Unknown [Class 3] |
| PA14_21480 | PA14_21480 | hypothetical protein | Hypothetical, unclassified, unknown | Unknown [Class 3] |
| PA14_24240 | PA14_24240 | hypothetical protein | Hypothetical, unclassified, unknown | Cytoplasmic Membrane [Class 3] |
| PA14_25100 | PA14_25100 | hypothetical protein | Hypothetical, unclassified, unknown | Unknown [Class 3] |
| PA14_25470 | PA14_25470 | hypothetical protein | Hypothetical, unclassified, unknown | Cytoplasmic Membrane [Class 3] |
| PA14_27200 | PA14_27200 | hypothetical protein | Hypothetical, unclassified, unknown | Cytoplasmic [Class 3] |
| PA14_28040 | PA14_28040 | hypothetical protein | Hypothetical, unclassified, unknown | Unknown [Class 3] |
| PA14_28390 | PA14_28390 | hypothetical protein | Hypothetical, unclassified, unknown | Unknown [Class 3] |
| PA14_28800 | PA14_28800 | hypothetical protein | Hypothetical, unclassified, unknown | Unknown [Class 3] |
| PA14_28895 | PA14_28895 | hypothetical protein | Hypothetical, unclassified, unknown | Cytoplasmic [Class 3] |
| PA14_31070 | PA14_31070 | hypothetical protein | Hypothetical, unclassified, unknown | Cytoplasmic [Class 3] |
| PA14_31190 | PA14_31190 | hypothetical protein | Hypothetical, unclassified, unknown | Cytoplasmic [Class 3] |
| PA14_32790 | PA14_32790 | hypothetical protein | Hypothetical, unclassified, unknown | Outer Membrane [Class 3] |
| PA14_32840 | PA14_32840 | hypothetical protein | Hypothetical, unclassified, unknown | Unknown [Class 3] |
| PA14_33940 | PA14_33940 | hypothetical protein | Hypothetical, unclassified, unknown | Unknown [Class 3] |
| PA14_34010 | PA14_34010 | hypothetical protein | Hypothetical, unclassified, unknown | Cytoplasmic [Class 3] |
| PA14_34030 | PA14_34030 | hypothetical protein | Hypothetical, unclassified, unknown | Extracellular [Class 3] |
| PA14_36030 | PA14_36030 | paraquat-inducible protein A | Hypothetical, unclassified, unknown | Cytoplasmic Membrane [Class 3] |
| PA14_36060 | PA14_36060 | hypothetical protein | Hypothetical, unclassified, unknown | Cytoplasmic [Class 3] |
| PA14_37200 | PA14_37200 | hypothetical protein | Hypothetical, unclassified, unknown | Cytoplasmic [Class 3] |
| PA14_38180 | PA14_38180 | hypothetical protein | Hypothetical, unclassified, unknown | Unknown [Class 3] |
| PA14_39470 | PA14_39470 | hypothetical protein | Hypothetical, unclassified, unknown | Cytoplasmic [Class 3] |
| PA14_40260 | PA14_40260 | hypothetical protein | Hypothetical, unclassified, unknown | Unknown [Class 3] |
| PA14_40370 | PA14_40370 | hypothetical protein | Hypothetical, unclassified, unknown | Cytoplasmic [Class 3] |
| PA14_41030 | PA14_41030 | hypothetical protein | Hypothetical, unclassified, unknown | Cytoplasmic [Class 3] |
| PA14_43240 | PA14_43240 | hypothetical protein | Hypothetical, unclassified, unknown | Unknown [Class 3] |
| PA14_46540 | PA14_46540 | hypothetical protein | Hypothetical, unclassified, unknown | Cytoplasmic [Class 3] |
| PA14_46610 | PA14_46610 | hypothetical protein | Hypothetical, unclassified, unknown | Cytoplasmic Membrane [Class 3] |
| PA14_49740 | PA14_49740 | hypothetical protein | Hypothetical, unclassified, unknown | Unknown [Class 3] |
| PA14_49910 | PA14_49910 | hypothetical protein | Hypothetical, unclassified, unknown | Cytoplasmic Membrane [Class 3] |
| PA14_50300 | PA14_50300 | hypothetical protein | Hypothetical, unclassified, unknown | Cytoplasmic Membrane [Class 3] |
| PA14_50650 | PA14_50650 | hypothetical protein | Hypothetical, unclassified, unknown | Cytoplasmic [Class 3] |
| PA14_51580 | PA14_51580 | hypothetical protein | Hypothetical, unclassified, unknown | Unknown [Class 3] |
| PA14_53140 | PA14_53140 | hypothetical protein | Hypothetical, unclassified, unknown | Cytoplasmic Membrane [Class 3] |
| PA14_53820 | PA14_53820 | hypothetical protein | Hypothetical, unclassified, unknown | Cytoplasmic [Class 3] |
| PA14_53830 | PA14_53830 | hypothetical protein | Hypothetical, unclassified, unknown | Cytoplasmic Membrane [Class 3] |
| PA14_54180 | PA14_54180 | hypothetical protein | Hypothetical, unclassified, unknown | Outer Membrane [Class 3] |
| PA14_54490 | PA14_54490 | hypothetical protein | Hypothetical, unclassified, unknown | Cytoplasmic [Class 3] |
| PA14_54540 | PA14_54540 | hypothetical protein | Hypothetical, unclassified, unknown | Unknown [Class 3] |
| PA14_54740 | PA14_54740 | hypothetical protein | Hypothetical, unclassified, unknown | Unknown [Class 3] |
| PA14_54850 | PA14_54850 | hypothetical protein | Hypothetical, unclassified, unknown | Unknown [Class 3] |
| PA14_55320 | PA14_55320 | hypothetical protein | Hypothetical, unclassified, unknown | Outer Membrane [Class 3] |
| PA14_55400 | PA14_55400 | hypothetical protein | Hypothetical, unclassified, unknown | Extracellular [Class 3] |
| PA14_56800 | PA14_56800 | hypothetical protein | Hypothetical, unclassified, unknown | Cytoplasmic [Class 3] |
| PA14_57850 | PA14_57850 | hypothetical protein | Hypothetical, unclassified, unknown | Unknown [Class 3] |
| PA14_58740 | PA14_58740 | hypothetical protein | Hypothetical, unclassified, unknown | Unknown [Class 3] |
| PA14_59000 | PA14_59000 | hypothetical protein | Hypothetical, unclassified, unknown | Unknown [Class 3] |
| PA14_59020 | PA14_59020 | hypothetical protein | Hypothetical, unclassified, unknown | Unknown [Class 3] |
| PA14_59370 | PA14_59370 | hypothetical protein | Hypothetical, unclassified, unknown | Unknown [Class 3] |
| PA14_59630 | PA14_59630 | hypothetical protein | Hypothetical, unclassified, unknown | Unknown [Class 3] |
| PA14_60080 | PA14_60080 | hypothetical protein | Hypothetical, unclassified, unknown | Cytoplasmic [Class 3] |
| PA14_61200 | PA14_61200 | hypothetical protein | Hypothetical, unclassified, unknown | Outer Membrane [Class 3] |
| PA14_62020 | PA14_62020 | paraquat-inducible protein B-like protein | Hypothetical, unclassified, unknown | Outer Membrane [Class 3] |
| PA14_62410 | PA14_62410 | hypothetical protein | Hypothetical, unclassified, unknown | Cytoplasmic [Class 3] |
| PA14_63410 | PA14_63410 | hypothetical protein | Hypothetical, unclassified, unknown | Cytoplasmic [Class 3] |
| PA14_64170 | PA14_64170 | hypothetical protein | Hypothetical, unclassified, unknown | Unknown [Class 3] |
| PA14_65060 | PA14_65060 | hypothetical protein | Hypothetical, unclassified, unknown | Cytoplasmic [Class 3] |
| PA14_65470 | PA14_65470 | hypothetical protein | Hypothetical, unclassified, unknown | Cytoplasmic Membrane [Class 3] |
| PA14_65520 | PA14_65520 | hypothetical protein | Hypothetical, unclassified, unknown | Unknown [Class 3] |
| PA14_66540 | PA14_66540 | hypothetical protein | Hypothetical, unclassified, unknown | Unknown [Class 3] |
| PA14_70170 | PA14_70170 | hypothetical protein | Hypothetical, unclassified, unknown | Unknown [Class 3] |
| PA14_73030 | PA14_73030 | hypothetical protein | Hypothetical, unclassified, unknown | Cytoplasmic Membrane [Class 3] |
| PA14_51380 | pqsE | quinolone signal response protein | Hypothetical, unclassified, unknown | Cytoplasmic [Class 3] |
| PA14_41680 | ydiA | hypothetical protein | Hypothetical, unclassified, unknown | Cytoplasmic [Class 3] |
| PA14_51810 | yebC | hypothetical protein | Hypothetical, unclassified, unknown | Cytoplasmic [Class 3] |
| PA14_49160 | yegE | putative sensor protein | Hypothetical, unclassified, unknown | Cytoplasmic Membrane [Class 3] |
| PA14_58660 | ampE | hypothetical protein | Membrane proteins | Cytoplasmic Membrane [Class 3] |
| PA14_26360 | btuC | putative permease of ABC transporter | Membrane proteins | Cytoplasmic Membrane [Class 3] |
| PA14_57030 | fxsA | FxsA | Membrane proteins | Cytoplasmic Membrane [Class 3] |
| PA14_56830 | icmP | metalloproteinase outer membrane | Membrane proteins | Outer Membrane [Class 3] |
| PA14_33410 | opdJ | porin | Membrane proteins | Outer Membrane [Class 3] |
| PA14_00510 | PA14_00510 | putative hemagglutinin | Membrane proteins | Outer Membrane [Class 3] |
| PA14_26420 | PA14_26420 | putative TonB-dependent receptor | Membrane proteins | Outer Membrane [Class 3] |
| PA14_28980 | PA14_28980 | Fe2+-dicitrate sensor | Membrane proteins | Periplasmic [Class 3] |
| PA14_36170 | PA14_36170 | hypothetical protein | Membrane proteins | Cytoplasmic Membrane [Class 3] |
| PA14_37900 | PA14_37900 | putative TonB-dependent receptor | Membrane proteins | Outer Membrane [Class 3] |
| PA14_39810 | PA14_39810 | putative transmembrane sensor | Membrane proteins | Periplasmic [Class 3] |
| PA14_41750 | PA14_41750 | hypothetical protein | Membrane proteins | Outer Membrane [Class 3] |
| PA14_49570 | PA14_49570 | amino acid permease | Membrane proteins | Cytoplasmic Membrane [Class 3] |
| PA14_55050 | PA14_55050 | TonB-dependent receptor | Membrane proteins | Outer Membrane [Class 3] |
| PA14_55820 | PA14_55820 | hypothetical protein | Membrane proteins | Cytoplasmic Membrane [Class 3] |
| PA14_60730 | PA14_60730 | putative outer membrane protein | Membrane proteins | Outer Membrane [Class 3] |
| PA14_62100 | PA14_62100 | putative sulfite oxidase subunit YedZ | Membrane proteins | Cytoplasmic Membrane [Class 3] |
| PA14_65030 | PA14_65030 | hypothetical protein | Membrane proteins | Outer Membrane [Class 3] |
| PA14_68280 | PA14_68280 | dicarboxylate transporter | Membrane proteins | Cytoplasmic Membrane [Class 3] |
| PA14_02810 | pcaT | dicarboxylic acid transporter PcaT | Membrane proteins | Cytoplasmic Membrane [Class 3] |
| PA14_17140 | yaeL | putative membrane-associated zinc metalloprotease | Membrane proteins | Cytoplasmic Membrane [Class 3] |
| PA14_40960 | fimL | pilin biosynthetic protein | Motility & Attachment | Cytoplasmic [Class 3] |
| PA14_23830 | fimV | pilus assembly protein | Motility & Attachment | Unknown [Class 3] |
| PA14_20740 | flgA | flagellar basal body P-ring biosynthesis protein FlgA | Motility & Attachment | Cytoplasmic [Class 3] |
| PA14_50160 | fliE | flagellar hook-basal body protein FliE | Motility & Attachment | Flagellar [Class 3] ; Periplasmic [Class 3] |
| PA14_50100 | fliI | flagellum-specific ATP synthase | Motility & Attachment | Cytoplasmic [Class 3] |
| PA14_69760 | PA14_69760 | putative fimbrial protein | Motility & Attachment | Unknown [Class 3] |
| PA14_58730 | pilA | type IV pilin structural subunit | Motility & Attachment | Fimbrial [Class 3] ; Extracellular [Class 3] |
| PA14_58750 | pilB | type 4 fimbrial biogenesis protein PilB | Motility & Attachment | Cytoplasmic [Class 3] |
| PA14_14850 | pilF | type 4 fimbrial biogenesis protein PilF | Motility & Attachment | Outer Membrane [Class 3] |
| PA14_66660 | pilM | type 4 fimbrial biogenesis protein PilM | Motility & Attachment | Cytoplasmic Membrane [Class 3] |
| PA14_59250 | pilN2 | Type IV B pilus protein | Motility & Attachment | Outer Membrane [Class 3] |
| PA14_66630 | pilP | type 4 fimbrial biogenesis protein PilP | Motility & Attachment | Cytoplasmic Membrane [Class 3] |
| PA14_66620 | pilQ | type 4 fimbrial biogenesis outer membrane protein PilQ precursor | Motility & Attachment | Outer Membrane [Class 3] |
| PA14_60290 | pilW | type 4 fimbrial biogenesis protein PilW | Motility & Attachment | Unknown [Class 3] |
| PA14_60310 | pilY1 | type 4 fimbrial biogenesis protein PilY1 | Motility & Attachment | Outer Membrane [Class 3] |
| PA14_16500 | wspR | two-component response regulator | Motility & Attachment | Cytoplasmic [Class 3] |
| PA14_20500 | PA14_20500 | tRNA-Arg | Non-coding RNA gene | Unknown [Class 3] |
| PA14_60180 | PA14_60180 | tRNA-Asn | Non-coding RNA gene | Unknown [Class 3] |
| PA14_62910 | carB | carbamoyl phosphate synthase large subunit | Nucleotide biosynthesis and metabolism | Cytoplasmic [Class 3] |
| PA14_49840 | dgt | deoxyguanosinetriphosphate triphosphohydrolase | Nucleotide biosynthesis and metabolism | Cytoplasmic [Class 3] |
| PA14_01660 | guaD | guanine deaminase | Nucleotide biosynthesis and metabolism | Cytoplasmic [Class 3] |
| PA14_12490 | PA14_12490 | AMP nucleosidase | Nucleotide biosynthesis and metabolism | Cytoplasmic [Class 3] |
| PA14_69230 | ppk | polyphosphate kinase | Nucleotide biosynthesis and metabolism | Cytoplasmic Membrane [Class 3] |
| PA14_15740 | purL | phosphoribosylformylglycinamidine synthase | Nucleotide biosynthesis and metabolism | Cytoplasmic [Class 3] |
| PA14_05740 | pydA | dihydropyrimidine dehydrogenase | Nucleotide biosynthesis and metabolism | Cytoplasmic [Class 3] |
| PA14_73070 | pyrQ | dihydroorotase | Nucleotide biosynthesis and metabolism | Cytoplasmic [Class 3] |
| PA14_25390 | sth | soluble pyridine nucleotide transhydrogenase | Nucleotide biosynthesis and metabolism | Cytoplasmic [Class 3] |
| PA14_61470 | upp | uracil phosphoribosyltransferase | Nucleotide biosynthesis and metabolism | Cytoplasmic [Class 3] |
| PA14_42400 | exsB | exoenzyme S synthesis protein B | Protein secretion/export apparatus | Unknown [Class 3] |
| PA14_20030 | hasD | transport protein HasD | Protein secretion/export apparatus | Cytoplasmic Membrane [Class 3] |
| PA14_29510 | hplT | putative type II secretion system protein | Protein secretion/export apparatus | Cytoplasmic Membrane [Class 3] |
| PA14_55440 | hxcR | putative type II secretion system protein | Protein secretion/export apparatus | Cytoplasmic [Class 3] |
| PA14_55430 | hxcS | putative type II secretion system protein | Protein secretion/export apparatus | Cytoplasmic Membrane [Class 3] |
| PA14_31920 | opmB | putative outer membrane protein | Protein secretion/export apparatus | Outer Membrane [Class 3] |
| PA14_45910 | PA14_45910 | putative RND efflux membrane fusion protein precursor | Protein secretion/export apparatus | Cytoplasmic Membrane [Class 3] |
| PA14_55930 | PA14_55930 | putative pilus assembly protein | Protein secretion/export apparatus | Unknown [Class 3] |
| PA14_42500 | pcrD | type III secretory apparatus protein PcrD | Protein secretion/export apparatus | Cytoplasmic Membrane [Class 3] |
| PA14_23970 | xcpQ | general secretion pathway protein D | Protein secretion/export apparatus | Outer Membrane [Class 3] |
| PA14_40320 | xqhA | secretion protein XqhA | Protein secretion/export apparatus | Outer Membrane [Class 3] |
| PA14_53420 | btuE | glutathione peroxidase | Putative enzymes | Periplasmic [Class 3] |
| PA14_50840 | dinG | ATP-dependent DNA helicase DinG | Putative enzymes | Cytoplasmic [Class 3] |
| PA14_49800 | gsp69 | oxidoreductase | Putative enzymes | Cytoplasmic [Class 3] |
| PA14_00750 | PA14_00750 | hypothetical protein | Putative enzymes | Unknown [Class 3] |
| PA14_01490 | PA14_01490 | putative hemolysin | Putative enzymes | Extracellular [Class 3] |
| PA14_10840 | PA14_10840 | putative dehydrogenase | Putative enzymes | Cytoplasmic [Class 3] |
| PA14_11580 | PA14_11580 | hypothetical protein | Putative enzymes | Cytoplasmic [Class 3] |
| PA14_29130 | PA14_29130 | ATPase | Putative enzymes | Cytoplasmic [Class 3] |
| PA14_29230 | PA14_29230 | hypothetical protein | Putative enzymes | Unknown [Class 3] |
| PA14_34580 | PA14_34580 | hypothetical protein | Putative enzymes | Cytoplasmic [Class 3] |
| PA14_34840 | PA14_34840 | putative non-ribosomal peptide synthetase | Putative enzymes | Cytoplasmic [Class 3] |
| PA14_35790 | PA14_35790 | Putative homospermidine synthase | Putative enzymes | Unknown [Class 3] |
| PA14_42050 | PA14_42050 | putative oxidoreductase | Putative enzymes | Cytoplasmic [Class 3] |
| PA14_44090 | PA14_44090 | putative Fe-S-cluster oxidoreductase | Putative enzymes | Unknown [Class 3] |
| PA14_45170 | PA14_45170 | putative oxidoreductase | Putative enzymes | Cytoplasmic [Class 3] |
| PA14_49300 | PA14_49300 | lipoxygenase | Putative enzymes | Periplasmic [Class 3] |
| PA14_51100 | PA14_51100 | acyl-CoA dehydrogenase | Putative enzymes | Cytoplasmic [Class 3] |
| PA14_52880 | PA14_52880 | hypothetical protein | Putative enzymes | Cytoplasmic [Class 3] |
| PA14_53300 | PA14_53300 | alkyl hydroperoxide reductase | Putative enzymes | Cytoplasmic [Class 3] |
| PA14_54120 | PA14_54120 | acyl carrier protein phosphodiesterase | Putative enzymes | Cytoplasmic Membrane [Class 3] |
| PA14_54630 | PA14_54630 | acyl-CoA dehydrogenase | Putative enzymes | Cytoplasmic [Class 3] |
| PA14_54920 | PA14_54920 | putative non-ribosomal peptide synthetase | Putative enzymes | Unknown [Class 3] |
| PA14_56040 | PA14_56040 | hypothetical protein | Putative enzymes | Cytoplasmic [Class 3] |
| PA14_56840 | PA14_56840 | hypothetical protein | Putative enzymes | Unknown [Class 3] |
| PA14_58990 | PA14_58990 | Putative DNA helicase | Putative enzymes | Cytoplasmic [Class 3] |
| PA14_69870 | pchP | phosphorylcholine phosphatase | Putative enzymes | Unknown [Class 3] |
| PA14_62330 | phuS | putative hemin degrading factor | Putative enzymes | Cytoplasmic [Class 3] |
| PA14_58560 | piuB | oxidoreductase | Putative enzymes | Cytoplasmic Membrane [Class 3] |
| PA14_23650 | ygfF | short chain dehydrogenase | Putative enzymes | Cytoplasmic [Class 3] |
| PA14_67810 | ctpA | putative carboxyl-terminal protease | Related to phage, transposon, or plasmid | Cytoplasmic Membrane [Class 3] |
| PA14_35740 | tpnA | putative transposase | Related to phage, transposon, or plasmid | Unknown [Class 3] |
| PA14_30850 | trbI | TrbI-like protein | Related to phage, transposon, or plasmid | Unknown [Class 3] |
| PA14_15540 | trbL | putative mating pair formation protein TrbL | Related to phage, transposon, or plasmid | Cytoplasmic Membrane [Class 3] |
| PA14_18520 | algK | alginate biosynthetic protein AlgK precursor | Secreted Factors (toxins, enzymes, alginate) | Periplasmic [Class 3] |
| PA14_35390 | pvcD | pyoverdine biosynthesis protein PvcD | Secreted Factors (toxins, enzymes, alginate) | Periplasmic [Class 3] |
| PA14_19110 | rhlB | rhamnosyltransferase chain B | Secreted Factors (toxins, enzymes, alginate) | Cytoplasmic [Class 3] |
| PA14_52600 | alaS | alanyl-tRNA synthetase | Transcription, RNA processing and degradation | Cytoplasmic [Class 3] |
| PA14_51820 | aspS | aspartyl-tRNA synthetase | Transcription, RNA processing and degradation | Cytoplasmic [Class 3] |
| PA14_07620 | cca | multifunctional tRNA nucleotidyl transferase/2'3'-cyclic phosphodiesterase/2'nucleotidase/phosphatase | Transcription, RNA processing and degradation | Cytoplasmic [Class 3] |
| PA14_27370 | deaD | ATP-dependent RNA helicase | Transcription, RNA processing and degradation | Cytoplasmic [Class 3] |
| PA14_12540 | PA14_12540 | putative pseudouridine synthase | Transcription, RNA processing and degradation | Cytoplasmic [Class 3] |
| PA14_51960 | rbn | ribonuclease | Transcription, RNA processing and degradation | Cytoplasmic Membrane [Class 3] |
| PA14_68710 | tex | hypothetical protein | Transcription, RNA processing and degradation | Cytoplasmic [Class 3] |
| PA14_03840 | aguR | transcriptional regulator AguR | Transcriptional regulators | Cytoplasmic [Class 3] |
| PA14_38380 | amrR | putative transcriptional regulator | Transcriptional regulators | Cytoplasmic [Class 3] |
| PA14_37940 | cynR | DNA-binding transcriptional regulator CynR | Transcriptional regulators | Cytoplasmic [Class 3] |
| PA14_06870 | dnr | transcriptional regulator Dnr | Transcriptional regulators | Cytoplasmic [Class 3] |
| PA14_50220 | fleQ | transcriptional regulator FleQ | Transcriptional regulators | Cytoplasmic [Class 3] |
| PA14_45630 | fliA | flagellar biosynthesis sigma factor | Transcriptional regulators | Cytoplasmic [Class 3] |
| PA14_26860 | hpkR | LysR family transcriptional regulator | Transcriptional regulators | Cytoplasmic [Class 3] |
| PA14_45960 | lasR | transcriptional regulator LasR | Transcriptional regulators | Cytoplasmic [Class 3] |
| PA14_05520 | mexR | multidrug resistance operon repressor MexR | Transcriptional regulators | Cytoplasmic [Class 3] |
| PA14_03480 | PA14_03480 | GntR family transcriptional regulator | Transcriptional regulators | Cytoplasmic [Class 3] |
| PA14_04160 | PA14_04160 | putatitve transcriptional regulator | Transcriptional regulators | Cytoplasmic Membrane [Class 3] |
| PA14_06240 | PA14_06240 | LysR family transcriptional regulator | Transcriptional regulators | Cytoplasmic [Class 3] |
| PA14_06400 | PA14_06400 | LysR family transcriptional regulator | Transcriptional regulators | Cytoplasmic [Class 3] |
| PA14_06950 | PA14_06950 | LuxR family transcriptional regulator | Transcriptional regulators | Cytoplasmic [Class 3] |
| PA14_11830 | PA14_11830 | transcriptional regulator | Transcriptional regulators | Cytoplasmic [Class 3] |
| PA14_13000 | PA14_13000 | transcriptional regulator | Transcriptional regulators | Cytoplasmic [Class 3] |
| PA14_13060 | PA14_13060 | putative transcriptional regulator | Transcriptional regulators | Cytoplasmic [Class 3] |
| PA14_19850 | PA14_19850 | putative transcriptional regulator | Transcriptional regulators | Unknown [Class 3] |
| PA14_28420 | PA14_28420 | LysR family transcriptional regulator | Transcriptional regulators | Cytoplasmic [Class 3] |
| PA14_29180 | PA14_29180 | AraC family transcriptional regulator | Transcriptional regulators | Cytoplasmic [Class 3] |
| PA14_40380 | PA14_40380 | TetR family transcriptional regulator | Transcriptional regulators | Cytoplasmic [Class 3] |
| PA14_40910 | PA14_40910 | LysR family transcriptional regulatory protein | Transcriptional regulators | Unknown [Class 3] |
| PA14_43430 | PA14_43430 | IclR family transcriptional regulator | Transcriptional regulators | Cytoplasmic [Class 3] |
| PA14_44780 | PA14_44780 | putative transcriptional regulator | Transcriptional regulators | Cytoplasmic [Class 3] |
| PA14_46330 | PA14_46330 | putative transcriptional regulator | Transcriptional regulators | Cytoplasmic [Class 3] |
| PA14_47580 | PA14_47580 | MarR family transcriptional regulator | Transcriptional regulators | Cytoplasmic [Class 3] |
| PA14_52930 | PA14_52930 | transcriptional regulator | Transcriptional regulators | Cytoplasmic [Class 3] |
| PA14_55550 | PA14_55550 | ECF subfamily RNA polymerase sigma-70 factor | Transcriptional regulators | Cytoplasmic [Class 3] |
| PA14_63280 | PA14_63280 | putative transcriptional regulator | Transcriptional regulators | Cytoplasmic [Class 3] |
| PA14_64500 | PA14_64500 | putative transcriptional regulator | Transcriptional regulators | Cytoplasmic [Class 3] |
| PA14_70290 | PA14_70290 | putative transcriptional regulator | Transcriptional regulators | Cytoplasmic [Class 3] |
| PA14_70530 | PA14_70530 | AraC family transcriptional regulator | Transcriptional regulators | Cytoplasmic [Class 3] |
| PA14_70560 | PA14_70560 | LysR family transcriptional regulator | Transcriptional regulators | Cytoplasmic [Class 3] |
| PA14_71640 | PA14_71640 | LysR family transcriptional regulator | Transcriptional regulators | Cytoplasmic [Class 3] |
| PA14_19120 | rhlR | transcriptional regulator RhlR | Transcriptional regulators | Cytoplasmic [Class 3] |
| PA14_38250 | yjiR | putative transcriptional regulator | Transcriptional regulators | Cytoplasmic [Class 3] |
| PA14_08520 | anmK | anhydro-N-acetylmuramic acid kinase | Translation, post-translational modification, degradation | Cytoplasmic [Class 3] |
| PA14_06000 | clpA | putative ClpA/B protease ATP binding subunit | Translation, post-translational modification, degradation | Cytoplasmic [Class 3] |
| PA14_01100 | clpB | putative ClpA/B-type chaperone | Translation, post-translational modification, degradation | Cytoplasmic [Class 3] |
| PA14_32610 | dsbG | disulfide isomerase/thiol-disulfide oxidase | Translation, post-translational modification, degradation | Periplasmic [Class 3] |
| PA14_28450 | eco | ecotin | Translation, post-translational modification, degradation | Periplasmic [Class 3] |
| PA14_00100 | glyQ | glycyl-tRNA synthetase subunit alpha | Translation, post-translational modification, degradation | Cytoplasmic [Class 3] |
| PA14_60370 | ileS | isoleucyl-tRNA synthetase | Translation, post-translational modification, degradation | Cytoplasmic [Class 3] |
| PA14_00875 | ppkA | serine/threonine protein kinase PpkA | Translation, post-translational modification, degradation | Cytoplasmic Membrane [Class 3] |
| PA14_13410 | prfC | peptide chain release factor 3 | Translation, post-translational modification, degradation | Cytoplasmic [Class 3] |
| PA14_52190 | rumA | 23S rRNA 5-methyluridine methyltransferase | Translation, post-translational modification, degradation | Cytoplasmic [Class 3] |
| PA14_63060 | smpB | SsrA-binding protein | Translation, post-translational modification, degradation | Unknown [Class 3] |
| PA14_42890 | stp1 | serine/threonine phosphoprotein phosphatase Stp1 | Translation, post-translational modification, degradation | Cytoplasmic [Class 3] |
| PA14_17440 | truD | tRNA pseudouridine synthase D | Translation, post-translational modification, degradation | Cytoplasmic [Class 3] |
| PA14_61820 | ychF | GTP-dependent nucleic acid-binding protein EngD | Translation, post-translational modification, degradation | Cytoplasmic [Class 3] |
| PA14_64180 | yhdG | hypothetical protein | Translation, post-translational modification, degradation | Cytoplasmic [Class 3] |
| PA14_52720 | argD | putative class III pyridoxal phosphate-dependent aminotransferase | Transport of small molecules | Cytoplasmic Membrane [Class 3] |
| PA14_25270 | aroP1 | aromatic amino acid transport protein AroP1 | Transport of small molecules | Cytoplasmic Membrane [Class 3] |
| PA14_53050 | aroP2 | aromatic amino acid transport protein AroP2 | Transport of small molecules | Cytoplasmic Membrane [Class 3] |
| PA14_02340 | atsB | putative permease of ABC transporter | Transport of small molecules | Cytoplasmic Membrane [Class 3] |
| PA14_43160 | benE | putative transporter | Transport of small molecules | Cytoplasmic Membrane [Class 3] |
| PA14_09160 | bfrA | bacterioferritin | Transport of small molecules | Cytoplasmic [Class 3] |
| PA14_16660 | cadA | putative metal-transporting P-type ATPase | Transport of small molecules | Cytoplasmic Membrane [Class 3] |
| PA14_39650 | cirA | putative TonB-dependent receptor | Transport of small molecules | Outer Membrane [Class 3] |
| PA14_69570 | corA | magnesium/cobalt transport protein | Transport of small molecules | Cytoplasmic Membrane [Class 3] |
| PA14_01460 | dctA | C4-dicarboxylate transporter DctA | Transport of small molecules | Cytoplasmic Membrane [Class 3] |
| PA14_68140 | emrB | drug efflux transporter | Transport of small molecules | Cytoplasmic Membrane [Class 3] |
| PA14_21730 | fecA | Fe(III) dicitrate transport protein FecA | Transport of small molecules | Outer Membrane [Class 3] |
| PA14_09970 | fpvB | type I ferripyoverdine receptor, FpvB | Transport of small molecules | Outer Membrane [Class 3] |
| PA14_20010 | hasR | heme uptake outer membrane receptor HasR precursor | Transport of small molecules | Outer Membrane [Class 3] |
| PA14_62010 | hitB | putative iron ABC transporter, permease protein | Transport of small molecules | Cytoplasmic Membrane [Class 3] |
| PA14_61850 | iutA | putative TonB-dependent receptor | Transport of small molecules | Outer Membrane [Class 3] |
| PA14_48700 | kefB | glutathione-regulated potassium-efflux system protein KefB | Transport of small molecules | Cytoplasmic Membrane [Class 3] |
| PA14_35330 | kguT | putative 2-ketogluconate transporter | Transport of small molecules | Cytoplasmic Membrane [Class 3] |
| PA14_61250 | lysP | APC family lysine-specific permease | Transport of small molecules | Cytoplasmic Membrane [Class 3] |
| PA14_51880 | oprD | Basic amino acid, basic peptide and imipenem outer membrane porin OprD precursor | Transport of small molecules | Outer Membrane [Class 3] |
| PA14_00340 | PA14_00340 | putative sulfate transporter | Transport of small molecules | Cytoplasmic Membrane [Class 3] |
| PA14_01960 | PA14_01960 | putative RND efflux membrane fusion protein precursor | Transport of small molecules | Cytoplasmic Membrane [Class 3] |
| PA14_07860 | PA14_07860 | putative ATP-binding component of ABC transporter | Transport of small molecules | Cytoplasmic Membrane [Class 3] |
| PA14_11600 | PA14_11600 | ABC transporter | Transport of small molecules | Cytoplasmic Membrane [Class 3] |
| PA14_13580 | PA14_13580 | ABC transporter ATP-binding protein | Transport of small molecules | Cytoplasmic Membrane [Class 3] |
| PA14_15270 | PA14_15270 | hypothetical protein | Transport of small molecules | Cytoplasmic Membrane [Class 3] |
| PA14_15700 | PA14_15700 | putative amino acid permease | Transport of small molecules | Cytoplasmic Membrane [Class 3] |
| PA14_15780 | PA14_15780 | N-Acetyl-D-Glucosamine phosphotransferase system transporter | Transport of small molecules | Cytoplasmic Membrane [Class 3] |
| PA14_19310 | PA14_19310 | hypothetical protein | Transport of small molecules | Cytoplasmic Membrane [Class 3] |
| PA14_21300 | PA14_21300 | putative MFS transporte | Transport of small molecules | Cytoplasmic Membrane [Class 3] |
| PA14_26050 | PA14_26050 | putative transporter | Transport of small molecules | Cytoplasmic Membrane [Class 3] |
| PA14_29770 | PA14_29770 | putative transporter | Transport of small molecules | Cytoplasmic Membrane [Class 3] |
| PA14_30790 | PA14_30790 | hypothetical protein | Transport of small molecules | Cytoplasmic Membrane [Class 3] |
| PA14_31900 | PA14_31900 | putative efflux transporter | Transport of small molecules | Cytoplasmic Membrane [Class 3] |
| PA14_34330 | PA14_34330 | hypothetical protein | Transport of small molecules | Unknown [Class 3] |
| PA14_35920 | PA14_35920 | acetate permease | Transport of small molecules | Cytoplasmic Membrane [Class 3] |
| PA14_37730 | PA14_37730 | putative TonB dependent receptor | Transport of small molecules | Outer Membrane [Class 3] |
| PA14_38080 | PA14_38080 | hypothetical protein | Transport of small molecules | Outer Membrane [Class 3] |
| PA14_38220 | PA14_38220 | hypothetical protein | Transport of small molecules | Cytoplasmic [Class 3] |
| PA14_41930 | PA14_41930 | hypothetical protein | Transport of small molecules | Unknown [Class 3] |
| PA14_46110 | PA14_46110 | putative sodium:solute symport protein | Transport of small molecules | Cytoplasmic Membrane [Class 3] |
| PA14_54980 | PA14_54980 | poossible ABC-type transporter protein | Transport of small molecules | Cytoplasmic Membrane [Class 3] |
| PA14_64710 | PA14_64710 | putative extracellular heme-binding protein | Transport of small molecules | Outer Membrane [Class 3] |
| PA14_69340 | PA14_69340 | putative ATP-binding component of ABC transporter | Transport of small molecules | Cytoplasmic [Class 3] |
| PA14_70200 | PA14_70200 | putative binding protein component of ABC dipeptide transporter | Transport of small molecules | Periplasmic [Class 3] |
| PA14_71000 | PA14_71000 | putative lycine betaine/L-proline ABC transporter, ATP-binding subunit | Transport of small molecules | Cytoplasmic Membrane [Class 3] |
| PA14_73160 | PA14_73160 | putative permease | Transport of small molecules | Cytoplasmic Membrane [Class 3] |
| PA14_09270 | pchE | dihydroaeruginoic acid synthetase | Transport of small molecules | Unknown [Class 3] |
| PA14_20300 | phnC | ATP-binding component of ABC phosphonate transporter | Transport of small molecules | Cytoplasmic Membrane [Class 3] |
| PA14_20330 | phnE | phosphonate ABC tranporter permease protein | Transport of small molecules | Cytoplasmic Membrane [Class 3] |
| PA14_70800 | phoU | phosphate uptake regulatory protein PhoU | Transport of small molecules | Cytoplasmic [Class 3] |
| PA14_30570 | potF | putative periplasmic spermidine/putrescine-binding protein | Transport of small molecules | Periplasmic [Class 3] |
| PA14_67300 | proX | putative binding protein component of ABC transporter | Transport of small molecules | Periplasmic [Class 3] |
| PA14_39320 | rbsC | membrane protein component of ABC ribose transporter | Transport of small molecules | Cytoplasmic Membrane [Class 3] |
| PA14_31380 | sfpA | putative sulfate transporter | Transport of small molecules | Cytoplasmic Membrane [Class 3] |
| PA14_19500 | ssuA | hypothetical protein | Transport of small molecules | Unknown [Class 3] |
| PA14_39820 | ufrA | putative tonB-dependent receptor protein | Transport of small molecules | Outer Membrane [Class 3] |
| PA14_61480 | uraA | Uracil permease | Transport of small molecules | Cytoplasmic Membrane [Class 3] |
| PA14_64790 | vanK | putative MFS transporter | Transport of small molecules | Cytoplasmic Membrane [Class 3] |
| PA14_63230 | yedA | hypothetical protein | Transport of small molecules | Cytoplasmic Membrane [Class 3] |
| PA14_38110 | ygjU | serine/threonine transporter SstT | Transport of small molecules | Cytoplasmic Membrane [Class 3] |
| PA14_17010 | yjcE | putative putative Na(+)/H(+) exchanger protein | Transport of small molecules | Cytoplasmic Membrane [Class 3] |
| PA14_66510 | ynfM | putative MFS transporter | Transport of small molecules | Cytoplasmic Membrane [Class 3] |
| PA14_72550 | znuA | putative adhesin | Transport of small molecules | Periplasmic [Class 3] |
| PA14_69470 | algR | alginate biosynthesis regulatory protein AlgR | Two-component regulatory systems | Cytoplasmic [Class 3] |
| PA14_61020 | ankB | hypothetical protein | Two-component regulatory systems | Unknown [Class 3] |
| PA14_45590 | cheA | putative two-component sensor | Two-component regulatory systems | Cytoplasmic [Class 3] |
| PA14_50180 | fleR | two-component response regulator | Two-component regulatory systems | Cytoplasmic [Class 3] |
| PA14_43350 | kdpD | two-component sensor KdpD | Two-component regulatory systems | Cytoplasmic Membrane [Class 3] |
| PA14_43340 | kdpE | two-component response regulator KdpE | Two-component regulatory systems | Cytoplasmic [Class 3] |
| PA14_03720 | PA14_03720 | sensory box GGDEF domain-containing protein | Two-component regulatory systems | Cytoplasmic Membrane [Class 3] |
| PA14_10770 | PA14_10770 | putative sensor/response regulator hybrid | Two-component regulatory systems | Cytoplasmic Membrane [Class 3] |
| PA14_11630 | PA14_11630 | putative two-component sensor | Two-component regulatory systems | Cytoplasmic Membrane [Class 3] |
| PA14_12810 | PA14_12810 | two-component response regulator | Two-component regulatory systems | Cytoplasmic Membrane [Class 3] |
| PA14_16350 | PA14_16350 | putative two-component response regulator | Two-component regulatory systems | Cytoplasmic [Class 3] |
| PA14_20820 | PA14_20820 | putative two-component response regulator | Two-component regulatory systems | Cytoplasmic Membrane [Class 3] |
| PA14_22960 | PA14_22960 | putative two-component sensor | Two-component regulatory systems | Cytoplasmic Membrane [Class 3] |
| PA14_26810 | PA14_26810 | putative two-component sensor | Two-component regulatory systems | Cytoplasmic Membrane [Class 3] |
| PA14_27800 | PA14_27800 | putative two-component sensor | Two-component regulatory systems | Cytoplasmic Membrane [Class 3] |
| PA14_29740 | PA14_29740 | putative two-component sensor | Two-component regulatory systems | Cytoplasmic Membrane [Class 3] |
| PA14_30840 | PA14_30840 | putative signal transduction histidine kinase | Two-component regulatory systems | Cytoplasmic Membrane [Class 3] |
| PA14_45870 | PA14_45870 | putative two-component sensor | Two-component regulatory systems | Cytoplasmic Membrane [Class 3] |
| PA14_48160 | PA14_48160 | putative sensor/response regulator hybrid | Two-component regulatory systems | Cytoplasmic Membrane [Class 3] |
| PA14_54500 | PA14_54500 | two-component sensor | Two-component regulatory systems | Cytoplasmic Membrane [Class 3] |
| PA14_54510 | PA14_54510 | two-component response regulator | Two-component regulatory systems | Cytoplasmic [Class 3] |
| PA14_65860 | PA14_65860 | putative two-component sensor | Two-component regulatory systems | Cytoplasmic Membrane [Class 3] |
| PA14_69900 | PA14_69900 | hypothetical protein | Two-component regulatory systems | Cytoplasmic Membrane [Class 3] |
| PA14_60260 | pilR | two-component response regulator PilR | Two-component regulatory systems | Cytoplasmic [Class 3] |
| PA14_07680 | prkA | hypothetical protein | Two-component regulatory systems | Cytoplasmic [Class 3] |
| PA14_11120 | rcsB | putative response regulator | Two-component regulatory systems | Cytoplasmic [Class 3] |
| PA14_59780 | rcsC | putative kinase sensor protein | Two-component regulatory systems | Cytoplasmic Membrane [Class 3] |
