# Supplementary material for: A Comprehensive Analysis of In Vitro and In Vivo Genetic Fitness of Pseudomonas aeruginosa Using High-Throughput Sequencing of Transposon Libraries
Source: PLoS Pathog. 2013 Sep 5;9(9):e1003582. doi: 10.1371/journal.ppat.1003582 (PMC3764216; doi:10.1371/journal.ppat.1003582)
Supplement: Text S1 — Requirements for respiration and energy generation, ion pumps and redox reactions and potential targets for immunotherapy by P. aeruginosa during infection. (DOC) [file ppat.1003582.s022.doc]

## Requirements for respiration and energy generation, ion pumps and redox reactions and potential targets for immunotherapy by *P. aeruginosa* during infection

The ability of bacterial pathogens to proliferate in an infected tissue is an important component of the infection process, reliant on respiration, energy generation, nutrient utilization and biosynthesis of essential factors. Additionally, metabolic enzymes could perform a secondary function by detoxifying reactive oxygen or nitrogen radicals that are part of innate host defense mechanisms. Also, effects of Tn insertions on respiration and energy generation can likely have secondary effects on levels or functions of virulence factors. Not unexpectedly, a significant number of Tn insertions showing decreased fitness in the ceca and spleens were in genes within operons involved in respiration (Figure 6), likely due to the anaerobic environment of the GI tract, and genes involved in growth/or utilization of nutrients (Figure S7). Tn insertions in several oxygen independent energy generating pathways showed decreased fitness (Figure 6) such as the homologs of the 13 *nuo* genes whose products form a proton-pumping NADH: ubiquinone oxidoreductase also called the respiratory complex I, which generates energy by coupling the electron transfer from NADH to ubiquinone with the translocation of protons across the membrane. Interestingly, a few Tn­­ insertions in genes in these functional classes were enriched during GI colonization and/or dissemination (Figure 6, Table S4) indicative of genes whose inactivation may have led to an enhancement of the activity of metabolic pathways needed for virulence and therefore may be similar to the enhanced fitness of strains with Tn insertions into the *algR, algZ* and *rpoN* regulatory genes.

## Aerobic respiration and terminal oxidases

*P. aeruginosa* has respiration pathways terminated by oxygen and nitrogen acceptors, making it possible for this organism to grow by aerobic respiration when oxygen is available and by dissimilatory nitrate respiration (denitrification) under anaerobic conditions [1]. *P. aeruginosa* can also generate energy anaerobically via the breakdown of arginine [2]. *P. aeruginosa* prefers oxygen respiration, assembling the best suited electron transport chain for a particular environment by controlling the expression of terminal oxidases [3]. A branched aerobic respiratory chain terminated by five terminal oxidases supports the respiratory mode of energy generation [4] . We observed that Tn insertions into genes within the operons for the two quinol oxidases *cyoA-E* or *cioAB*, specifying the bo_3_ and the cyanide insensitive CIO oxidases, respectively, led to decreased *in vivo* fitness (Figure 6). Tn insertions into the genes for the three cytochrome C oxidases aa3 oxidase, cbb3 oxidase 1, and cbb3 oxidase 2 encoded in the *cyocoxBAPA14_0107coIII*, *ccoN1ccoO1ccQ1ccoP1* and *ccoN2ccoO2ccoP2* operons, respectively, were markedly underrepresented in the pool recovered from the ceca (Figure 6). The cbb3-1, cbb3-2 and CIO enzymes are high affinity terminal oxidases and have been shown to be highly expressed under micoraerophilic conditions such as those found in the respiratory secretions of patients with CF [3].

## Denitrification and arginine utilization pathways

The results of the INSeq analysis were consistent with the expected growth of *P. aeruginosa* in the GI tract under conditions of reduced oxygen making the cells extensively reliant on denitrification for energy generation, transferring electrons from nitrate through nitrite, nitric oxide, nitrous oxide and finally to N_2_. These reactions are catalyzed by the nitrate reductase NarGHI, nitrite reductase NirS, nitric oxide reductase NorCB, and nitrous oxide reductase NosZ, respectively. Strains with Tn insertions in operons containing the *nar*, *nir*, *nor,* and *nos* genes encoding electron transfer, cofactor biosynthesis, regulatory and assembly factors all showed reduced fitness for colonization of the ceca (Figure 6). Interestingly, *P. aeruginosa* can express two nitrate reductases, a membrane bound NarGHI and a periplasmic NapAB and only the membrane bound enzyme is required for anaerobic growth *in vitro* [5]. However, insertions in *napAB* were also attenuated showing reduced *in vivo* fitness, suggesting that this enzyme either contributes to anaerobic growth *in vivo* or has another unspecified function needed for maximal *P. aeruginosa* colonization. Finally, another benefit of an induced denitrification pathway is the ability of *P. aeruginosa* to detoxify nitric oxide (NO) produced by the host immune defense systems, utilizing the NO reductase NorCB[6]. This may account for the attenuation of Tn insertions in denitrification pathway genes during cecal colonization. In *P. aeruginosa*, energy can also be derived from utilization of arginine via the ADI pathway, which breaks down arginine to ammonia and carbon dioxide. The components of this pathway include ArcD (arginine/ornithine antiporter), ArcA (arginine deaminase), ArcB (ornithine carbamoyltransferase) and ArcC (carbamate kinase). The *arcABC* genes were necessary for GI colonization of mice, whereas insertions into the *arc*D gene appeared to be necessary only for systemic dissemination (Figure 6). ArcD allows an exchange of cellular arginine for external ornithine without consumption of energy [7], a process apparently unnecessary for bacterial survival in cecal samples (Figure 6).

## Ion pumps and redox reactions

A group of genes with Tn insertions that showed reduced *in vivo* fitness were in the homologs of the *rnf* genes (*rnfA-E,* PA14_18890-950) previously described as ion pumps that couple redox reactions to the translocation of Na^+^ across the cell membrane. It has been shown that in *Rhodobacter capsulatus* the Rnf proteins transport electrons from NADH to ferredoxin and are essential for nitrogen fixation. However, many microorganisms carry these genes although they do not fix nitrogen and their products are involved in diverse functions such as Na^+^-dependent NADH-quinone reductase involved in solute import, ATP synthesis, flagellar rotation while transporting sodium cations across the membrane [8], and keeping redox-sensitive transcriptional factors such as SoxR in its reduced (inactive) state during aerobic growth [9]. In *P. aeruginosa*, Tn insertions in all but one of the *rnf* homologs had reduced fitness for cecal colonization. Unexpectedly, insertions in the *rnf*C gene, but not the other *rnf* genes*,* had a strong positive fitness phenotype for systemic dissemination in this component of the infection model.

## Tn insertions in nutritional genes leading to enhanced *in vivo* fitness

Among the operons associated with the use or production of nutrients, there were some individual genes within the operons involved in histidine, malonate and trehalose synthesis that did not follow the overall pattern of decreased fitness for the Tn insertions in all of the other genes in these operons (Figure S8). These included the *hutD* (PA14_67410) gene that is in an operonic configuration with *hut*C (PA14_67420), encoding a known repressor of the utilization of histidine [10], *mdc*B, whose product is involved in malonate assimilation, and *glg*X, part of an operon associated with trehalose utilization (Figure S8A). The basis for these unusual phenotypes is unclear but indicative of the power of INSeq to parse out differential gene contributions for environmental fitness.

## Potential targets for immunotherapy

A potential but speculative application of the INSeq methodology is to combine the identification of annotated genes encoding outer membrane proteins and thus likely to have portions surface exposed, with a determination that they are also essential for virulence. These two factors point to possible vaccines. Genomic technologies can also be used to focus these investigations on important surface proteins that are highly conserved among sequenced genomes. An example of one such target is a very large exoprotein of 531.9 kDa potentially involved in heme utilization and/or adherence encoded by a 15,639 bp gene, PA14_32790, which forms an operon with the neighboring gene PA14_32780 and is present in all sequenced strains of *P. aeruginosa* where they have 88 to 94% genomic sequencing identity. The predicted protein product is similar to the filamentous hemagglutinin component of the *Bordetella pertussis* acellular vaccine. Tn insertions into PA14_32790 were able to colonize the ceca but unable to disseminate to the spleens (Figure S9) indicative of a target likely essential for serious *P. aeruginosa* infections. Similar potential vaccine targets based on their surface exposure and essentiality in virulence are the three different putative TonB-dependent receptors encoded by *cirA, optS* and PA14_37490 (Table S3).

# References

1. Williams HD, Zlosnik JE, Ryall B (2007) Oxygen, cyanide and energy generation in the cystic fibrosis pathogen *Pseudomonas aeruginosa*. Adv Microb Physiol 52: 1-71.

2. Itoh Y, Nakada Y (2004) Arginine and polyamine metabolism. In: Ramos J-L, editor. Pseudomonas. New York: Springer-Verlag. pp. 243-372.

3. Alvarez-Ortega C, Harwood CS (2007) Responses of *Pseudomonas aeruginosa* to low oxygen indicate that growth in the cystic fibrosis lung is by aerobic respiration. Mol Microbiol 65: 153-165.

4. Kawakami T, Kuroki M, Ishii M, Igarashi Y, Arai H (2010) Differential expression of multiple terminal oxidases for aerobic respiration in *Pseudomonas aeruginosa*. Environ Microbiol 12: 1399-1412.

5. Schreiber K, Krieger R, Benkert B, Eschbach M, Arai H, et al. (2007) The anaerobic regulatory network required for *Pseudomonas aeruginosa* nitrate respiration. J Bacteriol 189: 4310-4314.

6. Arai H (2011) Regulation andfunction of versatile aerobic and anaerobic respiratory metabolism in *Pseudomonas aeruginosa*. Front Microbiol 2: 103.

7. Verhoogt HJ, Smit H, Abee T, Gamper M, Driessen AJ, et al. (1992) *arcD,* the first gene of the arc operon for anaerobic arginine catabolism in *Pseudomonas aeruginosa*, encodes an arginine-ornithine exchanger. J Bacteriol 174: 1568-1573.

8. Steuber J (2001) Na(+) translocation by bacterial NADH:quinone oxidoreductases: an extension to the complex-I family of primary redox pumps. Biochim Biophys Acta 1505: 45-56.

9. Koo MS, Lee JH, Rah SY, Yeo WS, Lee JW, et al. (2003) A reducing system of the superoxide sensor SoxR in *Escherichia coli*. EMBO J 22: 2614-2622.

10. Allison SL, Phillips AT (1990) Nucleotide sequence of the gene encoding the repressor for the histidine utilization genes of *Pseudomonas putida*. J Bacteriol 172: 5470-5476.
